# Population structure and adaptability analysis of *Schizothorax o’connori* based on whole-genome resequencing

**DOI:** 10.1186/s12864-024-09975-9

**Published:** 2024-02-06

**Authors:** Kuo Gao, Zhi He, Jinxin Xiong, Qiqi Chen, Bolin Lai, Fei Liu, Ping Chen, Mingqiang Chen, Wenjie Luo, Junjie Huang, Wenxiang Ding, Haochen Wang, Yong Pu, Li Zheng, Yuanyuan Jiao, Mingwang Zhang, Ziting Tang, Qingsong Yue, Deying Yang, Taiming Yan

**Affiliations:** 1 College of Animal Science and Technology, Sichuan Agricultural University, Chengdu, China; 2 Huadian Tibet Hydropower Development Co.,Ltd, Dagu Hydropower Station, Sangri, 856200 Shannan China

**Keywords:** *Schizothorax o’connori*, Genetic diversity, Environmental adaptation, Whole genome resequencing, Yarlung Zangbo River

## Abstract

**Background:**

*Schizothorax o’connori* is an endemic fish distributed in the upper and lower reaches of the Yarlung Zangbo River in China. It has experienced a fourth round of whole gene replication events and is a good model for exploring the genetic differentiation and environmental adaptability of fish in the Qinghai-Tibet Plateau. The uplift of the Qinghai-Tibet Plateau has led to changes in the river system, thereby affecting gene exchange and population differentiation between fish populations. With the release of fish whole genome data, whole genome resequencing has been widely used in genetic evolutionary analysis and screening of selected genes in fish, which can better elucidate the genetic basis and molecular environmental adaptation mechanisms of fish. Therefore, our purpose of this study was to understand the population structure and adaptive characteristics of *S. o’connori* using the whole-genome resequencing method*.*

**Results:**

The results showed that 23,602,746 SNPs were identified from seven populations, mostly distributed on chromosomes 2 and 23. There was no significant genetic differentiation between the populations, and the genetic diversity was relatively low. However, the Zangga population could be separated from the Bomi, Linzhi, and Milin populations in the cluster analysis. Based on historical dynamics analysis of the population, the size of the ancestral population of *S. o’connori* was affected by the late accelerated uplift of the Qinghai Tibet Plateau and the Fourth Glacial Age. The selected sites were mostly enriched in pathways related to DNA repair and energy metabolism.

**Conclusion:**

Overall, the whole-genome resequencing analysis provides valuable insights into the population structure and adaptive characteristics of *S. o’connori*. There was no obvious genetic differentiation at the genome level between the *S. o’connori* populations upstream and downstream of the Yarlung Zangbo River. The current distribution pattern and genetic diversity are influenced by the late accelerated uplift of the Qinghai Tibet Plateau and the Fourth Ice Age. The selected sites of *S. o’connori* are enriched in the energy metabolism and DNA repair pathways to adapt to the low temperature and strong ultraviolet radiation environment at high altitude.

**Supplementary Information:**

The online version contains supplementary material available at 10.1186/s12864-024-09975-9.

## Background

The Qinghai-Tibet Plateau, known as the roof of the world, has unique environmental conditions, including high altitude, low temperature, large temperature difference and high ultraviolet radiation, which make the unique organisms in this area ideal models for studying the molecular mechanism of plateau adaptability [1–7]. Studies have shown that fish in the Qinghai-Tibet Plateau have undergone adaptive evolution [8–11]. Compared with other fish, their evolutionary rates are significantly accelerated in Gene Ontology (GO) terms such as antioxidant stress, vascular morphogenesis, glucose metabolism, and DNA repair. Many genes related to the hypoxia response and energy metabolism were selected [12–16]. As a dominant group, the distribution and evolution of Schizothoracinae fishes were closely related to the uplift of the Qinghai-Tibet Plateau, which has attracted attention to the fields of phylogenetic, evolutionary and extreme environmental adaptation mechanisms [17–19]. Previous studies have shown that Schizothoracinae fishes form a new species adaptive ecological niche in the form of sympatric speciation or allopatric speciation under geographical barriers and natural selection, but there are few empirical cases and further exploration is needed [20, 21].

*Schizothorax o’connori* is an endemic species in China that is widely distributed in the Yarlung Zangbo River and has great ecological and economic value. Previous studies have found that the uplift of the Tibetan Plateau has led to changes in the water system of the plateau rivers, and the Palong Zangbo River, a tributary of the lower Yarlung Zangbo River, may have been formed due to river capture [22, 23]. The Palong Zangbo River population of *S. o’connori* may also originate from the upper reaches of the Yarlung Zangbo River Grand Canyon, but there is no evidence of gene exchange [22]. As a young tetraploid fish, *S. o’connori* experienced the fourth round of genome-wide replication recently, and its evolution rate was higher than that of most Schizothorax fishes [1, 24]. Because of the existence of Yarlung Zangbo Grand Canyon, whether there is a difference of the *S. o’connori* population in the upper and lower reaches of the Grand Canyon, and *S. o’connori* has evolved independently in different water environments to adapt to the environment is not clear. Furthmore, due to the intensification of human activities, invasion of alien species and construction of water conservancy and hydropower projects, the natural population of *S. o’connori* has decreased sharply in recent years [25–27]. At present, research on *S. o’connori* has mainly focused on age and growth, liver metabolism and pigment distribution [28–32]. However, there is no genome-wide study on the population genetics of *S. o’connori*, which was based only on mtDNA sequences and microsatellite markers to study its genetic diversity [22, 23]. Therefore, it is necessary to further explore the differentiation evidence of *S. o’connori* at the genomic level, providing a reference basis for the parallel evolution research of plateau fish.

In recent years, with the publication of more fish genome-wide data, whole genome resequencing has been widely used. It not only analyses the genetic diversity, phylogenetic relationship and population historical dynamics of different fish populations [33–35], but also screens out the genes and pathways related to environmental adaptation, which could also better elucidate the genetic basis and molecular mechanism of environmental adaptation of fish [36–40]. Whole-genome data for *S. o’connori* have been published, and the genome and transcriptome sequencing data were submitted to the National Biotechnology Information Center (NCBI) biological project number PRJNA557578 [24]. In the present study, the genetic diversity, population structure and environmental adaptation mechanism of seven wild populations of *S. o’connori* in the middle and lower reaches of the Yarlung Zangbo River were analysed by whole-genome resequencing. The results of this study can provide an important basis for resource protection and environmental adaptation of *S. o’connori*.

## Results

### Data quality control

From the Illumina NovaSeq 6000 sequencing platform, a total of 849.5 Gb raw reads and 803.9 Gb clean reads were obtained from 53 samples. The average sequencing depth was 10.99 X. The average depths of 1X, 5X, and 10X were 84.89%, 65.46%, and 43.65%, respectively. Xiao et al. [24] shared the assembly results and annotation information of the genome sequencing data with us, which was referred to as the reference genome of *S. o’connori*. Then, the average MapPERate ratio of reads to the reference sequence for comparison was 93.27%, and the average ProperlyRate ratio of reads to the reference sequence for correct alignment (insertion of fragments, direction, etc.) was 78.59%. The average coverage rate (coverage) of the reference sequence was 90.72%. The average Q20 was 97.40%, and the average Q30 was 91.42% (Supplementary Table 1).

### Cluster analysis of differential SNPs and InDels

**Fig. 1:**
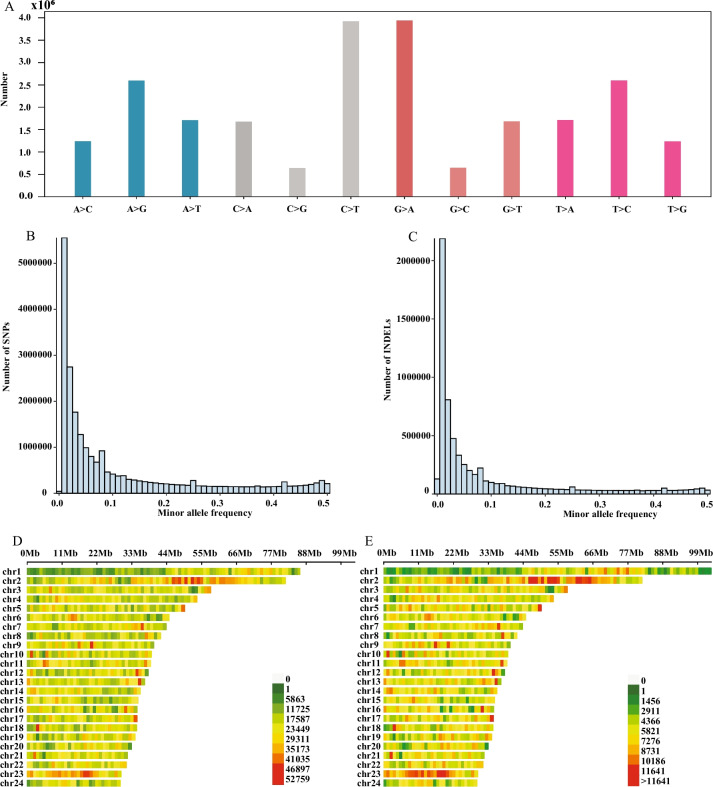
SNP and InDel information. **A**. SNP marker type distribution; **B**. Minor allele frequency (MAF) distribution of SNPs; **C**. MAF distribution of InDels; **D**. Density distribution of SNPs within 1 Mb on the chromosome; **E**. Density distribution of InDels within 1 Mb on the chromosome. A, G, C and T represent adenine deoxynucleotides, guanine deoxynucleotides, cytosine deoxynucleotides and thymine deoxynucleotides, respectively. Chr1-24 represents chromosomes 1–24; SNPs represent single nucleotide polymorphisms; InDels represent insertions and deletions; and Mb represents megabase

A total of 23,602,746 SNPs (single nucleotide polymorphisms) and 6,671,199 InDels (insertion/deletion polymorphisms) were identified by mutation detection and screening. The results showed that the transition (AT → GC or GC → AT) was the main mutation type (Fig. 1A). In addition, most SNPs were in intergenic (59.55%) and intron regions (31.97%), followed by exons (3.38%), downstream (2.46%), and upstream regions (2.46%) (Table 1). The minor allele frequency (MAF) analysis showed that SNPs and InDels were mainly distributed on smaller MAFs (Fig. 1B, C). Both SNPs and InDels had high density distributions on chromosome 2 and chromosome 23, which implied that these two chromosomes may play an important role in evolution (Fig. 1D, E).

**Table 1:** Number of SNPs and InDels in different regions of the genome

Type	SNP number	InDel number
Downstream	580,449	189,116
Exons	797,885	87,090
Intergenic	14,054,479	3,971,385
Intronic	7,545,840	2,230,496
Splicing	4068	2942
Upstream	580,359	178,834
Upstream; downstream	23,148	7981
3 prime UTR	7580	1437
5 prime UTR	8938	1917
3 prime UTR; 5 prime UTR	0	1

*SNPs* represent single nucleotide polymorphisms, *InDels* represent insertions and deletions, and *UTRs* represent untranslated regions

### Population genetic diversity analysis

According to SNP data, the values of *H*_*O*_, *H*_*E*_, π and *PIC* were 0.1878–0.2167, 0.1414–0.1638, 0.0019–0.0020 and 0.01143–0.0348, respectively (Table 2). The average values of *H*_*O*_, *H*_*E*_, π and *PIC* in the LZ population were the lowest, indicating that genetic diversity was the lowest. The *H*_*O*_, *H*_*E*_ and *PIC* of the ZG population were larger than those of the other populations, and π was only smaller than that of the ML population, indicating that the ZG population had the highest genetic diversity. The genetic diversity of the DG, JC and ZM populations was similar, while the BM population was closest to that of the DG population.

**Table 2:** Genetic diversity parameters among *Schizothorax o’connori* populations

Population	*H*_*O*_	*H*_*E*_	π	*PIC*
ZG	0.2167	0.1638	0.0019	0.1348
DG	0.2113	0.1607	0.0019	0.1321
ZM	0.2162	0.1624	0.0019	0.1334
JC	0.2084	0.1601	0.0019	0.1317
ML	0.225	0.1548	0.002	0.124
LZ	0.1878	0.1414	0.0018	0.1143
BM	0.2098	0.1608	0.0019	0.1322
Average value	0.2107	0.1577	0.0019	0.1289

*H*_*E*_, expected heterozygosity, *H*_*O*_ observed heterozygosity, *PIC* polymorphism information content, π nucleotide diversity, *ZG* Zangga, *DG* Dagu *ZM* Znagmu, *JC* Jiacha, *ML* Milin, *LZ* Linzhi, *BM* Bomi

The range of the genetic fixation index (*F*_*st*_) between the two populations was 0.0132–0.0437 (Table 3). The *F*_*st*_ of the ML population was relatively higher than that of the other six populations, ranging from 0.0346 to 0.0437. Next was the LZ population, with an *F*_*st*_ between 0.0230 and 0.0437 compared to the other six populations. The genetic differentiation between the seven populations was very weak.

**Table 3:** Genetic fixation index between seven populations of *Schizothorax o’connori*

Population	BM	JC	LZ	ZG	DG	ML	ZM
BM	0	0.0139	0.0233	0.0167	0.0135	0.0339	0.0132
JC	0.0139	0	0.0243	0.0161	0.0137	0.0346	0.0133
LZ	0.0233	0.0243	0	0.0244	0.0237	0.0437	0.0230
ZG	0.0167	0.0161	0.0244	0	0.0155	0.0380	0.0153
DG	0.0135	0.0137	0.0237	0.0155	0	0.0361	0.0133
ML	0.0339	0.0346	0.0437	0.0380	0.0361	0	0.0354
ZM	0.0132	.03133	0.0230	0.023	0.0133	0.0354	0

*BM* Bomi, *JC* Jiacha, *LZ* Linzhi, *ZG* Zangga, *DG* Dagu, *ML* Milin, *ZM* Zangmu

### Systemic development and population structure

**Fig. 2:**
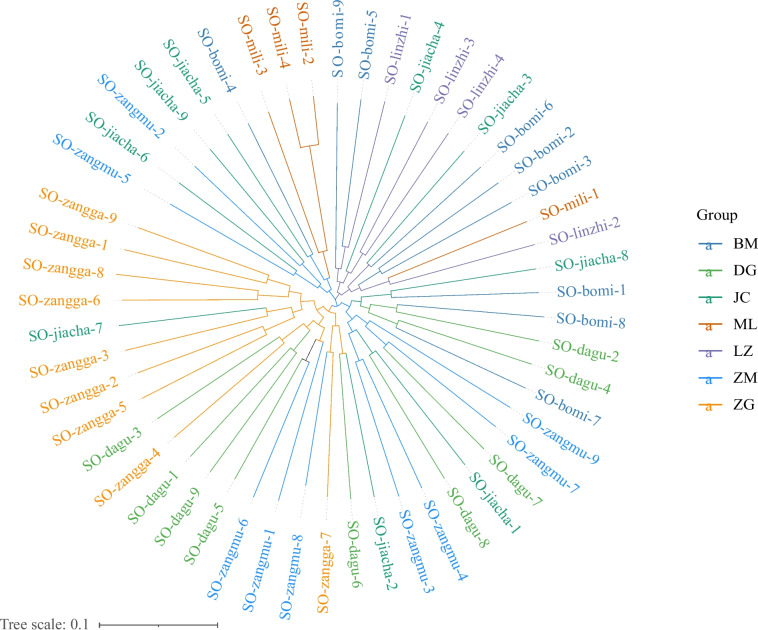
The neighbourhood-joining phylogenetic tree of the *Schizothorax o’connori* system. ZG, Zangga; DG, Dagu; ZM, Znagmu; JC, Jiacha; ML, Milin; LZ, Linzhi; BM, Bomi

According to the NJ phylogenetic tree analysis, the seven populations crossed each other and could not be obviously clustered into a single branch (Fig. 2). However, focusing on each population, the ZG population was completely separated from the BM, ML, and LZ populations, while there was only one individual staggered with the JC population. The ML, LZ and DG populations were completely separated and not clustered on the same branch. The JC and ZM populations were dispersed in other populations, especially the JC populations, which intersected with each population.

Phylogenetic analysis showed that the populations from the upstream (ZG population), downstream (ML and LZ populations), and tributary Palongzangbu (BM population) of the sampling section were differentiated into two genetic lineages. DG, ZM and JC in the middle reaches of the sampling section were scattered in two genetic lineages, especially the JC population. This hinted at a phylogenetic relationship related to geographical location.

**Fig. 3:**
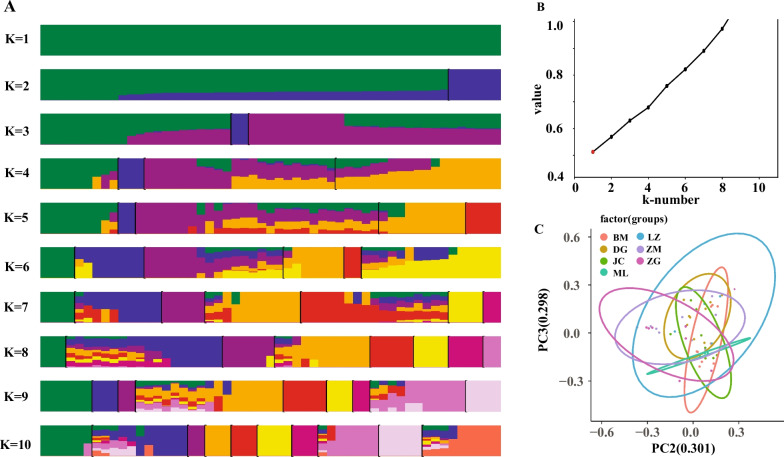
Principal component analysis and the error rate of the *Schizothorax o’connori* admixture K value. **A** The error rate of the *S. o’connori* admixture K value by cross − validation; **B** the clustering results of *S. o’connori*; **C** Principal component analysis for *S. o’connori*. ZG, Zangga; DG, Dagu; ZM, Znagmu; JC, Jiacha; ML, Milin; LZ, Linzhi; BM, Bomi

According to the clustering situation at different K values, the consistency between individuals in different subgroups and the river basin where the sampling point was located was not strong. Only when there was one ancestor (K = 1) could 53 samples be clustered together (Fig. 3A). Furthermore, considering the cross-validation error rate of different K values (Fig. 3B), the results were selected when K = 1 as the clustering criterion; that is, the 53 samples in this experiment were a single population at the level of population genetic structure analysis.

### PCA analysis

PCA showed that the contribution rates of the second principal component (PC2) and the third principal component (PC3) were 30.10% and 29.80%, respectively (Fig. 3C). Then, seven groups could not be separated. According to the output of PCA parameters, the 53 samples of this project could not be clustered into different subgroups at the level of group principal component analysis.

### Gene flow and linkage disequilibrium analysis

**Fig. 4:**
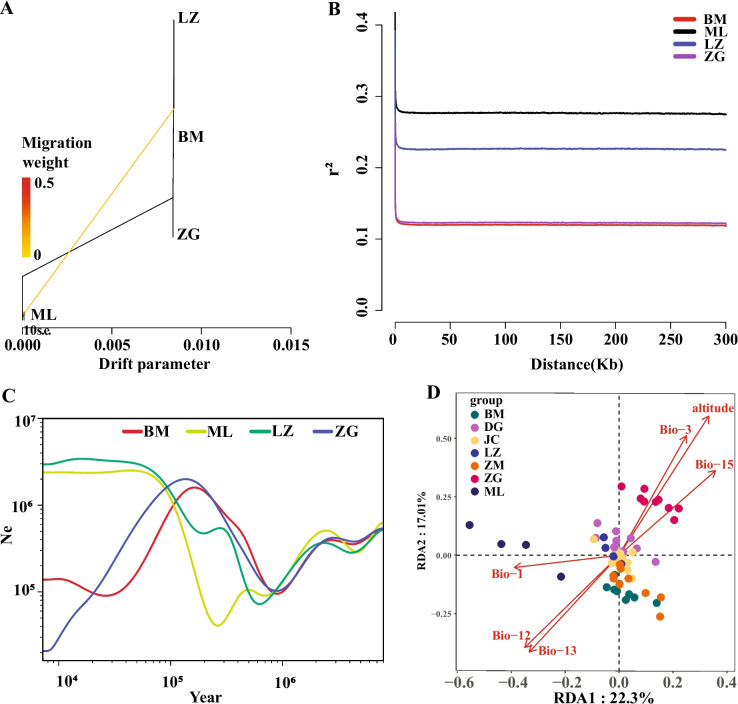
Further analysis of the four groups and redundancy analysis. **A** gene flow analysis; **B** linkage disequilibrium analysis; **C** estimation of historical effective population size for four populations; **D** redundancy analysis based on six factors (Bio1: annual mean temperature; Bio3: isothermality; Bio12: annual precipitation; Bio13: precipitation of wettest month; Bio15: precipitation seasonality and altitude). ZG, Zangga; DG, Dagu; ZM, Znagmu; JC, Jiacha; ML, Milin; LZ, Linzhi; BM, Bomi

Based on the results of genetic differentiation and cluster analysis, only the gene flow and linkage disequilibrium analysis of ZG, BM, LZ and ML were performed here (Fig. 4A). Gene flow analysis showed that when one migration event or two migration events occurred, only the ML population gene flowed to BM among the four populations, and no gene flow occurred among other populations, which was similar to the results of cluster analysis. This implies that the ML and BM populations originated from a single ancestor.

LD analysis was conducted for four populations. The LD coefficients of the BM and ZG populations at 0.8 kb on the genome were approximately 0.13, the ML at 2.2 kb on the genome was approximately 0.28, and the LZ at 1.8 kb on the genome was approximately 0.23 (Fig. 4B). The decay rate of the LD coefficient in the four populations was BM = ZG > LZ > ML. The decay distance of the BM and ZG populations was the smallest, which may be due to the high genetic diversity of these two populations. This was consistent with the results of the genetic diversity analysis.

### Effective population size analysis

The historical effective population size and variation trend of ML and LZ populations were basically consistent, and the BM and ZG populations were consistent (Fig. 4C). About 8.0 million years ago, the effective population size of ML, LZ, BM, and ZG populations was still at a relatively high level. However, approximately 8.0–1.0 million years ago, the overall population showed a downward trend. The BM, ZG, LZ and ML populations experienced rapid expansion at approximately 1.0–0.13 Ma, 1.0–0.13 Ma, 0.18–0.019 Ma and 0.14–0.019 Ma, respectively. Then, the effective population size of the BM and ML remained almost unchanged from approximately 0.019 to 0.01 Ma. Conversely, the ZG and BM populations rapidly decreased from approximately 0.013 to 0.01 Ma and 0.13 to 0.015 Ma, respectively. Otherwise, the BM population slowly expanded from 0.015 to 0.01 Ma.

### Genome-environment association analysis and functional annotation of selected SNPs

Five climatic factors (Bio1: annual mean temperature; Bio3: isothermality; Bio12: annual precipitation; Bio13: precipitation of wettest month; Bio15: precipitation seasonality) and altitude factors were selected for genome-environment association analysis (GEA). The results showed that the variance explanatory rates of the first two components were 22.30% and 17.01%, respectively, while the six factors revealed the significant statistical differences with genotype (*P* = 0.001, *R*^*2*^*adj* = 0.3277) (Fig. 4D). Among them, altitude factors have the greatest correlation with population genetic distribution. On the plateau, altitude was closely related to temperature and ultraviolet radiation, which provides a reference for the analysis of selected sites in the future.

**Fig. 5:**
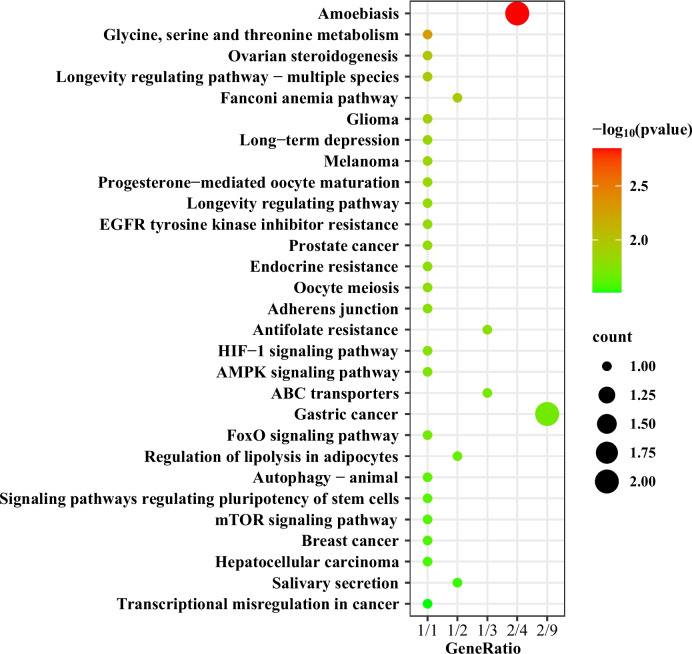
The top 30 KEGG pathways significantly enriched in *Schizothorax o’connori*

Based on lnθ_π_ ratio and *F*_*ST*_ value, the functional annotation of selected SNPs was analysed through the KEGG signalling pathway and GO terms (*p* ≤ 0.05) (Fig. 5, Table 4). In the pairwise comparison of all populations, 77 significant KEGG pathways were identified, and the most common pathways contained olfactory transduction, the Fanconi anaemia pathway, and glycine serine and threonine metabolism (Supplementary Table 2).

**Table 4:** Significantly selected pathways and genes among *Schizothorax o’connori* populations

Signalling Pathway	Number of occurrences	Enriched genes
Olfactory transduction	10	Soc_16G0006340 Soc_19G0002040
Fanconi anaemia pathway	7	Soc_20G0005860
Glycine, serine and threonine metabolism	6	Soc_14G0000910
Amoebiasis	5	Soc_20G0005900 Soc_20G0005830
Signalling pathways regulating pluripotency of stem cells	3	Soc_14G0000300 Soc_3G0009700
Apoptosis—multiple species	2	Soc_16G0007700
Platinum drug resistance	2	Soc_17G0007270
Long-term depression	2	Soc_3G0009700 Soc_19G0002040
ABC transporters	2	Soc_17G0007270
Antifolate resistance	2	Soc_17G0007270

A total of 86 significant GO terms were determined from pairwise comparisons of all populations, such as olfactory receptor activity, cellular protein modification process, peptidase activity and ubiquitin-protein transferase activity (Supplementary Table 3).

### Extended haplotype homozygosity between the populations

**Fig. 6:**
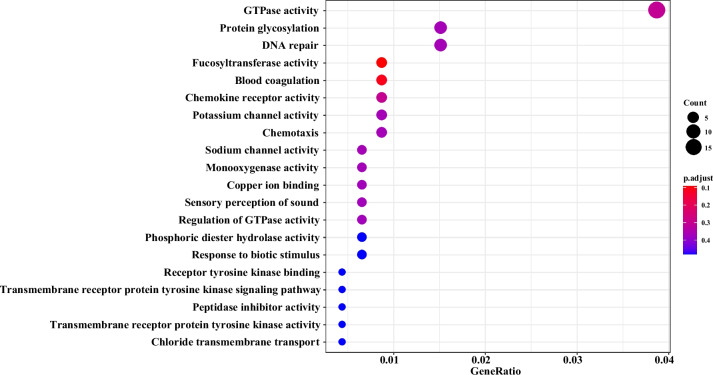
GO functional enrichment results of candidate genes in the Bomi (BM) vs. Milin (ML) populations

**Fig. 7:**
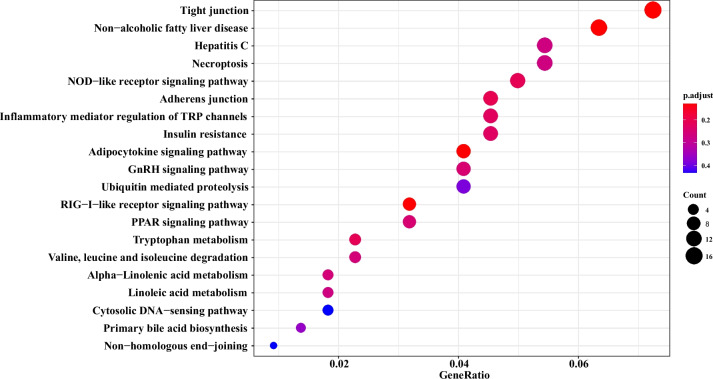
KEGG functional enrichments of candidate genes in the Bomi (BM) vs. Milin (ML) populations

Based on the XP-EHH values of each locus combined with *p* values, the top 5 regions were selected as candidate regions. The candidate genes in this region were enriched and analysed (Supplementary Table 4 and 5). The analysis result of XP-EHH was similar to that of selective clear analysis. The selected items were mostly related to DNA repair and energy metabolism. Only one example is shown here. Comparing the BM and ML populations, more than 700 candidate genes were extracted in the top 5 regions. The KEGG and GO functional enrichment items mostly included fucosyltransferase activity, nucleosome, endopeptidase inhibitor activity, and nucleosome assembly (Figs. 6 and 7).

## Discussion

Maintaining genetic diversity within species and populations is important for their long-term survival and health, as it provides the ability to adapt and change according to environmental conditions [41–44]. The average level of heterozygosity and genetic diversity is positively correlated with environmental adaptability [43, 45]. In this study, the π of the seven populations of *S. o’connori* was low, and the *PIC* belonged to weak polymorphism sites. However, *H*_*O*_ (0.2107) and *H*_*E*_ (0.1577) were higher than those in *S. kozlovi* (*H*_*O*_ 0.09578 and *H*_*E*_ 0.06743) [46] but lower than those in most schizothoracids, such as *S. lissolabiatus* (*H*_*O*_ 0.2695 and *H*_*E*_ 0.2892) [47] and *S. curvilabiatus* (*H*_*O*_ 0.2489 and *H*_*E*_ 0.2931) [48]. This may be because *S. o’connori* has been in a stable state after experiencing bottleneck effects and has not undergone population expansion, thus exhibiting a low pattern of genetic diversity [49–52]. This was similar to the reason for genetic diversity scarcity in the *Schizopygopsis pylzovi* population [53]. In addition, changes in the aquatic environment and the decrease in population resources caused by overfishing might also be the reasons for the low genetic diversity of *S. o’connori* [25, 26]. Therefore, it is necessary to increase monitoring and protection of *S. o’connori*, especially the LZ population.

*Fst* and cluster analysis can determine the level of genetic differentiation and genetic relationships between different populations [54, 55]. Natural fish populations are gradually differentiated due to gene exchange between populations because of geographical isolation. For example, there was no barrier between the habitats of the Taiwan and Guangdong populations of the oblique banded grouper, but the straight-line distance was approximately 600 km, indicating geographical differentiation between the two populations [56]. Based on the classification standard of *Fst* values, the genetic differentiation between the seven populations of *S. o’connori* was relatively small (0 < *Fst* < 0.05). However, the ZG population was completely separated from the LZ, ML and BM populations but intersected with DG, ZM and JC, which may be due to geographical isolation. In this study, the ZG population was approximately 340 km away from the ML population and 630 km away from the BM population. There were differences in the habitat environment between different populations at long distances and a long-term lack of communication, leading to such population differentiation. Although there was a dam barrier between ZG and DG and between ZM and JC, the dam construction time was relatively short (construction started between 2010 and 2020) [57]. Thus, it was not the current cause of group differentiation, but it may lead to group differentiation upstream and downstream of the dam in the long term.

Numerous studies have shown that ancient climate and geological events might be important factors affecting the current geographical distribution and genetic differentiation of species in the Qinghai-Tibet Plateau [58–61]. In this study, the effective population of *S. o’connori* decreased for a short time in the late accelerated uplift stage of the Qinghai Tibet Plateau (~ 1 million years ago) and the Quaternary Ice Age [24, 62, 63]. Therefore, we speculate that the geological movement and temperature decrease during this period brought great pressure on *S. o’connori* survival, which led to a sharp decline in the population. In addition, the Palong Zangbo River may not be a tributary of the lower reaches of the Yarlung Zangbo River approximately 4 megaannus (Ma) ago but a part of the main stream of the lower reaches [23]. Before the ancient Yarlung Zangbo River was captured, Palong Zangbo belonged to the lower reaches of the main stream of Yarlung Zangbo River, which was connected with the upper reaches of the Grand Canyon, so there was extensive gene exchange among *S. o’connori* in this section without geographical barriers [23, 64–66]. With the formation of the Grand Canyon, geographical barriers, such as waterfalls, allowed populations upstream of the adjacent Grand Canyon to flow downstream and undergo gene exchange with downstream populations. However, the Palong Zangbo River population could not undergo gene exchange upstream of the Grand Canyon [23, 67], which greatly explains the communication phenomenon between the BM and ML populations in this gene flow and evolutionary tree analysis. Meanwhile, this also confirms that our hypothesis that the Parlung Tsangpo colony population (Bomi) evolved independently was not valid.

In addition, the *Cytb* + Control region sequences were utilized to explore the genetic results of different *S. o’connori* populations, and significant genetic differentiation was found between the BM population and the upstream Grand Canyon population [22]. However, this study found that the BM population only showed differentiation from the ZG population at the genomic level, suggesting that the genetic differentiation of different populations at the molecular level was gradual.

Revealing the genetic basis of plateau fish for extreme environments could further our understanding of their adaptive evolution under environmental changes [68–70]. In this study, we utilized selective clearance analysis and XP-EHH to determine the potential features of high-altitude adaptation in *S. o’connori*. Based on the two analysis methods, the selected SNP sites were mostly concentrated in pathways such as olfactory transduction, the Fanconi anaemia pathway, and amino acid metabolism, which were mostly related to DNA repair and energy metabolism.

In extremely high-altitude environments, both low temperature and ultraviolet radiation can cause DNA damage [71–74]. DNA damage repair plays an important role in maintaining DNA integrity and stability. The GO enrichment analysis of positively selected genes in *S. malacanthus* and *S. pylzovi* was mainly related to DNA repair, which helps them adapt to high altitude and strong ultraviolet radiation environments [75]. Pathways related to DNA repair, such as homologous recombination and the P53 signalling pathway, were selected in *Trilophysa bleekeri*, forming an integrated DNA repair mechanism to cope with extremely high-altitude environments [76]. The reptiles [77], mammals [78], and birds [79] living on the Qinghai Tibet Plateau have also undergone adaptive evolution in DNA repair. The above results suggest that high-altitude animals have similar adaptive convergent evolution in terms of DNA damage repair function.

Fish undergo adaptive evolution for energy metabolism in environments with low water temperatures and large temperature differences between day and night throughout the year [80–82]. *Gymnodiptychus pachycheilus* exhibits accelerated genome evolution, and genes exhibiting rapid evolution and positive selection characteristics in its lineage enrich functions related to energy metabolism [83]. Compared to plain fish such as *Ctenopharyngodon idellus* at different altitudes, the dN/dS values of all schizothoracine fishes were significantly increased, and the evolution rate of some GO items related to energy metabolism, hypoxia response, and DNA repair related to altitude adaptation was also significantly accelerated [8]. Thus, the fish on the Qinghai-Tibet Plateau have undergone a rapid evolutionary process. Fish living in other cold regions have similar evolutionary strategies. For example, to adapt to the cold and highly seasonal Antarctic environment, transposable elements from intergenic Antarctic krill have expanded and formed a large genome; moreover, the gene family related to moulting and energy metabolism has also expanded [84]. In this study, we found that the selected genes of *S. o’connori* were enriched in pathways such as amino acid metabolism and sugar metabolism. Amino acid and sugar metabolism are important metabolic pathways that play an essential role in regulating metabolism and maintaining energy needs. This signifies that *S. o’connori* has undergone adaptive evolution in high-altitude, low-temperature environments.

Determining conservation order and units based on the genetic diversity of different species populations is essential for proposing targeted conservation strategies [85]. It is generally believed that populations with higher diversity have greater evolutionary potential, better adaptability to environmental changes, and higher conservation value [22]. In this study, seven populations of *S. o’connori* did not have significant differentiation. However, in the evolutionary tree analysis, the ML, LZ and BM populations were more concentrated, and the other four populations were less concentrated. Therefore, we propose to divide seven populations into two management units: the BM, LZ, and ML groups near Grand Canyon as one management unit and the ZG, DG, ZM, and JC groups as another management unit. Moreover, among the seven populations, the ZG population had the highest genetic diversity and could be prioritized for protection. We suggest establishing small-scale *S. o’connori* reserves, including strengthening fishery supervision and habitat assessment in these areas [86]. For other populations, measures against catching and carrying out fish stocking and other related work should be strictly implemented, which is crucial to the effective population size and genetic variation.

## Conclusion

In this study, we analysed the population structure and adaptive characteristics of *S. o’connori* upstream and downstream of the Yarlung Zangbo Grand Canyon. There was no significant genetic differentiation between the seven populations, but in cluster analysis, the upstream Zangga population and the downstream Milin, Linzhi, and Bomi populations could be separated, indicating that geographical distance and waterfall impacted the genetic differentiation of the populations. The Bomi population was more closely related to the Milin and Linzhi populations, which may be due to the accelerated uplift of the Qinghai Tibet Plateau in the later stage and the impact of the Fourth Ice Age, which caused the Bomi population to separate from the mainstream and become a tributary population. In addition, the enrichment of selected sites and energy metabolism and DNA repair pathways help *S. o’connori* adapt to the low temperature and strong ultraviolet radiation environment at high altitude. These results provide a basis for the resource protection and adaptability of *S. o’connori*.

## Materials and methods

### Sample collection

**Fig. 8:**
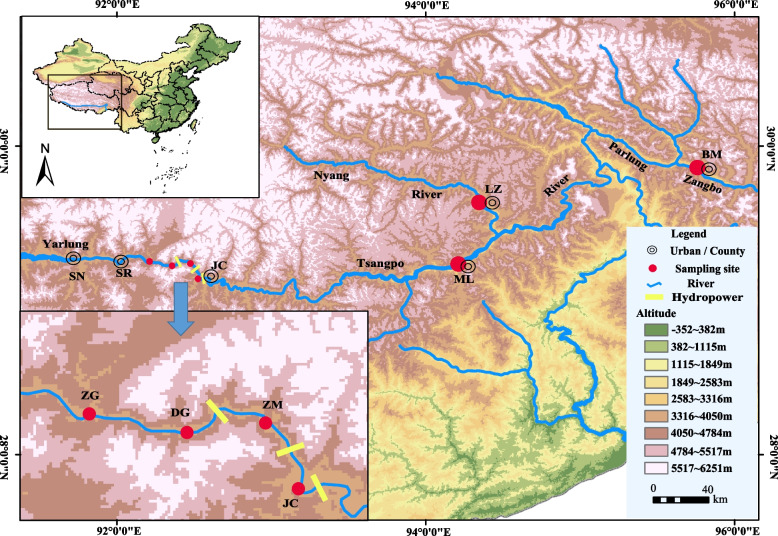
Sampling locations of *Schizothorax o’connori* in the Yarlung Zangbo River. SN, Shannan; SR, Sangri; ZG, Zangga; DG, Dagu; ZM, Zangmu; JC, Jiacha; ML, Milin; LZ, Linzhi; BM, Bomi

From May to July 2022, 53 samples of *S. o’connori* were collected from seven sampling sites in the middle reaches of the Yarlung Zangbo River, including the Zangga Village section (ZG), Dagu Power Hydropower Station reservoir section (DG), Zangmu Power Hydropower Station reservoir section (ZM), Jiacha Hydropower Station reservoir section (JC), Milin section (ML), Linzhi section (LZ) and tributary Palong Zangbo Bomi section (BM). The sample information is shown in Table 5 and Fig. 8. All fins were stored in 95% ethanol and kept in a refrigerator at -20 °C.

**Table 5:** Information on *Schizothorax o’connori* samples

Population	Coordinates	Altitude(m)	Sample Number
ZG	E 92°12′43.55" N 29°15′5.47"	3525.340	9
DG	E 92°21′30.96" N 29°13′24.92"	3488.712	9
ZM	E 92°28′36.49" N 29°14′16.42"	3301.608	9
JC	E 92°31′33.69" N 29°8′22.16"	3223.796	9
LZ	E 94°21′11.39" N 29°38′26.52"	2981.009	4
BM	E 95°45′51.12" N 29°51′41.24"	2724.141	9
ML	E 94°12′40.94" N 29°13′18.64"	2976.733	4

*ZG* Zangga, *DG* Zangga, *ZM* Zangmu Hydropower Station Reservoir Area, *JC* Jiacha Hydropower Station Reservoir Area, *LZ* Linzhi, *BM* Bomi, *ML* Milin

### Whole-genome resequencing

DNA was isolated from the tissues and evaluated by 1% agarose gel electrophoresis. DNA purity and concentration were detected by a Nano Photometer Spectrophotometer and Qubit 2.0 Fluorometer, respectively. Qualified DNA fragmentation was performed using an ultrasonic processor, and the length of the inserted fragment was approximately 350 bp. Then, terminal repair, base A addition, sequencing adapter addition, purification and polymerase chain reaction (PCR) amplification were performed to complete the preparation of the 350 bp library. Subsequently, the library concentration was diluted to 1 ng/μL by Qubit 2.0, and Agilent 2100 was used to detect the size of the inserted fragments in the library. Then, real-time quantitative polymerase chain reaction (RT‒qPCR) was implemented to ensure the effective quantitative concentrations of the library. When the concentration of q-PCR in the library was greater than 3 nmol/L, it was considered an effective quantitative concentration. Finally, the DNA genome was sequenced by the Illumina NovaSeq 6000 platform according to the PE150 strategy at Wuhan Wanmo Technology Co., Ltd.

### Read alignment and SNP calling

Fastp software [87] was used to perform quality filtering on raw data generated by high-throughput sequencing to obtain clean reads that could be used for analysis. Then, using BWA (Version: 0.7.12) [88], clean reads were aligned to the reference genome, and the alignment algorithm was bwa mem. The reference genome and annotation information were provided by Xiao et al., who published the whole genome of *S. o’connori* [24]. The comparison results were formatted and sorted using SAMtools software (Version: 1.9) [89] and then marked with duplicate reads using MarkDuplicates in Picard software (Version: 2.18.17) [90]. The results were compared and statistically analysed using Qualimap software [91].

Based on the comparison results between the sample and the reference genome, SNP and InDel detection were performed using the Haplotypecall module of GATK software (Version: 3.8.1) [92], generating gvcf files for each sample. Then, population SNP and InDel detection was performed using the GenotypeGVCFs module, and the obtained population SNP and InDel data were strictly filtered and screened. The parameter indicators are as follows [34, 93–95]: (1) GATK filtering parameters: QD < 2.0 | MQ < 40.0 | FS > 60.0 | SOR > 3.0 | MQRankSum < -12.5 | ReadPosRankSum < -8.0, sites that met any indicator were excluded; (2) Allelic type: SNP loci are generally of second allelic genotype, so we filtered out loci with two or more completely different genotypes; (3) minor allele frequency (MAF): We removed SNP sites with MAF less than 0.05. The filtered SNP data were annotated using SnpEff (Version 4.3 T) [96] software combined with annotation information from the reference genome.

### Genetic diversity analysis

The parameters of genetic diversity were calculated using VCFTOOLS4.0 [97], including nucleotide diversity (π), observed heterozygosity (*Ho*), expected heterozygosity (*H*_*E*_), and polymorphism information content (*PIC*). The genetic differentiation index (*F*_*st*_) and analysis of molecular variance (AMOVA) between populations were calculated using ARLEQUIN3.5.1.3 [98].

### Phylogenetic analysis

Using PLINK [99] to filter the SNPs of the entire genome for linkage disequilibrium (LD), the parameter was “- independent airwise 50 10 0.2”, and there was no tight selection chain-linked SNPs. Subsequent phylogenetic tree analysis, principal component analysis, and population structure analysis were conducted based on these SNPs.

ADMIXTURE [100] software was used to perform population structure analysis from K = 1 to K = 10, 10 different seeds were selected for 10 repeated analyses, and then the results were clustered 10 times using pong [101]. The optimal K value was determined based on cross-validation error (CV). The optimal number of clusters was determined according to the valley value of the cross-validation error rate. Population gene exchange and differentiation were analysed using TreeMix V1.12 [102].

A neighbour-joining method (NJ) phylogenetic tree was constructed using MEGA v11 [103, 104]. The distance matrix was calculated using TreeBest software [105], and the reliability of the NJ tree was tested using the bootstrap method (repeated 1000 times) [106]. Based on SNP markers, principal component analysis (PCA) was performed using Plink software to obtain the two most influential feature vectors [99].

### Linkage disequilibrium and population historical dynamics analysis

PopLDdecay (Version 3.40) [107] was used for linkage disequilibrium (LD) analysis. The parameters were -MaxDist 500 and -MAF 0.05, and the other parameters were default parameters. PSMC software [108] was utilized to analyse the population dynamic history. The parameters of PSMC were as follows: -N 30–T 5–R 5–P, "4 + 30 * 2 + 4 + 6 + 10", generation G was 2 years, and the base mutation rate μ was 2.5 × 10^–8^.

### Analysis of genome-environment association and selective sweep regions

From Worldclim (https://www.worldclim.org/data/worldclim21.html) download 19 climate data, and then calculate the Spearman correlation coefficient between 19 factors. Once the correlation between two variables was larger than 0.8, one of them was removed [109]. This study used redundancy analysis (RDA) for environmental correlation analysis, which was executed by the *rda* function in the VEGAN software package (Version: 2.5) [91, 109–111].

A combination of lnθπ ratio and *F*_*ST*_ value screened the selected genomic regions. VCFtools software [112] calculated the lnθπ ratio and *F*_*ST*_ value using a sliding window, with a window size of 100 kb and a step size of 10 kb. Select the regions that meet both the top 5% and bottom 5% of the lnθπ ratio, as well as the top 5% of the *F*_*ST*_ value, as the selected regions, and jointly screen for stronger selection signals to obtain the target gene. In addition, the R package rehh was used to detect the gene segments with differentiation between populations using the cross-population extended haplotype homozygosity (XP-EHH) method [113], and the sites with *p* < 0.05 after false discovery rate (FDR) correction were considered the selected sites.

The selected sites were compared to 6 databases for annotation, including Nonredundant (NR), Nucleotide sequences (NT), Universal Protein (UniProt), Kyoto Encyclopedia of Genes and Genomes (KEGG), Gene Ontology (GO) and Evolutionary genealogy of genes: Nonsupervised Orthologous Groups (EggNOG).

For functional enrichment analysis, all selected SNPs were mapped to terms in the GO and KEGG databases [114–116]. Then, with *p* < 0.05 as the threshold, the significantly enriched GO terms and KEGG pathways were searched in the selected SNPs.

### Supplementary Information

**Additional file 1: Supplementary Table 1.** Statistical table of quality control results.

**Additional file 2: Supplementary Table 2.** GO terms of loci under selection among each comparison population of Schizothorax o’connori.

**Additional file 3: Supplementary Table 3.** KEGG pathways of loci under selection among each comparison population of Schizothorax.

**Additional file 4: Supplementary Table 4.** GO annotation for XP-EHH analysis.

**Additional file 5: Supplementary Table 5.** KEGG annotation for XP-EHH analysis.

## Data Availability

The datasets analysed during the current study are available in the Sequence Read Archive under accession number PRJNA1007290. ( https://submit.ncbi.nlm.nih.gov/subs/bioproject/SUB13776086/overview).
