# Supplementary material for: Population structure and adaptability analysis of Schizothorax o’connori based on whole-genome resequencing
Source: BMC Genomics. 2024 Feb 6;25:145. doi: 10.1186/s12864-024-09975-9 (PMC10845765; doi:10.1186/s12864-024-09975-9)
Supplement: Supplementary file 1 — Additional file 1: Supplementary Table 1. Statistical table of quality control results. [file 12864_2024_9975_MOESM1_ESM.pdf]

Supplementary Table 1. Statistical table of quality control results

Sample_name	Total_raw_read	Total_clean_read	Total_clean_base	Clean_read_q20	Clean_read_q30	Clean_read_ratio
SO-bomi-1	96.03	96.03	14.33	96.91	89.89	100
SO-bomi-2	101	101	15.09	97.8	92.6	100
SO-bomi-3	110.48	110.48	16.51	97.49	91.92	100
SO-bomi-4	130.14	130.14	19.45	97.5	92.06	100
SO-bomi-5	94.81	94.81	14.16	97.83	92.78	100
SO-bomi-6	120.33	120.33	17.98	97.82	92.82	100
SO-bomi-7	98.25	98.25	14.68	97.44	91.74	100
SO-bomi-8	94.04	94.04	14.05	97.64	92.1	100
SO-bomi-9	91.84	91.84	13.72	97.78	92.65	100
SO-dagu-1	99.06	99.06	14.81	97.05	90.18	100
SO-dagu-2	99.04	99.04	14.8	97.21	90.55	100
SO-dagu-3	101.32	101.32	15.14	97.16	90.65	100
SO-dagu-4	107.76	107.76	16.1	97.31	91.05	100
SO-dagu-5	97.49	97.49	14.57	97.01	90.02	100
SO-dagu-6	96.04	96.04	14.35	97.42	91.25	100
SO-dagu-7	104.85	104.84	15.67	97.64	92	99.99
SO-dagu-8	104.5	104.49	15.62	97.67	92.09	99.99
SO-dagu-9	105.57	105.57	15.78	97.53	91.46	100
SO-jiacha-1	106.19	106.19	15.87	97.15	90.61	100
SO-jiacha-2	96.22	96.22	14.38	97.02	90.03	100
SO-jiacha-3	100.44	100.44	15.01	97.37	91.11	100
SO-jiacha-4	93.11	93.11	13.92	97.4	91.17	100
SO-jiacha-5	105.78	105.78	15.82	97.5	91.57	100
SO-jiacha-6	99.74	99.73	14.91	97.53	91.7	99.99
SO-jiacha-7	102.01	102.01	15.24	97.02	90.12	100
SO-jiacha-8	97.11	97.11	14.52	96.97	89.86	100
SO-jiacha-9	95.97	95.97	14.34	97.51	91.46	100
SO-zangmu-1	102.58	102.58	15.33	97.14	90.31	100
SO-zangmu-2	110.44	110.42	16.5	97.4	91.26	99.99
SO-zangmu-3	107.32	107.32	16.04	97.04	90.16	100
SO-zangmu-4	112.1	112.09	16.76	97.67	92.13	99.99
SO-zangmu-5	108.38	108.38	16.2	97.16	90.75	100
SO-zangmu-6	109.53	109.53	16.37	97.07	90.3	100
SO-zangmu-7	107.29	107.29	16.04	97.28	90.78	100
SO-zangmu-8	102.33	102.33	15.3	97.32	91.13	100

SO-zangmu-9	99.61	99.61	14.89	97.41	91.18	100
SO-zangga-1	101.45	101.45	15.16	97.43	91.23	100
SO-zangga-2	107.11	107.11	16.01	97.21	90.5	100
SO-zangga-3	102.7	102.7	15.35	97.45	91.15	100
SO-zangga-4	108.3	108.3	16.19	97.35	91.05	100
SO-zangga-5	112.58	112.58	16.85	97.91	93.17	100
SO-zangga-6	99.54	99.54	14.9	97.89	93.03	100
SO-zangga-7	96.82	96.82	14.47	97.35	91.12	100
SO-zangga-8	95.54	95.54	14.28	97.18	90.6	100
SO-zangga-9	101.5	101.5	15.17	97.1	90.4	100
SO-linzhi-1	76.7	76.7	11.46	97.87	92.76	100
SO-linzhi-2	76.36	76.36	11.41	97.89	92.93	100
SO-linzhi-3	80.73	80.73	12.07	97.3	91.34	100
SO-linzhi-4	79.97	79.97	11.95	98.01	93.43	100
SO-mili-1	112.11	111.99	16.78	97.22	92.11	99.89
SO-mili-2	108.05	107.96	16.18	97.28	92.34	99.92
SO-mili-3	117.09	117.02	17.54	97.5	92.89	99.94
SO-mili-4	93.22	93.14	13.96	97.1	91.9	99.91
