# Supplementary material for: Population structure and adaptability analysis of Schizothorax o’connori based on whole-genome resequencing
Source: BMC Genomics. 2024 Feb 6;25:145. doi: 10.1186/s12864-024-09975-9 (PMC10845765; doi:10.1186/s12864-024-09975-9)
Supplement: Supplementary file 2 — Additional file 2: Supplementary Table 2. GO terms of loci under selection among each comparison population of Schizothorax o’connori. [file 12864_2024_9975_MOESM2_ESM.pdf]

Supplementary Table 2. GO terms of loci under selection among each comparison population of Schizothorax o'connori

Populations	ID	Description	GeneRatio	BgRatio	*p*- value	GeneID
bomi vs zangmu	GO:0007169	tran smembrane receptor protein tyrosine kinase signaling pathway	1/3	11/15848	0.002080968	Soc_3G0009700
bomi vs zangmu	GO:0004714	transmembrane receptor protein tyrosine kinase activity	1/3	12/15848	0.002270004	Soc_3G0009700
bomi vs dagu	GO:0005044	scavenger receptor activity	1/1	66/15848	0.004164563	Soc_2G0002500
dagu vs jiacha	GO:0016887	ATPase activity	1/1	71/15848	0.004480061	Soc_1G0008210
dagu vs milin	GO:0050839	cell adhesion molecule binding	1/10	10/15848	0.00629384	Soc_7G0008770
jiacha vs linzhi	GO:0036459	thiol-dependent ubiquitin-specific protease activity	1/2	52/15848	0.006551783	Soc_20G0008160
dagu vs jiacha	GO:0004842	ubiquitin-protein transferase activity	1/1	105/15848	0.006625442	Soc_1G0008210
jiacha vs milin	GO:0004984	olfactory receptor activity	1/2	53/15848	0.006677567	Soc_16G0006340
dagu vs milin	GO:0046982	protein heterodimerization activity	1/10	12/15848	0.007548321	Soc_7G0008770
jiacha vs linzhi	GO:0016579	protein deubiquitination	1/2	63/15848	0.007934977	Soc_20G0008160
dagu vs milin	GO:0005911	cell-cell junction	1/10	13/15848	0.008175027	Soc_7G0008770
zangmu vs milin	GO:0004984	olfactory receptor activity	1/3	53/15848	0.009999926	Soc_16G0006340
zangag vs milin	GO:0004984	olfactory receptor activity	1/3	53/15848	0.009999926	Soc_16G0006340
dagu vs milin	GO:0042803	protein homodimerization activity	1/10	16/15848	0.010053009	Soc_7G0008770
zangag vs milin	GO:0004842	ubiquitin-protein transferase activity	2/24	105/15848	0.010912788	Soc_1G0003540/Soc_14G0000800
zangga vs linzhi	GO:0006281	DNA repair	1/2	88/15848	0.011075018	Soc_20G0005860
dagu vs zangga	GO:0006464	cellular protein modification process	1/6	30/15848	0.01130606	Soc_14G0000320
dagu vs zangga	GO:0004571	mannosyl-oligosaccharide 1,2-alpha-mannosidase activity	1/21	10/15848	0.013175868	Soc_U0000420
dagu vs zangga	GO:0070403	NAD+ binding	1/21	10/15848	0.013175868	Soc_16G0006050
dagu vs jiacha	GO:0046872	metal ion binding	1/1	210/15848	0.013250883	Soc_1G0008210
zangmu vs linzhi	GO:0004984	olfactory receptor activity	1/4	53/15848	0.013311381	Soc_16G0006340
jiacha vs zangmu	GO:0016887	ATPase activity	1/3	71/15848	0.013380899	Soc_1G0008210
bomi vs mili	GO:0004190	aspartic-type endopeptidase activity	1/12	18/15848	0.013549334	Soc_24G0000080
bomi vs mili	GO:0006352	DNA-templated transcription, initiation	1/12	20/15848	0.015044381	Soc_20G0000330
milin vs linzhi	GO:0006352	DNA-templated transcription, initiation	1/12	20/15848	0.015044381	Soc_20G0000330
dagu vs zangga	GO:0000724	double-strand break repair via homologous recombination	1/21	12/15848	0.015791125	Soc_14G0007230
zangmu vs milin	GO:0006352	DNA-templated transcription, initiation	1/13	20/15848	0.016288326	Soc_20G0000330
jiacha vs zangga	GO:0008233	peptidase activity	1/9	29/15848	0.016353021	Soc_14G0000500
zangmu vs zangga	GO:0008233	peptidase activity	1/9	29/15848	0.016353021	Soc_14G0000500
bomi vs linzhi	GO:0006281	DNA repair	1/3	88/15848	0.016566965	Soc_20G0005860
dagu vs linzhi	GO:0006281	DNA repair	1/3	88/15848	0.016566965	Soc_20G0005860

jiacha vs zangga	GO:0006464	cellular protein modification process	1/9	30/15848	0.016912653	Soc_14G0000320
zangmu vs zangga	GO:0006464	cellular protein modification process	1/9	30/15848	0.016912653	Soc_14G0000320
bomi vs zangmu	GO:0004713	protein tyrosine kinase activity	1/3	91/15848	0.017128499	Soc_3G0009700
bomi vs zangga	GO:0004842	ubiquitin-protein transferase activity	1/3	105/15848	0.019746164	Soc_14G0000800
jiacha vs zangmu	GO:0004842	ubiquitin-protein transferase activity	1/3	105/15848	0.019746164	Soc_1G0008210
bomi vs zangga	GO:0004571	mannosyl-oligosaccharide 1,2-alpha-mannosidase activity	1/41	10/15848	0.025578837	Soc_U0000420
bomi vs zangga	GO:0008134	transcription factor binding	1/41	10/15848	0.025578837	Soc_16G0006880
bomi vs zangga	GO:0045944	positive regulation of transcription by RNA polymerase II	1/41	10/15848	0.025578837	Soc_16G0006720
bomi vs zangga	GO:0070403	NAD+ binding	1/41	10/15848	0.025578837	Soc_16G0006050
jiacha vs zangga	GO:0004867	serine-type endopeptidase inhibitor activity	1/9	46/15848	0.025828362	Soc_14G0000500
zangmu vs zangga	GO:0004867	serine-type endopeptidase inhibitor activity	1/9	46/15848	0.025828362	Soc_14G0000500
jiacha vs milin	GO:0004984	olfactory receptor activity	1/8	53/15848	0.026448868	Soc_16G0006340
jiacha vs zangga	GO:0003700	DNA-binding transcription factor activity	2/9	476/15848	0.028178035	Soc_14G0000300/Soc_14G0000540
zangmu vs zangga	GO:0003700	DNA-binding transcription factor activity	2/9	476/15848	0.028178035	Soc_14G0000300/Soc_14G0000540
zangga vs linzhi	GO:0004984	olfactory receptor activity	1/9	53/15848	0.029706331	Soc_16G0006340
bomi vs zangga	GO:0004842	ubiquitin-protein transferase activity	2/41	105/15848	0.030148443	Soc_1G0003540/Soc_14G0000800
bomi vs zangga	GO:0005694	chromosome	1/41	12/15848	0.030617455	Soc_16G0007620
bomi vs zangga	GO:0004222	metalloendopeptidase activity	2/41	109/15848	0.032291534	Soc_14G0000500/Soc_16G0006860
dagu vs milin	GO:0004984	olfactory receptor activity	1/10	53/15848	0.032953099	Soc_16G0006340
jiacha vs zangga	GO:0003707	steroid hormone receptor activity	1/9	61/15848	0.034121487	Soc_14G0000300
jiacha vs zangga	GO:0043401	steroid hormone mediated signaling pathway	1/9	61/15848	0.034121487	Soc_14G0000300
zangmu vs zangga	GO:0003707	steroid hormone receptor activity	1/9	61/15848	0.034121487	Soc_14G0000300
zangmu vs zangga	GO:0043401	steroid hormone mediated signaling pathway	1/9	61/15848	0.034121487	Soc_14G0000300
bomi vs mili	GO:0042626	ATPase-coupled transmembrane transporter activity	1/12	46/15848	0.034291906	Soc_17G0007270
jiacha vs milin	GO:0016887	ATPase activity	1/8	71/15848	0.035291178	Soc_1G0008210
milin vs linzhi	GO:0005622	intracellular	2/12	400/15848	0.035482856	Soc_1G0020680/Soc_1G0021560
bomi vs zangga	GO:0003712	transcription coregulator activity	1/41	14/15848	0.035630651	Soc_16G0006880
zangmu vs milin	GO:0042626	ATPase-coupled transmembrane transporter activity	1/13	46/15848	0.037097069	Soc_17G0007270
dagu vs zangga	GO:0008233	peptidase activity	1/21	29/15848	0.03775586	Soc_14G0000500
jiacha vs zangga	GO:0008233	peptidase activity	1/21	29/15848	0.03775586	Soc_14G0000500

zangmu vs zangga	GO:0008233	peptidase activity	1/21	29/15848	0.03775586	Soc_14G0000500
bomi vs zangga	GO:0005813	centrosome	1/41	15/15848	0.038127754	Soc_16G0007630
dagu vs zangga	GO:0006464	cellular protein modification process	1/21	30/15848	0.039033256	Soc_14G0000320
jiacha vs zangga	GO:0005578	extracellular matrix	1/21	30/15848	0.039033256	Soc_4G0011300
jiacha vs zangga	GO:0006464	cellular protein modification process	1/21	30/15848	0.039033256	Soc_14G0000320
zangmu vs zangga	GO:0006464	cellular protein modification process	1/21	30/15848	0.039033256	Soc_14G0000320
dagu vs zangga	GO:0004842	ubiquitin-protein transferase activity	1/6	105/15848	0.039106058	Soc_14G0000800
jiacha vs zangmu	GO:0046872	metal ion binding	1/3	210/15848	0.039230662	Soc_1G0008210
bomi vs mili	GO:0004984	olfactory receptor activity	1/12	53/15848	0.03941469	Soc_16G0006340
milin vs linzhi	GO:0004984	olfactory receptor activity	1/12	53/15848	0.03941469	Soc_16G0006340
dagu vs zangga	GO:0071805	potassium ion transmembrane transport	1/21	31/15848	0.040309037	Soc_14G0000040
jiacha vs zangga	GO:0071805	potassium ion transmembrane transport	1/21	31/15848	0.040309037	Soc_14G0000040
zangmu vs zangga	GO:0071805	potassium ion transmembrane transport	1/21	31/15848	0.040309037	Soc_14G0000040
bomi vs zangga	GO:0008324	cation transmembrane transporter activity	1/41	16/15848	0.04061855	Soc_17G0007800
dagu vs zangga	GO:0005267	potassium channel activity	1/21	32/15848	0.041583205	Soc_14G0000040
jiacha vs zangga	GO:0005267	potassium channel activity	1/21	32/15848	0.041583205	Soc_14G0000040
zangmu vs zangga	GO:0005267	potassium channel activity	1/21	32/15848	0.041583205	Soc_14G0000040
zangmu vs milin	GO:0004984	olfactory receptor activity	1/13	53/15848	0.042629582	Soc_16G0006340
zangag vs milin	GO:0008233	peptidase activity	1/24	29/15848	0.043035901	Soc_14G0000500
milin vs linzhi	GO:0005085	guanyl-nucleotide exchange factor activity	1/12	58/15848	0.043058558	Soc_2G0017770
zangag vs milin	GO:0006464	cellular protein modification process	1/24	30/15848	0.044487772	Soc_14G0000320
bomi vs zangga	GO:0007219	Notch signaling pathway	1/41	18/15848	0.045581277	Soc_16G0006720
zangag vs milin	GO:0071805	potassium ion transmembrane transport	1/24	31/15848	0.045937531	Soc_14G0000040
zangag vs milin	GO:0005267	potassium channel activity	1/24	32/15848	0.047385182	Soc_14G0000040
zangga vs linzhi	GO:0006281	DNA repair	1/9	88/15848	0.048891101	Soc_20G0005860
jiacha vs milin	GO:0004842	ubiquitin-protein transferase activity	1/8	105/15848	0.051801762	Soc_1G0008210
bomi vs mili	GO:0016887	ATPase activity	1/12	71/15848	0.052473396	Soc_17G0007270
dagu vs milin	GO:0007166	cell surface receptor signaling pathway	1/10	86/15848	0.052974059	Soc_4G0002720
zangmu vs milin	GO:0016887	ATPase activity	1/13	71/15848	0.056721589	Soc_17G0007270
jiacha vs zangga	GO:0004842	ubiquitin-protein transferase activity	1/9	105/15848	0.058087167	Soc_14G0000800
zangmu vs zangga	GO:0004842	ubiquitin-protein transferase activity	1/9	105/15848	0.058087167	Soc_14G0000800
zangga vs linzhi	GO:0004842	ubiquitin-protein transferase activity	1/9	105/15848	0.058087167	Soc_14G0000800
dagu vs zangga	GO:0004867	serine-type endopeptidase inhibitor activity	1/21	46/15848	0.05925325	Soc_14G0000500
jiacha vs zangga	GO:0004867	serine-type endopeptidase inhibitor activity	1/21	46/15848	0.05925325	Soc_14G0000500
zangmu vs zangga	GO:0004867	serine-type endopeptidase inhibitor activity	1/21	46/15848	0.05925325	Soc_14G0000500
jiacha vs zangga	GO:0004222	metalloendopeptidase activity	1/9	109/15848	0.060239426	Soc_14G0000500
zangmu vs zangga	GO:0004222	metalloendopeptidase activity	1/9	109/15848	0.060239426	Soc_14G0000500
bomi vs zangga	GO:0003713	transcription coactivator activity	1/41	25/15848	0.062754372	Soc_16G0006720

bomi vs mili	GO:0007166	cell surface receptor signaling pathway	1/12	86/15848	0.06323112	Soc_4G0002720
zangag vs milin	GO:0004867	serine-type endopeptidase inhibitor activity	1/24	46/15848	0.06743258	Soc_14G0000500
bomi vs zangga	GO:0008233	peptidase activity	1/41	29/15848	0.072431826	Soc_14G0000500
bomi vs zangga	GO:0005578	extracellular matrix	1/41	30/15848	0.074835916	Soc_4G0011300
bomi vs zangga	GO:0006464	cellular protein modification process	1/41	30/15848	0.074835916	Soc_14G0000320
bomi vs zangga	GO:0071805	potassium ion transmembrane transport	1/41	31/15848	0.077233926	Soc_14G0000040
zangag vs milin	GO:0004984	olfactory receptor activity	1/24	53/15848	0.077304036	Soc_16G0006340
dagu vs zangga	GO:0003707	steroid hormone receptor activity	1/21	61/15848	0.077840977	Soc_14G0000300
dagu vs zangga	GO:0043401	steroid hormone mediated signaling pathway	1/21	61/15848	0.077840977	Soc_14G0000300
jiacha vs zangga	GO:0003707	steroid hormone receptor activity	1/21	61/15848	0.077840977	Soc_14G0000300
jiacha vs zangga	GO:0043401	steroid hormone mediated signaling pathway	1/21	61/15848	0.077840977	Soc_14G0000300
zangmu vs zangga	GO:0003707	steroid hormone receptor activity	1/21	61/15848	0.077840977	Soc_14G0000300
zangmu vs zangga	GO:0043401	steroid hormone mediated signaling pathway	1/21	61/15848	0.077840977	Soc_14G0000300
bomi vs zangga	GO:0005267	potassium channel activity	1/41	32/15848	0.079625872	Soc_14G0000040
zangmu vs milin	GO:0004842	ubiquitin-protein transferase activity	1/13	105/15848	0.082818758	Soc_1G0003540
bomi vs zangga	GO:0005667	transcription regulator complex	1/41	34/15848	0.08439163	Soc_16G0006880
bomi vs zangga	GO:0003700	DNA-binding transcription factor activity	1/3	476/15848	0.087432088	Soc_14G0000540
zangag vs milin	GO:0003700	DNA-binding transcription factor activity	1/3	476/15848	0.087432088	Soc_14G0000540
zangag vs milin	GO:0003707	steroid hormone receptor activity	1/24	61/15848	0.088463086	Soc_14G0000300
zangag vs milin	GO:0043401	steroid hormone mediated signaling pathway	1/24	61/15848	0.088463086	Soc_14G0000300
dagu vs milin	GO:0007155	cell adhesion	1/10	149/15848	0.09016307	Soc_7G0008770
zangmu vs linzhi	GO:0005622	intracellular	1/4	400/15848	0.097209595	Soc_1G0020680
jiacha vs zangga	GO:0042981	regulation of apoptotic process	1/21	77/15848	0.097282146	Soc_1G0023160
bomi vs zangga	GO:0006812	cation transport	1/41	40/15848	0.098544908	Soc_17G0007800
bomi vs zangga	GO:0007049	cell cycle	1/41	40/15848	0.098544908	Soc_16G0006880
bomi vs mili	GO:0000786	nucleosome	1/12	137/15848	0.098975471	Soc_20G0000330
milin vs linzhi	GO:0000786	nucleosome	1/12	137/15848	0.098975471	Soc_20G0000330
dagu vs milin	GO:0004888	transmembrane signaling receptor activity	1/10	165/15848	0.099396107	Soc_4G0002720
milin vs linzhi	GO:0007264	small GTPase mediated signal transduction	1/12	139/15848	0.100351387	Soc_2G0017770
jiacha vs milin	GO:0046872	metal ion binding	1/8	210/15848	0.101240151	Soc_1G0008210
bomi vs mili	GO:0005887	integral component of plasma membrane	1/12	141/15848	0.101725377	Soc_17G0007270
jiacha vs zangga	GO:0043565	sequence-specific DNA binding	1/9	191/15848	0.103408201	Soc_14G0000300
zangmu vs zangga	GO:0043565	sequence-specific DNA binding	1/9	191/15848	0.103408201	Soc_14G0000300
zangmu vs milin	GO:0000786	nucleosome	1/13	137/15848	0.106770392	Soc_20G0000330
zangmu vs milin	GO:0005887	integral component of plasma membrane	1/13	141/15848	0.109723402	Soc_17G0007270
dagu vs zangga	GO:0006281	DNA repair	1/21	88/15848	0.110420836	Soc_14G0007230
jiacha vs zangga	GO:0006281	DNA repair	1/21	88/15848	0.110420836	Soc_20G0005860
zangmu vs zangga	GO:0006281	DNA repair	1/21	88/15848	0.110420836	Soc_20G0005860

bomi vs zangga	GO:0004867	serine-type endopeptidase inhibitor activity	1/41	46/15848	0.112484664	Soc_14G0000500
dagu vs zangga	GO:0004713	protein tyrosine kinase activity	1/21	91/15848	0.113972384	Soc_16G0007750
bomi vs mili	GO:0004888	transmembrane signaling receptor activity	1/12	165/15848	0.118063897	Soc_4G0002720
bomi vs zangga	GO:0003700	DNA-binding transcription factor activity	3/41	476/15848	0.124538828	Soc_14G0000300/Soc_14G0000540/Soc_16G0006880
zangag vs milin	GO:0006281	DNA repair	1/24	88/15848	0.12517791	Soc_20G0005860
dagu vs zangga	GO:0003700	DNA-binding transcription factor activity	2/21	476/15848	0.130089526	Soc_14G0000300/Soc_14G0000540
jiacha vs zangga	GO:0003700	DNA-binding transcription factor activity	2/21	476/15848	0.130089526	Soc_14G0000300/Soc_14G0000540
zangmu vs zangga	GO:0003700	DNA-binding transcription factor activity	2/21	476/15848	0.130089526	Soc_14G0000300/Soc_14G0000540
dagu vs zangga	GO:0004842	ubiquitin-protein transferase activity	1/21	105/15848	0.130368473	Soc_14G0000800
jiacha vs zangga	GO:0004842	ubiquitin-protein transferase activity	1/21	105/15848	0.130368473	Soc_14G0000800
zangmu vs zangga	GO:0004842	ubiquitin-protein transferase activity	1/21	105/15848	0.130368473	Soc_14G0000800
dagu vs zangga	GO:0004222	metalloendopeptidase activity	1/21	109/15848	0.134999735	Soc_14G0000500
jiacha vs zangga	GO:0004222	metalloendopeptidase activity	1/21	109/15848	0.134999735	Soc_14G0000500
zangmu vs zangga	GO:0004222	metalloendopeptidase activity	1/21	109/15848	0.134999735	Soc_14G0000500
bomi vs zangga	GO:0005085	guanyl-nucleotide exchange factor activity	1/41	58/15848	0.139736128	Soc_16G0007130
bomi vs zangga	GO:0003707	steroid hormone receptor activity	1/41	61/15848	0.146420397	Soc_14G0000300
bomi vs zangga	GO:0043401	steroid hormone mediated signaling pathway	1/41	61/15848	0.146420397	Soc_14G0000300
zangag vs milin	GO:0004222	metalloendopeptidase activity	1/24	109/15848	0.15274973	Soc_14G0000500
zangag vs milin	GO:0003700	DNA-binding transcription factor activity	2/24	476/15848	0.161458753	Soc_14G0000300/Soc_14G0000540
dagu vs zangga	GO:0003700	DNA-binding transcription factor activity	1/6	476/15848	0.167234455	Soc_14G0000540
bomi vs zangga	GO:0016705	oxidoreductase activity, acting on paired donors, with incorporation or reduction of molecular oxygen	1/41	74/15848	0.174804722	Soc_16G0007770
bomi vs zangga	GO:0004871	obsolete signal transducer activity	1/41	78/15848	0.183351576	Soc_16G0007220
jiacha vs milin	GO:0005622	intracellular	1/8	400/15848	0.184990799	Soc_1G0021560
bomi vs zangga	GO:0006281	DNA repair	1/41	88/15848	0.204342634	Soc_20G0005860
bomi vs zangga	GO:0004713	protein tyrosine kinase activity	1/41	91/15848	0.210536647	Soc_16G0007750
bomi vs zangga	GO:0020037	heme binding	1/41	91/15848	0.210536647	Soc_16G0007770
bomi vs zangga	GO:0015074	DNA integration	1/41	94/15848	0.216683609	Soc_16G0006700
dagu vs zangga	GO:0043565	sequence-specific DNA binding	1/21	191/15848	0.22492165	Soc_14G0000300
jiacha vs zangga	GO:0043565	sequence-specific DNA binding	1/21	191/15848	0.22492165	Soc_14G0000300
zangmu vs zangga	GO:0043565	sequence-specific DNA binding	1/21	191/15848	0.22492165	Soc_14G0000300
dagu vs milin	GO:0005622	intracellular	1/10	400/15848	0.225634424	Soc_1G0021560

zangga vs linzhi	GO:0003700	DNA-binding transcription factor activity	1/9	476/15848	0.240071627	Soc_14G0000540
dagu vs zangga	GO:0046872	metal ion binding	1/21	210/15848	0.244448061	Soc_4G0000930
zangag vs milin	GO:0043565	sequence-specific DNA binding	1/24	191/15848	0.252647006	Soc_14G0000300
bomi vs zangga	GO:0005506	iron ion binding	1/41	114/15848	0.256486389	Soc_16G0007770
bomi vs mili	GO:0005622	intracellular	1/12	400/15848	0.264256009	Soc_1G0021560
bomi vs mili	GO:0006508	proteolysis	1/12	422/15848	0.276735962	Soc_24G0000080
zangmu vs milin	GO:0005622	intracellular	1/13	400/15848	0.282840095	Soc_1G0021560
bomi vs zangga	GO:0006508	proteolysis	2/41	422/15848	0.298415034	Soc_16G0006860/Soc_16G0007340
bomi vs zangga	GO:0007264	small GTPase mediated signal transduction	1/41	139/15848	0.303472947	Soc_16G0007130
bomi vs zangga	GO:0004252	serine-type endopeptidase activity	1/41	143/15848	0.310716872	Soc_16G0007340
bomi vs zangga	GO:0035556	intracellular signal transduction	1/41	170/15848	0.35772647	Soc_16G0007220
bomi vs zangga	GO:0003824	catalytic activity	1/41	178/15848	0.371044096	Soc_17G0007820
bomi vs zangga	GO:0043565	sequence-specific DNA binding	1/41	191/15848	0.392112721	Soc_14G0000300
zangag vs milin	GO:0005622	intracellular	1/24	400/15848	0.45880831	Soc_1G0021560
bomi vs zangga	GO:0046983	protein dimerization activity	1/41	236/15848	0.459859163	Soc_1G0000770
bomi vs zangga	GO:0007165	signal transduction	1/41	351/15848	0.60125968	Soc_16G0007220
bomi vs zangga	GO:0055114	oxidation-reduction process	1/41	413/15848	0.661766425	Soc_16G0007770
