# Supplementary material for: Population structure and adaptability analysis of Schizothorax o’connori based on whole-genome resequencing
Source: BMC Genomics. 2024 Feb 6;25:145. doi: 10.1186/s12864-024-09975-9 (PMC10845765; doi:10.1186/s12864-024-09975-9)
Supplement: Supplementary file 3 — Additional file 3: Supplementary Table 3. KEGG pathways of loci under selection among each comparison population of Schizothorax. [file 12864_2024_9975_MOESM3_ESM.pdf]

Supplementary Table 3. KEGG pathways of loci under selection among each comparison population of Schizothora›

| Population | ID | Description | GeneRatio | BgRatio | pvalue |
|---|---|---|---|---|---|
| dagu vs milin | ko04740 | Olfactory transduction | 2/2 | 93/8136 | 0.000129271 |
| zangga vs linzhi | ko05146 | Amoebiasis | 2/4 | 127/8136 | 0.001421078 |
| zangga vs linzhi | ko05226 | Gastric cancer | 2/4 | 206/8136 | 0.003701462 |
| bomi vs zangga | ko00260 | Glycine, serine and threonine metabolism | 1/1 | 42/8136 | 0.005162242 |
| dagu vs zangga | ko00260 | Glycine, serine and threonine metabolism | 1/1 | 42/8136 | 0.005162242 |
| zangmu vs zangga | ko05146 | Amoebiasis | 2/9 | 127/8136 | 0.008100578 |
| bomi vs zangmu | ko04913 | Ovarian steroidogenesis | 1/1 | 85/8136 | 0.010447394 |
| bomi vs zangmu | ko04213 | Longevity regulating pathway - multiple species | 1/1 | 91/8136 | 0.011184857 |
| bomi vs linzhi | ko03460 | Fanconi anemia pathway | 1/2 | 46/8136 | 0.011276493 |
| dagu vs linzhi | ko03460 | Fanconi anemia pathway | 1/2 | 46/8136 | 0.011276493 |
| zangga vs linzhi | ko03460 | Fanconi anemia pathway | 1/2 | 46/8136 | 0.011276493 |
| jiacha vs milin | ko04740 | Olfactory transduction | 1/1 | 93/8136 | 0.011430678 |
| jiacha vs milin | ko04740 | Olfactory transduction | 1/1 | 93/8136 | 0.011430678 |
| zangmu vs milin | ko04740 | Olfactory transduction | 1/1 | 93/8136 | 0.011430678 |
| zangmu vs linzhi | ko04740 | Olfactory transduction | 1/1 | 93/8136 | 0.011430678 |
| zangga vs milin | ko04740 | Olfactory transduction | 1/1 | 93/8136 | 0.011430678 |
| bomi vs zangmu | ko05214 | Glioma | 1/1 | 100/8136 | 0.012291052 |
| bomi vs zangmu | ko04730 | Long-term depression | 1/1 | 114/8136 | 0.014011799 |
| bomi vs zangmu | ko05218 | Melanoma | 1/1 | 114/8136 | 0.014011799 |
| bomi vs zangmu | ko04914 | Progesterone-mediated oocyte maturation | 1/1 | 116/8136 | 0.01425762 |
| bomi vs zangmu | ko04211 | Longevity regulating pathway | 1/1 | 122/8136 | 0.014995084 |
| bomi vs zangmu | ko01521 | EGFR tyrosine kinase inhibitor resistance | 1/1 | 124/8136 | 0.015240905 |
| bomi vs zangmu | ko05215 | Prostate cancer | 1/1 | 126/8136 | 0.015486726 |
| bomi vs zangmu | ko01522 | Endocrine resistance | 1/1 | 130/8136 | 0.015978368 |
| bomi vs zangmu | ko04114 | Oocyte meiosis | 1/1 | 134/8136 | 0.01647001 |
| bomi vs zangmu | ko04520 | Adherens junction | 1/1 | 135/8136 | 0.01659292 |
| zangmu vs milin | ko01523 | Antifolate resistance | 1/3 | 46/8136 | 0.016867995 |
| bomi vs zangmu | ko04066 | HIF-1 signaling pathway | 1/1 | 145/8136 | 0.017822026 |
| bomi vs zangmu | ko04152 | AMPK signaling pathway | 1/1 | 152/8136 | 0.018682399 |
| zangmu vs milin | ko02010 | ABC transporters | 1/3 | 54/8136 | 0.019782056 |
| zangmu vs zangga | ko05226 | Gastric cancer | 2/9 | 206/8136 | 0.020427405 |
| bomi vs zangmu | ko04068 | FoxO signaling pathway | 1/1 | 168/8136 | 0.020648968 |
| dagu vs milin | ko04923 | Regulation of lipolysis in adipocytes | 1/2 | 90/8136 | 0.022002872 |
| bomi vs mili | ko01523 | Antifolate resistance | 1/4 | 46/8136 | 0.02242856 |
| zangga vs linzhi | ko03460 | Fanconi anemia pathway | 1/4 | 46/8136 | 0.02242856 |
| bomi vs zangmu | ko04140 | Autophagy - animal | 1/1 | 189/8136 | 0.023230088 |

| | | | | | |
|---|---|---|---|---|---|
| bomi vs zangmu | ko04550 | Signaling pathways regulating pluripotency of stem cells | 1/1 | 195/8136 | 0.023967552 |
| jiacha vs zangga | ko04550 | Signaling pathways regulating pluripotency of stem cells | 1/1 | 195/8136 | 0.023967552 |
| zangmu vs zangga | ko04550 | Signaling pathways regulating pluripotency of stem cells | 1/1 | 195/8136 | 0.023967552 |
| bomi vs zangmu | ko04150 | mTOR signaling pathway | 1/1 | 202/8136 | 0.024827925 |
| bomi vs zangmu | ko05224 | Breast cancer | 1/1 | 209/8136 | 0.025688299 |
| bomi vs mili | ko02010 | ABC transporters | 1/4 | 54/8136 | 0.026290327 |
| bomi vs zangmu | ko05225 | Hepatocellular carcinoma | 1/1 | 214/8136 | 0.026302852 |
| dagu vs milin | ko04730 | Long-term depression | 1/2 | 114/8136 | 0.027828967 |
| dagu vs milin | ko04970 | Salivary secretion | 1/2 | 114/8136 | 0.027828967 |
| zangmu vs milin | ko01524 | Platinum drug resistance | 1/3 | 81/8136 | 0.029574491 |
| bomi vs zangmu | ko05202 | Transcriptional misregulation in cancer | 1/1 | 247/8136 | 0.030358899 |
| bomi vs linzhi | ko05146 | Amoebiasis | 1/2 | 127/8136 | 0.030977501 |
| dagu vs linzhi | ko05146 | Amoebiasis | 1/2 | 127/8136 | 0.030977501 |
| zangga vs linzhi | ko05146 | Amoebiasis | 1/2 | 127/8136 | 0.030977501 |
| milin vs linzhi | ko04740 | Olfactory transduction | 1/3 | 93/8136 | 0.033905668 |
| zangmu vs milin | ko04740 | Olfactory transduction | 1/3 | 93/8136 | 0.033905668 |
| bomi vs zangmu | ko04510 | Focal adhesion | 1/1 | 276/8136 | 0.033923304 |
| dagu vs milin | ko04540 | Gap junction | 1/2 | 145/8136 | 0.035328578 |
| dagu vs zangga | ko00260 | Glycine, serine and threonine metabolism | 1/7 | 42/8136 | 0.035593783 |
| jiacha vs zangga | ko00260 | Glycine, serine and threonine metabolism | 1/7 | 42/8136 | 0.035593783 |
| zangga vs milin | ko00260 | Glycine, serine and threonine metabolism | 1/7 | 42/8136 | 0.035593783 |
| bomi vs zangmu | ko05205 | Proteoglycans in cancer | 1/1 | 291/8136 | 0.035766962 |
| dagu vs zangga | ko04215 | Apoptosis - multiple species | 1/7 | 44/8136 | 0.037261276 |
| jiacha vs zangga | ko04215 | Apoptosis - multiple species | 1/7 | 44/8136 | 0.037261276 |
| bomi vs zangmu | ko04014 | Ras signaling pathway | 1/1 | 309/8136 | 0.037979351 |
| jiacha vs zangga | ko03460 | Fanconi anemia pathway | 1/7 | 46/8136 | 0.038926296 |
| zangga vs milin | ko03460 | Fanconi anemia pathway | 1/7 | 46/8136 | 0.038926296 |
| bomi vs zangmu | ko04015 | Rap1 signaling pathway | 1/1 | 317/8136 | 0.038962635 |
| bomi vs mili | ko01524 | Platinum drug resistance | 1/4 | 81/8136 | 0.039239371 |
| bomi vs zangmu | ko04144 | Endocytosis | 1/1 | 329/8136 | 0.040437561 |
| dagu vs zangga | ko00513 | Various types of N-glycan biosynthesis | 1/7 | 48/8136 | 0.040588848 |
| dagu vs milin | ko04611 | Platelet activation | 1/2 | 180/8136 | 0.04376098 |
| dagu vs milin | ko04713 | Circadian entrainment | 1/2 | 182/8136 | 0.044241714 |
| bomi vs mili | ko04740 | Olfactory transduction | 1/4 | 93/8136 | 0.044952855 |
| zangga vs linzhi | ko04740 | Olfactory transduction | 1/4 | 93/8136 | 0.044952855 |
| zangmu vs zangga | ko00260 | Glycine, serine and threonine metabolism | 1/9 | 42/8136 | 0.045534219 |
| zangmu vs zangga | ko04215 | Apoptosis - multiple species | 1/9 | 44/8136 | 0.047655778 |
| zangmu vs milin | ko04976 | Bile secretion | 1/3 | 133/8136 | 0.048249817 |

| | | | | | |
|---|---|---|---|---|---|
| dagu vs zangga | ko00510 | N-Glycan biosynthesis | 1/7 | 58/8136 | 0.048864679 |
| dagu vs milin | ko04270 | Vascular smooth muscle contraction | 1/2 | 203/8136 | 0.049282118 |
| zangmu vs zangga | ko03460 | Fanconi anemia pathway | 1/9 | 46/8136 | 0.049773143 |
| bomi vs linzhi | ko05226 | Gastric cancer | 1/2 | 206/8136 | 0.050001088 |
| dagu vs linzhi | ko05226 | Gastric cancer | 1/2 | 206/8136 | 0.050001088 |
| zangga vs linzhi | ko05226 | Gastric cancer | 1/2 | 206/8136 | 0.050001088 |
| bomi vs zangmu | ko04010 | MAPK signaling pathway | 1/1 | 431/8136 | 0.052974435 |
| bomi vs zangmu | ko04151 | PI3K-Akt signaling pathway | 1/1 | 437/8136 | 0.053711898 |
| dagu vs milin | ko04714 | Thermogenesis | 1/2 | 246/8136 | 0.059561366 |
| dagu vs zangga | ko04742 | Taste transduction | 1/7 | 72/8136 | 0.060348005 |
| jiacha vs zangga | ko04742 | Taste transduction | 1/7 | 72/8136 | 0.060348005 |
| zangga vs milin | ko04742 | Taste transduction | 1/7 | 72/8136 | 0.060348005 |
| dagu vs milin | ko04022 | cGMP-PKG signaling pathway | 1/2 | 252/8136 | 0.060991237 |
| milin vs linzhi | ko05322 | Systemic lupus erythematosus | 1/3 | 174/8136 | 0.062804489 |
| zangmu vs milin | ko05322 | Systemic lupus erythematosus | 1/3 | 174/8136 | 0.062804489 |
| bomi vs mili | ko04976 | Bile secretion | 1/4 | 133/8136 | 0.063813911 |
| bomi vs zangga | ko00260 | Glycine, serine and threonine metabolism | 1/13 | 42/8136 | 0.065115941 |
| bomi vs zangga | ko04215 | Apoptosis - multiple species | 1/13 | 44/8136 | 0.068116801 |
| bomi vs zangga | ko03460 | Fanconi anemia pathway | 1/13 | 46/8136 | 0.071108768 |
| milin vs linzhi | ko04613 | NA | 1/3 | 202/8136 | 0.07265851 |
| zangmu vs milin | ko04613 | NA | 1/3 | 202/8136 | 0.07265851 |
| milin vs linzhi | ko05034 | Alcoholism | 1/3 | 205/8136 | 0.073710183 |
| zangmu vs milin | ko05034 | Alcoholism | 1/3 | 205/8136 | 0.073710183 |
| bomi vs zangga | ko00513 | Various types of N-glycan biosynthesis | 1/13 | 48/8136 | 0.074091866 |
| zangmu vs zangga | ko04742 | Taste transduction | 1/9 | 72/8136 | 0.076920634 |
| zangga vs milin | ko04740 | Olfactory transduction | 1/7 | 93/8136 | 0.077350113 |
| bomi vs mili | ko05322 | Systemic lupus erythematosus | 1/4 | 174/8136 | 0.082855149 |
| milin vs linzhi | ko05202 | Transcriptional misregulation in cancer | 1/3 | 247/8136 | 0.088350216 |
| zangmu vs milin | ko05202 | Transcriptional misregulation in cancer | 1/3 | 247/8136 | 0.088350216 |
| bomi vs zangga | ko00510 | N-Glycan biosynthesis | 1/13 | 58/8136 | 0.088875152 |
| dagu vs zangga | ko04974 | Protein digestion and absorption | 1/7 | 112/8136 | 0.092505074 |
| jiacha vs zangga | ko04974 | Protein digestion and absorption | 1/7 | 112/8136 | 0.092505074 |
| zangga vs milin | ko04974 | Protein digestion and absorption | 1/7 | 112/8136 | 0.092505074 |
| bomi vs mili | ko04613 | NA | 1/4 | 202/8136 | 0.095690968 |
| bomi vs mili | ko05034 | Alcoholism | 1/4 | 205/8136 | 0.097058199 |
| dagu vs zangga | ko01521 | EGFR tyrosine kinase inhibitor resistance | 1/7 | 124/8136 | 0.101966275 |
| jiacha vs zangga | ko05146 | Amoebiasis | 1/7 | 127/8136 | 0.10431832 |
| zangga vs milin | ko05146 | Amoebiasis | 1/7 | 127/8136 | 0.10431832 |

| | | | | | |
|---|---|---|---|---|---|
| bomi vs zangga | ko04330 | Notch signaling pathway | 1/13 | 70/8136 | 0.106327405 |
| bomi vs zangga | ko04742 | Taste transduction | 1/13 | 72/8136 | 0.109205932 |
| milin vs linzhi | ko04014 | Ras signaling pathway | 1/3 | 309/8136 | 0.109678506 |
| milin vs linzhi | ko04015 | Rap1 signaling pathway | 1/3 | 317/8136 | 0.112406065 |
| bomi vs mili | ko05202 | Transcriptional misregulation in cancer | 1/4 | 247/8136 | 0.116037108 |
| zangmu vs zangga | ko04974 | Protein digestion and absorption | 1/9 | 112/8136 | 0.117340948 |
| dagu vs zangga | ko04550 | Signaling pathways regulating pluripotency of stem cells | 1/7 | 195/8136 | 0.156233553 |
| jiacha vs zangga | ko04550 | Signaling pathways regulating pluripotency of stem cells | 1/7 | 195/8136 | 0.156233553 |
| zangga vs milin | ko04550 | Signaling pathways regulating pluripotency of stem cells | 1/7 | 195/8136 | 0.156233553 |
| dagu vs zangga | ko04141 | Protein processing in endoplasmic reticulum | 1/7 | 199/8136 | 0.159205306 |
| dagu vs zangga | ko04210 | Apoptosis | 1/7 | 205/8136 | 0.163646113 |
| jiacha vs zangga | ko04210 | Apoptosis | 1/7 | 205/8136 | 0.163646113 |
| jiacha vs zangga | ko05226 | Gastric cancer | 1/7 | 206/8136 | 0.16438429 |
| zangga vs milin | ko05226 | Gastric cancer | 1/7 | 206/8136 | 0.16438429 |
| bomi vs zangga | ko04974 | Protein digestion and absorption | 1/13 | 112/8136 | 0.16500944 |
| bomi vs zangga | ko01521 | EGFR tyrosine kinase inhibitor resistance | 1/13 | 124/8136 | 0.181110121 |
| bomi vs zangga | ko04658 | Th1 and Th2 cell differentiation | 1/13 | 125/8136 | 0.182438824 |
| bomi vs zangga | ko05146 | Amoebiasis | 1/13 | 127/8136 | 0.185090262 |
| bomi vs zangga | ko04110 | Cell cycle | 1/13 | 135/8136 | 0.195616836 |
| zangmu vs zangga | ko04550 | Signaling pathways regulating pluripotency of stem cells | 1/9 | 195/8136 | 0.196231313 |
| zangmu vs zangga | ko04210 | Apoptosis | 1/9 | 205/8136 | 0.205299688 |
| dagu vs zangga | ko04514 | Cell adhesion molecules | 1/7 | 267/8136 | 0.208370113 |
| jiacha vs zangga | ko04514 | Cell adhesion molecules | 1/7 | 267/8136 | 0.208370113 |
| zangga vs milin | ko04514 | Cell adhesion molecules | 1/7 | 267/8136 | 0.208370113 |
| bomi vs zangga | ko04722 | Neurotrophin signaling pathway | 1/13 | 160/8136 | 0.227708845 |
| bomi vs zangga | ko04910 | Insulin signaling pathway | 1/13 | 173/8136 | 0.243925596 |
| zangmu vs zangga | ko04514 | Cell adhesion molecules | 1/9 | 267/8136 | 0.259521933 |
| bomi vs zangga | ko04550 | Signaling pathways regulating pluripotency of stem cells | 1/13 | 195/8136 | 0.270655048 |
| bomi vs zangga | ko04141 | Protein processing in endoplasmic reticulum | 1/13 | 199/8136 | 0.275420195 |
| bomi vs zangga | ko04210 | Apoptosis | 1/13 | 205/8136 | 0.282514056 |
| bomi vs zangga | ko05226 | Gastric cancer | 1/13 | 206/8136 | 0.283690114 |
| bomi vs zangga | ko04514 | Cell adhesion molecules | 1/13 | 267/8136 | 0.352155987 |
| bomi vs zangga | ko05165 | Human papillomavirus infection | 1/13 | 417/8136 | 0.495656824 |

x o’connori

| GeneID |
| --- |
| Soc_16G0006340/Soc_19G0002040 |
| Soc_20G0005830/Soc_20G0005900 |
| Soc_20G0005830/Soc_20G0005900 |
| Soc_14G0000910 |
| Soc_14G0000910 |
| Soc_20G0005830/Soc_20G0005900 |
| Soc_3G0009700 |
| Soc_3G0009700 |
| Soc_20G0005860 |
| Soc_20G0005860 |
| Soc_20G0005860 |
| Soc_16G0006340 |
| Soc_16G0006340 |
| Soc_16G0006340 |
| Soc_16G0006340 |
| Soc_16G0006340 |
| Soc_3G0009700 |
| Soc_3G0009700 |
| Soc_3G0009700 |
| Soc_3G0009700 |
| Soc_3G0009700 |
| Soc_3G0009700 |
| Soc_3G0009700 |
| Soc_3G0009700 |
| Soc_3G0009700 |
| Soc_3G0009700 |
| Soc_17G0007270 |
| Soc_3G0009700 |
| Soc_3G0009700 |
| Soc_17G0007270 |
| Soc_20G0005830/Soc_20G0005900 |
| Soc_3G0009700 |
| Soc_19G0002040 |
| Soc_17G0007270 |
| Soc_20G0005860 |
| Soc_3G0009700 |

| |
|---|
| Soc_3G0009700 |
| Soc_14G0000300 |
| Soc_14G0000300 |
| Soc_3G0009700 |
| Soc_3G0009700 |
| Soc_17G0007270 |
| Soc_3G0009700 |
| Soc_19G0002040 |
| Soc_19G0002040 |
| Soc_17G0007270 |
| Soc_3G0009700 |
| Soc_20G0005900 |
| Soc_20G0005900 |
| Soc_20G0005900 |
| Soc_16G0006340 |
| Soc_16G0006340 |
| Soc_3G0009700 |
| Soc_19G0002040 |
| Soc_14G0000910 |
| Soc_14G0000910 |
| Soc_14G0000910 |
| Soc_3G0009700 |
| Soc_16G0007700 |
| Soc_16G0007700 |
| Soc_3G0009700 |
| Soc_20G0005860 |
| Soc_20G0005860 |
| Soc_3G0009700 |
| Soc_17G0007270 |
| Soc_3G0009700 |
| Soc_U0000420 |
| Soc_19G0002040 |
| Soc_19G0002040 |
| Soc_16G0006340 |
| Soc_16G0006340 |
| Soc_14G0000910 |
| Soc_16G0007700 |
| Soc_17G0007270 |

| |
|---|
| Soc_U0000420 |
| Soc_19G0002040 |
| Soc_20G0005860 |
| Soc_20G0005900 |
| Soc_20G0005900 |
| Soc_20G0005900 |
| Soc_3G0009700 |
| Soc_3G0009700 |
| Soc_19G0002040 |
| Soc_14G0000040 |
| Soc_14G0000040 |
| Soc_14G0000040 |
| Soc_19G0002040 |
| Soc_20G0000330 |
| Soc_20G0000330 |
| Soc_17G0007270 |
| Soc_14G0000910 |
| Soc_16G0007700 |
| Soc_20G0005860 |
| Soc_20G0000330 |
| Soc_20G0000330 |
| Soc_20G0000330 |
| Soc_20G0000330 |
| Soc_U0000420 |
| Soc_14G0000040 |
| Soc_16G0006340 |
| Soc_20G0000330 |
| Soc_20G0000330 |
| Soc_20G0000330 |
| Soc_U0000420 |
| Soc_14G0000040 |
| Soc_14G0000040 |
| Soc_14G0000040 |
| Soc_20G0000330 |
| Soc_20G0000330 |
| Soc_16G0007750 |
| Soc_20G0005900 |
| Soc_20G0005900 |

| |
|---|
| Soc_16G0006720 |
| Soc_14G0000040 |
| Soc_2G0017770 |
| Soc_2G0017770 |
| Soc_20G0000330 |
| Soc_14G0000040 |
| Soc_14G0000300 |
| Soc_14G0000300 |
| Soc_14G0000300 |
| Soc_U0000420 |
| Soc_16G0007700 |
| Soc_16G0007700 |
| Soc_20G0005900 |
| Soc_20G0005900 |
| Soc_14G0000040 |
| Soc_16G0007750 |
| Soc_16G0006720 |
| Soc_20G0005900 |
| Soc_16G0006880 |
| Soc_14G0000300 |
| Soc_16G0007700 |
| Soc_14G0000750 |
| Soc_14G0000750 |
| Soc_14G0000750 |
| Soc_16G0007220 |
| Soc_16G0007220 |
| Soc_14G0000750 |
| Soc_14G0000300 |
| Soc_U0000420 |
| Soc_16G0007700 |
| Soc_20G0005900 |
| Soc_14G0000750 |
| Soc_16G0006720 |
