# Supplementary material for: Population structure and adaptability analysis of Schizothorax o’connori based on whole-genome resequencing
Source: BMC Genomics. 2024 Feb 6;25:145. doi: 10.1186/s12864-024-09975-9 (PMC10845765; doi:10.1186/s12864-024-09975-9)
Supplement: Supplementary file 4 — Additional file 4: Supplementary Table 4. GO annotation for XP-EHH analysis. [file 12864_2024_9975_MOESM4_ESM.pdf]

Supplementary Table 4. GO annotation for XP-EHH analysis

population	ID	Descriptio	GeneRatio	BgRatio	pvalue	p.adjust	qvalue	geneID	Count
bomi vs milin	GO:0008417	fucosyltrar	4/468	13/15848	0.000434	0.091612	0.087293	Soc_13G0(	4
bomi vs milin	GO:0007596	blood coa	4/468	16/15848	0.00103	0.108669	0.103546	Soc_18G0(	4
bomi vs milin	GO:0004950	chemokine	4/468	23/15848	0.004256	0.299321	0.28521	Soc_15G0(	4
bomi vs milin	GO:0003924	GTPase ac	18/468	314/15848	0.005782	0.304985	0.290607	Soc_13G0(	18
bomi vs milin	GO:0005272	sodium ch	3/468	15/15848	0.008935	0.365828	0.348581	Soc_22G0(	3
bomi vs milin	GO:0005267	potassium	4/468	32/15848	0.014032	0.365828	0.348581	Soc_14G0(	4
bomi vs milin	GO:0006486	protein gly	7/468	87/15848	0.014397	0.365828	0.348581	Soc_10G0(	7
bomi vs milin	GO:0006281	DNA repai	7/468	88/15848	0.015257	0.365828	0.348581	Soc_14G0(	7
bomi vs milin	GO:0006935	chemotaxi	4/468	33/15848	0.015604	0.365828	0.348581	Soc_15G0(	4
bomi vs milin	GO:0004497	monooxyg	3/468	19/15848	0.017439	0.367965	0.350618	Soc_20G0(	3
bomi vs milin	GO:0005507	copper ior	3/468	20/15848	0.020076	0.372004	0.354466	Soc_21G0(	3
bomi vs milin	GO:0007605	sensory pe	3/468	21/15848	0.02292	0.372004	0.354466	Soc_3G000	3
bomi vs milin	GO:0043087	regulation	3/468	21/15848	0.02292	0.372004	0.354466	Soc_3G000	3
bomi vs milin	GO:0030971	receptor ty	2/468	10/15848	0.033471	0.481286	0.458596	Soc_15G0(	2
bomi vs milin	GO:0008081	phosphori	3/468	25/15848	0.036357	0.481286	0.458596	Soc_14G0(	3
bomi vs milin	GO:0007169	transmeml	2/468	11/15848	0.040122	0.481286	0.458596	Soc_11G0(	2
bomi vs milin	GO:0030414	peptidase	2/468	11/15848	0.040122	0.481286	0.458596	Soc_19G0(	2
bomi vs milin	GO:0009607	response t	3/468	27/15848	0.044292	0.481286	0.458596	Soc_12G0(	3
bomi vs milin	GO:0004714	transmeml	2/468	12/15848	0.047224	0.481286	0.458596	Soc_11G0(	2
bomi vs milin	GO:1902476	chloride tr	2/468	12/15848	0.047224	0.481286	0.458596	Soc_24G0(	2
bomi vs milin	GO:0004713	protein tyr	6/468	91/15848	0.052443	0.481286	0.458596	Soc_11G0(	6
bomi vs milin	GO:0004620	phospholi	2/468	13/15848	0.054743	0.481286	0.458596	Soc_5G000	2
bomi vs milin	GO:0009395	phospholi	2/468	13/15848	0.054743	0.481286	0.458596	Soc_5G000	2
bomi vs milin	GO:0032947	molecular	2/468	13/15848	0.054743	0.481286	0.458596	Soc_13G0(	2
bomi vs milin	GO:0004725	protein tyr	4/468	50/15848	0.059626	0.503243	0.479518	Soc_13G0(	4
bomi vs milin	GO:0071805	potassium	3/468	31/15848	0.062489	0.507124	0.483216	Soc_14G0(	3
bomi vs milin	GO:0042157	lipoprotein	3/468	32/15848	0.067502	0.527516	0.502647	Soc_11G0(	3
bomi vs milin	GO:0016042	lipid catab	2/468	16/15848	0.079507	0.599142	0.570896	Soc_13G0(	2
bomi vs milin	GO:0004879	nuclear rec	2/468	17/15848	0.088404	0.621772	0.592459	Soc_20G0(	2
bomi vs milin	GO:0051536	iron-sulfur	2/468	17/15848	0.088404	0.621772	0.592459	Soc_23G0(	2
bomi vs milin	GO:0003684	damaged	2/468	18/15848	0.097578	0.66416	0.632849	Soc_7G000	2
bomi vs milin	GO:0042127	regulation	2/468	20/15848	0.116665	0.753674	0.718142	Soc_10G0(	2
bomi vs milin	GO:0030170	pyridoxal p	3/468	41/15848	0.119998	0.753674	0.718142	Soc_12G0(	3
bomi vs milin	GO:0005506	iron ion bi	6/468	114/15848	0.121445	0.753674	0.718142	Soc_12G0(	6
bomi vs milin	GO:0005044	scavenger	4/468	66/15848	0.130599	0.787324	0.750207	Soc_21G0(	4
bomi vs milin	GO:0004129	cytochrom	2/468	23/15848	0.146814	0.796243	0.758705	Soc_5G000	2

bomi vs milin	GO:0016459	myosin co	4/468	70/15848	0.152153	0.796243	0.758705	Soc_22G0(	4
bomi vs milin	GO:0004867	serine-typ	3/468	46/15848	0.153988	0.796243	0.758705	Soc_14G0(	3
bomi vs milin	GO:0006470	protein de	6/468	123/15848	0.156704	0.796243	0.758705	Soc_11G0(	6
bomi vs milin	GO:0003774	motor acti	4/468	71/15848	0.157741	0.796243	0.758705	Soc_22G0(	4
bomi vs milin	GO:0006869	lipid trans	3/468	47/15848	0.161118	0.796243	0.758705	Soc_11G0(	3
bomi vs milin	GO:0055114	oxidation-	16/468	413/15848	0.164064	0.796243	0.758705	Soc_11G0(	16
bomi vs milin	GO:0005923	bicellular t	4/468	74/15848	0.174945	0.796243	0.758705	Soc_13G0(	4
bomi vs milin	GO:0042981	regulation	4/468	77/15848	0.192755	0.796243	0.758705	Soc_11G0(	4
bomi vs milin	GO:0003950	NAD+ AD	2/468	28/15848	0.199848	0.796243	0.758705	Soc_11G0(	2
bomi vs milin	GO:0008285	negative re	2/468	28/15848	0.199848	0.796243	0.758705	Soc_11G0(	2
bomi vs milin	GO:0004984	olfactory r	3/468	53/15848	0.205761	0.796243	0.758705	Soc_16G0(	3
bomi vs milin	GO:0006814	sodium ior	3/468	54/15848	0.213462	0.796243	0.758705	Soc_22G0(	3
bomi vs milin	GO:0000226	microtubu	2/468	30/15848	0.221658	0.796243	0.758705	Soc_15G0(	2
bomi vs milin	GO:0005578	extracellula	2/468	30/15848	0.221658	0.796243	0.758705	Soc_17G0(	2
bomi vs milin	GO:0005922	connexin c	2/468	31/15848	0.232635	0.796243	0.758705	Soc_3G001	2
bomi vs milin	GO:0016746	transferase	2/468	31/15848	0.232635	0.796243	0.758705	Soc_11G0(	2
bomi vs milin	GO:0008092	cytoskeleta	2/468	32/15848	0.243644	0.796243	0.758705	Soc_14G0(	2
bomi vs milin	GO:0005829	cytosol	1/468	10/15848	0.259063	0.796243	0.758705	Soc_3G00(	1
bomi vs milin	GO:0005883	neurofilam	1/468	10/15848	0.259063	0.796243	0.758705	Soc_4G00(	1
bomi vs milin	GO:0016712	oxidoredu	1/468	10/15848	0.259063	0.796243	0.758705	Soc_21G0(	1
bomi vs milin	GO:0045087	innate imn	1/468	10/15848	0.259063	0.796243	0.758705	Soc_4G00(	1
bomi vs milin	GO:0050839	cell adhesi	1/468	10/15848	0.259063	0.796243	0.758705	Soc_7G00(	1
bomi vs milin	GO:0005096	GTPase ac	4/468	88/15848	0.262111	0.796243	0.758705	Soc_10G0(	4
bomi vs milin	GO:0008289	lipid bindi	4/468	88/15848	0.262111	0.796243	0.758705	Soc_11G0(	4
bomi vs milin	GO:0046983	protein dir	9/468	236/15848	0.263824	0.796243	0.758705	Soc_1G00(	9
bomi vs milin	GO:0003707	steroid ho	3/468	61/15848	0.268798	0.796243	0.758705	Soc_19G0(	3
bomi vs milin	GO:0008076	voltage-ga	3/468	61/15848	0.268798	0.796243	0.758705	Soc_20G0(	3
bomi vs milin	GO:0043401	steroid ho	3/468	61/15848	0.268798	0.796243	0.758705	Soc_19G0(	3
bomi vs milin	GO:0005737	cytoplasm	9/468	238/15848	0.271663	0.796243	0.758705	Soc_15G0(	9
bomi vs milin	GO:0003725	double-str	1/468	11/15848	0.280957	0.796243	0.758705	Soc_4G00(	1
bomi vs milin	GO:0006888	endoplasm	1/468	11/15848	0.280957	0.796243	0.758705	Soc_7G00(	1
bomi vs milin	GO:0032958	inositol ph	1/468	11/15848	0.280957	0.796243	0.758705	Soc_8G00(	1
bomi vs milin	GO:0044212	transcripti	1/468	11/15848	0.280957	0.796243	0.758705	Soc_11G0(	1
bomi vs milin	GO:0008373	sialyltransf	2/468	36/15848	0.287777	0.796243	0.758705	Soc_10G0(	2
bomi vs milin	GO:0015031	protein tra	2/468	36/15848	0.287777	0.796243	0.758705	Soc_3G00(	2
bomi vs milin	GO:0005856	cytoskelet	4/468	93/15848	0.295095	0.796243	0.758705	Soc_11G0(	4
bomi vs milin	GO:0007399	nervous sy	2/468	37/15848	0.298786	0.796243	0.758705	Soc_4G00(	2
bomi vs milin	GO:0004707	MAP kinas	1/468	12/15848	0.302206	0.796243	0.758705	Soc_18G0(	1

bomi vs milin	GO:0007179	transformi	1/468	12/15848	0.302206	0.796243	0.758705	Soc_6G000	1
bomi vs milin	GO:0008283	cell popula	1/468	12/15848	0.302206	0.796243	0.758705	Soc_20G00	1
bomi vs milin	GO:0031175	neuron pr	1/468	12/15848	0.302206	0.796243	0.758705	Soc_15G00	1
bomi vs milin	GO:0046982	protein he	1/468	12/15848	0.302206	0.796243	0.758705	Soc_7G000	1
bomi vs milin	GO:0051056	regulation	1/468	12/15848	0.302206	0.796243	0.758705	Soc_23G00	1
bomi vs milin	GO:0006260	DNA replic	2/468	39/15848	0.320712	0.796243	0.758705	Soc_1G001	2
bomi vs milin	GO:0003730	mRNA 3'-	1/468	13/15848	0.322828	0.796243	0.758705	Soc_11G00	1
bomi vs milin	GO:0005911	cell-cell ju	1/468	13/15848	0.322828	0.796243	0.758705	Soc_7G000	1
bomi vs milin	GO:0008253	5'-nucleot	1/468	13/15848	0.322828	0.796243	0.758705	Soc_6G000	1
bomi vs milin	GO:0016747	transferase	1/468	13/15848	0.322828	0.796243	0.758705	Soc_12G00	1
bomi vs milin	GO:0007049	cell cycle	2/468	40/15848	0.331614	0.796243	0.758705	Soc_1G002	2
bomi vs milin	GO:0051260	protein ho	3/468	69/15848	0.33364	0.796243	0.758705	Soc_20G00	3
bomi vs milin	GO:0003714	transcripti	1/468	14/15848	0.342841	0.796243	0.758705	Soc_3G000	1
bomi vs milin	GO:0005158	insulin rec	1/468	14/15848	0.342841	0.796243	0.758705	Soc_4G000	1
bomi vs milin	GO:0005635	nuclear en	1/468	14/15848	0.342841	0.796243	0.758705	Soc_15G00	1
bomi vs milin	GO:0006606	protein im	1/468	14/15848	0.342841	0.796243	0.758705	Soc_6G000	1
bomi vs milin	GO:0007165	signal tran	12/468	351/15848	0.343403	0.796243	0.758705	Soc_10G00	12
bomi vs milin	GO:0003729	mRNA bin	1/468	15/15848	0.362265	0.799882	0.762172	Soc_10G00	1
bomi vs milin	GO:0004993	G protein-	1/468	15/15848	0.362265	0.799882	0.762172	Soc_20G00	1
bomi vs milin	GO:0008033	tRNA proc	1/468	15/15848	0.362265	0.799882	0.762172	Soc_23G00	1
bomi vs milin	GO:0046873	metal ion	1/468	15/15848	0.362265	0.799882	0.762172	Soc_19G00	1
bomi vs milin	GO:0004842	ubiquitin-	4/468	105/15848	0.375695	0.799882	0.762172	Soc_1G002	4
bomi vs milin	GO:0006310	DNA recor	1/468	16/15848	0.381115	0.799882	0.762172	Soc_4G000	1
bomi vs milin	GO:0042803	protein ho	1/468	16/15848	0.381115	0.799882	0.762172	Soc_7G000	1
bomi vs milin	GO:0003723	RNA bindi	7/468	202/15848	0.387937	0.799882	0.762172	Soc_10G00	7
bomi vs milin	GO:0042626	ATPase-co	2/468	46/15848	0.395762	0.799882	0.762172	Soc_13G00	2
bomi vs milin	GO:0008083	growth fac	4/468	108/15848	0.395841	0.799882	0.762172	Soc_14G00	4
bomi vs milin	GO:0006520	cellular am	1/468	17/15848	0.39941	0.799882	0.762172	Soc_8G000	1
bomi vs milin	GO:0005249	voltage-ga	3/468	79/15848	0.414182	0.799882	0.762172	Soc_20G00	3
bomi vs milin	GO:0004190	aspartic-ty	1/468	18/15848	0.417164	0.799882	0.762172	Soc_24G00	1
bomi vs milin	GO:0017137	Rab GTPas	1/468	18/15848	0.417164	0.799882	0.762172	Soc_1G002	1
bomi vs milin	GO:0042310	vasoconstr	1/468	18/15848	0.417164	0.799882	0.762172	Soc_20G00	1
bomi vs milin	GO:0007154	cell comm	2/468	49/15848	0.426781	0.799882	0.762172	Soc_3G001	2
bomi vs milin	GO:0003824	catalytic a	6/468	178/15848	0.429828	0.799882	0.762172	Soc_14G00	6
bomi vs milin	GO:0003333	amino acid	1/468	19/15848	0.434395	0.799882	0.762172	Soc_1G000	1
bomi vs milin	GO:0005201	extracellul	1/468	19/15848	0.434395	0.799882	0.762172	Soc_18G00	1
bomi vs milin	GO:0015171	amino acid	1/468	19/15848	0.434395	0.799882	0.762172	Soc_1G000	1
bomi vs milin	GO:0016627	oxidoredu	1/468	19/15848	0.434395	0.799882	0.762172	Soc_15G00	1

bomi vs milin	GO:0009058	biosynthet	2/468	50/15848	0.436935	0.799882	0.762172	Soc_12G00	2
bomi vs milin	GO:0005198	structural	6/468	181/15848	0.445559	0.799882	0.762172	Soc_14G00	6
bomi vs milin	GO:0008168	methyltran	2/468	51/15848	0.446992	0.799882	0.762172	Soc_15G00	2
bomi vs milin	GO:0000398	mRNA spli	1/468	20/15848	0.451118	0.799882	0.762172	Soc_6G000	1
bomi vs milin	GO:0004114	3',5'-cyclic	1/468	20/15848	0.451118	0.799882	0.762172	Soc_18G00	1
bomi vs milin	GO:0004843	thiol-depe	1/468	20/15848	0.451118	0.799882	0.762172	Soc_3G000	1
bomi vs milin	GO:0098609	cell-cell ad	1/468	20/15848	0.451118	0.799882	0.762172	Soc_11G00	1
bomi vs milin	GO:0005242	inward rec	1/468	21/15848	0.467347	0.80171	0.763914	Soc_1G002	1
bomi vs milin	GO:0005540	hyaluronic	1/468	21/15848	0.467347	0.80171	0.763914	Soc_10G00	1
bomi vs milin	GO:0006338	chromatin	1/468	21/15848	0.467347	0.80171	0.763914	Soc_8G000	1
bomi vs milin	GO:0007015	actin filam	1/468	21/15848	0.467347	0.80171	0.763914	Soc_7G000	1
bomi vs milin	GO:0007601	visual perc	2/468	54/15848	0.47654	0.803956	0.766054	Soc_20G00	2
bomi vs milin	GO:0005794	Golgi appa	1/468	22/15848	0.483098	0.803956	0.766054	Soc_13G00	1
bomi vs milin	GO:0045892	negative re	1/468	22/15848	0.483098	0.803956	0.766054	Soc_13G00	1
bomi vs milin	GO:0022857	transmeml	3/468	88/15848	0.483898	0.803956	0.766054	Soc_18G00	3
bomi vs milin	GO:0016301	kinase acti	2/468	56/15848	0.495698	0.803995	0.766092	Soc_6G000	2
bomi vs milin	GO:0030001	metal ion t	1/468	23/15848	0.498383	0.803995	0.766092	Soc_19G00	1
bomi vs milin	GO:0042613	MHC class	1/468	23/15848	0.498383	0.803995	0.766092	Soc_13G00	1
bomi vs milin	GO:0006813	potassium	4/468	125/15848	0.506756	0.803995	0.766092	Soc_1G002	4
bomi vs milin	GO:0005262	calcium ch	1/468	24/15848	0.513218	0.803995	0.766092	Soc_9G000	1
bomi vs milin	GO:0008378	galactosylt	1/468	24/15848	0.513218	0.803995	0.766092	Soc_18G00	1
bomi vs milin	GO:0019882	antigen pr	1/468	24/15848	0.513218	0.803995	0.766092	Soc_13G00	1
bomi vs milin	GO:0008138	protein tyr	2/468	58/15848	0.514405	0.803995	0.766092	Soc_11G00	2
bomi vs milin	GO:0008565	obsolete p	1/468	25/15848	0.527615	0.818579	0.779988	Soc_6G000	1
bomi vs milin	GO:0007268	chemical s	1/468	26/15848	0.541587	0.826742	0.787766	Soc_2G000	1
bomi vs milin	GO:0009966	regulation	1/468	26/15848	0.541587	0.826742	0.787766	Soc_11G00	1
bomi vs milin	GO:0005200	structural	1/468	27/15848	0.555146	0.826742	0.787766	Soc_13G00	1
bomi vs milin	GO:0005328	neurotrans	1/468	27/15848	0.555146	0.826742	0.787766	Soc_9G000	1
bomi vs milin	GO:0009055	electron tr	1/468	27/15848	0.555146	0.826742	0.787766	Soc_2G000	1
bomi vs milin	GO:0016311	dephosph	3/468	98/15848	0.556385	0.826742	0.787766	Soc_11G00	3
bomi vs milin	GO:0016567	protein ub	2/468	64/15848	0.567704	0.832725	0.793467	Soc_20G00	2
bomi vs milin	GO:0043547	positive re	1/468	28/15848	0.568305	0.832725	0.793467	Soc_10G00	1
bomi vs milin	GO:0005234	extracellul	1/468	29/15848	0.581076	0.84467	0.804849	Soc_6G000	1
bomi vs milin	GO:0046872	metal ion I	6/468	210/15848	0.59013	0.84467	0.804849	Soc_12G00	6
bomi vs milin	GO:0009190	cyclic nucl	1/468	30/15848	0.59347	0.84467	0.804849	Soc_15G00	1
bomi vs milin	GO:0016849	phosphoru	1/468	30/15848	0.59347	0.84467	0.804849	Soc_15G00	1
bomi vs milin	GO:0005887	integral co	4/468	141/15848	0.60209	0.84467	0.804849	Soc_13G00	4
bomi vs milin	GO:0006897	endocytos	1/468	31/15848	0.605497	0.84467	0.804849	Soc_5G000	1

bomi vs milin	GO:0006811	ion transp	8/468	285/15848	0.60795	0.84467	0.804849	Soc_20G0		8
bomi vs milin	GO:0016791	phosphata	3/468	106/15848	0.609779	0.84467	0.804849	Soc_11G0		3
bomi vs milin	GO:0007017	microtubu	1/468	32/15848	0.61717	0.84467	0.804849	Soc_13G0		1
bomi vs milin	GO:0016887	ATPase ac	2/468	71/15848	0.624386	0.84467	0.804849	Soc_13G0		2
bomi vs milin	GO:0004970	ionotropic	1/468	33/15848	0.628498	0.84467	0.804849	Soc_6G000		1
bomi vs milin	GO:0005886	plasma me	1/468	33/15848	0.628498	0.84467	0.804849	Soc_15G0		1
bomi vs milin	GO:0016616	oxidoredu	1/468	33/15848	0.628498	0.84467	0.804849	Soc_15G0		1
bomi vs milin	GO:0005667	transcripti	1/468	34/15848	0.639492	0.853044	0.812829	Soc_6G000		1
bomi vs milin	GO:0005216	ion channe	7/468	258/15848	0.642911	0.853044	0.812829	Soc_20G0		7
bomi vs milin	GO:0016705	oxidoredu	2/468	74/15848	0.646858	0.853044	0.812829	Soc_12G0		2
bomi vs milin	GO:0006836	neurotrans	1/468	36/15848	0.660514	0.865643	0.824833	Soc_9G000		1
bomi vs milin	GO:0008080	N-acetyltr	1/468	38/15848	0.680314	0.886088	0.844314	Soc_15G0		1
bomi vs milin	GO:0005783	endoplasm	1/468	39/15848	0.689777	0.892901	0.850807	Soc_9G000		1
bomi vs milin	GO:0016192	vesicle-me	2/468	82/15848	0.701569	0.902629	0.860075	Soc_12G0		2
bomi vs milin	GO:0005615	extracellul	2/468	83/15848	0.707889	0.90524	0.862564	Soc_14G0		2
bomi vs milin	GO:0005089	Rho guany	2/468	84/15848	0.714097	0.905306	0.862626	Soc_11G0		2
bomi vs milin	GO:0051082	unfolded p	1/468	42/15848	0.716522	0.905306	0.862626	Soc_15G0		1
bomi vs milin	GO:0006816	calcium io	1/468	43/15848	0.724915	0.910459	0.867537	Soc_9G000		1
bomi vs milin	GO:0005882	intermedia	2/468	87/15848	0.732061	0.913993	0.870904	Soc_4G000		2
bomi vs milin	GO:0035023	regulation	2/468	88/15848	0.737832	0.914291	0.871188	Soc_11G0		2
bomi vs milin	GO:0005874	microtubu	1/468	45/15848	0.740966	0.914291	0.871188	Soc_13G0		1
bomi vs milin	GO:0003700	DNA-bind	12/468	476/15848	0.752401	0.915947	0.872766	Soc_11G0		12
bomi vs milin	GO:0020037	heme bind	2/468	91/15848	0.754513	0.915947	0.872766	Soc_12G0		2
bomi vs milin	GO:0016757	transferase	1/468	47/15848	0.756081	0.915947	0.872766	Soc_11G0		1
bomi vs milin	GO:0031012	extracellul	1/468	48/15848	0.763306	0.915947	0.872766	Soc_5G000		1
bomi vs milin	GO:0015074	DNA integ	2/468	94/15848	0.770271	0.915947	0.872766	Soc_12G0		2
bomi vs milin	GO:0016491	oxidoredu	4/468	179/15848	0.778713	0.915947	0.872766	Soc_11G0		4
bomi vs milin	GO:0008017	microtubu	2/468	96/15848	0.780282	0.915947	0.872766	Soc_13G0		2
bomi vs milin	GO:0007264	small GTPa	3/468	139/15848	0.782493	0.915947	0.872766	Soc_10G0		3
bomi vs milin	GO:0008234	cysteine-ty	1/468	52/15848	0.790131	0.915947	0.872766	Soc_20G0		1
bomi vs milin	GO:0036459	thiol-depe	1/468	52/15848	0.790131	0.915947	0.872766	Soc_8G000		1
bomi vs milin	GO:0004252	serine-typ	3/468	143/15848	0.79841	0.915947	0.872766	Soc_1G000		3
bomi vs milin	GO:0008152	metabolic	5/468	226/15848	0.801294	0.915947	0.872766	Soc_11G0		5
bomi vs milin	GO:0000287	magnesiur	1/468	54/15848	0.802383	0.915947	0.872766	Soc_6G000		1
bomi vs milin	GO:0004872	signaling r	1/468	55/15848	0.808238	0.915947	0.872766	Soc_6G000		1
bomi vs milin	GO:0070588	calcium io	1/468	55/15848	0.808238	0.915947	0.872766	Soc_9G000		1
bomi vs milin	GO:0006396	RNA proce	1/468	56/15848	0.813921	0.915947	0.872766	Soc_5G001		1
bomi vs milin	GO:0005739	mitochond	1/468	57/15848	0.819435	0.915947	0.872766	Soc_8G000		1

bomi vs milin	GO:0007155	cell adhesi	3/468	149/15848	0.820452	0.915947	0.872766	Soc_15G0	3
bomi vs milin	GO:0005085	guanyl-nu	1/468	58/15848	0.824787	0.915947	0.872766	Soc_10G0	1
bomi vs milin	GO:0006915	apoptotic	1/468	60/15848	0.835019	0.922456	0.878968	Soc_8G000	1
bomi vs milin	GO:0005622	intracellula	9/468	400/15848	0.83961	0.922696	0.879197	Soc_10G0	9
bomi vs milin	GO:0016579	protein de	1/468	63/15848	0.849261	0.927929	0.884183	Soc_8G000	1
bomi vs milin	GO:0006886	intracellula	2/468	115/15848	0.857739	0.927929	0.884183	Soc_1G001	2
bomi vs milin	GO:0007018	microtubu	1/468	66/15848	0.862276	0.927929	0.884183	Soc_1G000	1
bomi vs milin	GO:0008146	sulfotransf	1/468	66/15848	0.862276	0.927929	0.884183	Soc_13G0	1
bomi vs milin	GO:0003777	microtubu	1/468	67/15848	0.86636	0.927929	0.884183	Soc_1G000	1
bomi vs milin	GO:0035556	intracellula	3/468	170/15848	0.882107	0.940023	0.895707	Soc_15G0	3
bomi vs milin	GO:0006629	lipid metal	1/468	76/15848	0.898085	0.950184	0.905388	Soc_4G000	1
bomi vs milin	GO:0005576	extracellul	6/468	311/15848	0.900782	0.950184	0.905388	Soc_11G0	6
bomi vs milin	GO:0016787	hydrolase	2/468	134/15848	0.909473	0.950184	0.905388	Soc_20G0	2
bomi vs milin	GO:0007275	multicellul	1/468	80/15848	0.909655	0.950184	0.905388	Soc_11G0	1
bomi vs milin	GO:0000786	nucleosom	2/468	137/15848	0.915821	0.951912	0.907035	Soc_3G001	2
bomi vs milin	GO:0043565	sequence-	3/468	191/15848	0.924182	0.953964	0.90899	Soc_20G0	3
bomi vs milin	GO:0005840	ribosome	1/468	87/15848	0.926837	0.953964	0.90899	Soc_18G0	1
bomi vs milin	GO:0006412	translation	1/468	91/15848	0.935148	0.957846	0.912689	Soc_18G0	1
bomi vs milin	GO:0003735	structural	1/468	97/15848	0.945882	0.96416	0.918705	Soc_18G0	1
bomi vs milin	GO:0006955	immune re	1/468	127/15848	0.978123	0.989998	0.943326	Soc_13G0	1
bomi vs milin	GO:0007156	homophili	1/468	131/15848	0.980614	0.989998	0.943326	Soc_22G0	1
bomi vs milin	GO:0004888	transmem	1/468	165/15848	0.993071	0.995385	0.948459	Soc_4G000	1
bomi vs milin	GO:0006508	proteolysis	5/468	422/15848	0.995385	0.995385	0.948459	Soc_1G000	5
bomi vs linzhi	GO:0000786	nucleosom	17/420	137/15848	1.30E-07	2.68E-05	2.55E-05	Soc_21G0	17
bomi vs linzhi	GO:0004866	endopepti	5/420	22/15848	0.000232	0.019743	0.018764	Soc_5G000	5
bomi vs linzhi	GO:0008417	fucosyltrar	4/420	13/15848	0.000288	0.019743	0.018764	Soc_13G0	4
bomi vs linzhi	GO:0045095	keratin fila	3/420	12/15848	0.003402	0.175194	0.16651	Soc_15G0	3
bomi vs linzhi	GO:0006397	mRNA pro	4/420	29/15848	0.006834	0.196244	0.186517	Soc_3G000	4
bomi vs linzhi	GO:0007596	blood coa	3/420	16/15848	0.008004	0.196244	0.186517	Soc_20G0	3
bomi vs linzhi	GO:0005882	intermedia	7/420	87/15848	0.008281	0.196244	0.186517	Soc_15G0	7
bomi vs linzhi	GO:0006486	protein gly	7/420	87/15848	0.008281	0.196244	0.186517	Soc_13G0	7
bomi vs linzhi	GO:0006334	nucleosom	5/420	48/15848	0.008574	0.196244	0.186517	Soc_3G000	5
bomi vs linzhi	GO:0008168	methyltrar	5/420	51/15848	0.01103	0.227212	0.21595	Soc_18G0	5
bomi vs linzhi	GO:0004888	transmem	10/420	165/15848	0.012636	0.236635	0.224906	Soc_13G0	10
bomi vs linzhi	GO:0007166	cell surfac	6/420	86/15848	0.026596	0.335938	0.319287	Soc_14G0	6
bomi vs linzhi	GO:0004571	mannosyl-	2/420	10/15848	0.02739	0.335938	0.319287	Soc_6G001	2
bomi vs linzhi	GO:0005883	neurofilam	2/420	10/15848	0.02739	0.335938	0.319287	Soc_18G0	2
bomi vs linzhi	GO:0050662	obsolete c	2/420	10/15848	0.02739	0.335938	0.319287	Soc_12G0	2

bomi vs linzhi	GO:0022848	acetylcholi	3/420	25/15848	0.02759	0.335938	0.319287	Soc_4G000		3
bomi vs linzhi	GO:0003700	DNA-bind	20/420	476/15848	0.029111	0.335938	0.319287	Soc_11G0		20
bomi vs linzhi	GO:0022857	transmeml	6/420	88/15848	0.029354	0.335938	0.319287	Soc_18G0		6
bomi vs linzhi	GO:1902476	chloride tr	2/420	12/15848	0.038798	0.420648	0.399799	Soc_24G0		2
bomi vs linzhi	GO:0045211	postsynap	3/420	31/15848	0.048035	0.494761	0.470238	Soc_4G000		3
bomi vs linzhi	GO:0006364	rRNA proc	2/420	15/15848	0.058601	0.524863	0.498848	Soc_5G000		2
bomi vs linzhi	GO:0008033	tRNA proc	2/420	15/15848	0.058601	0.524863	0.498848	Soc_14G0		2
bomi vs linzhi	GO:0019905	syntaxin bi	2/420	15/15848	0.058601	0.524863	0.498848	Soc_12G0		2
bomi vs linzhi	GO:0008138	protein tyr	4/420	58/15848	0.067466	0.549471	0.522236	Soc_11G0		4
bomi vs linzhi	GO:0008373	sialyltransf	3/420	36/15848	0.069351	0.549471	0.522236	Soc_24G0		3
bomi vs linzhi	GO:0015031	protein tra	3/420	36/15848	0.069351	0.549471	0.522236	Soc_20G0		3
bomi vs linzhi	GO:0004190	aspartic-ty	2/420	18/15848	0.081101	0.618769	0.588099	Soc_12G0		2
bomi vs linzhi	GO:0000398	mRNA spli	2/420	20/15848	0.097332	0.650594	0.618347	Soc_23G0		2
bomi vs linzhi	GO:0000413	protein pe	2/420	20/15848	0.097332	0.650594	0.618347	Soc_21G0		2
bomi vs linzhi	GO:0006954	inflammato	2/420	20/15848	0.097332	0.650594	0.618347	Soc_19G0		2
bomi vs linzhi	GO:0003755	peptidyl-p	2/420	21/15848	0.105762	0.650594	0.618347	Soc_21G0		2
bomi vs linzhi	GO:0006470	protein de	6/420	123/15848	0.108754	0.650594	0.618347	Soc_11G0		6
bomi vs linzhi	GO:0016459	myosin co	4/420	70/15848	0.114712	0.650594	0.618347	Soc_10G0		4
bomi vs linzhi	GO:0016311	dephosph	5/420	98/15848	0.118872	0.650594	0.618347	Soc_11G0		5
bomi vs linzhi	GO:0003774	motor acti	4/420	71/15848	0.119173	0.650594	0.618347	Soc_10G0		4
bomi vs linzhi	GO:0005230	extracellul	4/420	71/15848	0.119173	0.650594	0.618347	Soc_13G0		4
bomi vs linzhi	GO:0004298	threonine-	2/420	23/15848	0.123171	0.650594	0.618347	Soc_14G0		2
bomi vs linzhi	GO:0005839	proteasom	2/420	23/15848	0.123171	0.650594	0.618347	Soc_14G0		2
bomi vs linzhi	GO:0051603	proteolysis	2/420	23/15848	0.123171	0.650594	0.618347	Soc_14G0		2
bomi vs linzhi	GO:0007010	cytoskelet	2/420	25/15848	0.141207	0.727216	0.691171	Soc_15G0		2
bomi vs linzhi	GO:0005789	endoplasm	2/420	26/15848	0.150425	0.737799	0.701229	Soc_12G0		2
bomi vs linzhi	GO:0007160	cell-matrix	2/420	26/15848	0.150425	0.737799	0.701229	Soc_19G0		2
bomi vs linzhi	GO:0005249	voltage-ga	4/420	79/15848	0.157393	0.74796	0.710887	Soc_14G0		4
bomi vs linzhi	GO:0009607	response t	2/420	27/15848	0.159758	0.74796	0.710887	Soc_12G0		2
bomi vs linzhi	GO:0003950	NAD+ AD	2/420	28/15848	0.169195	0.773707	0.735358	Soc_20G0		2
bomi vs linzhi	GO:0034220	ion transm	4/420	82/15848	0.172769	0.773707	0.735358	Soc_13G0		4
bomi vs linzhi	GO:0005739	mitochond	3/420	57/15848	0.191896	0.828029	0.786987	Soc_14G0		3
bomi vs linzhi	GO:0005198	structural	7/420	181/15848	0.205475	0.828029	0.786987	Soc_15G0		7
bomi vs linzhi	GO:0008076	voltage-ga	3/420	61/15848	0.21937	0.828029	0.786987	Soc_17G0		3
bomi vs linzhi	GO:0003956	NAD(P)+-	1/420	10/15848	0.235605	0.828029	0.786987	Soc_20G0		1
bomi vs linzhi	GO:0005829	cytosol	1/420	10/15848	0.235605	0.828029	0.786987	Soc_11G0		1
bomi vs linzhi	GO:0016868	intramolec	1/420	10/15848	0.235605	0.828029	0.786987	Soc_18G0		1
bomi vs linzhi	GO:0016567	protein ub	3/420	64/15848	0.24047	0.828029	0.786987	Soc_11G0		3

bomi vs linzhi	GO:0008146	sulfotransf	3/420	66/15848	0.254718	0.828029	0.786987	Soc_13G0(	3
bomi vs linzhi	GO:0005212	structural (	1/420	11/15848	0.255875	0.828029	0.786987	Soc_21G0(	1
bomi vs linzhi	GO:0006471	protein AD	1/420	11/15848	0.255875	0.828029	0.786987	Soc_20G0(	1
bomi vs linzhi	GO:0007224	smoothene	1/420	11/15848	0.255875	0.828029	0.786987	Soc_14G0(	1
bomi vs linzhi	GO:0030414	peptidase	1/420	11/15848	0.255875	0.828029	0.786987	Soc_15G0(	1
bomi vs linzhi	GO:0044212	transcripti	1/420	11/15848	0.255875	0.828029	0.786987	Soc_11G0(	1
bomi vs linzhi	GO:0006357	regulation	4/420	97/15848	0.256053	0.828029	0.786987	Soc_11G0(	4
bomi vs linzhi	GO:0005622	intracellula	13/420	400/15848	0.264579	0.828029	0.786987	Soc_12G0(	13
bomi vs linzhi	GO:0000139	Golgi mem	1/420	12/15848	0.275609	0.828029	0.786987	Soc_12G0(	1
bomi vs linzhi	GO:0000724	double-str	1/420	12/15848	0.275609	0.828029	0.786987	Soc_11G0(	1
bomi vs linzhi	GO:0004707	MAP kinas	1/420	12/15848	0.275609	0.828029	0.786987	Soc_15G0(	1
bomi vs linzhi	GO:0019752	carboxylic	1/420	12/15848	0.275609	0.828029	0.786987	Soc_22G0(	1
bomi vs linzhi	GO:0031175	neuron pr	1/420	12/15848	0.275609	0.828029	0.786987	Soc_15G0(	1
bomi vs linzhi	GO:0003723	RNA bindi	7/420	202/15848	0.289051	0.828029	0.786987	Soc_14G0(	7
bomi vs linzhi	GO:0008253	5'-nucleot	1/420	13/15848	0.294822	0.828029	0.786987	Soc_17G0(	1
bomi vs linzhi	GO:0008271	secondary	1/420	13/15848	0.294822	0.828029	0.786987	Soc_15G0(	1
bomi vs linzhi	GO:0008272	sulfate tra	1/420	13/15848	0.294822	0.828029	0.786987	Soc_15G0(	1
bomi vs linzhi	GO:0015116	sulfate tra	1/420	13/15848	0.294822	0.828029	0.786987	Soc_15G0(	1
bomi vs linzhi	GO:0004842	ubiquitin-	4/420	105/15848	0.303362	0.828029	0.786987	Soc_11G0(	4
bomi vs linzhi	GO:0016791	phosphata	4/420	106/15848	0.309353	0.828029	0.786987	Soc_11G0(	4
bomi vs linzhi	GO:0004175	endopepti	1/420	14/15848	0.313525	0.828029	0.786987	Soc_14G0(	1
bomi vs linzhi	GO:0005635	nuclear en	1/420	14/15848	0.313525	0.828029	0.786987	Soc_15G0(	1
bomi vs linzhi	GO:0005852	eukaryotic	1/420	14/15848	0.313525	0.828029	0.786987	Soc_20G0(	1
bomi vs linzhi	GO:0016477	cell migrat	1/420	14/15848	0.313525	0.828029	0.786987	Soc_15G0(	1
bomi vs linzhi	GO:0045028	G protein-	1/420	14/15848	0.313525	0.828029	0.786987	Soc_16G0(	1
bomi vs linzhi	GO:0004869	cysteine-ty	1/420	15/15848	0.331734	0.830112	0.788967	Soc_5G00(	1
bomi vs linzhi	GO:0030286	dynein cor	1/420	15/15848	0.331734	0.830112	0.788967	Soc_23G0(	1
bomi vs linzhi	GO:0046873	metal ion t	1/420	15/15848	0.331734	0.830112	0.788967	Soc_19G0(	1
bomi vs linzhi	GO:0003824	catalytic a(	6/420	178/15848	0.333586	0.830112	0.788967	Soc_11G0(	6
bomi vs linzhi	GO:0042981	regulation	3/420	77/15848	0.334463	0.830112	0.788967	Soc_1G00(	3
bomi vs linzhi	GO:0004867	serine-typ	2/420	46/15848	0.345409	0.840563	0.7989	Soc_15G0(	2
bomi vs linzhi	GO:0006869	lipid transp	2/420	47/15848	0.355072	0.840563	0.7989	Soc_12G0(	2
bomi vs linzhi	GO:0031012	extracellula	2/420	48/15848	0.364684	0.840563	0.7989	Soc_19G0(	2
bomi vs linzhi	GO:0004879	nuclear re(	1/420	17/15848	0.366719	0.840563	0.7989	Soc_11G0(	1
bomi vs linzhi	GO:0016192	vesicle-me	3/420	82/15848	0.370794	0.840563	0.7989	Soc_12G0(	3
bomi vs linzhi	GO:0003684	damaged	1/420	18/15848	0.38352	0.840563	0.7989	Soc_5G00(	1
bomi vs linzhi	GO:0008021	synaptic ve	1/420	18/15848	0.38352	0.840563	0.7989	Soc_20G0(	1
bomi vs linzhi	GO:0017137	Rab GTPas	1/420	18/15848	0.38352	0.840563	0.7989	Soc_22G0(	1

bomi vs linzhi	GO:0043565	sequence-	6/420	191/15848	0.395061	0.840563	0.7989	Soc_11G0(	6
bomi vs linzhi	GO:0004386	helicase ac	1/420	19/15848	0.399877	0.840563	0.7989	Soc_11G0(	1
bomi vs linzhi	GO:0005743	mitochond	1/420	19/15848	0.399877	0.840563	0.7989	Soc_9G000	1
bomi vs linzhi	GO:0006351	transcripti	2/420	53/15848	0.411842	0.840563	0.7989	Soc_15G0(	2
bomi vs linzhi	GO:0008289	lipid bindi	3/420	88/15848	0.413869	0.840563	0.7989	Soc_12G0(	3
bomi vs linzhi	GO:0004114	3',5'-cyclic	1/420	20/15848	0.4158	0.840563	0.7989	Soc_1G002	1
bomi vs linzhi	GO:0005104	fibroblast	1/420	20/15848	0.4158	0.840563	0.7989	Soc_11G0(	1
bomi vs linzhi	GO:0005507	copper ion	1/420	20/15848	0.4158	0.840563	0.7989	Soc_22G0(	1
bomi vs linzhi	GO:0006813	potassium	4/420	125/15848	0.423644	0.840563	0.7989	Soc_14G0(	4
bomi vs linzhi	GO:0045893	positive re	1/420	21/15848	0.431302	0.840563	0.7989	Soc_8G000	1
bomi vs linzhi	GO:0005576	extracellula	9/420	311/15848	0.441944	0.840563	0.7989	Soc_11G0(	9
bomi vs linzhi	GO:0005737	cytoplasm	7/420	238/15848	0.443945	0.840563	0.7989	Soc_11G0(	7
bomi vs linzhi	GO:0005520	insulin-like	1/420	22/15848	0.446393	0.840563	0.7989	Soc_20G0(	1
bomi vs linzhi	GO:0005794	Golgi appa	1/420	22/15848	0.446393	0.840563	0.7989	Soc_13G0(	1
bomi vs linzhi	GO:0045892	negative re	1/420	22/15848	0.446393	0.840563	0.7989	Soc_13G0(	1
bomi vs linzhi	GO:0005085	guanyl-nu	2/420	58/15848	0.457211	0.840563	0.7989	Soc_17G0(	2
bomi vs linzhi	GO:0001558	regulation	1/420	23/15848	0.461085	0.840563	0.7989	Soc_20G0(	1
bomi vs linzhi	GO:0004129	cytochrom	1/420	23/15848	0.461085	0.840563	0.7989	Soc_9G000	1
bomi vs linzhi	GO:0004890	GABA-A re	1/420	23/15848	0.461085	0.840563	0.7989	Soc_13G0(	1
bomi vs linzhi	GO:0008543	fibroblast	1/420	23/15848	0.461085	0.840563	0.7989	Soc_11G0(	1
bomi vs linzhi	GO:0030001	metal ion	1/420	23/15848	0.461085	0.840563	0.7989	Soc_19G0(	1
bomi vs linzhi	GO:0042613	MHC class	1/420	23/15848	0.461085	0.840563	0.7989	Soc_13G0(	1
bomi vs linzhi	GO:0006139	nucleobas	1/420	24/15848	0.475388	0.844626	0.802762	Soc_23G0(	1
bomi vs linzhi	GO:0019882	antigen pr	1/420	24/15848	0.475388	0.844626	0.802762	Soc_13G0(	1
bomi vs linzhi	GO:0003707	steroid ho	2/420	61/15848	0.483446	0.844626	0.802762	Soc_11G0(	2
bomi vs linzhi	GO:0043401	steroid ho	2/420	61/15848	0.483446	0.844626	0.802762	Soc_11G0(	2
bomi vs linzhi	GO:0004722	protein ser	1/420	25/15848	0.489313	0.844626	0.802762	Soc_11G0(	1
bomi vs linzhi	GO:0008565	obsolete p	1/420	25/15848	0.489313	0.844626	0.802762	Soc_23G0(	1
bomi vs linzhi	GO:0016055	Wnt signal	2/420	62/15848	0.492015	0.844626	0.802762	Soc_14G0(	2
bomi vs linzhi	GO:0007268	chemical s	1/420	26/15848	0.502868	0.852682	0.810418	Soc_20G0(	1
bomi vs linzhi	GO:0007264	small GTPa	4/420	139/15848	0.504986	0.852682	0.810418	Soc_12G0(	4
bomi vs linzhi	GO:0015078	proton tra	1/420	27/15848	0.516065	0.857124	0.81464	Soc_6G001	1
bomi vs linzhi	GO:0015991	proton tra	1/420	27/15848	0.516065	0.857124	0.81464	Soc_6G001	1
bomi vs linzhi	GO:0004252	serine-typ	4/420	143/15848	0.527312	0.857124	0.81464	Soc_19G0(	4
bomi vs linzhi	GO:0005216	ion channe	7/420	258/15848	0.528542	0.857124	0.81464	Soc_11G0(	7
bomi vs linzhi	GO:0043547	positive re	1/420	28/15848	0.528912	0.857124	0.81464	Soc_21G0(	1
bomi vs linzhi	GO:0006096	glycolytic	1/420	29/15848	0.541418	0.857124	0.81464	Soc_18G0(	1
bomi vs linzhi	GO:0008233	peptidase	1/420	29/15848	0.541418	0.857124	0.81464	Soc_11G0(	1

bomi vs linzhi	GO:0005578	extracellula	1/420	30/15848	0.553594	0.857124	0.81464	Soc_17G00	1
bomi vs linzhi	GO:0016887	ATPase ac	2/420	71/15848	0.564959	0.857124	0.81464	Soc_15G00	2
bomi vs linzhi	GO:0005245	voltage-ga	1/420	31/15848	0.565447	0.857124	0.81464	Soc_11G00	1
bomi vs linzhi	GO:0006897	endocytos	1/420	31/15848	0.565447	0.857124	0.81464	Soc_11G00	1
bomi vs linzhi	GO:0008009	chemokine	1/420	31/15848	0.565447	0.857124	0.81464	Soc_20G00	1
bomi vs linzhi	GO:0071805	potassium	1/420	31/15848	0.565447	0.857124	0.81464	Soc_14G00	1
bomi vs linzhi	GO:0005179	hormone a	2/420	72/15848	0.572585	0.857124	0.81464	Soc_11G00	2
bomi vs linzhi	GO:0008092	cytoskeleta	1/420	32/15848	0.576986	0.857124	0.81464	Soc_15G00	1
bomi vs linzhi	GO:0042157	lipoprotein	1/420	32/15848	0.576986	0.857124	0.81464	Soc_3G001	1
bomi vs linzhi	GO:0016705	oxidoredu	2/420	74/15848	0.587545	0.857124	0.81464	Soc_12G00	2
bomi vs linzhi	GO:0005886	plasma me	1/420	33/15848	0.588219	0.857124	0.81464	Soc_21G00	1
bomi vs linzhi	GO:0016616	oxidoredu	1/420	33/15848	0.588219	0.857124	0.81464	Soc_20G00	1
bomi vs linzhi	GO:0046983	protein dir	6/420	236/15848	0.598292	0.857124	0.81464	Soc_13G00	6
bomi vs linzhi	GO:0005891	voltage-ga	1/420	34/15848	0.599155	0.857124	0.81464	Soc_11G00	1
bomi vs linzhi	GO:0006511	ubiquitin-(	1/420	34/15848	0.599155	0.857124	0.81464	Soc_14G00	1
bomi vs linzhi	GO:0008305	integrin co	1/420	36/15848	0.620165	0.881062	0.837391	Soc_1G001	1
bomi vs linzhi	GO:0007275	multicellul	2/420	80/15848	0.630077	0.88215	0.838425	Soc_8G000	2
bomi vs linzhi	GO:0007399	nervous sy	1/420	37/15848	0.630254	0.88215	0.838425	Soc_7G000	1
bomi vs linzhi	GO:0006811	ion transpo	7/420	285/15848	0.633778	0.88215	0.838425	Soc_11G00	7
bomi vs linzhi	GO:0006260	DNA replic	1/420	39/15848	0.649637	0.885119	0.841247	Soc_4G000	1
bomi vs linzhi	GO:0046872	metal ion b	5/420	210/15848	0.657386	0.885119	0.841247	Soc_13G00	5
bomi vs linzhi	GO:0003743	translation	1/420	41/15848	0.668007	0.885119	0.841247	Soc_20G00	1
bomi vs linzhi	GO:0030170	pyridoxal p	1/420	41/15848	0.668007	0.885119	0.841247	Soc_22G00	1
bomi vs linzhi	GO:0005840	ribosome	2/420	87/15848	0.675292	0.885119	0.841247	Soc_18G00	2
bomi vs linzhi	GO:0051082	unfolded p	1/420	42/15848	0.676828	0.885119	0.841247	Soc_15G00	1
bomi vs linzhi	GO:0006281	DNA repai	2/420	88/15848	0.681371	0.885119	0.841247	Soc_11G00	2
bomi vs linzhi	GO:0006508	proteolysis	10/420	422/15848	0.685261	0.885119	0.841247	Soc_11G00	10
bomi vs linzhi	GO:0000166	nucleotide	1/420	43/15848	0.685415	0.885119	0.841247	Soc_17G00	1
bomi vs linzhi	GO:0016787	hydrolase	3/420	134/15848	0.693475	0.885119	0.841247	Soc_11G00	3
bomi vs linzhi	GO:0016758	transferase	1/420	44/15848	0.693775	0.885119	0.841247	Soc_7G000	1
bomi vs linzhi	GO:0004713	protein tyr	2/420	91/15848	0.699054	0.885119	0.841247	Soc_16G00	2
bomi vs linzhi	GO:0006412	translation	2/420	91/15848	0.699054	0.885119	0.841247	Soc_18G00	2
bomi vs linzhi	GO:0020037	heme binc	2/420	91/15848	0.699054	0.885119	0.841247	Soc_12G00	2
bomi vs linzhi	GO:0030036	actin cytos	1/420	45/15848	0.701913	0.885119	0.841247	Soc_15G00	1
bomi vs linzhi	GO:0042626	ATPase-co	1/420	46/15848	0.709836	0.885119	0.841247	Soc_17G00	1
bomi vs linzhi	GO:0015074	DNA integ	2/420	94/15848	0.715916	0.885119	0.841247	Soc_1G000	2
bomi vs linzhi	GO:0007165	signal tran	8/420	351/15848	0.716464	0.885119	0.841247	Soc_11G00	8
bomi vs linzhi	GO:0016757	transferase	1/420	47/15848	0.717548	0.885119	0.841247	Soc_15G00	1

bomi vs linzhi	GO:0003735	structural c	2/420	97/15848	0.731978	0.890808	0.846654	Soc_18G00	2
bomi vs linzhi	GO:0004725	protein tyr	1/420	50/15848	0.739479	0.890808	0.846654	Soc_12G00	1
bomi vs linzhi	GO:0009058	biosynthet	1/420	50/15848	0.739479	0.890808	0.846654	Soc_4G001	1
bomi vs linzhi	GO:0005102	signaling r	1/420	52/15848	0.753148	0.890808	0.846654	Soc_8G000	1
bomi vs linzhi	GO:0008234	cysteine-ty	1/420	52/15848	0.753148	0.890808	0.846654	Soc_24G00	1
bomi vs linzhi	GO:0036459	thiol-depe	1/420	52/15848	0.753148	0.890808	0.846654	Soc_11G00	1
bomi vs linzhi	GO:0004984	olfactory r	1/420	53/15848	0.759711	0.890808	0.846654	Soc_16G00	1
bomi vs linzhi	GO:0000287	magnesium	1/420	54/15848	0.766101	0.890808	0.846654	Soc_17G00	1
bomi vs linzhi	GO:0007601	visual perc	1/420	54/15848	0.766101	0.890808	0.846654	Soc_1G002	1
bomi vs linzhi	GO:0004872	signaling r	1/420	55/15848	0.772321	0.890808	0.846654	Soc_21G00	1
bomi vs linzhi	GO:0070588	calcium io	1/420	55/15848	0.772321	0.890808	0.846654	Soc_11G00	1
bomi vs linzhi	GO:0006396	RNA proce	1/420	56/15848	0.778375	0.890808	0.846654	Soc_18G00	1
bomi vs linzhi	GO:0016301	kinase acti	1/420	56/15848	0.778375	0.890808	0.846654	Soc_7G000	1
bomi vs linzhi	GO:0008083	growth fac	2/420	108/15848	0.784408	0.892568	0.848327	Soc_11G00	2
bomi vs linzhi	GO:0004222	metalloend	2/420	109/15848	0.788698	0.892568	0.848327	Soc_19G00	2
bomi vs linzhi	GO:0004674	protein ser	2/420	110/15848	0.792912	0.892568	0.848327	Soc_6G000	2
bomi vs linzhi	GO:0005506	iron ion bi	2/420	114/15848	0.809038	0.905771	0.860876	Soc_12G00	2
bomi vs linzhi	GO:0016579	protein de	1/420	63/15848	0.816492	0.909175	0.864111	Soc_11G00	1
bomi vs linzhi	GO:0005044	scavenger	1/420	66/15848	0.830755	0.910187	0.865073	Soc_24G00	1
bomi vs linzhi	GO:0007018	microtubu	1/420	66/15848	0.830755	0.910187	0.865073	Soc_23G00	1
bomi vs linzhi	GO:0003777	microtubu	1/420	67/15848	0.835259	0.910187	0.865073	Soc_23G00	1
bomi vs linzhi	GO:0051015	actin filam	1/420	67/15848	0.835259	0.910187	0.865073	Soc_16G00	1
bomi vs linzhi	GO:0008237	metallopep	1/420	69/15848	0.843911	0.910187	0.865073	Soc_19G00	1
bomi vs linzhi	GO:0051260	protein ho	1/420	69/15848	0.843911	0.910187	0.865073	Soc_17G00	1
bomi vs linzhi	GO:0006955	immune re	2/420	127/15848	0.853982	0.914876	0.86953	Soc_13G00	2
bomi vs linzhi	GO:0016491	oxidoredu	3/420	179/15848	0.857142	0.914876	0.86953	Soc_21G00	3
bomi vs linzhi	GO:0007156	homophili	2/420	131/15848	0.865739	0.91929	0.873724	Soc_10G00	2
bomi vs linzhi	GO:0005887	integral co	2/420	141/15848	0.891432	0.935878	0.889491	Soc_14G00	2
bomi vs linzhi	GO:0005615	extracellula	1/420	83/15848	0.893028	0.935878	0.889491	Soc_9G000	1
bomi vs linzhi	GO:0005089	Rho guany	1/420	84/15848	0.895878	0.935878	0.889491	Soc_9G000	1
bomi vs linzhi	GO:0005096	GTPase ac	1/420	88/15848	0.906539	0.935878	0.889491	Soc_1G002	1
bomi vs linzhi	GO:0035023	regulation	1/420	88/15848	0.906539	0.935878	0.889491	Soc_9G000	1
bomi vs linzhi	GO:0007155	cell adhesi	2/420	149/15848	0.90862	0.935878	0.889491	Soc_15G00	2
bomi vs linzhi	GO:0055114	oxidation-	7/420	413/15848	0.924557	0.947556	0.900589	Soc_12G00	7
bomi vs linzhi	GO:0003779	actin bindi	1/420	105/15848	0.940966	0.955095	0.907755	Soc_15G00	1
bomi vs linzhi	GO:0008152	metabolic	3/420	226/15848	0.941185	0.955095	0.907755	Soc_13G00	3
bomi vs linzhi	GO:0006886	intracellula	1/420	115/15848	0.954957	0.964319	0.916522	Soc_1G001	1
bomi vs linzhi	GO:0003924	GTPase ac	4/420	314/15848	0.968784	0.97351	0.925257	Soc_12G00	4

bomi vs linzhi	GO:0035556	intracellula	1/420	170/15848	0.989857	0.989857	0.940794	Soc_8G000	1
bomi vs zangga	GO:0008417	fucosyltran	4/424	13/15848	0.000298	0.0339	0.033371	Soc_13G00	4
bomi vs zangga	GO:0000786	nucleosom	12/424	137/15848	0.000314	0.0339	0.033371	Soc_20G00	12
bomi vs zangga	GO:0005200	structural	4/424	27/15848	0.005443	0.391893	0.385782	Soc_15G00	4
bomi vs zangga	GO:0045211	postsynap	4/424	31/15848	0.008974	0.43389	0.427124	Soc_18G00	4
bomi vs zangga	GO:0007017	microtubu	4/424	32/15848	0.010044	0.43389	0.427124	Soc_15G00	4
bomi vs zangga	GO:0008137	NADH deh	2/424	10/15848	0.027877	0.624073	0.614341	Soc_13G00	2
bomi vs zangga	GO:0030971	receptor ty	2/424	10/15848	0.027877	0.624073	0.614341	Soc_15G00	2
bomi vs zangga	GO:0006486	protein gly	6/424	87/15848	0.029095	0.624073	0.614341	Soc_10G00	6
bomi vs zangga	GO:0005874	microtubu	4/424	45/15848	0.031775	0.624073	0.614341	Soc_15G00	4
bomi vs zangga	GO:0006334	nucleosom	4/424	48/15848	0.039012	0.624073	0.614341	Soc_3G000	4
bomi vs zangga	GO:1902476	chloride tr	2/424	12/15848	0.039475	0.624073	0.614341	Soc_24G00	2
bomi vs zangga	GO:0005230	extracellula	5/424	71/15848	0.041363	0.624073	0.614341	Soc_13G00	5
bomi vs zangga	GO:0016705	oxidoredu	5/424	74/15848	0.048018	0.624073	0.614341	Soc_12G00	5
bomi vs zangga	GO:0005245	voltage-ga	3/424	31/15848	0.049168	0.624073	0.614341	Soc_11G00	3
bomi vs zangga	GO:0048015	phosphatid	2/424	14/15848	0.052557	0.624073	0.614341	Soc_1G000	2
bomi vs zangga	GO:0055114	oxidation-	17/424	413/15848	0.052865	0.624073	0.614341	Soc_10G00	17
bomi vs zangga	GO:0042981	regulation	5/424	77/15848	0.055271	0.624073	0.614341	Soc_1G000	5
bomi vs zangga	GO:0006935	chemotaxi	3/424	33/15848	0.057417	0.624073	0.614341	Soc_14G00	3
bomi vs zangga	GO:0070588	calcium io	4/424	55/15848	0.059254	0.624073	0.614341	Soc_11G00	4
bomi vs zangga	GO:0005891	voltage-ga	3/424	34/15848	0.061771	0.624073	0.614341	Soc_11G00	3
bomi vs zangga	GO:0007596	blood coa	2/424	16/15848	0.066935	0.624073	0.614341	Soc_24G00	2
bomi vs zangga	GO:0016192	vesicle-me	5/424	82/15848	0.068686	0.624073	0.614341	Soc_12G00	5
bomi vs zangga	GO:0034220	ion transm	5/424	82/15848	0.068686	0.624073	0.614341	Soc_13G00	5
bomi vs zangga	GO:0005615	extracellula	5/424	83/15848	0.071567	0.624073	0.614341	Soc_12G00	5
bomi vs zangga	GO:0005215	transporte	3/424	37/15848	0.075715	0.624073	0.614341	Soc_3G001	3
bomi vs zangga	GO:0046854	phosphatid	2/424	18/15848	0.082436	0.624073	0.614341	Soc_1G000	2
bomi vs zangga	GO:0016055	Wnt signa	4/424	62/15848	0.084082	0.624073	0.614341	Soc_18G00	4
bomi vs zangga	GO:0005506	iron ion bi	6/424	114/15848	0.085542	0.624073	0.614341	Soc_12G00	6
bomi vs zangga	GO:0006260	DNA replic	3/424	39/15848	0.085711	0.624073	0.614341	Soc_13G00	3
bomi vs zangga	GO:0035556	intracellula	8/424	170/15848	0.086677	0.624073	0.614341	Soc_13G00	8
bomi vs zangga	GO:0020037	heme bind	5/424	91/15848	0.096939	0.667598	0.657187	Soc_12G00	5
bomi vs zangga	GO:0006352	DNA-temp	2/424	20/15848	0.098903	0.667598	0.657187	Soc_18G00	2
bomi vs zangga	GO:0006816	calcium io	3/424	43/15848	0.107251	0.702009	0.691061	Soc_8G000	3
bomi vs zangga	GO:0042613	MHC class	2/424	23/15848	0.125101	0.73689	0.725399	Soc_13G00	2
bomi vs zangga	GO:0019882	antigen pr	2/424	24/15848	0.134168	0.73689	0.725399	Soc_13G00	2
bomi vs zangga	GO:0004725	protein tyr	3/424	50/15848	0.149259	0.73689	0.725399	Soc_15G00	3
bomi vs zangga	GO:0004888	transmeml	7/424	165/15848	0.154398	0.73689	0.725399	Soc_13G00	7

bomi vs zangga	GO:0016791	phosphata	5/424	106/15848	0.15481	0.73689	0.725399	Soc_11G0(	5
bomi vs zangga	GO:0008168	methyltran	3/424	51/15848	0.155643	0.73689	0.725399	Soc_15G0(	3
bomi vs zangga	GO:0008234	cysteine-ty	3/424	52/15848	0.16211	0.73689	0.725399	Soc_23G0(	3
bomi vs zangga	GO:0036459	thiol-depe	3/424	52/15848	0.16211	0.73689	0.725399	Soc_18G0(	3
bomi vs zangga	GO:0009055	electron tr	2/424	27/15848	0.162163	0.73689	0.725399	Soc_13G0(	2
bomi vs zangga	GO:0004222	metalloend	5/424	109/15848	0.167807	0.73689	0.725399	Soc_13G0(	5
bomi vs zangga	GO:0007601	visual perc	3/424	54/15848	0.17528	0.73689	0.725399	Soc_2G000	3
bomi vs zangga	GO:0009190	cyclic nucl	2/424	30/15848	0.191078	0.73689	0.725399	Soc_15G0(	2
bomi vs zangga	GO:0016849	phosphoru	2/424	30/15848	0.191078	0.73689	0.725399	Soc_15G0(	2
bomi vs zangga	GO:0016702	oxidoredu	2/424	31/15848	0.200864	0.73689	0.725399	Soc_13G0(	2
bomi vs zangga	GO:0071805	potassium	2/424	31/15848	0.200864	0.73689	0.725399	Soc_16G0(	2
bomi vs zangga	GO:0008289	lipid bindi	4/424	88/15848	0.209569	0.73689	0.725399	Soc_1G000	4
bomi vs zangga	GO:0005267	potassium	2/424	32/15848	0.210708	0.73689	0.725399	Soc_16G0(	2
bomi vs zangga	GO:0042157	lipoprotein	2/424	32/15848	0.210708	0.73689	0.725399	Soc_1G000	2
bomi vs zangga	GO:0003707	steroid ho	3/424	61/15848	0.223416	0.73689	0.725399	Soc_13G0(	3
bomi vs zangga	GO:0043401	steroid ho	3/424	61/15848	0.223416	0.73689	0.725399	Soc_13G0(	3
bomi vs zangga	GO:0005667	transcriptio	2/424	34/15848	0.230524	0.73689	0.725399	Soc_21G0(	2
bomi vs zangga	GO:0006470	protein de	5/424	123/15848	0.233426	0.73689	0.725399	Soc_11G0(	5
bomi vs zangga	GO:0004383	guanylate	1/424	10/15848	0.237585	0.73689	0.725399	Soc_3G000	1
bomi vs zangga	GO:0004571	mannosyl-	1/424	10/15848	0.237585	0.73689	0.725399	Soc_6G001	1
bomi vs zangga	GO:0005764	lysosome	1/424	10/15848	0.237585	0.73689	0.725399	Soc_18G0(	1
bomi vs zangga	GO:0006182	cGMP bios	1/424	10/15848	0.237585	0.73689	0.725399	Soc_3G000	1
bomi vs zangga	GO:0006694	steroid bic	1/424	10/15848	0.237585	0.73689	0.725399	Soc_1G000	1
bomi vs zangga	GO:0016712	oxidoredu	1/424	10/15848	0.237585	0.73689	0.725399	Soc_20G0(	1
bomi vs zangga	GO:0016579	protein de	3/424	63/15848	0.237626	0.73689	0.725399	Soc_18G0(	3
bomi vs zangga	GO:0008373	sialyltransf	2/424	36/15848	0.250452	0.73689	0.725399	Soc_10G0(	2
bomi vs zangga	GO:0008017	microtubu	4/424	96/15848	0.255544	0.73689	0.725399	Soc_3G000	4
bomi vs zangga	GO:0005247	voltage-ga	1/424	11/15848	0.257995	0.73689	0.725399	Soc_16G0(	1
bomi vs zangga	GO:0007224	smoothen	1/424	11/15848	0.257995	0.73689	0.725399	Soc_22G0(	1
bomi vs zangga	GO:0044212	transcriptio	1/424	11/15848	0.257995	0.73689	0.725399	Soc_11G0(	1
bomi vs zangga	GO:0008146	sulfotransf	3/424	66/15848	0.259214	0.73689	0.725399	Soc_13G0(	3
bomi vs zangga	GO:0016311	dephosph	4/424	98/15848	0.267353	0.73689	0.725399	Soc_11G0(	4
bomi vs zangga	GO:0004707	MAP kinas	1/424	12/15848	0.277861	0.73689	0.725399	Soc_18G0(	1
bomi vs zangga	GO:0007179	transformi	1/424	12/15848	0.277861	0.73689	0.725399	Soc_21G0(	1
bomi vs zangga	GO:0009116	nucleoside	1/424	12/15848	0.277861	0.73689	0.725399	Soc_6G000	1
bomi vs zangga	GO:0030117	membrane	1/424	12/15848	0.277861	0.73689	0.725399	Soc_24G0(	1
bomi vs zangga	GO:0030833	regulation	1/424	12/15848	0.277861	0.73689	0.725399	Soc_7G000	1
bomi vs zangga	GO:0008237	metallope	3/424	69/15848	0.281045	0.73689	0.725399	Soc_13G0(	3

bomi vs zangga	GO:0016459	myosin co	3/424	70/15848	0.288361	0.73689	0.725399	Soc_10G0(	3
bomi vs zangga	GO:0007049	cell cycle	2/424	40/15848	0.290384	0.73689	0.725399	Soc_10G0(	2
bomi vs zangga	GO:0003774	motor acti	3/424	71/15848	0.295691	0.73689	0.725399	Soc_10G0(	3
bomi vs zangga	GO:0003723	RNA bindi	7/424	202/15848	0.297096	0.73689	0.725399	Soc_10G0(	7
bomi vs zangga	GO:0004620	phospholi	1/424	13/15848	0.297196	0.73689	0.725399	Soc_5G000	1
bomi vs zangga	GO:0006950	response t	1/424	13/15848	0.297196	0.73689	0.725399	Soc_24G0(	1
bomi vs zangga	GO:0008271	secondary	1/424	13/15848	0.297196	0.73689	0.725399	Soc_15G0(	1
bomi vs zangga	GO:0008272	sulfate trar	1/424	13/15848	0.297196	0.73689	0.725399	Soc_15G0(	1
bomi vs zangga	GO:0009395	phospholi	1/424	13/15848	0.297196	0.73689	0.725399	Soc_5G000	1
bomi vs zangga	GO:0015116	sulfate trar	1/424	13/15848	0.297196	0.73689	0.725399	Soc_15G0(	1
bomi vs zangga	GO:0042813	Wnt-activa	1/424	13/15848	0.297196	0.73689	0.725399	Soc_4G000	1
bomi vs zangga	GO:0046983	protein dir	8/424	236/15848	0.298122	0.73689	0.725399	Soc_11G0(	8
bomi vs zangga	GO:0000981	DNA-bind	1/424	14/15848	0.316014	0.73689	0.725399	Soc_22G0(	1
bomi vs zangga	GO:0005158	insulin rec	1/424	14/15848	0.316014	0.73689	0.725399	Soc_3G000	1
bomi vs zangga	GO:0005730	nucleolus	1/424	14/15848	0.316014	0.73689	0.725399	Soc_5G000	1
bomi vs zangga	GO:0046872	metal ion	7/424	210/15848	0.331377	0.73689	0.725399	Soc_13G0(	7
bomi vs zangga	GO:0003924	GTPase ac	10/424	314/15848	0.332294	0.73689	0.725399	Soc_10G0(	10
bomi vs zangga	GO:0001614	purinergic	1/424	15/15848	0.33433	0.73689	0.725399	Soc_3G000	1
bomi vs zangga	GO:0003729	mRNA bin	1/424	15/15848	0.33433	0.73689	0.725399	Soc_10G0(	1
bomi vs zangga	GO:0004931	extracellul	1/424	15/15848	0.33433	0.73689	0.725399	Soc_3G000	1
bomi vs zangga	GO:0006289	nucleotide	1/424	15/15848	0.33433	0.73689	0.725399	Soc_20G0(	1
bomi vs zangga	GO:0007411	axon guid	1/424	15/15848	0.33433	0.73689	0.725399	Soc_22G0(	1
bomi vs zangga	GO:0033198	response t	1/424	15/15848	0.33433	0.73689	0.725399	Soc_3G000	1
bomi vs zangga	GO:0016491	oxidoredu	6/424	179/15848	0.346315	0.742414	0.730837	Soc_10G0(	6
bomi vs zangga	GO:0004867	serine-typ	2/424	46/15848	0.349658	0.742414	0.730837	Soc_24G0(	2
bomi vs zangga	GO:0005885	Arp2/3 pro	1/424	16/15848	0.352156	0.742414	0.730837	Soc_7G000	1
bomi vs zangga	GO:0008324	cation trar	1/424	16/15848	0.352156	0.742414	0.730837	Soc_9G000	1
bomi vs zangga	GO:0006811	ion transp	9/424	285/15848	0.354022	0.742414	0.730837	Soc_11G0(	9
bomi vs zangga	GO:0006869	lipid trans	2/424	47/15848	0.359391	0.742868	0.731283	Soc_1G000	2
bomi vs zangga	GO:0006520	cellular am	1/424	17/15848	0.369506	0.742868	0.731283	Soc_17G0(	1
bomi vs zangga	GO:0034314	Arp2/3 co	1/424	17/15848	0.369506	0.742868	0.731283	Soc_7G000	1
bomi vs zangga	GO:0042254	ribosome	1/424	17/15848	0.369506	0.742868	0.731283	Soc_5G000	1
bomi vs zangga	GO:0003684	damaged	1/424	18/15848	0.386393	0.742868	0.731283	Soc_20G0(	1
bomi vs zangga	GO:0005089	Rho guany	3/424	84/15848	0.390998	0.742868	0.731283	Soc_15G0(	3
bomi vs zangga	GO:0003333	amino acid	1/424	19/15848	0.402828	0.742868	0.731283	Soc_13G0(	1
bomi vs zangga	GO:0005201	extracellul	1/424	19/15848	0.402828	0.742868	0.731283	Soc_2G000	1
bomi vs zangga	GO:0015171	amino acid	1/424	19/15848	0.402828	0.742868	0.731283	Soc_13G0(	1
bomi vs zangga	GO:0043065	positive re	1/424	19/15848	0.402828	0.742868	0.731283	Soc_5G000	1

bomi vs zangga	GO:0098655	cation tran	1/424	19/15848	0.402828	0.742868	0.731283	Soc_3G000	1
bomi vs zangga	GO:0035091	phosphati	2/424	53/15848	0.416526	0.742868	0.731283	Soc_11G00	2
bomi vs zangga	GO:0003887	DNA-direc	1/424	20/15848	0.418824	0.742868	0.731283	Soc_13G00	1
bomi vs zangga	GO:0005104	fibroblast	1/424	20/15848	0.418824	0.742868	0.731283	Soc_11G00	1
bomi vs zangga	GO:0098609	cell-cell ad	1/424	20/15848	0.418824	0.742868	0.731283	Soc_11G00	1
bomi vs zangga	GO:0035023	regulation	3/424	88/15848	0.419827	0.742868	0.731283	Soc_15G00	3
bomi vs zangga	GO:0005540	hyaluronic	1/424	21/15848	0.434392	0.742868	0.731283	Soc_2G000	1
bomi vs zangga	GO:0006338	chromatin	1/424	21/15848	0.434392	0.742868	0.731283	Soc_8G000	1
bomi vs zangga	GO:0015629	actin cytos	1/424	21/15848	0.434392	0.742868	0.731283	Soc_7G000	1
bomi vs zangga	GO:0043087	regulation	1/424	21/15848	0.434392	0.742868	0.731283	Soc_5G000	1
bomi vs zangga	GO:0006955	immune re	4/424	127/15848	0.442663	0.742868	0.731283	Soc_13G00	4
bomi vs zangga	GO:0006396	RNA proce	2/424	56/15848	0.444127	0.742868	0.731283	Soc_13G00	2
bomi vs zangga	GO:0004866	endopepti	1/424	22/15848	0.449545	0.742868	0.731283	Soc_12G00	1
bomi vs zangga	GO:0005739	mitochond	2/424	57/15848	0.453166	0.742868	0.731283	Soc_4G000	2
bomi vs zangga	GO:0008138	protein tyr	2/424	58/15848	0.46212	0.742868	0.731283	Soc_11G00	2
bomi vs zangga	GO:0004129	cytochrom	1/424	23/15848	0.464292	0.742868	0.731283	Soc_8G000	1
bomi vs zangga	GO:0004298	threonine-	1/424	23/15848	0.464292	0.742868	0.731283	Soc_8G000	1
bomi vs zangga	GO:0004890	GABA-A re	1/424	23/15848	0.464292	0.742868	0.731283	Soc_13G00	1
bomi vs zangga	GO:0005839	proteasom	1/424	23/15848	0.464292	0.742868	0.731283	Soc_8G000	1
bomi vs zangga	GO:0008543	fibroblast	1/424	23/15848	0.464292	0.742868	0.731283	Soc_11G00	1
bomi vs zangga	GO:0030001	metal ion	1/424	23/15848	0.464292	0.742868	0.731283	Soc_3G001	1
bomi vs zangga	GO:0051603	proteolysis	1/424	23/15848	0.464292	0.742868	0.731283	Soc_8G000	1
bomi vs zangga	GO:0006139	nucleobas	1/424	24/15848	0.478645	0.760035	0.748182	Soc_6G000	1
bomi vs zangga	GO:0016787	hydrolase	4/424	134/15848	0.483806	0.760035	0.748182	Soc_10G00	4
bomi vs zangga	GO:0006821	chloride tr	1/424	25/15848	0.492615	0.760035	0.748182	Soc_16G00	1
bomi vs zangga	GO:0007010	cytoskelet	1/424	25/15848	0.492615	0.760035	0.748182	Soc_23G00	1
bomi vs zangga	GO:0022848	acetylcholi	1/424	25/15848	0.492615	0.760035	0.748182	Soc_4G000	1
bomi vs zangga	GO:0004197	cysteine-ty	1/424	26/15848	0.506211	0.760428	0.74857	Soc_23G00	1
bomi vs zangga	GO:0007160	cell-matrix	1/424	26/15848	0.506211	0.760428	0.74857	Soc_24G00	1
bomi vs zangga	GO:0007268	chemical s	1/424	26/15848	0.506211	0.760428	0.74857	Soc_21G00	1
bomi vs zangga	GO:0003700	DNA-bind	13/424	476/15848	0.509827	0.760428	0.74857	Soc_11G00	13
bomi vs zangga	GO:0005328	neurotrans	1/424	27/15848	0.519444	0.760428	0.74857	Soc_22G00	1
bomi vs zangga	GO:0005044	scavenger	2/424	66/15848	0.530512	0.760428	0.74857	Soc_24G00	2
bomi vs zangga	GO:0003950	NAD+ AD	1/424	28/15848	0.532323	0.760428	0.74857	Soc_7G000	1
bomi vs zangga	GO:0008285	negative re	1/424	28/15848	0.532323	0.760428	0.74857	Soc_5G000	1
bomi vs zangga	GO:0043547	positive re	1/424	28/15848	0.532323	0.760428	0.74857	Soc_18G00	1
bomi vs zangga	GO:0004842	ubiquitin-	3/424	105/15848	0.535971	0.760428	0.74857	Soc_1G000	3
bomi vs zangga	GO:0005216	ion channe	7/424	258/15848	0.538625	0.760428	0.74857	Soc_11G00	7

bomi vs zangga	GO:0003777	microtubu	2/424	67/15848	0.538637	0.760428	0.74857	Soc_3G001		2
bomi vs zangga	GO:0051015	actin filam	2/424	67/15848	0.538637	0.760428	0.74857	Soc_23G00		2
bomi vs zangga	GO:0000226	microtubu	1/424	30/15848	0.557056	0.768055	0.756077	Soc_22G00		1
bomi vs zangga	GO:0005578	extracellul	1/424	30/15848	0.557056	0.768055	0.756077	Soc_10G00		1
bomi vs zangga	GO:0006464	cellular pr	1/424	30/15848	0.557056	0.768055	0.756077	Soc_5G000		1
bomi vs zangga	GO:0008152	metabolic	6/424	226/15848	0.565199	0.768055	0.756077	Soc_10G00		6
bomi vs zangga	GO:0008009	chemokine	1/424	31/15848	0.568929	0.768055	0.756077	Soc_4G000		1
bomi vs zangga	GO:0016746	transferase	1/424	31/15848	0.568929	0.768055	0.756077	Soc_18G00		1
bomi vs zangga	GO:0051726	regulation	1/424	31/15848	0.568929	0.768055	0.756077	Soc_15G00		1
bomi vs zangga	GO:0006457	protein fol	1/424	32/15848	0.580485	0.776435	0.764327	Soc_1G002		1
bomi vs zangga	GO:0043565	sequence-	5/424	191/15848	0.582326	0.776435	0.764327	Soc_13G00		5
bomi vs zangga	GO:0005886	plasma me	1/424	33/15848	0.591731	0.779354	0.7672	Soc_15G00		1
bomi vs zangga	GO:0016616	oxidoredu	1/424	33/15848	0.591731	0.779354	0.7672	Soc_1G000		1
bomi vs zangga	GO:0006887	exocytosis	1/424	34/15848	0.602677	0.788959	0.776656	Soc_18G00		1
bomi vs zangga	GO:0006836	neurotrans	1/424	36/15848	0.623698	0.806699	0.794119	Soc_22G00		1
bomi vs zangga	GO:0015031	protein tra	1/424	36/15848	0.623698	0.806699	0.794119	Soc_24G00		1
bomi vs zangga	GO:0005783	endoplasm	1/424	39/15848	0.653167	0.835914	0.822879	Soc_13G00		1
bomi vs zangga	GO:0006813	potassium	3/424	125/15848	0.654026	0.835914	0.822879	Soc_11G00		3
bomi vs zangga	GO:0006812	cation tran	1/424	40/15848	0.662469	0.837672	0.824609	Soc_9G000		1
bomi vs zangga	GO:0030170	pyridoxal p	1/424	41/15848	0.671522	0.837672	0.824609	Soc_12G00		1
bomi vs zangga	GO:0007166	cell surface	2/424	86/15848	0.674213	0.837672	0.824609	Soc_13G00		2
bomi vs zangga	GO:0051082	unfolded p	1/424	42/15848	0.680333	0.837672	0.824609	Soc_1G002		1
bomi vs zangga	GO:0005882	intermedia	2/424	87/15848	0.680367	0.837672	0.824609	Soc_18G00		2
bomi vs zangga	GO:0005096	GTPase ac	2/424	88/15848	0.686426	0.837672	0.824609	Soc_18G00		2
bomi vs zangga	GO:0006281	DNA repai	2/424	88/15848	0.686426	0.837672	0.824609	Soc_8G000		2
bomi vs zangga	GO:0022857	transmemb	2/424	88/15848	0.686426	0.837672	0.824609	Soc_18G00		2
bomi vs zangga	GO:0016758	transferase	1/424	44/15848	0.697254	0.846106	0.832911	Soc_23G00		1
bomi vs zangga	GO:0015074	DNA integ	2/424	94/15848	0.720822	0.869819	0.856255	Soc_2G000		2
bomi vs zangga	GO:0005576	extracellul	7/424	311/15848	0.730296	0.873332	0.859713	Soc_11G00		7
bomi vs zangga	GO:0005887	integral co	3/424	141/15848	0.73182	0.873332	0.859713	Soc_22G00		3
bomi vs zangga	GO:0009058	biosynthet	1/424	50/15848	0.74284	0.881613	0.867864	Soc_12G00		1
bomi vs zangga	GO:0005622	intracellula	9/424	400/15848	0.747274	0.882028	0.868273	Soc_11G00		9
bomi vs zangga	GO:0005102	signaling r	1/424	52/15848	0.756459	0.888017	0.874169	Soc_8G000		1
bomi vs zangga	GO:0006351	transcripti	1/424	53/15848	0.762996	0.888671	0.874812	Soc_15G00		1
bomi vs zangga	GO:0005737	cytoplasm	5/424	238/15848	0.766995	0.888671	0.874812	Soc_15G00		5
bomi vs zangga	GO:0045454	cell redox	1/424	54/15848	0.769359	0.888671	0.874812	Soc_20G00		1
bomi vs zangga	GO:0004872	signaling r	1/424	55/15848	0.77555	0.890704	0.876814	Soc_18G00		1
bomi vs zangga	GO:0016301	kinase acti	1/424	56/15848	0.781576	0.890704	0.876814	Soc_1G000		1

bomi vs zangga	GO:0005975	carbohydr	2/424	107/15848	0.784519	0.890704	0.876814	Soc_8G00(	2
bomi vs zangga	GO:0008083	growth fac	2/424	108/15848	0.788848	0.890704	0.876814	Soc_11G0(	2
bomi vs zangga	GO:0005085	guanyl-nu	1/424	58/15848	0.793148	0.890704	0.876814	Soc_18G0(	1
bomi vs zangga	GO:0004674	protein se	2/424	110/15848	0.797277	0.890704	0.876814	Soc_12G0(	2
bomi vs zangga	GO:0006508	proteolysis	9/424	422/15848	0.80003	0.890704	0.876814	Soc_13G0(	9
bomi vs zangga	GO:0006915	apoptotic	1/424	60/15848	0.804108	0.890704	0.876814	Soc_8G00(	1
bomi vs zangga	GO:0008076	voltage-ga	1/424	61/15848	0.809369	0.891958	0.878048	Soc_11G0(	1
bomi vs zangga	GO:0006886	intracellula	2/424	115/15848	0.817059	0.895862	0.881891	Soc_1G001	2
bomi vs zangga	GO:0016567	protein ub	1/424	64/15848	0.824321	0.899259	0.885235	Soc_4G00(	1
bomi vs zangga	GO:0007018	microtubu	1/424	66/15848	0.833633	0.904847	0.890737	Soc_7G00(	1
bomi vs zangga	GO:0051260	protein ho	1/424	69/15848	0.846685	0.913948	0.899695	Soc_11G0(	1
bomi vs zangga	GO:0016887	ATPase ac	1/424	71/15848	0.854814	0.913948	0.899695	Soc_1G00(	1
bomi vs zangga	GO:0005179	hormone a	1/424	72/15848	0.858716	0.913948	0.899695	Soc_24G0(	1
bomi vs zangga	GO:0003824	catalytic a	3/424	178/15848	0.858942	0.913948	0.899695	Soc_24G0(	3
bomi vs zangga	GO:0005198	structural	3/424	181/15848	0.866471	0.91744	0.903133	Soc_18G0(	3
bomi vs zangga	GO:0005249	voltage-ga	1/424	79/15848	0.883251	0.92856	0.914079	Soc_11G0(	1
bomi vs zangga	GO:0007275	multicellul	1/424	80/15848	0.88639	0.92856	0.914079	Soc_8G00(	1
bomi vs zangga	GO:0007264	small GTPa	2/424	139/15848	0.88987	0.92856	0.914079	Soc_18G0(	2
bomi vs zangga	GO:0005840	ribosome	1/424	87/15848	0.906129	0.94098	0.926305	Soc_24G0(	1
bomi vs zangga	GO:0007165	signal tran	6/424	351/15848	0.911502	0.941618	0.926933	Soc_11G0(	6
bomi vs zangga	GO:0006412	translation	1/424	91/15848	0.91583	0.941618	0.926933	Soc_24G0(	1
bomi vs zangga	GO:0005856	cytoskeleto	1/424	93/15848	0.920299	0.941618	0.926933	Soc_11G0(	1
bomi vs zangga	GO:0003735	structural	1/424	97/15848	0.92854	0.941618	0.926933	Soc_24G0(	1
bomi vs zangga	GO:0006357	regulation	1/424	97/15848	0.92854	0.941618	0.926933	Soc_21G0(	1
bomi vs zangga	GO:0003779	actin bindi	1/424	105/15848	0.942557	0.951366	0.93653	Soc_23G0(	1
bomi vs zangga	GO:0004252	serine-typ	1/424	143/15848	0.97967	0.98275	0.967424	Soc_2G00(	1
bomi vs zangga	GO:0007155	cell adhesi	1/424	149/15848	0.98275	0.98275	0.967424	Soc_4G00(	1
milin vs linzhi	GO:0005923	bicellular t	11/496	74/15848	1.78E-05	0.003909	0.003666	Soc_13G0(	11
milin vs linzhi	GO:0004415	hyalurono	4/496	10/15848	0.000171	0.018843	0.017671	Soc_8G00(	4
milin vs linzhi	GO:0004298	threonine-	5/496	23/15848	0.00062	0.027095	0.02541	Soc_2G001	5
milin vs linzhi	GO:0005839	proteasom	5/496	23/15848	0.00062	0.027095	0.02541	Soc_2G001	5
milin vs linzhi	GO:0051603	proteolysis	5/496	23/15848	0.00062	0.027095	0.02541	Soc_2G001	5
milin vs linzhi	GO:0004175	endopepti	4/496	14/15848	0.000739	0.027095	0.02541	Soc_2G001	4
milin vs linzhi	GO:0005272	sodium ch	4/496	15/15848	0.000983	0.030891	0.02897	Soc_22G0(	4
milin vs linzhi	GO:0003684	damaged	4/496	18/15848	0.002045	0.056243	0.052745	Soc_5G00(	4
milin vs linzhi	GO:0008009	chemokine	5/496	31/15848	0.00255	0.062336	0.058458	Soc_11G0(	5
milin vs linzhi	GO:0016757	transferase	6/496	47/15848	0.003297	0.072531	0.06802	Soc_11G0(	6
milin vs linzhi	GO:0008152	metabolic	15/496	226/15848	0.005116	0.10233	0.095965	Soc_11G0(	15

milin vs linzhi	GO:0009966	regulation	4/496	26/15848	0.008202	0.150363	0.14101	Soc_11G0(	4
milin vs linzhi	GO:0005198	structural r	12/496	181/15848	0.011689	0.177549	0.166505	Soc_13G0(	12
milin vs linzhi	GO:0016758	transferase	5/496	44/15848	0.011699	0.177549	0.166505	Soc_21G0(	5
milin vs linzhi	GO:0008233	peptidase	4/496	29/15848	0.012106	0.177549	0.166505	Soc_10G0(	4
milin vs linzhi	GO:0005975	carbohydr	8/496	107/15848	0.019083	0.25378	0.237994	Soc_15G0(	8
milin vs linzhi	GO:0004725	protein tyr	5/496	50/15848	0.01961	0.25378	0.237994	Soc_15G0(	5
milin vs linzhi	GO:0004713	protein tyr	7/496	91/15848	0.023927	0.292443	0.274253	Soc_11G0(	7
milin vs linzhi	GO:0006814	sodium ior	5/496	54/15848	0.026467	0.306459	0.287397	Soc_22G0(	5
milin vs linzhi	GO:0004890	GABA-A re	3/496	23/15848	0.033903	0.371468	0.348362	Soc_10G0(	3
milin vs linzhi	GO:0016779	nucleotidy	2/496	10/15848	0.037247	0.371468	0.348362	Soc_16G0(	2
milin vs linzhi	GO:0030971	receptor ty	2/496	10/15848	0.037247	0.371468	0.348362	Soc_15G0(	2
milin vs linzhi	GO:0003924	GTPase ac	16/496	314/15848	0.038835	0.371468	0.348362	Soc_10G0(	16
milin vs linzhi	GO:0030414	peptidase	2/496	11/15848	0.044598	0.408811	0.383382	Soc_15G0(	2
milin vs linzhi	GO:0009607	response t	3/496	27/15848	0.05112	0.449855	0.421873	Soc_12G0(	3
milin vs linzhi	GO:0006281	DNA repai	6/496	88/15848	0.057666	0.479515	0.449689	Soc_13G0(	6
milin vs linzhi	GO:0032947	molecular	2/496	13/15848	0.060711	0.479515	0.449689	Soc_13G0(	2
milin vs linzhi	GO:0006397	mRNA pro	3/496	29/15848	0.061032	0.479515	0.449689	Soc_3G000	3
milin vs linzhi	GO:0016459	myosin co	5/496	70/15848	0.067981	0.479515	0.449689	Soc_22G0(	5
milin vs linzhi	GO:0060271	cilium asse	2/496	14/15848	0.0694	0.479515	0.449689	Soc_4G000	2
milin vs linzhi	GO:0003774	motor acti	5/496	71/15848	0.071346	0.479515	0.449689	Soc_22G0(	5
milin vs linzhi	GO:0071805	potassium	3/496	31/15848	0.071766	0.479515	0.449689	Soc_14G0(	3
milin vs linzhi	GO:0008168	methyltran	4/496	51/15848	0.074885	0.479515	0.449689	Soc_15G0(	4
milin vs linzhi	GO:0005267	potassium	3/496	32/15848	0.077428	0.479515	0.449689	Soc_14G0(	3
milin vs linzhi	GO:0008092	cytoskeleta	3/496	32/15848	0.077428	0.479515	0.449689	Soc_14G0(	3
milin vs linzhi	GO:0005813	centrosom	2/496	15/15848	0.078466	0.479515	0.449689	Soc_4G000	2
milin vs linzhi	GO:0006470	protein de	7/496	123/15848	0.091486	0.543974	0.510138	Soc_11G0(	7
milin vs linzhi	GO:0006955	immune re	7/496	127/15848	0.103888	0.601455	0.564044	Soc_11G0(	7
milin vs linzhi	GO:0005085	guanyl-nu	4/496	58/15848	0.10772	0.607654	0.569857	Soc_10G0(	4
milin vs linzhi	GO:0005576	extracellula	14/496	311/15848	0.111226	0.611746	0.573695	Soc_11G0(	14
milin vs linzhi	GO:0005737	cytoplasm	11/496	238/15848	0.12832	0.645121	0.604993	Soc_15G0(	11
milin vs linzhi	GO:0042127	regulation	2/496	20/15848	0.128379	0.645121	0.604993	Soc_10G0(	2
milin vs linzhi	GO:0098609	cell-cell ad	2/496	20/15848	0.128379	0.645121	0.604993	Soc_11G0(	2
milin vs linzhi	GO:0007049	cell cycle	3/496	40/15848	0.129024	0.645121	0.604993	Soc_13G0(	3
milin vs linzhi	GO:0030170	pyridoxal p	3/496	41/15848	0.136166	0.6652	0.623824	Soc_12G0(	3
milin vs linzhi	GO:0043087	regulation	2/496	21/15848	0.139087	0.6652	0.623824	Soc_3G000	2
milin vs linzhi	GO:0005794	Golgi appa	2/496	22/15848	0.14998	0.702033	0.658366	Soc_13G0(	2
milin vs linzhi	GO:0042613	MHC class	2/496	23/15848	0.161035	0.738075	0.692166	Soc_13G0(	2
milin vs linzhi	GO:0019882	antigen pr	2/496	24/15848	0.172231	0.764388	0.716842	Soc_13G0(	2

milin vs linzhi	GO:0004867	serine-typ	3/496	46/15848	0.173725	0.764388	0.716842	Soc_14G0(	3
milin vs linzhi	GO:0005230	extracellul	4/496	71/15848	0.182159	0.785786	0.736909	Soc_10G0(	4
milin vs linzhi	GO:0016491	oxidoredu	8/496	179/15848	0.19942	0.788658	0.739603	Soc_10G0(	8
milin vs linzhi	GO:0009058	biosynthet	3/496	50/15848	0.2056	0.788658	0.739603	Soc_12G0(	3
milin vs linzhi	GO:0003950	NAD+ AD	2/496	28/15848	0.218058	0.788658	0.739603	Soc_11G0(	2
milin vs linzhi	GO:0008285	negative r	2/496	28/15848	0.218058	0.788658	0.739603	Soc_11G0(	2
milin vs linzhi	GO:0043547	positive re	2/496	28/15848	0.218058	0.788658	0.739603	Soc_10G0(	2
milin vs linzhi	GO:0005234	extracellul	2/496	29/15848	0.229689	0.788658	0.739603	Soc_10G0(	2
milin vs linzhi	GO:0007601	visual perc	3/496	54/15848	0.238663	0.788658	0.739603	Soc_1G002	3
milin vs linzhi	GO:0005578	extracellul	2/496	30/15848	0.24136	0.788658	0.739603	Soc_17G0(	2
milin vs linzhi	GO:0016746	transferase	2/496	31/15848	0.253056	0.788658	0.739603	Soc_11G0(	2
milin vs linzhi	GO:0034220	ion transm	4/496	82/15848	0.254913	0.788658	0.739603	Soc_10G0(	4
milin vs linzhi	GO:0016301	kinase acti	3/496	56/15848	0.255521	0.788658	0.739603	Soc_6G000	3
milin vs linzhi	GO:0004888	transmeml	7/496	165/15848	0.259452	0.788658	0.739603	Soc_10G0(	7
milin vs linzhi	GO:0042157	lipoprotein	2/496	32/15848	0.264765	0.788658	0.739603	Soc_1G000	2
milin vs linzhi	GO:0003956	NAD(P)+-	1/496	10/15848	0.272446	0.788658	0.739603	Soc_20G0(	1
milin vs linzhi	GO:0004957	prostaglan	1/496	10/15848	0.272446	0.788658	0.739603	Soc_14G0(	1
milin vs linzhi	GO:0045087	innate imn	1/496	10/15848	0.272446	0.788658	0.739603	Soc_4G001	1
milin vs linzhi	GO:0004970	ionotropic	2/496	33/15848	0.276474	0.788658	0.739603	Soc_10G0(	2
milin vs linzhi	GO:0004252	serine-typ	6/496	143/15848	0.291009	0.788658	0.739603	Soc_1G000	6
milin vs linzhi	GO:0005212	structural (	1/496	11/15848	0.295231	0.788658	0.739603	Soc_21G0(	1
milin vs linzhi	GO:0006471	protein AD	1/496	11/15848	0.295231	0.788658	0.739603	Soc_20G0(	1
milin vs linzhi	GO:0007169	transmeml	1/496	11/15848	0.295231	0.788658	0.739603	Soc_3G000	1
milin vs linzhi	GO:0007224	smoothen(	1/496	11/15848	0.295231	0.788658	0.739603	Soc_14G0(	1
milin vs linzhi	GO:0022857	transmeml	4/496	88/15848	0.296815	0.788658	0.739603	Soc_18G0(	4
milin vs linzhi	GO:0008373	sialyltransf	2/496	36/15848	0.31149	0.788658	0.739603	Soc_10G0(	2
milin vs linzhi	GO:0015031	protein tra	2/496	36/15848	0.31149	0.788658	0.739603	Soc_18G0(	2
milin vs linzhi	GO:0004707	MAP kinas	1/496	12/15848	0.317304	0.788658	0.739603	Soc_18G0(	1
milin vs linzhi	GO:0004714	transmeml	1/496	12/15848	0.317304	0.788658	0.739603	Soc_3G000	1
milin vs linzhi	GO:0006270	DNA replic	1/496	12/15848	0.317304	0.788658	0.739603	Soc_13G0(	1
milin vs linzhi	GO:0008283	cell popula	1/496	12/15848	0.317304	0.788658	0.739603	Soc_20G0(	1
milin vs linzhi	GO:0019752	carboxylic	1/496	12/15848	0.317304	0.788658	0.739603	Soc_22G0(	1
milin vs linzhi	GO:0031175	neuron pr(	1/496	12/15848	0.317304	0.788658	0.739603	Soc_15G0(	1
milin vs linzhi	GO:0043066	negative r	1/496	12/15848	0.317304	0.788658	0.739603	Soc_6G000	1
milin vs linzhi	GO:0005856	cytoskelet(	4/496	93/15848	0.332338	0.788658	0.739603	Soc_11G0(	4
milin vs linzhi	GO:0001518	voltage-ga	1/496	13/15848	0.338686	0.788658	0.739603	Soc_6G000	1
milin vs linzhi	GO:0003730	mRNA 3'-(	1/496	13/15848	0.338686	0.788658	0.739603	Soc_11G0(	1
milin vs linzhi	GO:0004983	neuropept	1/496	13/15848	0.338686	0.788658	0.739603	Soc_13G0(	1

milin vs linzhi	GO:0008253	5'-nucleot	1/496	13/15848	0.338686	0.788658	0.739603	Soc_6G00(	1
milin vs linzhi	GO:0008146	sulfotransf	3/496	66/15848	0.341361	0.788658	0.739603	Soc_13G0(	3
milin vs linzhi	GO:0005635	nuclear en	1/496	14/15848	0.359401	0.788658	0.739603	Soc_15G0(	1
milin vs linzhi	GO:0006606	protein im	1/496	14/15848	0.359401	0.788658	0.739603	Soc_6G00(	1
milin vs linzhi	GO:0045028	G protein-	1/496	14/15848	0.359401	0.788658	0.739603	Soc_16G0(	1
milin vs linzhi	GO:0048015	phosphatid	1/496	14/15848	0.359401	0.788658	0.739603	Soc_9G00(	1
milin vs linzhi	GO:0016311	dephosph	4/496	98/15848	0.368053	0.788658	0.739603	Soc_11G0(	4
milin vs linzhi	GO:0001614	purinergic	1/496	15/15848	0.379468	0.788658	0.739603	Soc_10G0(	1
milin vs linzhi	GO:0003729	mRNA bin	1/496	15/15848	0.379468	0.788658	0.739603	Soc_10G0(	1
milin vs linzhi	GO:0003810	protein-gl	1/496	15/15848	0.379468	0.788658	0.739603	Soc_15G0(	1
milin vs linzhi	GO:0004931	extracellul	1/496	15/15848	0.379468	0.788658	0.739603	Soc_10G0(	1
milin vs linzhi	GO:0004993	G protein-	1/496	15/15848	0.379468	0.788658	0.739603	Soc_20G0(	1
milin vs linzhi	GO:0005834	heterotrim	1/496	15/15848	0.379468	0.788658	0.739603	Soc_11G0(	1
milin vs linzhi	GO:0033179	proton-tra	1/496	15/15848	0.379468	0.788658	0.739603	Soc_2G00(	1
milin vs linzhi	GO:0033198	response t	1/496	15/15848	0.379468	0.788658	0.739603	Soc_10G0(	1
milin vs linzhi	GO:0046873	metal ion t	1/496	15/15848	0.379468	0.788658	0.739603	Soc_19G0(	1
milin vs linzhi	GO:0051082	unfolded p	2/496	42/15848	0.380191	0.788658	0.739603	Soc_15G0(	2
milin vs linzhi	GO:0007218	neuropept	1/496	16/15848	0.398907	0.788658	0.739603	Soc_9G00(	1
milin vs linzhi	GO:0007596	blood coa	1/496	16/15848	0.398907	0.788658	0.739603	Soc_20G0(	1
milin vs linzhi	GO:0042803	protein ho	1/496	16/15848	0.398907	0.788658	0.739603	Soc_24G0(	1
milin vs linzhi	GO:0042626	ATPase-co	2/496	46/15848	0.424372	0.788658	0.739603	Soc_19G0(	2
milin vs linzhi	GO:0016791	phosphata	4/496	106/15848	0.424848	0.788658	0.739603	Soc_11G0(	4
milin vs linzhi	GO:0006869	lipid trans	2/496	47/15848	0.435164	0.788658	0.739603	Soc_1G00(	2
milin vs linzhi	GO:0008021	synaptic ve	1/496	18/15848	0.435981	0.788658	0.739603	Soc_13G0(	1
milin vs linzhi	GO:0018149	peptide cr	1/496	18/15848	0.435981	0.788658	0.739603	Soc_15G0(	1
milin vs linzhi	GO:0042310	vasoconstr	1/496	18/15848	0.435981	0.788658	0.739603	Soc_20G0(	1
milin vs linzhi	GO:0046854	phosphatid	1/496	18/15848	0.435981	0.788658	0.739603	Soc_9G00(	1
milin vs linzhi	GO:0007264	small GTPa	5/496	139/15848	0.440612	0.788658	0.739603	Soc_10G0(	5
milin vs linzhi	GO:0031012	extracellul	2/496	48/15848	0.445845	0.788658	0.739603	Soc_1G002	2
milin vs linzhi	GO:0005887	integral co	5/496	141/15848	0.452876	0.788658	0.739603	Soc_10G0(	5
milin vs linzhi	GO:0004181	metallocar	1/496	19/15848	0.453654	0.788658	0.739603	Soc_4G00(	1
milin vs linzhi	GO:0004386	helicase ac	1/496	19/15848	0.453654	0.788658	0.739603	Soc_13G0(	1
milin vs linzhi	GO:0051287	NAD bindi	1/496	19/15848	0.453654	0.788658	0.739603	Soc_3G00(	1
milin vs linzhi	GO:0098655	cation tran	1/496	19/15848	0.453654	0.788658	0.739603	Soc_10G0(	1
milin vs linzhi	GO:0007154	cell comm	2/496	49/15848	0.456413	0.788658	0.739603	Soc_16G0(	2
milin vs linzhi	GO:0000398	mRNA spli	1/496	20/15848	0.470773	0.788658	0.739603	Soc_6G00(	1
milin vs linzhi	GO:0004114	3',5'-cyclic	1/496	20/15848	0.470773	0.788658	0.739603	Soc_1G002	1
milin vs linzhi	GO:0005104	fibroblast (	1/496	20/15848	0.470773	0.788658	0.739603	Soc_11G0(	1

milin vs linzhi	GO:0005507	copper ior	1/496	20/15848	0.470773	0.788658	0.739603	Soc_22G0(	1
milin vs linzhi	GO:0006954	inflammat(	1/496	20/15848	0.470773	0.788658	0.739603	Soc_4G001	1
milin vs linzhi	GO:0005506	iron ion bi	4/496	114/15848	0.480292	0.788658	0.739603	Soc_12G0(	4
milin vs linzhi	GO:0005615	extracellul	3/496	83/15848	0.483753	0.788658	0.739603	Soc_14G0(	3
milin vs linzhi	GO:0003824	catalytic a	6/496	178/15848	0.485268	0.788658	0.739603	Soc_11G0(	6
milin vs linzhi	GO:0006914	autophagy	1/496	21/15848	0.487358	0.788658	0.739603	Soc_8G000	1
milin vs linzhi	GO:0007605	sensory pe	1/496	21/15848	0.487358	0.788658	0.739603	Soc_3G00(	1
milin vs linzhi	GO:0008236	serine-typ	1/496	21/15848	0.487358	0.788658	0.739603	Soc_13G0(	1
milin vs linzhi	GO:0045893	positive re	1/496	21/15848	0.487358	0.788658	0.739603	Soc_23G0(	1
milin vs linzhi	GO:0004866	endopepti	1/496	22/15848	0.503423	0.788658	0.739603	Soc_16G0(	1
milin vs linzhi	GO:0005520	insulin-like	1/496	22/15848	0.503423	0.788658	0.739603	Soc_4G000	1
milin vs linzhi	GO:0006418	tRNA amir	1/496	22/15848	0.503423	0.788658	0.739603	Soc_7G00(	1
milin vs linzhi	GO:0045892	negative r	1/496	22/15848	0.503423	0.788658	0.739603	Soc_13G0(	1
milin vs linzhi	GO:0045454	cell redox	2/496	54/15848	0.507438	0.788658	0.739603	Soc_11G0(	2
milin vs linzhi	GO:0007166	cell surface	3/496	86/15848	0.507457	0.788658	0.739603	Soc_13G0(	3
milin vs linzhi	GO:0006486	protein gly	3/496	87/15848	0.515231	0.788658	0.739603	Soc_10G0(	3
milin vs linzhi	GO:0001558	regulation	1/496	23/15848	0.518986	0.788658	0.739603	Soc_4G000	1
milin vs linzhi	GO:0008543	fibroblast	1/496	23/15848	0.518986	0.788658	0.739603	Soc_11G0(	1
milin vs linzhi	GO:0030001	metal ion	1/496	23/15848	0.518986	0.788658	0.739603	Soc_19G0(	1
milin vs linzhi	GO:0005096	GTPase ac	3/496	88/15848	0.52294	0.788658	0.739603	Soc_10G0(	3
milin vs linzhi	GO:0055114	oxidation-	13/496	413/15848	0.531674	0.788658	0.739603	Soc_10G0(	13
milin vs linzhi	GO:0005262	calcium ch	1/496	24/15848	0.534063	0.788658	0.739603	Soc_9G00(	1
milin vs linzhi	GO:0008378	galactosylt	1/496	24/15848	0.534063	0.788658	0.739603	Soc_18G0(	1
milin vs linzhi	GO:0007165	signal tran	11/496	351/15848	0.541539	0.788658	0.739603	Soc_10G0(	11
milin vs linzhi	GO:0004722	protein ser	1/496	25/15848	0.548668	0.788658	0.739603	Soc_11G0(	1
milin vs linzhi	GO:0008081	phosphori	1/496	25/15848	0.548668	0.788658	0.739603	Soc_18G0(	1
milin vs linzhi	GO:0008565	obsolete p	1/496	25/15848	0.548668	0.788658	0.739603	Soc_6G00(	1
milin vs linzhi	GO:0022848	acetylcholi	1/496	25/15848	0.548668	0.788658	0.739603	Soc_4G000	1
milin vs linzhi	GO:0005216	ion channe	8/496	258/15848	0.56099	0.788658	0.739603	Soc_10G0(	8
milin vs linzhi	GO:0004197	cysteine-ty	1/496	26/15848	0.562815	0.788658	0.739603	Soc_8G000	1
milin vs linzhi	GO:0007160	cell-matrix	1/496	26/15848	0.562815	0.788658	0.739603	Soc_24G0(	1
milin vs linzhi	GO:0007268	chemical s	1/496	26/15848	0.562815	0.788658	0.739603	Soc_5G001	1
milin vs linzhi	GO:0005328	neurotrans	1/496	27/15848	0.576521	0.792716	0.743408	Soc_9G00(	1
milin vs linzhi	GO:0015078	proton tra	1/496	27/15848	0.576521	0.792716	0.743408	Soc_2G00(	1
milin vs linzhi	GO:0015991	proton tra	1/496	27/15848	0.576521	0.792716	0.743408	Soc_2G00(	1
milin vs linzhi	GO:0004812	aminoacyl	1/496	29/15848	0.602658	0.818424	0.767518	Soc_7G00(	1
milin vs linzhi	GO:0006096	glycolytic	1/496	29/15848	0.602658	0.818424	0.767518	Soc_1G00(	1
milin vs linzhi	GO:0005044	scavenger	2/496	66/15848	0.616473	0.821314	0.770227	Soc_24G0(	2

milin vs linzhi	GO:0000786	nucleosom	4/496	137/15848	0.625335	0.821314	0.770227	Soc_21G00	4
milin vs linzhi	GO:0005922	connexin c	1/496	31/15848	0.627185	0.821314	0.770227	Soc_4G000	1
milin vs linzhi	GO:0006897	endocytos	1/496	31/15848	0.627185	0.821314	0.770227	Soc_5G000	1
milin vs linzhi	GO:0016702	oxidoredu	1/496	31/15848	0.627185	0.821314	0.770227	Soc_24G00	1
milin vs linzhi	GO:0045211	postsynap	1/496	31/15848	0.627185	0.821314	0.770227	Soc_4G000	1
milin vs linzhi	GO:0006457	protein fol	1/496	32/15848	0.638876	0.831673	0.779942	Soc_24G00	1
milin vs linzhi	GO:0004842	ubiquitin-	3/496	105/15848	0.642783	0.831716	0.779983	Soc_12G00	3
milin vs linzhi	GO:0046872	metal ion	6/496	210/15848	0.646471	0.831716	0.779983	Soc_13G00	6
milin vs linzhi	GO:0016887	ATPase ac	2/496	71/15848	0.656115	0.836486	0.784456	Soc_19G00	2
milin vs linzhi	GO:0006887	exocytosis	1/496	34/15848	0.661172	0.836486	0.784456	Soc_10G00	1
milin vs linzhi	GO:0008083	growth fac	3/496	108/15848	0.661585	0.836486	0.784456	Soc_11G00	3
milin vs linzhi	GO:0006811	ion transp	8/496	285/15848	0.672335	0.845221	0.792648	Soc_10G00	8
milin vs linzhi	GO:0006836	neurotrans	1/496	36/15848	0.682094	0.852617	0.799583	Soc_9G000	1
milin vs linzhi	GO:0007399	nervous sy	1/496	37/15848	0.692066	0.860195	0.80669	Soc_7G000	1
milin vs linzhi	GO:0008080	N-acetyltr	1/496	38/15848	0.701726	0.867302	0.813355	Soc_15G00	1
milin vs linzhi	GO:0005783	endoplasm	1/496	39/15848	0.711084	0.873957	0.819596	Soc_9G000	1
milin vs linzhi	GO:0007275	multicellul	2/496	80/15848	0.719134	0.878941	0.82427	Soc_11G00	2
milin vs linzhi	GO:0016192	vesicle-me	2/496	82/15848	0.731745	0.889413	0.834091	Soc_10G00	2
milin vs linzhi	GO:0000166	nucleotide	1/496	43/15848	0.745674	0.896439	0.84068	Soc_7G000	1
milin vs linzhi	GO:0006816	calcium io	1/496	43/15848	0.745674	0.896439	0.84068	Soc_9G000	1
milin vs linzhi	GO:0046983	protein dir	6/496	236/15848	0.752204	0.899374	0.843432	Soc_16G00	6
milin vs linzhi	GO:0030036	actin cytos	1/496	45/15848	0.761387	0.905433	0.849114	Soc_14G00	1
milin vs linzhi	GO:0008289	lipid bindi	2/496	88/15848	0.766721	0.906874	0.850465	Soc_1G000	2
milin vs linzhi	GO:0007156	homophili	3/496	131/15848	0.782018	0.920021	0.862795	Soc_10G00	3
milin vs linzhi	GO:0016787	hydrolase	3/496	134/15848	0.794781	0.928501	0.870748	Soc_11G00	3
milin vs linzhi	GO:0015074	DNA integ	2/496	94/15848	0.797667	0.928501	0.870748	Soc_12G00	2
milin vs linzhi	GO:0036459	thiol-depe	1/496	52/15848	0.809134	0.932525	0.874521	Soc_11G00	1
milin vs linzhi	GO:0004984	olfactory r	1/496	53/15848	0.815127	0.932525	0.874521	Soc_16G00	1
milin vs linzhi	GO:0035091	phosphatid	1/496	53/15848	0.815127	0.932525	0.874521	Soc_9G000	1
milin vs linzhi	GO:0000287	magnesiur	1/496	54/15848	0.820932	0.932525	0.874521	Soc_6G000	1
milin vs linzhi	GO:0004872	signaling r	1/496	55/15848	0.826556	0.932525	0.874521	Soc_10G00	1
milin vs linzhi	GO:0070588	calcium io	1/496	55/15848	0.826556	0.932525	0.874521	Soc_9G000	1
milin vs linzhi	GO:0005739	mitochond	1/496	57/15848	0.83728	0.939804	0.881347	Soc_5G001	1
milin vs linzhi	GO:0008138	protein tyr	1/496	58/15848	0.842391	0.940741	0.882226	Soc_11G00	1
milin vs linzhi	GO:0007155	cell adhesi	3/496	149/15848	0.849563	0.942708	0.884071	Soc_15G00	3
milin vs linzhi	GO:0006508	proteolysis	10/496	422/15848	0.855054	0.942708	0.884071	Soc_10G00	10
milin vs linzhi	GO:0004222	metalloend	2/496	109/15848	0.85965	0.942708	0.884071	Soc_10G00	2
milin vs linzhi	GO:0004674	protein ser	2/496	110/15848	0.863093	0.942708	0.884071	Soc_10G00	2

milin vs linzhi	GO:0016579	protein de	1/496	63/15848	0.865641	0.942708	0.884071	Soc_11G0(	1
milin vs linzhi	GO:0016567	protein ub	1/496	64/15848	0.869863	0.942708	0.884071	Soc_12G0(	1
milin vs linzhi	GO:0006886	intracellula	2/496	115/15848	0.879176	0.945306	0.886507	Soc_18G0(	2
milin vs linzhi	GO:0003723	RNA bindi	4/496	202/15848	0.880853	0.945306	0.886507	Soc_10G0(	4
milin vs linzhi	GO:0005179	hormone a	1/496	72/15848	0.899203	0.960314	0.900582	Soc_11G0(	1
milin vs linzhi	GO:0016705	oxidoredu	1/496	74/15848	0.905442	0.962306	0.902449	Soc_12G0(	1
milin vs linzhi	GO:0006629	lipid metal	1/496	76/15848	0.911295	0.963185	0.903274	Soc_18G0(	1
milin vs linzhi	GO:0004871	obsolete s	1/496	78/15848	0.916787	0.963185	0.903274	Soc_11G0(	1
milin vs linzhi	GO:0005249	voltage-ga	1/496	79/15848	0.919404	0.963185	0.903274	Soc_14G0(	1
milin vs linzhi	GO:0005089	Rho guany	1/496	84/15848	0.931308	0.965897	0.905818	Soc_9G00(	1
milin vs linzhi	GO:0005622	intracellula	8/496	400/15848	0.936254	0.965897	0.905818	Soc_11G0(	8
milin vs linzhi	GO:0005882	intermedia	1/496	87/15848	0.937591	0.965897	0.905818	Soc_6G00(	1
milin vs linzhi	GO:0035023	regulation	1/496	88/15848	0.939555	0.965897	0.905818	Soc_9G00(	1
milin vs linzhi	GO:0020037	heme binc	1/496	91/15848	0.945084	0.967063	0.906911	Soc_12G0(	1
milin vs linzhi	GO:0003779	actin bindi	1/496	105/15848	0.964913	0.982782	0.921652	Soc_14G0(	1
milin vs linzhi	GO:0035556	intracellula	2/496	170/15848	0.971421	0.98485	0.923592	Soc_5G00(	2
milin vs linzhi	GO:0006813	potassium	1/496	125/15848	0.981511	0.990516	0.928905	Soc_14G0(	1
milin vs linzhi	GO:0003700	DNA-bind	7/496	476/15848	0.993191	0.997726	0.935667	Soc_1G001	7
milin vs linzhi	GO:0043565	sequence-	1/496	191/15848	0.997781	0.997781	0.935718	Soc_24G0(	1
milin vs zangga	GO:0006935	chemotaxi	6/441	33/15848	0.000263	0.038444	0.037187	Soc_15G0(	6
milin vs zangga	GO:0008417	fucosyltrar	4/441	13/15848	0.000346	0.038444	0.037187	Soc_13G0(	4
milin vs zangga	GO:0008009	chemokine	5/441	31/15848	0.001524	0.112805	0.109114	Soc_11G0(	5
milin vs zangga	GO:0004950	chemokine	4/441	23/15848	0.003441	0.166361	0.160918	Soc_15G0(	4
milin vs zangga	GO:0006814	sodium ior	6/441	54/15848	0.003747	0.166361	0.160918	Soc_20G0(	6
milin vs zangga	GO:0022848	acetylcholi	4/441	25/15848	0.004705	0.174095	0.168399	Soc_4G00(	4
milin vs zangga	GO:0016758	transferase	5/441	44/15848	0.007254	0.210595	0.203705	Soc_5G00(	5
milin vs zangga	GO:0005272	sodium ch	3/441	15/15848	0.007589	0.210595	0.203705	Soc_22G0(	3
milin vs zangga	GO:0007596	blood coa	3/441	16/15848	0.00915	0.225698	0.218314	Soc_18G0(	3
milin vs zangga	GO:0045211	postsynap	4/441	31/15848	0.010268	0.227949	0.220491	Soc_4G00(	4
milin vs zangga	GO:0004713	protein tyr	7/441	91/15848	0.013439	0.27123	0.262356	Soc_11G0(	7
milin vs zangga	GO:0005242	inward rec	3/441	21/15848	0.019614	0.334942	0.323984	Soc_11G0(	3
milin vs zangga	GO:0007605	sensory pe	3/441	21/15848	0.019614	0.334942	0.323984	Soc_3G00(	3
milin vs zangga	GO:0030971	receptor ty	2/441	10/15848	0.029988	0.475521	0.459963	Soc_15G0(	2
milin vs zangga	GO:0005230	extracellul	5/441	71/15848	0.047582	0.615826	0.595678	Soc_13G0(	5
milin vs zangga	GO:0004983	neuropept	2/441	13/15848	0.04921	0.615826	0.595678	Soc_13G0(	2
milin vs zangga	GO:0000226	microtubu	3/441	30/15848	0.049884	0.615826	0.595678	Soc_15G0(	3
milin vs zangga	GO:0004725	protein tyr	4/441	50/15848	0.049932	0.615826	0.595678	Soc_15G0(	4
milin vs zangga	GO:0008168	methyltrar	4/441	51/15848	0.053041	0.616629	0.596455	Soc_15G0(	4

milin vs zangga	GO:0005635	nuclear en	2/441	14/15848	0.056379	0.616629	0.596455	Soc_12G00	2
milin vs zangga	GO:0006351	transcripti	4/441	53/15848	0.059567	0.616629	0.596455	Soc_15G00	4
milin vs zangga	GO:0003810	protein-gl	2/441	15/15848	0.063885	0.616629	0.596455	Soc_15G00	2
milin vs zangga	GO:0051225	spindle ass	2/441	15/15848	0.063885	0.616629	0.596455	Soc_4G000	2
milin vs zangga	GO:0034220	ion transm	5/441	82/15848	0.078315	0.677804	0.655629	Soc_13G00	5
milin vs zangga	GO:0003684	damaged	2/441	18/15848	0.088191	0.677804	0.655629	Soc_7G000	2
milin vs zangga	GO:0018149	peptide cr	2/441	18/15848	0.088191	0.677804	0.655629	Soc_15G00	2
milin vs zangga	GO:0004888	transmeml	8/441	165/15848	0.090269	0.677804	0.655629	Soc_13G00	8
milin vs zangga	GO:0005576	extracellul	13/441	311/15848	0.095364	0.677804	0.655629	Soc_11G00	13
milin vs zangga	GO:0005086	ARF guany	2/441	19/15848	0.096815	0.677804	0.655629	Soc_17G00	2
milin vs zangga	GO:0032012	regulation	2/441	19/15848	0.096815	0.677804	0.655629	Soc_17G00	2
milin vs zangga	GO:0006281	DNA repai	5/441	88/15848	0.098666	0.677804	0.655629	Soc_17G00	5
milin vs zangga	GO:0008152	metabolic	10/441	226/15848	0.100687	0.677804	0.655629	Soc_11G00	10
milin vs zangga	GO:0003924	GTPase ac	13/441	314/15848	0.100755	0.677804	0.655629	Soc_1G000	13
milin vs zangga	GO:0007015	actin filam	2/441	21/15848	0.114724	0.727675	0.703868	Soc_20G00	2
milin vs zangga	GO:0043087	regulation	2/441	21/15848	0.114724	0.727675	0.703868	Soc_3G000	2
milin vs zangga	GO:0045892	negative r	2/441	22/15848	0.123971	0.753226	0.728583	Soc_13G00	2
milin vs zangga	GO:0004298	threonine-	2/441	23/15848	0.13339	0.753226	0.728583	Soc_17G00	2
milin vs zangga	GO:0005839	proteasom	2/441	23/15848	0.13339	0.753226	0.728583	Soc_17G00	2
milin vs zangga	GO:0051603	proteolysis	2/441	23/15848	0.13339	0.753226	0.728583	Soc_17G00	2
milin vs zangga	GO:0004867	serine-typ	3/441	46/15848	0.135716	0.753226	0.728583	Soc_14G00	3
milin vs zangga	GO:0005923	bicellular t	4/441	74/15848	0.150795	0.777059	0.751636	Soc_13G00	4
milin vs zangga	GO:0007601	visual perc	3/441	54/15848	0.189794	0.777059	0.751636	Soc_17G00	3
milin vs zangga	GO:0005234	extracellul	2/441	29/15848	0.192627	0.777059	0.751636	Soc_10G00	2
milin vs zangga	GO:0016301	kinase acti	3/441	56/15848	0.204084	0.777059	0.751636	Soc_17G00	3
milin vs zangga	GO:0006486	protein gly	4/441	87/15848	0.223564	0.777059	0.751636	Soc_13G00	4
milin vs zangga	GO:0004970	ionotropic	2/441	33/15848	0.23372	0.777059	0.751636	Soc_10G00	2
milin vs zangga	GO:0004957	prostaglan	1/441	10/15848	0.245949	0.777059	0.751636	Soc_13G00	1
milin vs zangga	GO:0006885	regulation	1/441	10/15848	0.245949	0.777059	0.751636	Soc_3G000	1
milin vs zangga	GO:0015385	sodium:pr	1/441	10/15848	0.245949	0.777059	0.751636	Soc_3G000	1
milin vs zangga	GO:0006470	protein de	5/441	123/15848	0.258055	0.777059	0.751636	Soc_11G00	5
milin vs zangga	GO:0015031	protein tra	2/441	36/15848	0.264865	0.777059	0.751636	Soc_18G00	2
milin vs zangga	GO:0005247	voltage-ga	1/441	11/15848	0.266945	0.777059	0.751636	Soc_13G00	1
milin vs zangga	GO:0007169	transmeml	1/441	11/15848	0.266945	0.777059	0.751636	Soc_3G000	1
milin vs zangga	GO:0007224	smoothen	1/441	11/15848	0.266945	0.777059	0.751636	Soc_14G00	1
milin vs zangga	GO:0030414	peptidase	1/441	11/15848	0.266945	0.777059	0.751636	Soc_19G00	1
milin vs zangga	GO:0044212	transcripti	1/441	11/15848	0.266945	0.777059	0.751636	Soc_11G00	1
milin vs zangga	GO:0006813	potassium	5/441	125/15848	0.26865	0.777059	0.751636	Soc_11G00	5

milin vs zangga	GO:0006811	ion transp	10/441	285/15848	0.271688	0.777059	0.751636	Soc_10G0(	10
milin vs zangga	GO:0005215	transporte	2/441	37/15848	0.275258	0.777059	0.751636	Soc_20G0(	2
milin vs zangga	GO:0006955	immune re	5/441	127/15848	0.279339	0.777059	0.751636	Soc_11G0(	5
milin vs zangga	GO:0008080	N-acetyltr	2/441	38/15848	0.285643	0.777059	0.751636	Soc_15G0(	2
milin vs zangga	GO:0000139	Golgi mem	1/441	12/15848	0.287358	0.777059	0.751636	Soc_6G000	1
milin vs zangga	GO:0004707	MAP kinas	1/441	12/15848	0.287358	0.777059	0.751636	Soc_18G0(	1
milin vs zangga	GO:0004714	transmeml	1/441	12/15848	0.287358	0.777059	0.751636	Soc_3G000	1
milin vs zangga	GO:0008283	cell popula	1/441	12/15848	0.287358	0.777059	0.751636	Soc_20G0(	1
milin vs zangga	GO:0030833	regulation	1/441	12/15848	0.287358	0.777059	0.751636	Soc_7G000	1
milin vs zangga	GO:0031175	neuron pr	1/441	12/15848	0.287358	0.777059	0.751636	Soc_15G0(	1
milin vs zangga	GO:0043066	negative re	1/441	12/15848	0.287358	0.777059	0.751636	Soc_1G000	1
milin vs zangga	GO:0051056	regulation	1/441	12/15848	0.287358	0.777059	0.751636	Soc_23G0(	1
milin vs zangga	GO:0005216	ion channe	9/441	258/15848	0.292059	0.777059	0.751636	Soc_10G0(	9
milin vs zangga	GO:0007049	cell cycle	2/441	40/15848	0.306361	0.777059	0.751636	Soc_20G0(	2
milin vs zangga	GO:0005911	cell-cell ju	1/441	13/15848	0.307203	0.777059	0.751636	Soc_11G0(	1
milin vs zangga	GO:0015299	solute:prot	1/441	13/15848	0.307203	0.777059	0.751636	Soc_3G000	1
milin vs zangga	GO:0019904	protein do	1/441	13/15848	0.307203	0.777059	0.751636	Soc_6G000	1
milin vs zangga	GO:0032947	molecular	1/441	13/15848	0.307203	0.777059	0.751636	Soc_13G0(	1
milin vs zangga	GO:0016887	ATPase ac	3/441	71/15848	0.316604	0.777059	0.751636	Soc_19G0(	3
milin vs zangga	GO:0005179	hormone a	3/441	72/15848	0.32426	0.777059	0.751636	Soc_13G0(	3
milin vs zangga	GO:0003714	transcripti	1/441	14/15848	0.326497	0.777059	0.751636	Soc_3G000	1
milin vs zangga	GO:0005158	insulin rec	1/441	14/15848	0.326497	0.777059	0.751636	Soc_4G000	1
milin vs zangga	GO:0006606	protein im	1/441	14/15848	0.326497	0.777059	0.751636	Soc_6G000	1
milin vs zangga	GO:0048015	phosphati	1/441	14/15848	0.326497	0.777059	0.751636	Soc_9G000	1
milin vs zangga	GO:0005737	cytoplasm	8/441	238/15848	0.344013	0.777059	0.751636	Soc_13G0(	8
milin vs zangga	GO:0004993	G protein-	1/441	15/15848	0.345255	0.777059	0.751636	Soc_20G0(	1
milin vs zangga	GO:0006364	rRNA proc	1/441	15/15848	0.345255	0.777059	0.751636	Soc_5G000	1
milin vs zangga	GO:0008033	tRNA proc	1/441	15/15848	0.345255	0.777059	0.751636	Soc_23G0(	1
milin vs zangga	GO:0030131	clathrin ad	1/441	15/15848	0.345255	0.777059	0.751636	Soc_8G000	1
milin vs zangga	GO:0030286	dynein cor	1/441	15/15848	0.345255	0.777059	0.751636	Soc_4G000	1
milin vs zangga	GO:0033179	proton-tra	1/441	15/15848	0.345255	0.777059	0.751636	Soc_11G0(	1
milin vs zangga	GO:0005885	Arp2/3 pro	1/441	16/15848	0.363492	0.777059	0.751636	Soc_7G000	1
milin vs zangga	GO:0006310	DNA recor	1/441	16/15848	0.363492	0.777059	0.751636	Soc_4G000	1
milin vs zangga	GO:0007218	neuropept	1/441	16/15848	0.363492	0.777059	0.751636	Soc_3G000	1
milin vs zangga	GO:0016042	lipid catab	1/441	16/15848	0.363492	0.777059	0.751636	Soc_13G0(	1
milin vs zangga	GO:0004252	serine-typ	5/441	143/15848	0.366992	0.777059	0.751636	Soc_13G0(	5
milin vs zangga	GO:0016757	transferase	2/441	47/15848	0.377637	0.777059	0.751636	Soc_15G0(	2
milin vs zangga	GO:0034314	Arp2/3 cor	1/441	17/15848	0.381222	0.777059	0.751636	Soc_7G000	1

milin vs zangga	GO:0051536	iron-sulfur	1/441	17/15848	0.381222	0.777059	0.751636	Soc_23G00		1
milin vs zangga	GO:0005198	structural r	6/441	181/15848	0.390559	0.777059	0.751636	Soc_14G00		6
milin vs zangga	GO:0006886	intracellula	4/441	115/15848	0.39826	0.777059	0.751636	Soc_6G000		4
milin vs zangga	GO:0004702	obsolete s	1/441	18/15848	0.398459	0.777059	0.751636	Soc_22G00		1
milin vs zangga	GO:0007178	transmeml	1/441	18/15848	0.398459	0.777059	0.751636	Soc_22G00		1
milin vs zangga	GO:0008484	sulfuric est	1/441	18/15848	0.398459	0.777059	0.751636	Soc_3G000		1
milin vs zangga	GO:0017137	Rab GTPas	1/441	18/15848	0.398459	0.777059	0.751636	Soc_1G002		1
milin vs zangga	GO:0042310	vasoconstr	1/441	18/15848	0.398459	0.777059	0.751636	Soc_20G00		1
milin vs zangga	GO:0046854	phosphatid	1/441	18/15848	0.398459	0.777059	0.751636	Soc_9G000		1
milin vs zangga	GO:0016192	vesicle-me	3/441	82/15848	0.400356	0.777059	0.751636	Soc_12G00		3
milin vs zangga	GO:0005201	extracellula	1/441	19/15848	0.415217	0.777059	0.751636	Soc_18G00		1
milin vs zangga	GO:0005743	mitochond	1/441	19/15848	0.415217	0.777059	0.751636	Soc_12G00		1
milin vs zangga	GO:0006366	transcriptio	1/441	19/15848	0.415217	0.777059	0.751636	Soc_4G000		1
milin vs zangga	GO:0005102	signaling r	2/441	52/15848	0.426662	0.777059	0.751636	Soc_15G00		2
milin vs zangga	GO:0000398	mRNA spli	1/441	20/15848	0.431509	0.777059	0.751636	Soc_6G000		1
milin vs zangga	GO:0004114	3',5'-cyclic	1/441	20/15848	0.431509	0.777059	0.751636	Soc_18G00		1
milin vs zangga	GO:0006413	translation	1/441	20/15848	0.431509	0.777059	0.751636	Soc_12G00		1
milin vs zangga	GO:0042127	regulation	1/441	20/15848	0.431509	0.777059	0.751636	Soc_10G00		1
milin vs zangga	GO:0005096	GTPase ac	3/441	88/15848	0.444923	0.777059	0.751636	Soc_10G00		3
milin vs zangga	GO:0008289	lipid bindi	3/441	88/15848	0.444923	0.777059	0.751636	Soc_13G00		3
milin vs zangga	GO:0006338	chromatin	1/441	21/15848	0.447349	0.777059	0.751636	Soc_8G000		1
milin vs zangga	GO:0006914	autophagy	1/441	21/15848	0.447349	0.777059	0.751636	Soc_8G000		1
milin vs zangga	GO:0015629	actin cytos	1/441	21/15848	0.447349	0.777059	0.751636	Soc_7G000		1
milin vs zangga	GO:0045893	positive re	1/441	21/15848	0.447349	0.777059	0.751636	Soc_8G000		1
milin vs zangga	GO:0004872	signaling r	2/441	55/15848	0.455094	0.777059	0.751636	Soc_10G00		2
milin vs zangga	GO:0003899	DNA-direc	1/441	22/15848	0.462748	0.777059	0.751636	Soc_6G000		1
milin vs zangga	GO:0005794	Golgi appa	1/441	22/15848	0.462748	0.777059	0.751636	Soc_5G000		1
milin vs zangga	GO:0006418	tRNA amir	1/441	22/15848	0.462748	0.777059	0.751636	Soc_6G000		1
milin vs zangga	GO:0004890	GABA-A re	1/441	23/15848	0.477719	0.777059	0.751636	Soc_13G00		1
milin vs zangga	GO:0005856	cytoskelete	3/441	93/15848	0.481036	0.777059	0.751636	Soc_11G00		3
milin vs zangga	GO:0005085	guanyl-nu	2/441	58/15848	0.4827	0.777059	0.751636	Soc_10G00		2
milin vs zangga	GO:0046983	protein dir	7/441	236/15848	0.485916	0.777059	0.751636	Soc_17G00		7
milin vs zangga	GO:0003723	RNA bindi	6/441	202/15848	0.49384	0.777059	0.751636	Soc_18G00		6
milin vs zangga	GO:0006821	chloride tr	1/441	25/15848	0.506423	0.777059	0.751636	Soc_13G00		1
milin vs zangga	GO:0007010	cytoskelete	1/441	25/15848	0.506423	0.777059	0.751636	Soc_11G00		1
milin vs zangga	GO:0008081	phosphori	1/441	25/15848	0.506423	0.777059	0.751636	Soc_18G00		1
milin vs zangga	GO:0008565	obsolete p	1/441	25/15848	0.506423	0.777059	0.751636	Soc_6G000		1
milin vs zangga	GO:0003707	steroid ho	2/441	61/15848	0.509426	0.777059	0.751636	Soc_19G00		2

milin vs zangga	GO:0043401	steroid ho	2/441	61/15848	0.509426	0.777059	0.751636	Soc_19G0(	2
milin vs zangga	GO:0016311	dephosph(	3/441	98/15848	0.516006	0.777059	0.751636	Soc_11G0(	3
milin vs zangga	GO:0004197	cysteine-ty	1/441	26/15848	0.520179	0.777059	0.751636	Soc_8G00(	1
milin vs zangga	GO:0016579	protein de	2/441	63/15848	0.526735	0.777059	0.751636	Soc_17G0(	2
milin vs zangga	GO:0046872	metal ion	6/441	210/15848	0.53176	0.777059	0.751636	Soc_11G0(	6
milin vs zangga	GO:0005200	structural (	1/441	27/15848	0.533553	0.777059	0.751636	Soc_1G001	1
milin vs zangga	GO:0009607	response t	1/441	27/15848	0.533553	0.777059	0.751636	Soc_13G0(	1
milin vs zangga	GO:0015078	proton tra	1/441	27/15848	0.533553	0.777059	0.751636	Soc_11G0(	1
milin vs zangga	GO:0015991	proton tra	1/441	27/15848	0.533553	0.777059	0.751636	Soc_11G0(	1
milin vs zangga	GO:0003950	NAD+ AD	1/441	28/15848	0.546555	0.777059	0.751636	Soc_2G00(	1
milin vs zangga	GO:0043547	positive re	1/441	28/15848	0.546555	0.777059	0.751636	Soc_10G0(	1
milin vs zangga	GO:0005044	scavenger	2/441	66/15848	0.551913	0.777059	0.751636	Soc_24G0(	2
milin vs zangga	GO:0007018	microtubu	2/441	66/15848	0.551913	0.777059	0.751636	Soc_1G00(	2
milin vs zangga	GO:0008146	sulfotransf	2/441	66/15848	0.551913	0.777059	0.751636	Soc_11G0(	2
milin vs zangga	GO:0003824	catalytic a(	5/441	178/15848	0.554578	0.777059	0.751636	Soc_12G0(	5
milin vs zangga	GO:0005887	integral co	4/441	141/15848	0.554866	0.777059	0.751636	Soc_14G0(	4
milin vs zangga	GO:0004812	aminoacyl	1/441	29/15848	0.559195	0.777059	0.751636	Soc_6G00(	1
milin vs zangga	GO:0008233	peptidase	1/441	29/15848	0.559195	0.777059	0.751636	Soc_1G00(	1
milin vs zangga	GO:0003777	microtubu	2/441	67/15848	0.560094	0.777059	0.751636	Soc_1G00(	2
milin vs zangga	GO:0051015	actin filam	2/441	67/15848	0.560094	0.777059	0.751636	Soc_11G0(	2
milin vs zangga	GO:0003779	actin bindi	3/441	105/15848	0.562752	0.777059	0.751636	Soc_20G0(	3
milin vs zangga	GO:0004842	ubiquitin-	3/441	105/15848	0.562752	0.777059	0.751636	Soc_1G00(	3
milin vs zangga	GO:0016791	phosphata	3/441	106/15848	0.569204	0.777059	0.751636	Soc_11G0(	3
milin vs zangga	GO:0005578	extracellul	1/441	30/15848	0.571484	0.777059	0.751636	Soc_17G0(	1
milin vs zangga	GO:0009190	cyclic nucl	1/441	30/15848	0.571484	0.777059	0.751636	Soc_13G0(	1
milin vs zangga	GO:0016849	phosphoru	1/441	30/15848	0.571484	0.777059	0.751636	Soc_13G0(	1
milin vs zangga	GO:0019001	guanyl nu(	1/441	30/15848	0.571484	0.777059	0.751636	Soc_9G00(	1
milin vs zangga	GO:0031683	G-protein	1/441	30/15848	0.571484	0.777059	0.751636	Soc_9G00(	1
milin vs zangga	GO:0008083	growth fa(	3/441	108/15848	0.58193	0.777059	0.751636	Soc_21G0(	3
milin vs zangga	GO:0006897	endocytos	1/441	31/15848	0.583431	0.777059	0.751636	Soc_5G00(	1
milin vs zangga	GO:0016746	transferase	1/441	31/15848	0.583431	0.777059	0.751636	Soc_11G0(	1
milin vs zangga	GO:0071805	potassium	1/441	31/15848	0.583431	0.777059	0.751636	Soc_14G0(	1
milin vs zangga	GO:0016459	myosin co	2/441	70/15848	0.583992	0.777059	0.751636	Soc_21G0(	2
milin vs zangga	GO:0003774	motor acti	2/441	71/15848	0.591742	0.777059	0.751636	Soc_21G0(	2
milin vs zangga	GO:0007017	microtubu	1/441	32/15848	0.595045	0.777059	0.751636	Soc_1G001	1
milin vs zangga	GO:0008092	cytoskelet	1/441	32/15848	0.595045	0.777059	0.751636	Soc_15G0(	1
milin vs zangga	GO:0042157	lipoprotein	1/441	32/15848	0.595045	0.777059	0.751636	Soc_3G001	1
milin vs zangga	GO:0016616	oxidoredu	1/441	33/15848	0.606337	0.787174	0.76142	Soc_15G0(	1

milin vs zangga	GO:0042981	regulation	2/441	77/15848	0.635979	0.819168	0.792368	Soc_11G00	2
milin vs zangga	GO:0008305	integrin co	1/441	36/15848	0.638361	0.819168	0.792368	Soc_6G000	1
milin vs zangga	GO:0005249	voltage-ga	2/441	79/15848	0.649866	0.82914	0.802013	Soc_14G00	2
milin vs zangga	GO:0006260	DNA replic	1/441	39/15848	0.667785	0.847133	0.819418	Soc_1G001	1
milin vs zangga	GO:0006812	cation tran	1/441	40/15848	0.677052	0.854009	0.826069	Soc_3G000	1
milin vs zangga	GO:0003743	translation	1/441	41/15848	0.686062	0.85711	0.829068	Soc_12G00	1
milin vs zangga	GO:0051082	unfolded p	1/441	42/15848	0.69482	0.85711	0.829068	Soc_15G00	1
milin vs zangga	GO:0007166	cell surface	2/441	86/15848	0.695167	0.85711	0.829068	Soc_13G00	2
milin vs zangga	GO:0035556	intracellula	4/441	170/15848	0.700345	0.85711	0.829068	Soc_13G00	4
milin vs zangga	GO:0005882	intermedia	2/441	87/15848	0.701228	0.85711	0.829068	Soc_6G000	2
milin vs zangga	GO:0000166	nucleotide	1/441	43/15848	0.703335	0.85711	0.829068	Soc_6G000	1
milin vs zangga	GO:0022857	transmemb	2/441	88/15848	0.707189	0.85711	0.829068	Soc_18G00	2
milin vs zangga	GO:0007156	homophilic	3/441	131/15848	0.710397	0.85711	0.829068	Soc_15G00	3
milin vs zangga	GO:0005874	microtubu	1/441	45/15848	0.71966	0.863592	0.835338	Soc_1G001	1
milin vs zangga	GO:0042626	ATPase-co	1/441	46/15848	0.727483	0.868287	0.839879	Soc_19G00	1
milin vs zangga	GO:0006869	lipid transp	1/441	47/15848	0.735089	0.872672	0.844121	Soc_3G001	1
milin vs zangga	GO:0015074	DNA integ	2/441	94/15848	0.740897	0.874889	0.846265	Soc_12G00	2
milin vs zangga	GO:0008017	microtubu	2/441	96/15848	0.751373	0.882565	0.85369	Soc_13G00	2
milin vs zangga	GO:0036459	thiol-depe	1/441	52/15848	0.770054	0.897798	0.868425	Soc_8G000	1
milin vs zangga	GO:0004984	olfactory r	1/441	53/15848	0.776474	0.897798	0.868425	Soc_16G00	1
milin vs zangga	GO:0035091	phosphatid	1/441	53/15848	0.776474	0.897798	0.868425	Soc_9G000	1
milin vs zangga	GO:0007155	cell adhesi	3/441	149/15848	0.788019	0.906427	0.876771	Soc_15G00	3
milin vs zangga	GO:0005739	mitochond	1/441	57/15848	0.800413	0.915937	0.88597	Soc_8G000	1
milin vs zangga	GO:0008138	protein tyr	1/441	58/15848	0.805987	0.917586	0.887565	Soc_11G00	1
milin vs zangga	GO:0004674	protein ser	2/441	110/15848	0.814939	0.920312	0.890202	Soc_17G00	2
milin vs zangga	GO:0006915	apoptotic	1/441	60/15848	0.816673	0.920312	0.890202	Soc_8G000	1
milin vs zangga	GO:0008076	voltage-ga	1/441	61/15848	0.821794	0.921406	0.89126	Soc_20G00	1
milin vs zangga	GO:0016055	Wnt signal	1/441	62/15848	0.826772	0.922329	0.892153	Soc_15G00	1
milin vs zangga	GO:0016567	protein ub	1/441	64/15848	0.836316	0.928311	0.897939	Soc_17G00	1
milin vs zangga	GO:0008237	metallopep	1/441	69/15848	0.857942	0.941606	0.910799	Soc_10G00	1
milin vs zangga	GO:0051260	protein ho	1/441	69/15848	0.857942	0.941606	0.910799	Soc_20G00	1
milin vs zangga	GO:0007165	signal tran	7/441	351/15848	0.861018	0.941606	0.910799	Soc_10G00	7
milin vs zangga	GO:0016491	oxidoredu	3/441	179/15848	0.878716	0.956249	0.924964	Soc_10G00	3
milin vs zangga	GO:0004871	obsolete s	1/441	78/15848	0.889938	0.963737	0.932207	Soc_9G000	1
milin vs zangga	GO:0007275	multicellul	1/441	80/15848	0.896007	0.965059	0.933485	Soc_15G00	1
milin vs zangga	GO:0006508	proteolysis	8/441	422/15848	0.904913	0.965059	0.933485	Soc_10G00	8
milin vs zangga	GO:0005089	Rho guany	1/441	84/15848	0.907163	0.965059	0.933485	Soc_19G00	1
milin vs zangga	GO:0005840	ribosome	1/441	87/15848	0.914739	0.965059	0.933485	Soc_18G00	1

milin vs zangga	GO:0035023	regulation	1/441	88/15848	0.917125	0.965059	0.933485	Soc_19G00		1
milin vs zangga	GO:0003700	DNA-bind	9/441	476/15848	0.917241	0.965059	0.933485	Soc_11G00		9
milin vs zangga	GO:0006412	translation	1/441	91/15848	0.923889	0.967469	0.935817	Soc_18G00		1
milin vs zangga	GO:0003735	structural (	1/441	97/15848	0.935811	0.975352	0.943441	Soc_18G00		1
milin vs zangga	GO:0005975	carbohydr	1/441	107/15848	0.951683	0.984992	0.952767	Soc_15G00		1
milin vs zangga	GO:0004222	metalloend	1/441	109/15848	0.954353	0.984992	0.952767	Soc_10G00		1
milin vs zangga	GO:0005506	iron ion bi	1/441	114/15848	0.9604	0.984992	0.952767	Soc_18G00		1
milin vs zangga	GO:0043565	sequence-	2/441	191/15848	0.971163	0.984992	0.952767	Soc_5G000		2
milin vs zangga	GO:0055114	oxidation-	6/441	413/15848	0.975061	0.984992	0.952767	Soc_10G00		6
milin vs zangga	GO:0016787	hydrolase	1/441	134/15848	0.977581	0.984992	0.952767	Soc_6G000		1
milin vs zangga	GO:0000786	nucleosom	1/441	137/15848	0.979416	0.984992	0.952767	Soc_3G001		1
milin vs zangga	GO:0007264	small GTPa	1/441	139/15848	0.980556	0.984992	0.952767	Soc_10G00		1
milin vs zangga	GO:0005622	intracellula	3/441	400/15848	0.999119	0.999119	0.966431	Soc_10G00		3
linzhi vs zangga	GO:0000786	nucleosom	19/457	137/15848	1.44E-08	3.09E-06	2.92E-06	Soc_20G00		19
linzhi vs zangga	GO:0004415	hyalurono	4/457	10/15848	0.000125	0.013356	0.012613	Soc_8G000		4
linzhi vs zangga	GO:0004866	endopepti	5/457	22/15848	0.000342	0.022425	0.021178	Soc_5G000		5
linzhi vs zangga	GO:0006334	nucleosom	7/457	48/15848	0.000419	0.022425	0.021178	Soc_20G00		7
linzhi vs zangga	GO:0045095	keratin fila	3/457	12/15848	0.004316	0.153928	0.145372	Soc_15G00		3
linzhi vs zangga	GO:0046982	protein he	3/457	12/15848	0.004316	0.153928	0.145372	Soc_7G000		3
linzhi vs zangga	GO:0022848	acetylcholi	4/457	25/15848	0.005337	0.163167	0.154098	Soc_4G000		4
linzhi vs zangga	GO:0016311	dephosph	8/457	98/15848	0.007363	0.19696	0.186012	Soc_11G00		8
linzhi vs zangga	GO:0045211	postsynap	4/457	31/15848	0.011592	0.275638	0.260317	Soc_4G000		4
linzhi vs zangga	GO:0005104	fibroblast (	3/457	20/15848	0.018856	0.391477	0.369718	Soc_10G00		3
linzhi vs zangga	GO:0006470	protein de	8/457	123/15848	0.026056	0.391477	0.369718	Soc_11G00		8
linzhi vs zangga	GO:0008543	fibroblast (	3/457	23/15848	0.027493	0.391477	0.369718	Soc_10G00		3
linzhi vs zangga	GO:0004177	aminopep	2/457	10/15848	0.032033	0.391477	0.369718	Soc_24G00		2
linzhi vs zangga	GO:0004571	mannosyl-	2/457	10/15848	0.032033	0.391477	0.369718	Soc_6G001		2
linzhi vs zangga	GO:0005764	lysosome	2/457	10/15848	0.032033	0.391477	0.369718	Soc_11G00		2
linzhi vs zangga	GO:0016712	oxidoredu	2/457	10/15848	0.032033	0.391477	0.369718	Soc_21G00		2
linzhi vs zangga	GO:0050662	obsolete c	2/457	10/15848	0.032033	0.391477	0.369718	Soc_12G00		2
linzhi vs zangga	GO:0016791	phosphata	7/457	106/15848	0.033662	0.391477	0.369718	Soc_11G00		7
linzhi vs zangga	GO:0005789	endoplasm	3/457	26/15848	0.037894	0.391477	0.369718	Soc_24G00		3
linzhi vs zangga	GO:0007160	cell-matrix	3/457	26/15848	0.037894	0.391477	0.369718	Soc_19G00		3
linzhi vs zangga	GO:0007188	adenylate	2/457	11/15848	0.038416	0.391477	0.369718	Soc_8G000		2
linzhi vs zangga	GO:0022857	transmem	6/457	88/15848	0.04163	0.404948	0.38244	Soc_12G00		6
linzhi vs zangga	GO:0008271	secondary	2/457	13/15848	0.052462	0.440379	0.415902	Soc_15G00		2
linzhi vs zangga	GO:0008272	sulfate tra	2/457	13/15848	0.052462	0.440379	0.415902	Soc_15G00		2
linzhi vs zangga	GO:0015116	sulfate tra	2/457	13/15848	0.052462	0.440379	0.415902	Soc_15G00		2

linzhi vs zangga	GO:0005230	extracellula	5/457	71/15848	0.05392	0.440379	0.415902	Soc_4G000	5
linzhi vs zangga	GO:0004725	protein tyr	4/457	50/15848	0.055562	0.440379	0.415902	Soc_12G0(	4
linzhi vs zangga	GO:0008168	methyltran	4/457	51/15848	0.058978	0.450758	0.425704	Soc_21G0(	4
linzhi vs zangga	GO:0008138	protein tyr	4/457	58/15848	0.085895	0.607748	0.573967	Soc_11G0(	4
linzhi vs zangga	GO:0034220	ion transm	5/457	82/15848	0.088012	0.607748	0.573967	Soc_4G000	5
linzhi vs zangga	GO:0005975	carbohydr	6/457	107/15848	0.089093	0.607748	0.573967	Soc_10G0(	6
linzhi vs zangga	GO:0008083	growth fac	6/457	108/15848	0.092175	0.607748	0.573967	Soc_10G0(	6
linzhi vs zangga	GO:0061630	ubiquitin p	2/457	18/15848	0.093718	0.607748	0.573967	Soc_11G0(	2
linzhi vs zangga	GO:0005201	extracellula	2/457	19/15848	0.102818	0.642875	0.607142	Soc_4G000	2
linzhi vs zangga	GO:0004888	transmeml	8/457	165/15848	0.105143	0.642875	0.607142	Soc_21G0(	8
linzhi vs zangga	GO:0004114	3',5'-cyclic	2/457	20/15848	0.112147	0.648633	0.61258	Soc_1G002	2
linzhi vs zangga	GO:0006954	inflammato	2/457	20/15848	0.112147	0.648633	0.61258	Soc_19G0(	2
linzhi vs zangga	GO:0007015	actin filam	2/457	21/15848	0.121685	0.673089	0.635676	Soc_18G0(	2
linzhi vs zangga	GO:0005044	scavenger	4/457	66/15848	0.122666	0.673089	0.635676	Soc_12G0(	4
linzhi vs zangga	GO:0005856	cytoskelet	5/457	93/15848	0.130939	0.685903	0.647779	Soc_11G0(	5
linzhi vs zangga	GO:0005794	Golgi appa	2/457	22/15848	0.131411	0.685903	0.647779	Soc_13G0(	2
linzhi vs zangga	GO:0004129	cytochrom	2/457	23/15848	0.141308	0.72	0.67998	Soc_5G000	2
linzhi vs zangga	GO:0004197	cysteine-ty	2/457	26/15848	0.171847	0.734299	0.693485	Soc_8G000	2
linzhi vs zangga	GO:0009966	regulation	2/457	26/15848	0.171847	0.734299	0.693485	Soc_11G0(	2
linzhi vs zangga	GO:0009607	response t	2/457	27/15848	0.182255	0.734299	0.693485	Soc_12G0(	2
linzhi vs zangga	GO:0015078	proton tra	2/457	27/15848	0.182255	0.734299	0.693485	Soc_10G0(	2
linzhi vs zangga	GO:0015991	proton tra	2/457	27/15848	0.182255	0.734299	0.693485	Soc_10G0(	2
linzhi vs zangga	GO:0036459	thiol-depe	3/457	52/15848	0.188932	0.734299	0.693485	Soc_11G0(	3
linzhi vs zangga	GO:0043547	positive re	2/457	28/15848	0.192752	0.734299	0.693485	Soc_2G000	2
linzhi vs zangga	GO:0006351	transcriptio	3/457	53/15848	0.196299	0.734299	0.693485	Soc_15G0(	3
linzhi vs zangga	GO:0004812	aminoacyl	2/457	29/15848	0.203325	0.734299	0.693485	Soc_10G0(	2
linzhi vs zangga	GO:0006096	glycolytic	2/457	29/15848	0.203325	0.734299	0.693485	Soc_9G000	2
linzhi vs zangga	GO:0007601	visual perc	3/457	54/15848	0.203736	0.734299	0.693485	Soc_1G002	3
linzhi vs zangga	GO:0019001	guanyl nuc	2/457	30/15848	0.213961	0.734299	0.693485	Soc_8G000	2
linzhi vs zangga	GO:0031683	G-protein	2/457	30/15848	0.213961	0.734299	0.693485	Soc_8G000	2
linzhi vs zangga	GO:0003700	DNA-bind	17/457	476/15848	0.215124	0.734299	0.693485	Soc_10G0(	17
linzhi vs zangga	GO:0005882	intermedia	4/457	87/15848	0.242421	0.734299	0.693485	Soc_15G0(	4
linzhi vs zangga	GO:0008289	lipid bindi	4/457	88/15848	0.248709	0.734299	0.693485	Soc_15G0(	4
linzhi vs zangga	GO:0006915	apoptotic	3/457	60/15848	0.249536	0.734299	0.693485	Soc_11G0(	3
linzhi vs zangga	GO:0003956	NAD(P)+p	1/457	10/15848	0.253745	0.734299	0.693485	Soc_20G0(	1
linzhi vs zangga	GO:0004957	prostaglan	1/457	10/15848	0.253745	0.734299	0.693485	Soc_1G001	1
linzhi vs zangga	GO:0005741	mitochond	1/457	10/15848	0.253745	0.734299	0.693485	Soc_18G0(	1
linzhi vs zangga	GO:0050839	cell adhesi	1/457	10/15848	0.253745	0.734299	0.693485	Soc_7G000	1

linzhi vs zangga	GO:0003707	steroid ho	3/457	61/15848	0.257324	0.734299	0.693485	Soc_10G00		3
linzhi vs zangga	GO:0043401	steroid ho	3/457	61/15848	0.257324	0.734299	0.693485	Soc_10G00		3
linzhi vs zangga	GO:0016579	protein de	3/457	63/15848	0.272993	0.734299	0.693485	Soc_11G00		3
linzhi vs zangga	GO:0003725	double-str	1/457	11/15848	0.275278	0.734299	0.693485	Soc_1G001		1
linzhi vs zangga	GO:0005212	structural	1/457	11/15848	0.275278	0.734299	0.693485	Soc_21G00		1
linzhi vs zangga	GO:0006471	protein AD	1/457	11/15848	0.275278	0.734299	0.693485	Soc_20G00		1
linzhi vs zangga	GO:0006888	endoplasm	1/457	11/15848	0.275278	0.734299	0.693485	Soc_7G000		1
linzhi vs zangga	GO:0007034	vacuolar tr	1/457	11/15848	0.275278	0.734299	0.693485	Soc_10G00		1
linzhi vs zangga	GO:0007224	smoothene	1/457	11/15848	0.275278	0.734299	0.693485	Soc_14G00		1
linzhi vs zangga	GO:0016773	phosphotr	1/457	11/15848	0.275278	0.734299	0.693485	Soc_22G00		1
linzhi vs zangga	GO:0044212	transcriptio	1/457	11/15848	0.275278	0.734299	0.693485	Soc_11G00		1
linzhi vs zangga	GO:0008373	sialyltransf	2/457	36/15848	0.278443	0.734299	0.693485	Soc_24G00		2
linzhi vs zangga	GO:0006813	potassium	5/457	125/15848	0.292976	0.734299	0.693485	Soc_11G00		5
linzhi vs zangga	GO:0000139	Golgi mem	1/457	12/15848	0.296191	0.734299	0.693485	Soc_12G00		1
linzhi vs zangga	GO:0004707	MAP kinas	1/457	12/15848	0.296191	0.734299	0.693485	Soc_15G00		1
linzhi vs zangga	GO:0019752	carboxylic	1/457	12/15848	0.296191	0.734299	0.693485	Soc_11G00		1
linzhi vs zangga	GO:0030145	manganes	1/457	12/15848	0.296191	0.734299	0.693485	Soc_8G000		1
linzhi vs zangga	GO:0043039	tRNA amir	1/457	12/15848	0.296191	0.734299	0.693485	Soc_1G001		1
linzhi vs zangga	GO:0043066	negative re	1/457	12/15848	0.296191	0.734299	0.693485	Soc_8G000		1
linzhi vs zangga	GO:0008017	microtubu	4/457	96/15848	0.300064	0.734299	0.693485	Soc_20G00		4
linzhi vs zangga	GO:0006357	regulation	4/457	97/15848	0.306582	0.734299	0.693485	Soc_13G00		4
linzhi vs zangga	GO:0043565	sequence-	7/457	191/15848	0.313525	0.734299	0.693485	Soc_10G00		7
linzhi vs zangga	GO:0005911	cell-cell ju	1/457	13/15848	0.316502	0.734299	0.693485	Soc_7G000		1
linzhi vs zangga	GO:0007049	cell cycle	2/457	40/15848	0.321348	0.734299	0.693485	Soc_1G002		2
linzhi vs zangga	GO:0007156	homophili	5/457	131/15848	0.326943	0.734299	0.693485	Soc_10G00		5
linzhi vs zangga	GO:0005216	ion channe	9/457	258/15848	0.327969	0.734299	0.693485	Soc_11G00		9
linzhi vs zangga	GO:0016459	myosin co	3/457	70/15848	0.328393	0.734299	0.693485	Soc_10G00		3
linzhi vs zangga	GO:0003743	translation	2/457	41/15848	0.331989	0.734299	0.693485	Soc_10G00		2
linzhi vs zangga	GO:0006508	proteolysis	14/457	422/15848	0.333833	0.734299	0.693485	Soc_17G00		14
linzhi vs zangga	GO:0003712	transcriptio	1/457	14/15848	0.336228	0.734299	0.693485	Soc_21G00		1
linzhi vs zangga	GO:0016477	cell migrat	1/457	14/15848	0.336228	0.734299	0.693485	Soc_15G00		1
linzhi vs zangga	GO:0060271	cilium asse	1/457	14/15848	0.336228	0.734299	0.693485	Soc_6G000		1
linzhi vs zangga	GO:0003774	motor acti	3/457	71/15848	0.336329	0.734299	0.693485	Soc_10G00		3
linzhi vs zangga	GO:0016887	ATPase ac	3/457	71/15848	0.336329	0.734299	0.693485	Soc_15G00		3
linzhi vs zangga	GO:0004869	cysteine-ty	1/457	15/15848	0.355385	0.734299	0.693485	Soc_5G000		1
linzhi vs zangga	GO:0005813	centrosom	1/457	15/15848	0.355385	0.734299	0.693485	Soc_17G00		1
linzhi vs zangga	GO:0006364	rRNA proc	1/457	15/15848	0.355385	0.734299	0.693485	Soc_5G000		1
linzhi vs zangga	GO:0008033	tRNA proc	1/457	15/15848	0.355385	0.734299	0.693485	Soc_24G00		1

linzhi vs zangga	GO:0019905	syntaxin bi	1/457	15/15848	0.355385	0.734299	0.693485	Soc_12G0(	1
linzhi vs zangga	GO:0033179	proton-tra	1/457	15/15848	0.355385	0.734299	0.693485	Soc_10G0(	1
linzhi vs zangga	GO:0016705	oxidoredu	3/457	74/15848	0.360094	0.734299	0.693485	Soc_21G0(	3
linzhi vs zangga	GO:0016758	transferase	2/457	44/15848	0.363598	0.734299	0.693485	Soc_21G0(	2
linzhi vs zangga	GO:0005622	intracellula	13/457	400/15848	0.369446	0.734299	0.693485	Soc_11G0(	13
linzhi vs zangga	GO:0007596	blood coa	1/457	16/15848	0.373991	0.734299	0.693485	Soc_20G0(	1
linzhi vs zangga	GO:0042803	protein ho	1/457	16/15848	0.373991	0.734299	0.693485	Soc_7G00(	1
linzhi vs zangga	GO:0030036	actin cytos	2/457	45/15848	0.374012	0.734299	0.693485	Soc_15G0(	2
linzhi vs zangga	GO:0004879	nuclear re	1/457	17/15848	0.392062	0.749203	0.70756	Soc_11G0(	1
linzhi vs zangga	GO:0006820	anion tran	1/457	17/15848	0.392062	0.749203	0.70756	Soc_18G0(	1
linzhi vs zangga	GO:0016757	transferase	2/457	47/15848	0.394625	0.749203	0.70756	Soc_15G0(	2
linzhi vs zangga	GO:0004252	serine-typ	5/457	143/15848	0.395723	0.749203	0.70756	Soc_17G0(	5
linzhi vs zangga	GO:0046872	metal ion I	7/457	210/15848	0.402783	0.749203	0.70756	Soc_10G0(	7
linzhi vs zangga	GO:0031012	extracellula	2/457	48/15848	0.404815	0.749203	0.70756	Soc_19G0(	2
linzhi vs zangga	GO:0003684	damaged	1/457	18/15848	0.409611	0.749203	0.70756	Soc_5G000	1
linzhi vs zangga	GO:0004190	aspartic-ty	1/457	18/15848	0.409611	0.749203	0.70756	Soc_3G000	1
linzhi vs zangga	GO:0004497	monooxyg	1/457	19/15848	0.426655	0.752564	0.710734	Soc_4G000	1
linzhi vs zangga	GO:0016627	oxidoredu	1/457	19/15848	0.426655	0.752564	0.710734	Soc_1G001	1
linzhi vs zangga	GO:0006811	ion transp	9/457	285/15848	0.438585	0.752564	0.710734	Soc_11G0(	9
linzhi vs zangga	GO:0005507	copper ior	1/457	20/15848	0.443208	0.752564	0.710734	Soc_22G0(	1
linzhi vs zangga	GO:0006352	DNA-temp	1/457	20/15848	0.443208	0.752564	0.710734	Soc_9G000	1
linzhi vs zangga	GO:0006413	translation	1/457	20/15848	0.443208	0.752564	0.710734	Soc_10G0(	1
linzhi vs zangga	GO:0008234	cysteine-ty	2/457	52/15848	0.444712	0.752564	0.710734	Soc_24G0(	2
linzhi vs zangga	GO:0007166	cell surface	3/457	86/15848	0.453155	0.752564	0.710734	Soc_21G0(	3
linzhi vs zangga	GO:0035091	phosphati	2/457	53/15848	0.454454	0.752564	0.710734	Soc_11G0(	2
linzhi vs zangga	GO:0005242	inward rec	1/457	21/15848	0.459284	0.752564	0.710734	Soc_6G000	1
linzhi vs zangga	GO:0005540	hyaluronic	1/457	21/15848	0.459284	0.752564	0.710734	Soc_7G000	1
linzhi vs zangga	GO:0006914	autophagy	1/457	21/15848	0.459284	0.752564	0.710734	Soc_8G000	1
linzhi vs zangga	GO:0045893	positive re	1/457	21/15848	0.459284	0.752564	0.710734	Soc_8G000	1
linzhi vs zangga	GO:0006486	protein gly	3/457	87/15848	0.460681	0.752564	0.710734	Soc_10G0(	3
linzhi vs zangga	GO:0003899	DNA-direc	1/457	22/15848	0.474897	0.758418	0.716263	Soc_6G000	1
linzhi vs zangga	GO:0006418	tRNA amir	1/457	22/15848	0.474897	0.758418	0.716263	Soc_10G0(	1
linzhi vs zangga	GO:0045892	negative re	1/457	22/15848	0.474897	0.758418	0.716263	Soc_8G000	1
linzhi vs zangga	GO:0030001	metal ion t	1/457	23/15848	0.490061	0.771535	0.728651	Soc_10G0(	1
linzhi vs zangga	GO:0020037	heme binc	3/457	91/15848	0.490321	0.771535	0.728651	Soc_21G0(	3
linzhi vs zangga	GO:0008378	galactosylt	1/457	24/15848	0.504787	0.782785	0.739276	Soc_10G0(	1
linzhi vs zangga	GO:0050660	flavin ader	1/457	24/15848	0.504787	0.782785	0.739276	Soc_1G001	1
linzhi vs zangga	GO:0003713	transcriptic	1/457	25/15848	0.519089	0.787837	0.744046	Soc_9G000	1

linzhi vs zangga	GO:0007010	cytoskeleto	1/457	25/15848	0.519089	0.787837	0.744046	Soc_15G00	1
linzhi vs zangga	GO:0008081	phosphori	1/457	25/15848	0.519089	0.787837	0.744046	Soc_7G000	1
linzhi vs zangga	GO:0005200	structural	1/457	27/15848	0.546468	0.798016	0.75366	Soc_1G001	1
linzhi vs zangga	GO:0005328	neurotrans	1/457	27/15848	0.546468	0.798016	0.75366	Soc_13G00	1
linzhi vs zangga	GO:0003950	NAD ADP-	1/457	28/15848	0.559568	0.798016	0.75366	Soc_11G00	1
linzhi vs zangga	GO:0008285	negative re	1/457	28/15848	0.559568	0.798016	0.75366	Soc_11G00	1
linzhi vs zangga	GO:0007018	microtubu	2/457	66/15848	0.571437	0.798016	0.75366	Soc_20G00	2
linzhi vs zangga	GO:0005234	extracellul	1/457	29/15848	0.572291	0.798016	0.75366	Soc_10G00	1
linzhi vs zangga	GO:0006397	mRNA pro	1/457	29/15848	0.572291	0.798016	0.75366	Soc_5G000	1
linzhi vs zangga	GO:0003777	microtubu	2/457	67/15848	0.579648	0.798016	0.75366	Soc_20G00	2
linzhi vs zangga	GO:0005887	integral co	4/457	141/15848	0.583208	0.798016	0.75366	Soc_14G00	4
linzhi vs zangga	GO:0005578	extracellul	1/457	30/15848	0.584647	0.798016	0.75366	Soc_17G00	1
linzhi vs zangga	GO:0006464	cellular pro	1/457	30/15848	0.584647	0.798016	0.75366	Soc_8G000	1
linzhi vs zangga	GO:0030246	carbohydr	1/457	30/15848	0.584647	0.798016	0.75366	Soc_1G001	1
linzhi vs zangga	GO:0003824	catalytic ac	5/457	178/15848	0.586419	0.798016	0.75366	Soc_12G00	5
linzhi vs zangga	GO:0004842	ubiquitin-	3/457	105/15848	0.587103	0.798016	0.75366	Soc_11G00	3
linzhi vs zangga	GO:0008237	metallope	2/457	69/15848	0.595721	0.798016	0.75366	Soc_19G00	2
linzhi vs zangga	GO:0005245	voltage-ga	1/457	31/15848	0.596647	0.798016	0.75366	Soc_11G00	1
linzhi vs zangga	GO:0006897	endocytos	1/457	31/15848	0.596647	0.798016	0.75366	Soc_11G00	1
linzhi vs zangga	GO:0016702	oxidoredu	1/457	31/15848	0.596647	0.798016	0.75366	Soc_1G000	1
linzhi vs zangga	GO:0071805	potassium	1/457	31/15848	0.596647	0.798016	0.75366	Soc_14G00	1
linzhi vs zangga	GO:0007017	microtubu	1/457	32/15848	0.608302	0.798629	0.754239	Soc_1G001	1
linzhi vs zangga	GO:0008092	cytoskelet	1/457	32/15848	0.608302	0.798629	0.754239	Soc_6G000	1
linzhi vs zangga	GO:0042157	lipoprotein	1/457	32/15848	0.608302	0.798629	0.754239	Soc_1G000	1
linzhi vs zangga	GO:0004674	protein se	3/457	110/15848	0.618693	0.798787	0.754388	Soc_5G000	3
linzhi vs zangga	GO:0004970	ionotropic	1/457	33/15848	0.61962	0.798787	0.754388	Soc_10G00	1
linzhi vs zangga	GO:0016616	oxidoredu	1/457	33/15848	0.61962	0.798787	0.754388	Soc_20G00	1
linzhi vs zangga	GO:0005891	voltage-ga	1/457	34/15848	0.630611	0.806091	0.761286	Soc_11G00	1
linzhi vs zangga	GO:0005506	iron ion bi	3/457	114/15848	0.642759	0.806091	0.761286	Soc_21G00	3
linzhi vs zangga	GO:0006886	intracellula	3/457	115/15848	0.648606	0.806091	0.761286	Soc_13G00	3
linzhi vs zangga	GO:0055114	oxidation-	11/457	413/15848	0.648868	0.806091	0.761286	Soc_1G000	11
linzhi vs zangga	GO:0006836	neurotrans	1/457	36/15848	0.651653	0.806091	0.761286	Soc_13G00	1
linzhi vs zangga	GO:0008305	integrin co	1/457	36/15848	0.651653	0.806091	0.761286	Soc_1G001	1
linzhi vs zangga	GO:0015031	protein tra	1/457	36/15848	0.651653	0.806091	0.761286	Soc_24G00	1
linzhi vs zangga	GO:0007399	nervous sy	1/457	37/15848	0.661721	0.809933	0.764915	Soc_21G00	1
linzhi vs zangga	GO:0004871	obsolete s	2/457	78/15848	0.662328	0.809933	0.764915	Soc_8G000	2
linzhi vs zangga	GO:0005249	voltage-ga	2/457	79/15848	0.669158	0.813636	0.768411	Soc_14G00	2
linzhi vs zangga	GO:0005783	endoplasm	1/457	39/15848	0.680994	0.823349	0.777585	Soc_13G00	1

linzhi vs zangga	GO:0005737	cytoplasm	6/457	238/15848	0.686947	0.82588	0.779975	Soc_11G0(	6
linzhi vs zangga	GO:0003723	RNA bindi	5/457	202/15848	0.696265	0.829567	0.783457	Soc_10G0(	5
linzhi vs zangga	GO:0030170	pyridoxal p	1/457	41/15848	0.699171	0.829567	0.783457	Soc_11G0(	1
linzhi vs zangga	GO:0005089	Rho guany	2/457	84/15848	0.701643	0.829567	0.783457	Soc_15G0(	2
linzhi vs zangga	GO:0000166	nucleotide	1/457	43/15848	0.716315	0.842261	0.795445	Soc_10G0(	1
linzhi vs zangga	GO:0035023	regulation	2/457	88/15848	0.725687	0.848617	0.801449	Soc_15G0(	2
linzhi vs zangga	GO:0005874	microtubu	1/457	45/15848	0.732484	0.851911	0.804559	Soc_1G001	1
linzhi vs zangga	GO:0006869	lipid trans	1/457	47/15848	0.747733	0.864945	0.816869	Soc_1G00(	1
linzhi vs zangga	GO:0015074	DNA integ	2/457	94/15848	0.758666	0.87209	0.823617	Soc_10G0(	2
linzhi vs zangga	GO:0016491	oxidoredu	4/457	179/15848	0.762696	0.87209	0.823617	Soc_21G0(	4
linzhi vs zangga	GO:0009058	biosynthet	1/457	50/15848	0.768996	0.87209	0.823617	Soc_4G001	1
linzhi vs zangga	GO:0005198	structural	4/457	181/15848	0.77021	0.87209	0.823617	Soc_15G0(	4
linzhi vs zangga	GO:0005102	signaling r	1/457	52/15848	0.782168	0.880196	0.831273	Soc_21G0(	1
linzhi vs zangga	GO:0005576	extracellul	7/457	311/15848	0.796983	0.880196	0.831273	Soc_10G0(	7
linzhi vs zangga	GO:0007165	signal tran	8/457	351/15848	0.797509	0.880196	0.831273	Soc_11G0(	8
linzhi vs zangga	GO:0070588	calcium io	1/457	55/15848	0.800534	0.880196	0.831273	Soc_11G0(	1
linzhi vs zangga	GO:0006396	RNA proce	1/457	56/15848	0.806306	0.880196	0.831273	Soc_18G0(	1
linzhi vs zangga	GO:0016301	kinase acti	1/457	56/15848	0.806306	0.880196	0.831273	Soc_3G00(	1
linzhi vs zangga	GO:0007155	cell adhesi	3/457	149/15848	0.807778	0.880196	0.831273	Soc_1G00(	3
linzhi vs zangga	GO:0003779	actin bindi	2/457	105/15848	0.810274	0.880196	0.831273	Soc_15G0(	2
linzhi vs zangga	GO:0005085	guanyl-nu	1/457	58/15848	0.817354	0.883403	0.834301	Soc_5G00(	1
linzhi vs zangga	GO:0004222	metalloend	2/457	109/15848	0.826459	0.888755	0.839355	Soc_19G0(	2
linzhi vs zangga	GO:0008076	voltage-ga	1/457	61/15848	0.832759	0.891052	0.841525	Soc_8G00(	1
linzhi vs zangga	GO:0016567	protein ub	1/457	64/15848	0.846868	0.90164	0.851524	Soc_11G0(	1
linzhi vs zangga	GO:0008146	sulfotransf	1/457	66/15848	0.855607	0.906378	0.855998	Soc_11G0(	1
linzhi vs zangga	GO:0051015	actin filam	1/457	67/15848	0.859788	0.906378	0.855998	Soc_1G00(	1
linzhi vs zangga	GO:0051260	protein ho	1/457	69/15848	0.867791	0.91033	0.859732	Soc_8G00(	1
linzhi vs zangga	GO:0005179	hormone a	1/457	72/15848	0.87895	0.917538	0.866539	Soc_13G0(	1
linzhi vs zangga	GO:0008152	metabolic	4/457	226/15848	0.894487	0.929225	0.877576	Soc_20G0(	4
linzhi vs zangga	GO:0016787	hydrolase	2/457	134/15848	0.90227	0.930409	0.878694	Soc_11G0(	2
linzhi vs zangga	GO:0007275	multicellul	1/457	80/15848	0.904322	0.930409	0.878694	Soc_11G0(	1
linzhi vs zangga	GO:0016192	vesicle-me	1/457	82/15848	0.909788	0.930531	0.87881	Soc_12G0(	1
linzhi vs zangga	GO:0007264	small GTPa	2/457	139/15848	0.913138	0.930531	0.87881	Soc_24G0(	2
linzhi vs zangga	GO:0005096	GTPase ac	1/457	88/15848	0.924386	0.933107	0.881242	Soc_2G00(	1
linzhi vs zangga	GO:0006281	DNA repai	1/457	88/15848	0.924386	0.933107	0.881242	Soc_13G0(	1
linzhi vs zangga	GO:0003924	GTPase ac	5/457	314/15848	0.950834	0.955298	0.9022	Soc_13G0(	5
linzhi vs zangga	GO:0046983	protein dir	3/457	236/15848	0.968357	0.968357	0.914533	Soc_15G0(	3
