# Supplementary material for: Population structure and adaptability analysis of Schizothorax o’connori based on whole-genome resequencing
Source: BMC Genomics. 2024 Feb 6;25:145. doi: 10.1186/s12864-024-09975-9 (PMC10845765; doi:10.1186/s12864-024-09975-9)
Supplement: Supplementary file 5 — Additional file 5: Supplementary Table 5. KEGG annotation for XP-EHH analysis. [file 12864_2024_9975_MOESM5_ESM.pdf]

Supplementary Table 5. KEGG annotation for XP-EHH analysis

population	ID	Descriptio	GeneRatio	BgRatio	pvalue	p.adjust	qvalue
bomi vs milin	ko04932	Non-alcoh	14/222	203/8136	0.001287	0.129138	0.122044
bomi vs milin	ko04622	RIG-I-like	7/222	62/8136	0.001407	0.129138	0.122044
bomi vs milin	ko04920	Adipocyto	9/222	101/8136	0.001688	0.129138	0.122044
bomi vs milin	ko04530	Tight junct	16/222	259/8136	0.001885	0.129138	0.122044
bomi vs milin	ko04520	Adherens	10/222	135/8136	0.003759	0.206017	0.194699
bomi vs milin	ko00380	Tryptopha	5/222	41/8136	0.004867	0.211496	0.199877
bomi vs milin	ko04621	NOD-like	11/222	165/8136	0.005403	0.211496	0.199877
bomi vs milin	ko04750	Inflammat	10/222	146/8136	0.006522	0.223368	0.211097
bomi vs milin	ko04931	Insulin resi	10/222	149/8136	0.007497	0.228255	0.215716
bomi vs milin	ko03320	PPAR sign	7/222	87/8136	0.009433	0.247572	0.233971
bomi vs milin	ko04912	GnRH sign	9/222	132/8136	0.009939	0.247572	0.233971
bomi vs milin	ko00280	Valine, leu	5/222	51/8136	0.012252	0.257805	0.243642
bomi vs milin	ko04936	NA	9/222	138/8136	0.013059	0.257805	0.243642
bomi vs milin	ko00592	alpha-Linc	4/222	34/8136	0.013173	0.257805	0.243642
bomi vs milin	ko00591	Linoleic ac	4/222	36/8136	0.01604	0.268143	0.253412
bomi vs milin	ko05160	Hepatitis C	12/222	218/8136	0.016101	0.268143	0.253412
bomi vs milin	ko04217	Necroptos	12/222	219/8136	0.016637	0.268143	0.253412
bomi vs milin	ko00120	Primary bi	3/222	23/8136	0.023732	0.361261	0.341415
bomi vs milin	ko04120	Ubiquitin r	9/222	156/8136	0.026777	0.386148	0.364934
bomi vs milin	ko04623	Cytosolic D	4/222	45/8136	0.033594	0.431388	0.407689
bomi vs milin	ko03450	Non-hom	2/222	11/8136	0.034662	0.431388	0.407689
bomi vs milin	ko00512	Mucin type	4/222	46/8136	0.036033	0.431388	0.407689
bomi vs milin	ko00650	Butanoate	3/222	27/8136	0.036211	0.431388	0.407689
bomi vs milin	ko00900	Terpenoid	3/222	28/8136	0.039764	0.441201	0.416963
bomi vs milin	ko04068	FoxO signa	9/222	168/8136	0.040256	0.441201	0.416963
bomi vs milin	ko05208	NA	12/222	256/8136	0.047419	0.47246	0.446505
bomi vs milin	ko00450	Selenocom	2/222	13/8136	0.047438	0.47246	0.446505
bomi vs milin	ko00565	Ether lipid	4/222	51/8136	0.049732	0.47246	0.446505
bomi vs milin	ko05415	NA	11/222	230/8136	0.050005	0.47246	0.446505
bomi vs milin	ko04620	Toll-like re	6/222	100/8136	0.055334	0.479378	0.453043
bomi vs milin	ko00514	Other type	4/222	53/8136	0.055909	0.479378	0.453043
bomi vs milin	ko03013	RNA trans	6/222	101/8136	0.057552	0.479378	0.453043
bomi vs milin	ko04611	Platelet ac	9/222	180/8136	0.057735	0.479378	0.453043
bomi vs milin	ko05161	Hepatitis B	9/222	182/8136	0.061061	0.492077	0.465044
bomi vs milin	ko00564	Glyceroph	6/222	104/8136	0.064525	0.505135	0.477385
bomi vs milin	ko00590	Arachidon	5/222	80/8136	0.067017	0.510075	0.482053
bomi vs milin	ko04061	Viral prote	5/222	84/8136	0.079032	0.585265	0.553113
bomi vs milin	ko04913	Ovarian st	5/222	85/8136	0.082205	0.592744	0.560181
bomi vs milin	ko04922	Glucagon	7/222	143/8136	0.095971	0.657419	0.621303
bomi vs milin	ko04930	Type II dia	4/222	64/8136	0.096753	0.657419	0.621303
bomi vs milin	ko04921	Oxytocin s	10/222	232/8136	0.102466	0.657419	0.621303
bomi vs milin	ko04213	Longevity	5/222	91/8136	0.102632	0.657419	0.621303
bomi vs milin	ko04935	Growth ho	8/222	174/8136	0.103172	0.657419	0.621303
bomi vs milin	ko04260	Cardiac m	7/222	149/8136	0.112786	0.690376	0.652449
bomi vs milin	ko04514	Cell adhes	11/222	267/8136	0.113383	0.690376	0.652449
bomi vs milin	ko04211	Longevity	6/222	122/8136	0.116224	0.692289	0.654257
bomi vs milin	ko05133	Pertussis	5/222	96/8136	0.1214	0.699226	0.660812
bomi vs milin	ko05164	Influenza A	9/222	211/8136	0.12298	0.699226	0.660812
bomi vs milin	ko05012	Parkinson	12/222	303/8136	0.125044	0.699226	0.660812
bomi vs milin	ko00604	Glycosphir	2/222	23/8136	0.129102	0.707478	0.668612
bomi vs milin	ko05171	NA	10/222	246/8136	0.135326	0.727043	0.687102
bomi vs milin	ko00190	Oxidative	6/222	131/8136	0.148061	0.765447	0.723396
bomi vs milin	ko04668	TNF signal	6/222	131/8136	0.148061	0.765447	0.723396
bomi vs milin	ko03060	Protein ex	2/222	26/8136	0.15747	0.799012	0.755117
bomi vs milin	ko04975	Fat digesti	3/222	51/8136	0.161928	0.806698	0.762381
bomi vs milin	ko04971	Gastric aci	5/222	107/8136	0.167741	0.820732	0.775644

bomi vs milin	ko04940	Type I diak	4/222	80/8136	0.174233	0.837542	0.791531
bomi vs milin	ko04625	C-type lec	6/222	141/8136	0.187511	0.857139	0.810051
bomi vs milin	ko04972	Pancreatic	6/222	141/8136	0.187511	0.857139	0.810051
bomi vs milin	ko04270	Vascular sı	8/222	203/8136	0.190448	0.857139	0.810051
bomi vs milin	ko04910	Insulin sigı	7/222	173/8136	0.193801	0.857139	0.810051
bomi vs milin	ko05010	Alzheimer	16/222	462/8136	0.193951	0.857139	0.810051
bomi vs milin	ko04210	Apoptosis	8/222	205/8136	0.197393	0.857749	0.810627
bomi vs milin	ko04064	NF-kappa	5/222	114/8136	0.20035	0.857749	0.810627
bomi vs milin	ko00790	Folate bios	2/222	32/8136	0.216928	0.902186	0.852623
bomi vs milin	ko04659	Th17 cell d	6/222	148/8136	0.217315	0.902186	0.852623
bomi vs milin	ko05016	Huntingto	12/222	343/8136	0.226954	0.924608	0.873813
bomi vs milin	ko04714	Thermoge	9/222	246/8136	0.229465	0.924608	0.873813
bomi vs milin	ko04152	AMPK sigr	6/222	152/8136	0.235032	0.925383	0.874545
bomi vs milin	ko05417	NA	9/222	248/8136	0.236412	0.925383	0.874545
bomi vs milin	ko04657	IL-17 signa	4/222	92/8136	0.242516	0.935907	0.884491
bomi vs milin	ko05132	Salmonella	11/222	318/8136	0.251241	0.951298	0.899037
bomi vs milin	ko04927	Cortisol sy	4/222	96/8136	0.26653	0.951298	0.899037
bomi vs milin	ko04072	Phospholip	8/222	224/8136	0.268021	0.951298	0.899037
bomi vs milin	ko05332	Graft-vers	3/222	67/8136	0.276046	0.951298	0.899037
bomi vs milin	ko04726	Serotonerg	6/222	161/8136	0.276391	0.951298	0.899037
bomi vs milin	ko04512	ECM-recep	4/222	98/8136	0.278699	0.951298	0.899037
bomi vs milin	ko04062	Chemokin	8/222	227/8136	0.279817	0.951298	0.899037
bomi vs milin	ko00430	Taurine an	1/222	12/8136	0.282664	0.951298	0.899037
bomi vs milin	ko04370	VEGF signa	4/222	99/8136	0.284818	0.951298	0.899037
bomi vs milin	ko04380	Osteoclast	5/222	131/8136	0.28683	0.951298	0.899037
bomi vs milin	ko04010	MAPK sigr	14/222	431/8136	0.287979	0.951298	0.899037
bomi vs milin	ko05340	Primary im	2/222	39/8136	0.288167	0.951298	0.899037
bomi vs milin	ko04114	Oocyte me	5/222	134/8136	0.302827	0.987792	0.933526
bomi vs milin	ko04110	Cell cycle	5/222	135/8136	0.308191	0.993462	0.938885
bomi vs milin	ko05014	Amyotropl	13/222	406/8136	0.3157	0.997522	0.942722
bomi vs milin	ko00071	Fatty acid	2/222	43/8136	0.328701	0.997522	0.942722
bomi vs milin	ko04630	JAK-STAT	5/222	144/8136	0.35695	0.997522	0.942722
bomi vs milin	ko05168	Herpes sin	8/222	246/8136	0.357172	0.997522	0.942722
bomi vs milin	ko00730	Thiamine r	1/222	16/8136	0.357931	0.997522	0.942722
bomi vs milin	ko01523	Antifolate	2/222	46/8136	0.358699	0.997522	0.942722
bomi vs milin	ko03460	Fanconi ar	2/222	46/8136	0.358699	0.997522	0.942722
bomi vs milin	ko04540	Gap juncti	5/222	145/8136	0.362401	0.997522	0.942722
bomi vs milin	ko04015	Rap1 signa	10/222	317/8136	0.364877	0.997522	0.942722
bomi vs milin	ko00600	Sphingolip	3/222	79/8136	0.366096	0.997522	0.942722
bomi vs milin	ko05166	Human T-	9/222	284/8136	0.371288	0.997522	0.942722
bomi vs milin	ko05167	Kaposi sar	7/222	215/8136	0.371597	0.997522	0.942722
bomi vs milin	ko05017	Spinocerel	6/222	181/8136	0.372954	0.997522	0.942722
bomi vs milin	ko04730	Long-term	4/222	114/8136	0.377996	0.997522	0.942722
bomi vs milin	ko04914	Progestero	4/222	116/8136	0.390444	0.997522	0.942722
bomi vs milin	ko00910	Nitrogen r	1/222	18/8136	0.392561	0.997522	0.942722
bomi vs milin	ko04624	Toll and In	2/222	51/8136	0.407533	0.997522	0.942722
bomi vs milin	ko04664	Fc epsilon	4/222	119/8136	0.40905	0.997522	0.942722
bomi vs milin	ko04724	Glutamate	6/222	189/8136	0.412216	0.997522	0.942722
bomi vs milin	ko05330	Allograft r	3/222	86/8136	0.417991	0.997522	0.942722
bomi vs milin	ko04391	Hippo sigr	3/222	87/8136	0.425303	0.997522	0.942722
bomi vs milin	ko04610	Compleme	3/222	87/8136	0.425303	0.997522	0.942722
bomi vs milin	ko00340	Histidine n	1/222	20/8136	0.425332	0.997522	0.942722
bomi vs milin	ko05320	Autoimmu	3/222	88/8136	0.432583	0.997522	0.942722
bomi vs milin	ko02010	ABC transp	2/222	54/8136	0.435967	0.997522	0.942722
bomi vs milin	ko05231	Choline m	5/222	159/8136	0.438487	0.997522	0.942722
bomi vs milin	ko00563	Glycosylph	1/222	21/8136	0.441051	0.997522	0.942722
bomi vs milin	ko04722	Neurotrop	5/222	160/8136	0.443867	0.997522	0.942722
bomi vs milin	ko04371	Apelin sigr	6/222	196/8136	0.446394	0.997522	0.942722

bomi vs milin	ko04923	Regulation	3/222	90/8136	0.44704	0.997522	0.942722
bomi vs milin	ko04060	Cytokine-c	7/222	232/8136	0.448034	0.997522	0.942722
bomi vs milin	ko04962	Vasopressi	2/222	56/8136	0.454512	0.997522	0.942722
bomi vs milin	ko05143	African try	2/222	56/8136	0.454512	0.997522	0.942722
bomi vs milin	ko01210	2-Oxocarb	1/222	22/8136	0.456342	0.997522	0.942722
bomi vs milin	ko04212	Longevity	3/222	93/8136	0.468446	0.997522	0.942722
bomi vs milin	ko04740	Olfactory t	3/222	93/8136	0.468446	0.997522	0.942722
bomi vs milin	ko04014	Ras signali	9/222	309/8136	0.469357	0.997522	0.942722
bomi vs milin	ko04917	Prolactin s	3/222	94/8136	0.475501	0.997522	0.942722
bomi vs milin	ko04146	Peroxisom	3/222	96/8136	0.489479	0.997522	0.942722
bomi vs milin	ko00511	Other glyc	1/222	25/8136	0.499761	0.997522	0.942722
bomi vs milin	ko00020	Citrate cyc	1/222	26/8136	0.513453	0.997522	0.942722
bomi vs milin	ko03020	RNA polym	1/222	26/8136	0.513453	0.997522	0.942722
bomi vs milin	ko05169	Epstein-Ba	7/222	247/8136	0.514119	0.997522	0.942722
bomi vs milin	ko01212	Fatty acid	2/222	64/8136	0.525023	0.997522	0.942722
bomi vs milin	ko00640	Propanoat	1/222	27/8136	0.526771	0.997522	0.942722
bomi vs milin	ko04022	cGMP-PKC	7/222	252/8136	0.535564	0.997522	0.942722
bomi vs milin	ko04013	MAPK sign	3/222	103/8136	0.536908	0.997522	0.942722
bomi vs milin	ko04725	Cholinergi	5/222	178/8136	0.537873	0.997522	0.942722
bomi vs milin	ko00630	Glyoxylate	1/222	28/8136	0.539727	0.997522	0.942722
bomi vs milin	ko04670	Leukocyte	5/222	180/8136	0.547889	0.997522	0.942722
bomi vs milin	ko04919	Thyroid ho	5/222	180/8136	0.547889	0.997522	0.942722
bomi vs milin	ko00330	Arginine a	2/222	67/8136	0.549847	0.997522	0.942722
bomi vs milin	ko05135	Yersinia in	6/222	219/8136	0.554702	0.997522	0.942722
bomi vs milin	ko00601	Glycosphir	1/222	30/8136	0.564588	0.997522	0.942722
bomi vs milin	ko05020	Prion disea	9/222	335/8136	0.56789	0.997522	0.942722
bomi vs milin	ko04070	Phosphatid	3/222	111/8136	0.58793	0.997522	0.942722
bomi vs milin	ko04966	Collecting	1/222	32/8136	0.588112	0.997522	0.942722
bomi vs milin	ko00310	Lysine deg	2/222	72/8136	0.589168	0.997522	0.942722
bomi vs milin	ko04742	Taste trans	2/222	72/8136	0.589168	0.997522	0.942722
bomi vs milin	ko04974	Protein dig	3/222	112/8136	0.594052	0.997522	0.942722
bomi vs milin	ko00830	Retinol me	2/222	73/8136	0.596721	0.997522	0.942722
bomi vs milin	ko05162	Measles	4/222	152/8136	0.599801	0.997522	0.942722
bomi vs milin	ko04666	Fc gamma	4/222	153/8136	0.605004	0.997522	0.942722
bomi vs milin	ko05410	Hypertrop	4/222	154/8136	0.610166	0.997522	0.942722
bomi vs milin	ko04071	Sphingolip	5/222	193/8136	0.610315	0.997522	0.942722
bomi vs milin	ko04650	Natural kil	3/222	116/8136	0.617945	0.997522	0.942722
bomi vs milin	ko00240	Pyrimidine	2/222	76/8136	0.618756	0.997522	0.942722
bomi vs milin	ko05212	Pancreatic	3/222	118/8136	0.629531	0.997522	0.942722
bomi vs milin	ko00230	Purine me	4/222	158/8136	0.630397	0.997522	0.942722
bomi vs milin	ko04141	Protein pro	5/222	199/8136	0.637401	0.997522	0.942722
bomi vs milin	ko00350	Tyrosine m	1/222	37/8136	0.641533	0.997522	0.942722
bomi vs milin	ko04928	Parathyroi	4/222	161/8136	0.645127	0.997522	0.942722
bomi vs milin	ko04020	Calcium si	10/222	398/8136	0.652155	0.997522	0.942722
bomi vs milin	ko05206	MicroRNA	5/222	204/8136	0.659076	0.997522	0.942722
bomi vs milin	ko04918	Thyroid ho	2/222	82/8136	0.660035	0.997522	0.942722
bomi vs milin	ko04341	Hedgehog	1/222	39/8136	0.660917	0.997522	0.942722
bomi vs milin	ko04977	Vitamin di	1/222	39/8136	0.660917	0.997522	0.942722
bomi vs milin	ko04361	Axon rege	3/222	124/8136	0.662826	0.997522	0.942722
bomi vs milin	ko04723	Retrograde	5/222	206/8136	0.667511	0.997522	0.942722
bomi vs milin	ko04658	Th1 and Th	3/222	125/8136	0.668161	0.997522	0.942722
bomi vs milin	ko05202	Transcripti	6/222	247/8136	0.671785	0.997522	0.942722
bomi vs milin	ko05145	Toxoplasm	4/222	167/8136	0.673416	0.997522	0.942722
bomi vs milin	ko05215	Prostate ca	3/222	126/8136	0.673434	0.997522	0.942722
bomi vs milin	ko04710	Circadian r	1/222	41/8136	0.679257	0.997522	0.942722
bomi vs milin	ko05170	Human im	6/222	249/8136	0.679361	0.997522	0.942722
bomi vs milin	ko04310	Wnt signal	5/222	209/8136	0.679907	0.997522	0.942722
bomi vs milin	ko03015	mRNA sur	2/222	86/8136	0.685521	0.997522	0.942722

bomi vs milin	ko00260	Glycine, se	1/222	42/8136	0.688053	0.997522	0.942722
bomi vs milin	ko00620	Pyruvate n	1/222	42/8136	0.688053	0.997522	0.942722
bomi vs milin	ko03420	Nucleotide	1/222	42/8136	0.688053	0.997522	0.942722
bomi vs milin	ko05144	Malaria	1/222	42/8136	0.688053	0.997522	0.942722
bomi vs milin	ko04933	AGE-RAGE	3/222	130/8136	0.693912	0.997522	0.942722
bomi vs milin	ko00534	Glycosami	1/222	43/8136	0.696609	0.997522	0.942722
bomi vs milin	ko00983	Drug meta	2/222	89/8136	0.703596	0.997522	0.942722
bomi vs milin	ko00051	Fructose a	1/222	44/8136	0.704931	0.997522	0.942722
bomi vs milin	ko04215	Apoptosis	1/222	44/8136	0.704931	0.997522	0.942722
bomi vs milin	ko04976	Bile secret	3/222	133/8136	0.708629	0.997522	0.942722
bomi vs milin	ko05165	Human pa	10/222	417/8136	0.708731	0.997522	0.942722
bomi vs milin	ko04964	Proximal t	1/222	45/8136	0.713026	0.997522	0.942722
bomi vs milin	ko05414	Dilated ca	4/222	179/8136	0.725231	0.997522	0.942722
bomi vs milin	ko04925	Aldosteron	3/222	137/8136	0.727404	0.997522	0.942722
bomi vs milin	ko05418	Fluid shea	4/222	181/8136	0.733248	0.997522	0.942722
bomi vs milin	ko00513	Various ty	1/222	48/8136	0.736009	0.997522	0.942722
bomi vs milin	ko00760	Nicotinate	1/222	49/8136	0.743255	0.997522	0.942722
bomi vs milin	ko04960	Aldosteron	1/222	49/8136	0.743255	0.997522	0.942722
bomi vs milin	ko00270	Cysteine a	1/222	51/8136	0.757158	0.997522	0.942722
bomi vs milin	ko04151	PI3K-Akt s	10/222	437/8136	0.761682	0.997522	0.942722
bomi vs milin	ko05235	PD-L1 exp	2/222	101/8136	0.767447	0.997522	0.942722
bomi vs milin	ko04261	Adrenergic	5/222	236/8136	0.777424	0.997522	0.942722
bomi vs milin	ko04390	Hippo sign	4/222	196/8136	0.787858	0.997522	0.942722
bomi vs milin	ko03040	Spliceosor	3/222	152/8136	0.789474	0.997522	0.942722
bomi vs milin	ko04924	Renin secr	2/222	106/8136	0.790332	0.997522	0.942722
bomi vs milin	ko00510	N-Glycan	1/222	58/8136	0.800175	0.997522	0.942722
bomi vs milin	ko05220	Chronic m	2/222	109/8136	0.803094	0.997522	0.942722
bomi vs milin	ko05152	Tuberculos	5/222	245/8136	0.80438	0.997522	0.942722
bomi vs milin	ko04214	Apoptosis	1/222	59/8136	0.805667	0.997522	0.942722
bomi vs milin	ko04350	TGF-beta	2/222	110/8136	0.807193	0.997522	0.942722
bomi vs milin	ko05222	Small cell	2/222	110/8136	0.807193	0.997522	0.942722
bomi vs milin	ko05310	Asthma	1/222	62/8136	0.821256	0.997522	0.942722
bomi vs milin	ko04970	Salivary se	2/222	114/8136	0.822842	0.997522	0.942722
bomi vs milin	ko05100	Bacterial ir	2/222	115/8136	0.826572	0.997522	0.942722
bomi vs milin	ko03008	Ribosome	1/222	64/8136	0.830951	0.997522	0.942722
bomi vs milin	ko00480	Glutathion	1/222	66/8136	0.840122	0.997522	0.942722
bomi vs milin	ko04934	Cushing sy	4/222	215/8136	0.843854	0.997522	0.942722
bomi vs milin	ko01200	Carbon me	2/222	121/8136	0.847512	0.997522	0.942722
bomi vs milin	ko04024	cAMP sign	7/222	351/8136	0.850321	0.997522	0.942722
bomi vs milin	ko04961	Endocrine	1/222	69/8136	0.852959	0.997522	0.942722
bomi vs milin	ko04330	Notch sign	1/222	70/8136	0.857005	0.997522	0.942722
bomi vs milin	ko05030	Cocaine ad	1/222	70/8136	0.857005	0.997522	0.942722
bomi vs milin	ko05322	Systemic lu	3/222	174/8136	0.858985	0.997522	0.942722
bomi vs milin	ko04145	Phagosom	4/222	222/8136	0.861112	0.997522	0.942722
bomi vs milin	ko05221	Acute mye	1/222	73/8136	0.868492	0.997522	0.942722
bomi vs milin	ko05142	Chagas dis	2/222	128/8136	0.869032	0.997522	0.942722
bomi vs milin	ko05416	Viral myoc	2/222	128/8136	0.869032	0.997522	0.942722
bomi vs milin	ko04810	Regulation	6/222	317/8136	0.869909	0.997522	0.942722
bomi vs milin	ko04660	T cell rece	2/222	129/8136	0.871868	0.997522	0.942722
bomi vs milin	ko05412	Arrhythmo	2/222	129/8136	0.871868	0.997522	0.942722
bomi vs milin	ko01522	Endocrine	2/222	130/8136	0.874649	0.997522	0.942722
bomi vs milin	ko05032	Morphine	2/222	130/8136	0.874649	0.997522	0.942722
bomi vs milin	ko04115	p53 signal	1/222	75/8136	0.875634	0.997522	0.942722
bomi vs milin	ko05134	Legionello	1/222	75/8136	0.875634	0.997522	0.942722
bomi vs milin	ko04510	Focal adhe	5/222	276/8136	0.877966	0.997522	0.942722
bomi vs milin	ko05207	NA	5/222	276/8136	0.877966	0.997522	0.942722
bomi vs milin	ko05210	Colorectal	2/222	134/8136	0.885226	0.997522	0.942722
bomi vs milin	ko04360	Axon guid	5/222	281/8136	0.887324	0.997522	0.942722

bomi vs milin	ko04137	Mitophagy	1/222	79/8136	0.888781	0.997522	0.942722
bomi vs milin	ko01230	Biosynthes	1/222	80/8136	0.891845	0.997522	0.942722
bomi vs milin	ko04911	Insulin sec	2/222	137/8136	0.892612	0.997522	0.942722
bomi vs milin	ko01524	Platinum d	1/222	81/8136	0.894826	0.997522	0.942722
bomi vs milin	ko04728	Dopamine	3/222	193/8136	0.902004	0.997522	0.942722
bomi vs milin	ko04550	Signaling p	3/222	195/8136	0.905768	0.997522	0.942722
bomi vs milin	ko05163	Human cy	5/222	292/8136	0.905775	0.997522	0.942722
bomi vs milin	ko04613	NA	3/222	202/8136	0.917938	0.997522	0.942722
bomi vs milin	ko04720	Long-term	1/222	90/8136	0.918226	0.997522	0.942722
bomi vs milin	ko05321	Inflammat	1/222	90/8136	0.918226	0.997522	0.942722
bomi vs milin	ko03018	RNA degra	1/222	91/8136	0.920482	0.997522	0.942722
bomi vs milin	ko04672	Intestinal i	1/222	92/8136	0.922676	0.997522	0.942722
bomi vs milin	ko05217	Basal cell c	1/222	92/8136	0.922676	0.997522	0.942722
bomi vs milin	ko05230	Central ca	1/222	95/8136	0.928904	0.997522	0.942722
bomi vs milin	ko04926	Relaxin sig	2/222	157/8136	0.93163	0.997522	0.942722
bomi vs milin	ko05031	Amphetam	1/222	97/8136	0.932776	0.997522	0.942722
bomi vs milin	ko04929	GnRH secr	1/222	98/8136	0.934632	0.997522	0.942722
bomi vs milin	ko04612	Antigen pr	1/222	106/8136	0.947759	0.997522	0.942722
bomi vs milin	ko03010	Ribosome	1/222	107/8136	0.949203	0.997522	0.942722
bomi vs milin	ko04662	B cell rece	1/222	107/8136	0.949203	0.997522	0.942722
bomi vs milin	ko04144	Endocytos	5/222	329/8136	0.94994	0.997522	0.942722
bomi vs milin	ko05203	Viral carcir	3/222	230/8136	0.953606	0.997522	0.942722
bomi vs milin	ko05218	Melanoma	1/222	114/8136	0.958259	0.997522	0.942722
bomi vs milin	ko04012	ErbB signa	1/222	117/8136	0.96163	0.997522	0.942722
bomi vs milin	ko04218	Cellular se	2/222	183/8136	0.962664	0.997522	0.942722
bomi vs milin	ko05150	Staphyloco	1/222	118/8136	0.962692	0.997522	0.942722
bomi vs milin	ko04640	Hematopo	1/222	120/8136	0.964729	0.997522	0.942722
bomi vs milin	ko04915	Estrogen s	2/222	186/8136	0.965221	0.997522	0.942722
bomi vs milin	ko04140	Autophagy	2/222	189/8136	0.967609	0.997522	0.942722
bomi vs milin	ko04721	Synaptic v	1/222	126/8136	0.970201	0.997522	0.942722
bomi vs milin	ko05140	Leishmania	1/222	127/8136	0.971027	0.997522	0.942722
bomi vs milin	ko04727	GABAergic	1/222	129/8136	0.972611	0.997522	0.942722
bomi vs milin	ko04150	mTOR sign	2/222	202/8136	0.97626	0.997522	0.942722
bomi vs milin	ko05034	Alcoholism	2/222	205/8136	0.977914	0.997522	0.942722
bomi vs milin	ko05226	Gastric car	2/222	206/8136	0.97844	0.997522	0.942722
bomi vs milin	ko05323	Rheumato	1/222	138/8136	0.978737	0.997522	0.942722
bomi vs milin	ko04066	HIF-1 sign	1/222	145/8136	0.982541	0.997522	0.942722
bomi vs milin	ko04916	Melanoge	1/222	148/8136	0.983956	0.997522	0.942722
bomi vs milin	ko04142	Lysosome	1/222	150/8136	0.984836	0.997522	0.942722
bomi vs milin	ko05205	Proteoglyc	3/222	291/8136	0.987614	0.997522	0.942722
bomi vs milin	ko01240	NA	1/222	166/8136	0.990345	0.997522	0.942722
bomi vs milin	ko04713	Circadian e	1/222	182/8136	0.993859	0.997522	0.942722
bomi vs milin	ko05224	Breast can	1/222	209/8136	0.997144	0.997522	0.942722
bomi vs milin	ko05225	Hepatocel	1/222	214/8136	0.997522	0.997522	0.942722
bomi vs linzhi	ko03013	RNA trans	9/181	101/8136	0.000393	0.103044	0.103044
bomi vs linzhi	ko03460	Fanconi ar	5/181	46/8136	0.003376	0.35181	0.35181
bomi vs linzhi	ko04613	NA	11/181	202/8136	0.005341	0.35181	0.35181
bomi vs linzhi	ko05322	Systemic lu	10/181	174/8136	0.005371	0.35181	0.35181
bomi vs linzhi	ko05144	Malaria	4/181	42/8136	0.013728	0.699642	0.699642
bomi vs linzhi	ko05034	Alcoholism	10/181	205/8136	0.016022	0.699642	0.699642
bomi vs linzhi	ko05140	Leishmania	7/181	127/8136	0.022975	0.859908	0.859908
bomi vs linzhi	ko00510	N-Glycan	4/181	58/8136	0.039647	0.985713	0.985713
bomi vs linzhi	ko03015	mRNA sur	5/181	86/8136	0.042335	0.985713	0.985713
bomi vs linzhi	ko04217	Necroptos	9/181	219/8136	0.055412	0.985713	0.985713
bomi vs linzhi	ko05133	Pertussis	5/181	96/8136	0.062446	0.985713	0.985713
bomi vs linzhi	ko05146	Amoebiasi	6/181	127/8136	0.063654	0.985713	0.985713
bomi vs linzhi	ko04621	NOD-like	7/181	165/8136	0.074927	0.985713	0.985713
bomi vs linzhi	ko01523	Antifolate	3/181	46/8136	0.082031	0.985713	0.985713

bomi vs linzhi	ko00120	Primary bi	2/181	23/8136	0.091826	0.985713	0.985713
bomi vs linzhi	ko03050	Proteasom	3/181	51/8136	0.104033	0.985713	0.985713
bomi vs linzhi	ko04624	Toll and In	3/181	51/8136	0.104033	0.985713	0.985713
bomi vs linzhi	ko05014	Amyotropl	13/181	406/8136	0.118488	0.985713	0.985713
bomi vs linzhi	ko04926	Relaxin sig	6/181	157/8136	0.13721	0.985713	0.985713
bomi vs linzhi	ko04514	Cell adhes	9/181	267/8136	0.140729	0.985713	0.985713
bomi vs linzhi	ko05231	Choline m	6/181	159/8136	0.143147	0.985713	0.985713
bomi vs linzhi	ko04658	Th1 and Th	5/181	125/8136	0.145727	0.985713	0.985713
bomi vs linzhi	ko05010	Alzheimer	14/181	462/8136	0.147833	0.985713	0.985713
bomi vs linzhi	ko04979	Cholestero	3/181	60/8136	0.1486	0.985713	0.985713
bomi vs linzhi	ko04928	Parathyroi	6/181	161/8136	0.1492	0.985713	0.985713
bomi vs linzhi	ko00220	Arginine b	2/181	31/8136	0.150758	0.985713	0.985713
bomi vs linzhi	ko04622	RIG-I-like	3/181	62/8136	0.159235	0.985713	0.985713
bomi vs linzhi	ko05145	Toxoplasm	6/181	167/8136	0.168036	0.985713	0.985713
bomi vs linzhi	ko01040	Biosynthes	2/181	34/8136	0.174427	0.985713	0.985713
bomi vs linzhi	ko05171	NA	8/181	246/8136	0.18209	0.985713	0.985713
bomi vs linzhi	ko00062	Fatty acid	2/181	35/8136	0.182446	0.985713	0.985713
bomi vs linzhi	ko04744	Phototrans	2/181	35/8136	0.182446	0.985713	0.985713
bomi vs linzhi	ko00330	Arginine a	3/181	67/8136	0.18677	0.985713	0.985713
bomi vs linzhi	ko05417	NA	8/181	248/8136	0.187634	0.985713	0.985713
bomi vs linzhi	ko00130	Ubiquinon	1/181	10/8136	0.201568	0.985713	0.985713
bomi vs linzhi	ko04330	Notch sign	3/181	70/8136	0.203849	0.985713	0.985713
bomi vs linzhi	ko04950	Maturity o	2/181	39/8136	0.214994	0.985713	0.985713
bomi vs linzhi	ko05222	Small cell	4/181	110/8136	0.229016	0.985713	0.985713
bomi vs linzhi	ko05134	Legionello	3/181	75/8136	0.233054	0.985713	0.985713
bomi vs linzhi	ko04659	Th17 cell d	5/181	148/8136	0.233345	0.985713	0.985713
bomi vs linzhi	ko00430	Taurine an	1/181	12/8136	0.236743	0.985713	0.985713
bomi vs linzhi	ko04623	Cytosolic D	2/181	45/8136	0.264614	0.985713	0.985713
bomi vs linzhi	ko04918	Thyroid ho	3/181	82/8136	0.275057	0.985713	0.985713
bomi vs linzhi	ko05132	Salmonella	9/181	318/8136	0.276734	0.985713	0.985713
bomi vs linzhi	ko04061	Viral prote	3/181	84/8136	0.287214	0.985713	0.985713
bomi vs linzhi	ko00513	Various typ	2/181	48/8136	0.289498	0.985713	0.985713
bomi vs linzhi	ko00760	Nicotinate	2/181	49/8136	0.297776	0.985713	0.985713
bomi vs linzhi	ko01521	EGFR tyros	4/181	124/8136	0.297792	0.985713	0.985713
bomi vs linzhi	ko05226	Gastric car	6/181	206/8136	0.309465	0.985713	0.985713
bomi vs linzhi	ko05169	Epstein-Ba	7/181	247/8136	0.310956	0.985713	0.985713
bomi vs linzhi	ko05142	Chagas dis	4/181	128/8136	0.317954	0.985713	0.985713
bomi vs linzhi	ko00061	Fatty acid	1/181	17/8136	0.318083	0.985713	0.985713
bomi vs linzhi	ko05205	Proteoglyc	8/181	291/8136	0.321041	0.985713	0.985713
bomi vs linzhi	ko04727	GABAergic	4/181	129/8136	0.323015	0.985713	0.985713
bomi vs linzhi	ko04720	Long-term	3/181	90/8136	0.323885	0.985713	0.985713
bomi vs linzhi	ko00514	Other type	2/181	53/8136	0.330708	0.985713	0.985713
bomi vs linzhi	ko00670	One carbo	1/181	18/8136	0.333285	0.985713	0.985713
bomi vs linzhi	ko00910	Nitrogen n	1/181	18/8136	0.333285	0.985713	0.985713
bomi vs linzhi	ko04657	IL-17 signa	3/181	92/8136	0.33613	0.985713	0.985713
bomi vs linzhi	ko02010	ABC transp	2/181	54/8136	0.33888	0.985713	0.985713
bomi vs linzhi	ko00340	Histidine n	1/181	20/8136	0.362686	0.985713	0.985713
bomi vs linzhi	ko04725	Cholinergi	5/181	178/8136	0.363094	0.985713	0.985713
bomi vs linzhi	ko04512	ECM-recep	3/181	98/8136	0.372764	0.985713	0.985713
bomi vs linzhi	ko05017	Spinocereb	5/181	181/8136	0.376426	0.985713	0.985713
bomi vs linzhi	ko03430	Mismatch	1/181	21/8136	0.376899	0.985713	0.985713
bomi vs linzhi	ko04625	C-type lec	4/181	141/8136	0.383944	0.985713	0.985713
bomi vs linzhi	ko04620	Toll-like re	3/181	100/8136	0.384903	0.985713	0.985713
bomi vs linzhi	ko00770	Pantothen	1/181	22/8136	0.390797	0.985713	0.985713
bomi vs linzhi	ko01210	2-Oxocarb	1/181	22/8136	0.390797	0.985713	0.985713
bomi vs linzhi	ko05235	PD-L1 exp	3/181	101/8136	0.390953	0.985713	0.985713
bomi vs linzhi	ko05310	Asthma	2/181	62/8136	0.402958	0.985713	0.985713
bomi vs linzhi	ko01212	Fatty acid	2/181	64/8136	0.418536	0.985713	0.985713

bomi vs linzhi	ko03008	Ribosome	2/181	64/8136	0.418536	0.985713	0.985713
bomi vs linzhi	ko03010	Ribosome	3/181	107/8136	0.42691	0.985713	0.985713
bomi vs linzhi	ko04662	B cell rece	3/181	107/8136	0.42691	0.985713	0.985713
bomi vs linzhi	ko04261	Adrenergi	6/181	236/8136	0.428931	0.985713	0.985713
bomi vs linzhi	ko03040	Spliceoso	4/181	152/8136	0.439283	0.985713	0.985713
bomi vs linzhi	ko00020	Citrate cyc	1/181	26/8136	0.443372	0.985713	0.985713
bomi vs linzhi	ko05410	Hypertrop	4/181	154/8136	0.449211	0.985713	0.985713
bomi vs linzhi	ko00650	Butanoate	1/181	27/8136	0.455795	0.985713	0.985713
bomi vs linzhi	ko05152	Tuberculos	6/181	245/8136	0.46458	0.985713	0.985713
bomi vs linzhi	ko00900	Terpenoid	1/181	28/8136	0.467942	0.985713	0.985713
bomi vs linzhi	ko05168	Herpes sin	6/181	246/8136	0.468509	0.985713	0.985713
bomi vs linzhi	ko04150	mTOR sign	5/181	202/8136	0.46892	0.985713	0.985713
bomi vs linzhi	ko00310	Lysine deg	2/181	72/8136	0.478668	0.985713	0.985713
bomi vs linzhi	ko04640	Hematopo	3/181	120/8136	0.501965	0.985713	0.985713
bomi vs linzhi	ko04745	Phototrans	1/181	31/8136	0.502789	0.985713	0.985713
bomi vs linzhi	ko05164	Influenza A	5/181	211/8136	0.507394	0.985713	0.985713
bomi vs linzhi	ko00410	beta-Alan	1/181	32/8136	0.513893	0.985713	0.985713
bomi vs linzhi	ko04966	Collecting	1/181	32/8136	0.513893	0.985713	0.985713
bomi vs linzhi	ko05033	Nicotine a	2/181	79/8136	0.528082	0.985713	0.985713
bomi vs linzhi	ko01230	Biosynthes	2/181	80/8136	0.534881	0.985713	0.985713
bomi vs linzhi	ko04940	Type I diab	2/181	80/8136	0.534881	0.985713	0.985713
bomi vs linzhi	ko01250	NA	1/181	34/8136	0.535366	0.985713	0.985713
bomi vs linzhi	ko05135	Yersinia in	5/181	219/8136	0.540652	0.985713	0.985713
bomi vs linzhi	ko01524	Platinum d	2/181	81/8136	0.541612	0.985713	0.985713
bomi vs linzhi	ko03410	Base excisi	1/181	35/8136	0.545746	0.985713	0.985713
bomi vs linzhi	ko04392	Hippo sign	1/181	35/8136	0.545746	0.985713	0.985713
bomi vs linzhi	ko05412	Arrhythmo	3/181	129/8136	0.550913	0.985713	0.985713
bomi vs linzhi	ko04380	Osteoclast	3/181	131/8136	0.561404	0.985713	0.985713
bomi vs linzhi	ko04668	TNF signal	3/181	131/8136	0.561404	0.985713	0.985713
bomi vs linzhi	ko05414	Dilated ca	4/181	179/8136	0.567332	0.985713	0.985713
bomi vs linzhi	ko04611	Platelet ac	4/181	180/8136	0.571771	0.985713	0.985713
bomi vs linzhi	ko04670	Leukocyte	4/181	180/8136	0.571771	0.985713	0.985713
bomi vs linzhi	ko04114	Oocyte me	3/181	134/8136	0.576862	0.985713	0.985713
bomi vs linzhi	ko03320	PPAR sign	2/181	87/8136	0.580575	0.985713	0.985713
bomi vs linzhi	ko05203	Viral carcin	5/181	230/8136	0.584626	0.985713	0.985713
bomi vs linzhi	ko00970	Aminoacyl	1/181	39/8136	0.58501	0.985713	0.985713
bomi vs linzhi	ko04977	Vitamin di	1/181	39/8136	0.58501	0.985713	0.985713
bomi vs linzhi	ko04936	NA	3/181	138/8136	0.596939	0.985713	0.985713
bomi vs linzhi	ko04144	Endocytos	7/181	329/8136	0.602632	0.985713	0.985713
bomi vs linzhi	ko00380	Tryptopha	1/181	41/8136	0.603357	0.985713	0.985713
bomi vs linzhi	ko04710	Circadian r	1/181	41/8136	0.603357	0.985713	0.985713
bomi vs linzhi	ko04672	Intestinal i	2/181	92/8136	0.611158	0.985713	0.985713
bomi vs linzhi	ko05217	Basal cell c	2/181	92/8136	0.611158	0.985713	0.985713
bomi vs linzhi	ko00260	Glycine, se	1/181	42/8136	0.612226	0.985713	0.985713
bomi vs linzhi	ko04212	Longevity	2/181	93/8136	0.617068	0.985713	0.985713
bomi vs linzhi	ko05223	Non-small	2/181	93/8136	0.617068	0.985713	0.985713
bomi vs linzhi	ko00071	Fatty acid	1/181	43/8136	0.620897	0.985713	0.985713
bomi vs linzhi	ko00534	Glycosami	1/181	43/8136	0.620897	0.985713	0.985713
bomi vs linzhi	ko05020	Prion disea	7/181	335/8136	0.62181	0.985713	0.985713
bomi vs linzhi	ko04728	Dopamine	4/181	193/8136	0.627104	0.985713	0.985713
bomi vs linzhi	ko00051	Fructose a	1/181	44/8136	0.629376	0.985713	0.985713
bomi vs linzhi	ko00250	Alanine, as	1/181	44/8136	0.629376	0.985713	0.985713
bomi vs linzhi	ko04066	HIF-1 sign	3/181	145/8136	0.630563	0.985713	0.985713
bomi vs linzhi	ko04146	Peroxisom	2/181	96/8136	0.634386	0.985713	0.985713
bomi vs linzhi	ko04550	Signaling p	4/181	195/8136	0.635207	0.985713	0.985713
bomi vs linzhi	ko04750	Inflammat	3/181	146/8136	0.635207	0.985713	0.985713
bomi vs linzhi	ko04390	Hippo sign	4/181	196/8136	0.639216	0.985713	0.985713
bomi vs linzhi	ko05031	Amphetam	2/181	97/8136	0.640022	0.985713	0.985713

bomi vs linzhi	ko04916	Melanoge	3/181	148/8136	0.644373	0.985713	0.985713
bomi vs linzhi	ko00512	Mucin type	1/181	46/8136	0.645772	0.985713	0.985713
bomi vs linzhi	ko05202	Transcripti	5/181	247/8136	0.647905	0.985713	0.985713
bomi vs linzhi	ko04260	Cardiac m	3/181	149/8136	0.648895	0.985713	0.985713
bomi vs linzhi	ko04931	Insulin resi	3/181	149/8136	0.648895	0.985713	0.985713
bomi vs linzhi	ko04370	VEGF signa	2/181	99/8136	0.65109	0.985713	0.985713
bomi vs linzhi	ko05170	Human im	5/181	249/8136	0.654943	0.985713	0.985713
bomi vs linzhi	ko00520	Amino sug	1/181	48/8136	0.661446	0.985713	0.985713
bomi vs linzhi	ko04920	Adipocyto	2/181	101/8136	0.661887	0.985713	0.985713
bomi vs linzhi	ko05162	Measles	3/181	152/8136	0.662217	0.985713	0.985713
bomi vs linzhi	ko04960	Aldosteron	1/181	49/8136	0.669022	0.985713	0.985713
bomi vs linzhi	ko04013	MAPK sign	2/181	103/8136	0.672416	0.985713	0.985713
bomi vs linzhi	ko00280	Valine, leu	1/181	51/8136	0.683673	0.985713	0.985713
bomi vs linzhi	ko04975	Fat digesti	1/181	51/8136	0.683673	0.985713	0.985713
bomi vs linzhi	ko04612	Antigen pr	2/181	106/8136	0.687713	0.985713	0.985713
bomi vs linzhi	ko00230	Purine me	3/181	158/8136	0.687761	0.985713	0.985713
bomi vs linzhi	ko05224	Breast can	4/181	209/8136	0.688707	0.985713	0.985713
bomi vs linzhi	ko04722	Neurotrop	3/181	160/8136	0.695951	0.985713	0.985713
bomi vs linzhi	ko04070	Phosphatid	2/181	111/8136	0.711906	0.985713	0.985713
bomi vs linzhi	ko05165	Human pa	8/181	417/8136	0.71691	0.985713	0.985713
bomi vs linzhi	ko04962	Vasopress	1/181	56/8136	0.717539	0.985713	0.985713
bomi vs linzhi	ko05143	African try	1/181	56/8136	0.717539	0.985713	0.985713
bomi vs linzhi	ko01240	NA	3/181	166/8136	0.71955	0.985713	0.985713
bomi vs linzhi	ko05160	Hepatitis C	4/181	218/8136	0.720072	0.985713	0.985713
bomi vs linzhi	ko04064	NF-kappa	2/181	114/8136	0.72566	0.985713	0.985713
bomi vs linzhi	ko04914	Progestero	2/181	116/8136	0.73452	0.985713	0.985713
bomi vs linzhi	ko04510	Focal adhe	5/181	276/8136	0.741024	0.985713	0.985713
bomi vs linzhi	ko05150	Staphyloco	2/181	118/8136	0.743137	0.985713	0.985713
bomi vs linzhi	ko04664	Fc epsilon	2/181	119/8136	0.747355	0.985713	0.985713
bomi vs linzhi	ko04062	Chemokin	4/181	227/8136	0.749063	0.985713	0.985713
bomi vs linzhi	ko04010	MAPK sign	8/181	431/8136	0.750467	0.985713	0.985713
bomi vs linzhi	ko01200	Carbon me	2/181	121/8136	0.755614	0.985713	0.985713
bomi vs linzhi	ko05415	NA	4/181	230/8136	0.758206	0.985713	0.985713
bomi vs linzhi	ko04060	Cytokine-c	4/181	232/8136	0.764158	0.985713	0.985713
bomi vs linzhi	ko04919	Thyroid ho	3/181	180/8136	0.769088	0.985713	0.985713
bomi vs linzhi	ko04721	Synaptic v	2/181	126/8136	0.775255	0.985713	0.985713
bomi vs linzhi	ko05161	Hepatitis B	3/181	182/8136	0.775553	0.985713	0.985713
bomi vs linzhi	ko04218	Cellular se	3/181	183/8136	0.77873	0.985713	0.985713
bomi vs linzhi	ko05332	Graft-vers	1/181	67/8136	0.77988	0.985713	0.985713
bomi vs linzhi	ko05416	Viral myoc	2/181	128/8136	0.78272	0.985713	0.985713
bomi vs linzhi	ko04660	T cell rece	2/181	129/8136	0.78637	0.985713	0.985713
bomi vs linzhi	ko04020	Calcium si	7/181	398/8136	0.78948	0.985713	0.985713
bomi vs linzhi	ko04961	Endocrine	1/181	69/8136	0.789645	0.985713	0.985713
bomi vs linzhi	ko04933	AGE-RAGE	2/181	130/8136	0.789967	0.985713	0.985713
bomi vs linzhi	ko05032	Morphine	2/181	130/8136	0.789967	0.985713	0.985713
bomi vs linzhi	ko00190	Oxidative	2/181	131/8136	0.79351	0.985713	0.985713
bomi vs linzhi	ko05030	Cocaine ad	1/181	70/8136	0.794365	0.985713	0.985713
bomi vs linzhi	ko04912	GnRH sign	2/181	132/8136	0.797001	0.985713	0.985713
bomi vs linzhi	ko04024	cAMP sign	6/181	351/8136	0.79974	0.985713	0.985713
bomi vs linzhi	ko04976	Bile secret	2/181	133/8136	0.80044	0.985713	0.985713
bomi vs linzhi	ko05221	Acute mye	1/181	73/8136	0.807902	0.985713	0.985713
bomi vs linzhi	ko04071	Sphingolip	3/181	193/8136	0.808515	0.985713	0.985713
bomi vs linzhi	ko05012	Parkinson	5/181	303/8136	0.81039	0.985713	0.985713
bomi vs linzhi	ko04911	Insulin sec	2/181	137/8136	0.813688	0.985713	0.985713
bomi vs linzhi	ko00010	Glycolysis	1/181	75/8136	0.81643	0.985713	0.985713
bomi vs linzhi	ko04115	p53 signal	1/181	75/8136	0.81643	0.985713	0.985713
bomi vs linzhi	ko05323	Rheumato	2/181	138/8136	0.816875	0.985713	0.985713
bomi vs linzhi	ko00240	Pyrimidine	1/181	76/8136	0.820552	0.985713	0.985713

bomi vs linzhi	ko04141	Protein pro	3/181	199/8136	0.824723	0.985713	0.985713
bomi vs linzhi	ko04972	Pancreatic	2/181	141/8136	0.826148	0.985713	0.985713
bomi vs linzhi	ko04932	Non-alcoh	3/181	203/8136	0.83487	0.985713	0.985713
bomi vs linzhi	ko04210	Apoptosis	3/181	205/8136	0.839752	0.985713	0.985713
bomi vs linzhi	ko05213	Endometri	1/181	81/8136	0.83982	0.985713	0.985713
bomi vs linzhi	ko04015	Rap1 signa	5/181	317/8136	0.840117	0.985713	0.985713
bomi vs linzhi	ko04810	Regulation	5/181	317/8136	0.840117	0.985713	0.985713
bomi vs linzhi	ko04723	Retrograde	3/181	206/8136	0.842147	0.985713	0.985713
bomi vs linzhi	ko04310	Wnt signal	3/181	209/8136	0.849147	0.985713	0.985713
bomi vs linzhi	ko00562	Inositol ph	1/181	84/8136	0.850379	0.985713	0.985713
bomi vs linzhi	ko04913	Ovarian st	1/181	85/8136	0.853742	0.985713	0.985713
bomi vs linzhi	ko05330	Allograft r	1/181	86/8136	0.85703	0.985713	0.985713
bomi vs linzhi	ko05225	Hepatocel	3/181	214/8136	0.860218	0.985713	0.985713
bomi vs linzhi	ko04391	Hippo sign	1/181	87/8136	0.860245	0.985713	0.985713
bomi vs linzhi	ko04610	Compleme	1/181	87/8136	0.860245	0.985713	0.985713
bomi vs linzhi	ko04151	PI3K-Akt s	7/181	437/8136	0.861363	0.985713	0.985713
bomi vs linzhi	ko04934	Cushing sy	3/181	215/8136	0.862345	0.985713	0.985713
bomi vs linzhi	ko05167	Kaposi sar	3/181	215/8136	0.862345	0.985713	0.985713
bomi vs linzhi	ko05320	Autoimmu	1/181	88/8136	0.863388	0.985713	0.985713
bomi vs linzhi	ko05207	NA	4/181	276/8136	0.868223	0.985713	0.985713
bomi vs linzhi	ko05321	Inflammat	1/181	90/8136	0.869464	0.985713	0.985713
bomi vs linzhi	ko04213	Longevity	1/181	91/8136	0.8724	0.985713	0.985713
bomi vs linzhi	ko04726	Serotoner	2/181	161/8136	0.877908	0.985713	0.985713
bomi vs linzhi	ko04740	Olfactory t	1/181	93/8136	0.878078	0.985713	0.985713
bomi vs linzhi	ko04917	Prolactin s	1/181	94/8136	0.880821	0.985713	0.985713
bomi vs linzhi	ko05166	Human T-	4/181	284/8136	0.882204	0.985713	0.985713
bomi vs linzhi	ko05230	Central ca	1/181	95/8136	0.883504	0.985713	0.985713
bomi vs linzhi	ko04929	GnRH secr	1/181	98/8136	0.891196	0.985713	0.985713
bomi vs linzhi	ko04068	FoxO signa	2/181	168/8136	0.892396	0.985713	0.985713
bomi vs linzhi	ko04921	Oxytocin s	3/181	232/8136	0.894411	0.985713	0.985713
bomi vs linzhi	ko05163	Human cy	4/181	292/8136	0.894888	0.985713	0.985713
bomi vs linzhi	ko05214	Glioma	1/181	100/8136	0.896041	0.985713	0.985713
bomi vs linzhi	ko04910	Insulin sig	2/181	173/8136	0.901755	0.985713	0.985713
bomi vs linzhi	ko04935	Growth ho	2/181	174/8136	0.903533	0.985713	0.985713
bomi vs linzhi	ko05211	Renal cell	1/181	104/8136	0.905097	0.985713	0.985713
bomi vs linzhi	ko04924	Renin secr	1/181	106/8136	0.909326	0.985713	0.985713
bomi vs linzhi	ko04971	Gastric aci	1/181	107/8136	0.91137	0.985713	0.985713
bomi vs linzhi	ko05418	Fluid shea	2/181	181/8136	0.91517	0.985713	0.985713
bomi vs linzhi	ko05220	Chronic m	1/181	109/8136	0.915321	0.985713	0.985713
bomi vs linzhi	ko04713	Circadian	2/181	182/8136	0.916722	0.985713	0.985713
bomi vs linzhi	ko04350	TGF-beta	1/181	110/8136	0.917231	0.985713	0.985713
bomi vs linzhi	ko04014	Ras signali	4/181	309/8136	0.917946	0.985713	0.985713
bomi vs linzhi	ko04915	Estrogen s	2/181	186/8136	0.922666	0.985713	0.985713
bomi vs linzhi	ko04730	Long-term	1/181	114/8136	0.92445	0.985713	0.985713
bomi vs linzhi	ko04970	Salivary se	1/181	114/8136	0.92445	0.985713	0.985713
bomi vs linzhi	ko05218	Melanoma	1/181	114/8136	0.92445	0.985713	0.985713
bomi vs linzhi	ko05100	Bacterial in	1/181	115/8136	0.926154	0.985713	0.985713
bomi vs linzhi	ko04724	Glutamate	2/181	189/8136	0.92686	0.985713	0.985713
bomi vs linzhi	ko04650	Natural kil	1/181	116/8136	0.927821	0.985713	0.985713
bomi vs linzhi	ko05208	NA	3/181	256/8136	0.928341	0.985713	0.985713
bomi vs linzhi	ko04012	ErbB signa	1/181	117/8136	0.92945	0.985713	0.985713
bomi vs linzhi	ko04371	Apelin sign	2/181	196/8136	0.935831	0.985713	0.985713
bomi vs linzhi	ko04361	Axon rege	1/181	124/8136	0.939873	0.985713	0.985713
bomi vs linzhi	ko05215	Prostate ca	1/181	126/8136	0.942559	0.985713	0.985713
bomi vs linzhi	ko04270	Vascular sr	2/181	203/8136	0.943758	0.985713	0.985713
bomi vs linzhi	ko01522	Endocrine	1/181	130/8136	0.947579	0.985713	0.985713
bomi vs linzhi	ko05016	Huntingto	4/181	343/8136	0.95103	0.985713	0.985713
bomi vs linzhi	ko05210	Colorectal	1/181	134/8136	0.952162	0.985713	0.985713

bomi vs linzhi	ko04360	Axon guid	3/181	281/8136	0.952839	0.985713	0.985713
bomi vs linzhi	ko04110	Cell cycle	1/181	135/8136	0.953244	0.985713	0.985713
bomi vs linzhi	ko04925	Aldosteror	1/181	137/8136	0.955336	0.985713	0.985713
bomi vs linzhi	ko04145	Phagosom	2/181	222/8136	0.96086	0.985713	0.985713
bomi vs linzhi	ko04922	Glucagon	1/181	143/8136	0.961068	0.985713	0.985713
bomi vs linzhi	ko04630	JAK-STAT	1/181	144/8136	0.96195	0.985713	0.985713
bomi vs linzhi	ko04072	Phospholi	2/181	224/8136	0.96234	0.985713	0.985713
bomi vs linzhi	ko04540	Gap juncti	1/181	145/8136	0.962812	0.985713	0.985713
bomi vs linzhi	ko04142	Lysosome	1/181	150/8136	0.966838	0.985713	0.985713
bomi vs linzhi	ko04152	AMPK sigr	1/181	152/8136	0.968324	0.985713	0.985713
bomi vs linzhi	ko04666	Fc gamma	1/181	153/8136	0.969042	0.985713	0.985713
bomi vs linzhi	ko04120	Ubiquitin r	1/181	156/8136	0.971101	0.985713	0.985713
bomi vs linzhi	ko04714	Thermoge	2/181	246/8136	0.975456	0.985713	0.985713
bomi vs linzhi	ko04022	cGMP-PK(	2/181	252/8136	0.978189	0.985713	0.985713
bomi vs linzhi	ko05206	MicroRNA	1/181	204/8136	0.990424	0.994219	0.994219
bomi vs linzhi	ko04530	Tight junct	1/181	259/8136	0.997321	0.997321	0.997321
bomi vs zangga	ko00120	Primary bi	4/198	23/8136	0.002096	0.595191	0.595191
bomi vs zangga	ko05322	Systemic lu	10/198	174/8136	0.00989	0.944141	0.944141
bomi vs zangga	ko04911	Insulin sec	8/198	137/8136	0.018465	0.944141	0.944141
bomi vs zangga	ko03420	Nucleotide	4/198	42/8136	0.018515	0.944141	0.944141
bomi vs zangga	ko00020	Citrate cyc	3/198	26/8136	0.024455	0.944141	0.944141
bomi vs zangga	ko04659	Th17 cell d	8/198	148/8136	0.027825	0.944141	0.944141
bomi vs zangga	ko00900	Terpenoid	3/198	28/8136	0.029746	0.944141	0.944141
bomi vs zangga	ko05132	Salmonella	13/198	318/8136	0.046391	0.944141	0.944141
bomi vs zangga	ko01240	NA	8/198	166/8136	0.049477	0.944141	0.944141
bomi vs zangga	ko04061	Viral prote	5/198	84/8136	0.053517	0.944141	0.944141
bomi vs zangga	ko00730	Thiamine r	2/198	16/8136	0.05654	0.944141	0.944141
bomi vs zangga	ko04540	Gap juncti	7/198	145/8136	0.063402	0.944141	0.944141
bomi vs zangga	ko04622	RIG-I-like	4/198	62/8136	0.063701	0.944141	0.944141
bomi vs zangga	ko05310	Asthma	4/198	62/8136	0.063701	0.944141	0.944141
bomi vs zangga	ko04664	Fc epsilon	6/198	119/8136	0.069918	0.944141	0.944141
bomi vs zangga	ko04657	IL-17 signa	5/198	92/8136	0.073229	0.944141	0.944141
bomi vs zangga	ko01200	Carbon me	6/198	121/8136	0.07449	0.944141	0.944141
bomi vs zangga	ko05332	Graft-vers	4/198	67/8136	0.079869	0.944141	0.944141
bomi vs zangga	ko00620	Pyruvate n	3/198	42/8136	0.081645	0.944141	0.944141
bomi vs zangga	ko04658	Th1 and Th	6/198	125/8136	0.084133	0.944141	0.944141
bomi vs zangga	ko04217	Necroptos	9/198	219/8136	0.086324	0.944141	0.944141
bomi vs zangga	ko04961	Endocrine	4/198	69/8136	0.086856	0.944141	0.944141
bomi vs zangga	ko03430	Mismatch	2/198	21/8136	0.091488	0.944141	0.944141
bomi vs zangga	ko04145	Phagosom	9/198	222/8136	0.092049	0.944141	0.944141
bomi vs zangga	ko05412	Arrhythmo	6/198	129/8136	0.094438	0.944141	0.944141
bomi vs zangga	ko04072	Phospholi	9/198	224/8136	0.095987	0.944141	0.944141
bomi vs zangga	ko05010	Alzheimer	16/198	462/8136	0.097104	0.944141	0.944141
bomi vs zangga	ko04742	Taste trans	4/198	72/8136	0.097872	0.944141	0.944141
bomi vs zangga	ko00830	Retinol me	4/198	73/8136	0.101682	0.944141	0.944141
bomi vs zangga	ko04912	GnRH sign	6/198	132/8136	0.102593	0.944141	0.944141
bomi vs zangga	ko00604	Glycosphir	2/198	23/8136	0.106845	0.944141	0.944141
bomi vs zangga	ko05145	Toxoplasm	7/198	167/8136	0.112709	0.944141	0.944141
bomi vs zangga	ko04613	NA	8/198	202/8136	0.119465	0.944141	0.944141
bomi vs zangga	ko00280	Valine, leu	3/198	51/8136	0.126899	0.944141	0.944141
bomi vs zangga	ko04210	Apoptosis	8/198	205/8136	0.126926	0.944141	0.944141
bomi vs zangga	ko04940	Type I diab	4/198	80/8136	0.130189	0.944141	0.944141
bomi vs zangga	ko05414	Dilated ca	7/198	179/8136	0.146226	0.944141	0.944141
bomi vs zangga	ko00630	Glyoxylate	2/198	28/8136	0.147779	0.944141	0.944141
bomi vs zangga	ko04914	Progester	5/198	116/8136	0.15226	0.944141	0.944141
bomi vs zangga	ko05330	Allograft r	4/198	86/8136	0.156954	0.944141	0.944141
bomi vs zangga	ko05150	Staphyloco	5/198	118/8136	0.160046	0.944141	0.944141
bomi vs zangga	ko05320	Autoimmu	4/198	88/8136	0.1663	0.944141	0.944141

bomi vs zangga	ko05410	Hypertrop	6/198	154/8136	0.172749	0.944141	0.944141
bomi vs zangga	ko04745	Phototrans	2/198	31/8136	0.173638	0.944141	0.944141
bomi vs zangga	ko05321	Inflammat	4/198	90/8136	0.175842	0.944141	0.944141
bomi vs zangga	ko04530	Tight junct	9/198	259/8136	0.180016	0.944141	0.944141
bomi vs zangga	ko00030	Pentose pl	2/198	32/8136	0.182416	0.944141	0.944141
bomi vs zangga	ko04672	Intestinal i	4/198	92/8136	0.185566	0.944141	0.944141
bomi vs zangga	ko05020	Prion disea	11/198	335/8136	0.19308	0.944141	0.944141
bomi vs zangga	ko05140	Leishmania	5/198	127/8136	0.196959	0.944141	0.944141
bomi vs zangga	ko04726	Serotonerg	6/198	161/8136	0.198439	0.944141	0.944141
bomi vs zangga	ko01040	Biosynthes	2/198	34/8136	0.200154	0.944141	0.944141
bomi vs zangga	ko05416	Viral myoc	5/198	128/8136	0.201234	0.944141	0.944141
bomi vs zangga	ko05012	Parkinson	10/198	303/8136	0.203551	0.944141	0.944141
bomi vs zangga	ko05133	Pertussis	4/198	96/8136	0.205517	0.944141	0.944141
bomi vs zangga	ko00062	Fatty acid	2/198	35/8136	0.209099	0.944141	0.944141
bomi vs zangga	ko03030	DNA replic	2/198	35/8136	0.209099	0.944141	0.944141
bomi vs zangga	ko03410	Base excisi	2/198	35/8136	0.209099	0.944141	0.944141
bomi vs zangga	ko04933	AGE-RAGE	5/198	130/8136	0.209877	0.944141	0.944141
bomi vs zangga	ko04621	NOD-like	6/198	165/8136	0.213727	0.944141	0.944141
bomi vs zangga	ko05016	Huntingto	11/198	343/8136	0.213874	0.944141	0.944141
bomi vs zangga	ko04668	TNF signal	5/198	131/8136	0.214244	0.944141	0.944141
bomi vs zangga	ko04261	Adrenergic	8/198	236/8136	0.216949	0.944141	0.944141
bomi vs zangga	ko00130	Ubiquinon	1/198	10/8136	0.218477	0.944141	0.944141
bomi vs zangga	ko04370	VEGF signa	4/198	99/8136	0.220875	0.944141	0.944141
bomi vs zangga	ko04270	Vascular sı	7/198	203/8136	0.22522	0.944141	0.944141
bomi vs zangga	ko04068	FoxO signa	6/198	168/8136	0.225455	0.944141	0.944141
bomi vs zangga	ko04620	Toll-like re	4/198	100/8136	0.226062	0.944141	0.944141
bomi vs zangga	ko04950	Maturity o	2/198	39/8136	0.245211	0.962967	0.962967
bomi vs zangga	ko04936	NA	5/198	138/8136	0.245573	0.962967	0.962967
bomi vs zangga	ko05323	Rheumato	5/198	138/8136	0.245573	0.962967	0.962967
bomi vs zangga	ko05168	Herpes sin	8/198	246/8136	0.25023	0.962967	0.962967
bomi vs zangga	ko05169	Epstein-Ba	8/198	247/8136	0.253648	0.962967	0.962967
bomi vs zangga	ko05164	Influenza A	7/198	211/8136	0.254403	0.962967	0.962967
bomi vs zangga	ko00430	Taurine an	1/198	12/8136	0.256101	0.962967	0.962967
bomi vs zangga	ko04612	Antigen pr	4/198	106/8136	0.25779	0.962967	0.962967
bomi vs zangga	ko00010	Glycolysis	3/198	75/8136	0.275292	0.962967	0.962967
bomi vs zangga	ko05134	Legionello	3/198	75/8136	0.275292	0.962967	0.962967
bomi vs zangga	ko05017	Spinocereb	6/198	181/8136	0.278453	0.962967	0.962967
bomi vs zangga	ko00240	Pyrimidine	3/198	76/8136	0.281941	0.962967	0.962967
bomi vs zangga	ko04750	Inflammat	5/198	146/8136	0.282724	0.962967	0.962967
bomi vs zangga	ko05014	Amyotropl	12/198	406/8136	0.284774	0.962967	0.962967
bomi vs zangga	ko04978	Mineral ab	3/198	77/8136	0.288604	0.962967	0.962967
bomi vs zangga	ko04974	Protein dig	4/198	112/8136	0.290342	0.962967	0.962967
bomi vs zangga	ko04916	Melanoger	5/198	148/8136	0.292187	0.962967	0.962967
bomi vs zangga	ko04260	Cardiac m	5/198	149/8136	0.296939	0.962967	0.962967
bomi vs zangga	ko05033	Nicotine a	3/198	79/8136	0.301963	0.962967	0.962967
bomi vs zangga	ko00590	Arachidon	3/198	80/8136	0.308656	0.962967	0.962967
bomi vs zangga	ko00512	Mucin type	2/198	46/8136	0.308716	0.962967	0.962967
bomi vs zangga	ko04918	Thyroid ho	3/198	82/8136	0.322055	0.962967	0.962967
bomi vs zangga	ko04071	Sphingolip	6/198	193/8136	0.329657	0.962967	0.962967
bomi vs zangga	ko04640	Hematopo	4/198	120/8136	0.334518	0.962967	0.962967
bomi vs zangga	ko00760	Nicotinate	2/198	49/8136	0.335686	0.962967	0.962967
bomi vs zangga	ko04913	Ovarian st	3/198	85/8136	0.342158	0.962967	0.962967
bomi vs zangga	ko05231	Choline m	5/198	159/8136	0.345045	0.962967	0.962967
bomi vs zangga	ko03050	Proteasom	2/198	51/8136	0.353497	0.962967	0.962967
bomi vs zangga	ko04624	Toll and In	2/198	51/8136	0.353497	0.962967	0.962967
bomi vs zangga	ko00910	Nitrogen r	1/198	18/8136	0.358497	0.962967	0.962967
bomi vs zangga	ko00983	Drug meta	3/198	89/8136	0.368888	0.962967	0.962967
bomi vs zangga	ko00514	Other type	2/198	53/8136	0.37114	0.962967	0.962967

bomi vs zangga	ko05142	Chagas dis	4/198	128/8136	0.378991	0.962967	0.962967
bomi vs zangga	ko05034	Alcoholism	6/198	205/8136	0.381975	0.962967	0.962967
bomi vs zangga	ko04723	Retrograde	6/198	206/8136	0.386354	0.962967	0.962967
bomi vs zangga	ko05226	Gastric car	6/198	206/8136	0.386354	0.962967	0.962967
bomi vs zangga	ko05152	Tuberculos	7/198	245/8136	0.387406	0.962967	0.962967
bomi vs zangga	ko04340	Hedgehog	2/198	55/8136	0.38859	0.962967	0.962967
bomi vs zangga	ko05217	Basal cell c	3/198	92/8136	0.388815	0.962967	0.962967
bomi vs zangga	ko05223	Non-small	3/198	93/8136	0.395426	0.962967	0.962967
bomi vs zangga	ko04310	Wnt signal	6/198	209/8136	0.399489	0.962967	0.962967
bomi vs zangga	ko00533	Glycosami	1/198	21/8136	0.404306	0.962967	0.962967
bomi vs zangga	ko04144	Endocytos	9/198	329/8136	0.408312	0.962967	0.962967
bomi vs zangga	ko04114	Oocyte me	4/198	134/8136	0.412191	0.962967	0.962967
bomi vs zangga	ko04146	Peroxisom	3/198	96/8136	0.415141	0.962967	0.962967
bomi vs zangga	ko05163	Human cy	8/198	292/8136	0.417506	0.962967	0.962967
bomi vs zangga	ko04520	Adherens	4/198	135/8136	0.417693	0.962967	0.962967
bomi vs zangga	ko04935	Growth ho	5/198	174/8136	0.417874	0.962967	0.962967
bomi vs zangga	ko00770	Pantothen	1/198	22/8136	0.418841	0.962967	0.962967
bomi vs zangga	ko04214	Apoptosis	2/198	59/8136	0.422826	0.962967	0.962967
bomi vs zangga	ko05167	Kaposi sar	6/198	215/8136	0.425709	0.962967	0.962967
bomi vs zangga	ko05135	Yersinia in	6/198	219/8136	0.443108	0.962967	0.962967
bomi vs zangga	ko04625	C-type lec	4/198	141/8136	0.450436	0.962967	0.962967
bomi vs zangga	ko04972	Pancreatic	4/198	141/8136	0.450436	0.962967	0.962967
bomi vs zangga	ko05161	Hepatitis B	5/198	182/8136	0.456301	0.962967	0.962967
bomi vs zangga	ko04013	MAPK sign	3/198	103/8136	0.460282	0.962967	0.962967
bomi vs zangga	ko00511	Other glyc	1/198	25/8136	0.460361	0.962967	0.962967
bomi vs zangga	ko01212	Fatty acid	2/198	64/8136	0.464192	0.962967	0.962967
bomi vs zangga	ko04930	Type II dia	2/198	64/8136	0.464192	0.962967	0.962967
bomi vs zangga	ko04062	Chemokin	6/198	227/8136	0.477574	0.962967	0.962967
bomi vs zangga	ko04010	MAPK sign	11/198	431/8136	0.480214	0.962967	0.962967
bomi vs zangga	ko00640	Propanoat	1/198	27/8136	0.486387	0.962967	0.962967
bomi vs zangga	ko04724	Glutamate	5/198	189/8136	0.489351	0.962967	0.962967
bomi vs zangga	ko04921	Oxytocin s	6/198	232/8136	0.49881	0.962967	0.962967
bomi vs zangga	ko04070	Phosphati	3/198	111/8136	0.509971	0.962967	0.962967
bomi vs zangga	ko04666	Fc gamma	4/198	153/8136	0.51398	0.962967	0.962967
bomi vs zangga	ko04550	Signaling p	5/198	195/8136	0.517106	0.962967	0.962967
bomi vs zangga	ko04390	Hippo sign	5/198	196/8136	0.521672	0.962967	0.962967
bomi vs zangga	ko04064	NF-kappa	3/198	114/8136	0.527985	0.962967	0.962967
bomi vs zangga	ko04970	Salivary se	3/198	114/8136	0.527985	0.962967	0.962967
bomi vs zangga	ko05221	Acute mye	2/198	73/8136	0.534059	0.962967	0.962967
bomi vs zangga	ko04926	Relaxin sig	4/198	157/8136	0.534423	0.962967	0.962967
bomi vs zangga	ko00053	Ascorbate	1/198	31/8136	0.534752	0.962967	0.962967
bomi vs zangga	ko00220	Arginine b	1/198	31/8136	0.534752	0.962967	0.962967
bomi vs zangga	ko00230	Purine me	4/198	158/8136	0.539468	0.962967	0.962967
bomi vs zangga	ko05166	Human T-	7/198	284/8136	0.541129	0.962967	0.962967
bomi vs zangga	ko00410	beta-Alan	1/198	32/8136	0.546118	0.962967	0.962967
bomi vs zangga	ko00790	Folate bios	1/198	32/8136	0.546118	0.962967	0.962967
bomi vs zangga	ko04722	Neurotrop	4/198	160/8136	0.549475	0.962967	0.962967
bomi vs zangga	ko04932	Non-alcoh	5/198	203/8136	0.553105	0.962967	0.962967
bomi vs zangga	ko04714	Thermoge	6/198	246/8136	0.556557	0.962967	0.962967
bomi vs zangga	ko05202	Transcripti	6/198	247/8136	0.560568	0.962967	0.962967
bomi vs zangga	ko00592	alpha-Linc	1/198	34/8136	0.568027	0.962967	0.962967
bomi vs zangga	ko00600	Sphingolip	2/198	79/8136	0.577073	0.962967	0.962967
bomi vs zangga	ko03440	Homologo	1/198	35/8136	0.578584	0.962967	0.962967
bomi vs zangga	ko04744	Phototrans	1/198	35/8136	0.578584	0.962967	0.962967
bomi vs zangga	ko05224	Breast can	5/198	209/8136	0.579238	0.962967	0.962967
bomi vs zangga	ko04361	Axon rege	3/198	124/8136	0.585278	0.962967	0.962967
bomi vs zangga	ko00591	Linoleic ac	1/198	36/8136	0.588884	0.962967	0.962967
bomi vs zangga	ko04130	SNARE int	1/198	36/8136	0.588884	0.962967	0.962967

bomi vs zangga	ko04136	Autophagy	1/198	36/8136	0.588884	0.962967	0.962967
bomi vs zangga	ko05213	Endometri	2/198	81/8136	0.590756	0.962967	0.962967
bomi vs zangga	ko00040	Pentose ar	1/198	37/8136	0.598934	0.962967	0.962967
bomi vs zangga	ko05225	Hepatocel	5/198	214/8136	0.600391	0.962967	0.962967
bomi vs zangga	ko05146	Amoebiasi	3/198	127/8136	0.601586	0.962967	0.962967
bomi vs zangga	ko00562	Inositol ph	2/198	84/8136	0.61066	0.962967	0.962967
bomi vs zangga	ko04727	GABAergic	3/198	129/8136	0.612224	0.962967	0.962967
bomi vs zangga	ko01522	Endocrine	3/198	130/8136	0.617471	0.962967	0.962967
bomi vs zangga	ko05032	Morphine	3/198	130/8136	0.617471	0.962967	0.962967
bomi vs zangga	ko04380	Osteoclast	3/198	131/8136	0.62267	0.962967	0.962967
bomi vs zangga	ko03015	mRNA sur	2/198	86/8136	0.623518	0.962967	0.962967
bomi vs zangga	ko03022	Basal trans	1/198	40/8136	0.627639	0.962967	0.962967
bomi vs zangga	ko04391	Hippo sign	2/198	87/8136	0.629823	0.962967	0.962967
bomi vs zangga	ko04610	Compleme	2/198	87/8136	0.629823	0.962967	0.962967
bomi vs zangga	ko04976	Bile secret	3/198	133/8136	0.632925	0.962967	0.962967
bomi vs zangga	ko00380	Tryptopha	1/198	41/8136	0.636746	0.962967	0.962967
bomi vs zangga	ko04514	Cell adhes	6/198	267/8136	0.636947	0.962967	0.962967
bomi vs zangga	ko05210	Colorectal	3/198	134/8136	0.63798	0.962967	0.962967
bomi vs zangga	ko04670	Leukocyte	4/198	180/8136	0.642825	0.962967	0.962967
bomi vs zangga	ko00260	Glycine, se	1/198	42/8136	0.645631	0.962967	0.962967
bomi vs zangga	ko05144	Malaria	1/198	42/8136	0.645631	0.962967	0.962967
bomi vs zangga	ko04720	Long-term	2/198	90/8136	0.648248	0.962967	0.962967
bomi vs zangga	ko04923	Regulation	2/198	90/8136	0.648248	0.962967	0.962967
bomi vs zangga	ko04925	Aldosteron	3/198	137/8136	0.652854	0.962967	0.962967
bomi vs zangga	ko04213	Longevity	2/198	91/8136	0.654227	0.962967	0.962967
bomi vs zangga	ko04218	Cellular se	4/198	183/8136	0.655691	0.962967	0.962967
bomi vs zangga	ko00250	Alanine, as	1/198	44/8136	0.662757	0.962967	0.962967
bomi vs zangga	ko04215	Apoptosis	1/198	44/8136	0.662757	0.962967	0.962967
bomi vs zangga	ko05415	NA	5/198	230/8136	0.663893	0.962967	0.962967
bomi vs zangga	ko04212	Longevity	2/198	93/8136	0.665943	0.962967	0.962967
bomi vs zangga	ko05207	NA	6/198	276/8136	0.668669	0.962967	0.962967
bomi vs zangga	ko04623	Cytosolic D	1/198	45/8136	0.671009	0.962967	0.962967
bomi vs zangga	ko04964	Proximal tu	1/198	45/8136	0.671009	0.962967	0.962967
bomi vs zangga	ko04060	Cytokine-c	5/198	232/8136	0.671358	0.962967	0.962967
bomi vs zangga	ko04917	Prolactin s	2/198	94/8136	0.67168	0.962967	0.962967
bomi vs zangga	ko00860	Porphyrin	1/198	46/8136	0.67906	0.962967	0.962967
bomi vs zangga	ko03460	Fanconi ar	1/198	46/8136	0.67906	0.962967	0.962967
bomi vs zangga	ko04140	Autophagy	4/198	189/8136	0.680486	0.962967	0.962967
bomi vs zangga	ko05219	Bladder ca	1/198	47/8136	0.686915	0.962967	0.962967
bomi vs zangga	ko00513	Various typ	1/198	48/8136	0.694579	0.962967	0.962967
bomi vs zangga	ko04973	Carbohydr	1/198	48/8136	0.694579	0.962967	0.962967
bomi vs zangga	ko04728	Dopamine	4/198	193/8136	0.696315	0.962967	0.962967
bomi vs zangga	ko04960	Aldosteron	1/198	49/8136	0.702055	0.962967	0.962967
bomi vs zangga	ko04371	Apelin sign	4/198	196/8136	0.707818	0.962967	0.962967
bomi vs zangga	ko04931	Insulin resi	3/198	149/8136	0.707964	0.962967	0.962967
bomi vs zangga	ko04920	Adipocyto	2/198	101/8136	0.70964	0.962967	0.962967
bomi vs zangga	ko00565	Ether lipid	1/198	51/8136	0.716467	0.962967	0.962967
bomi vs zangga	ko05216	Thyroid ca	1/198	51/8136	0.716467	0.962967	0.962967
bomi vs zangga	ko05205	Proteoglyc	6/198	291/8136	0.717539	0.962967	0.962967
bomi vs zangga	ko05171	NA	5/198	246/8136	0.720537	0.962967	0.962967
bomi vs zangga	ko03040	Spliceosor	3/198	152/8136	0.720652	0.962967	0.962967
bomi vs zangga	ko05417	NA	5/198	248/8136	0.72712	0.962967	0.962967
bomi vs zangga	ko04150	mTOR sign	4/198	202/8136	0.729873	0.962967	0.962967
bomi vs zangga	ko04924	Renin secr	2/198	106/8136	0.734465	0.962967	0.962967
bomi vs zangga	ko04120	Ubiquitin r	3/198	156/8136	0.736901	0.962967	0.962967
bomi vs zangga	ko04971	Gastric aci	2/198	107/8136	0.739209	0.962967	0.962967
bomi vs zangga	ko04022	cGMP-PKG	5/198	252/8136	0.739951	0.962967	0.962967
bomi vs zangga	ko05220	Chronic m	2/198	109/8136	0.748481	0.962967	0.962967

bomi vs zangga	ko04962	Vasopress	1/198	56/8136	0.749534	0.962967	0.962967
bomi vs zangga	ko05143	African try	1/198	56/8136	0.749534	0.962967	0.962967
bomi vs zangga	ko04350	TGF-beta s	2/198	110/8136	0.75301	0.962967	0.962967
bomi vs zangga	ko00982	Drug meta	1/198	57/8136	0.755672	0.962967	0.962967
bomi vs zangga	ko00510	N-Glycan	1/198	58/8136	0.76166	0.962967	0.962967
bomi vs zangga	ko00140	Steroid ho	1/198	59/8136	0.767502	0.962967	0.962967
bomi vs zangga	ko04216	Ferroptosi	1/198	59/8136	0.767502	0.962967	0.962967
bomi vs zangga	ko04014	Ras signali	6/198	309/8136	0.769378	0.962967	0.962967
bomi vs zangga	ko04730	Long-term	2/198	114/8136	0.770435	0.962967	0.962967
bomi vs zangga	ko04979	Cholestero	1/198	60/8136	0.773201	0.962967	0.962967
bomi vs zangga	ko04934	Cushing sy	4/198	215/8136	0.773368	0.962967	0.962967
bomi vs zangga	ko05100	Bacterial in	2/198	115/8136	0.774621	0.962967	0.962967
bomi vs zangga	ko00980	Metabolism	1/198	61/8136	0.778762	0.962967	0.962967
bomi vs zangga	ko04012	ErbB signa	2/198	117/8136	0.782795	0.962967	0.962967
bomi vs zangga	ko05204	Chemical c	1/198	62/8136	0.784186	0.962967	0.962967
bomi vs zangga	ko05212	Pancreatic	2/198	118/8136	0.786784	0.962967	0.962967
bomi vs zangga	ko04015	Rap1 signa	6/198	317/8136	0.790039	0.962967	0.962967
bomi vs zangga	ko03008	Ribosome	1/198	64/8136	0.794642	0.964437	0.964437
bomi vs zangga	ko04211	Longevity	2/198	122/8136	0.802105	0.966053	0.966053
bomi vs zangga	ko00480	Glutathion	1/198	66/8136	0.804594	0.966053	0.966053
bomi vs zangga	ko01521	EGFR tyros	2/198	124/8136	0.809395	0.966053	0.966053
bomi vs zangga	ko04725	Cholinergi	3/198	178/8136	0.813286	0.966053	0.966053
bomi vs zangga	ko04721	Synaptic v	2/198	126/8136	0.816446	0.966053	0.966053
bomi vs zangga	ko04611	Platelet ac	3/198	180/8136	0.819205	0.966053	0.966053
bomi vs zangga	ko04330	Notch sign	1/198	70/8136	0.82308	0.966053	0.966053
bomi vs zangga	ko04713	Circadian e	3/198	182/8136	0.824965	0.966053	0.966053
bomi vs zangga	ko04660	T cell rece	2/198	129/8136	0.826588	0.966053	0.966053
bomi vs zangga	ko00310	Lysine deg	1/198	72/8136	0.83166	0.967998	0.967998
bomi vs zangga	ko04137	Mitophagy	1/198	79/8136	0.858556	0.991163	0.991163
bomi vs zangga	ko05170	Human im	4/198	249/8136	0.861351	0.991163	0.991163
bomi vs zangga	ko01230	Biosynthes	1/198	80/8136	0.862032	0.991163	0.991163
bomi vs zangga	ko04066	HIF-1 sign	2/198	145/8136	0.87264	0.994839	0.994839
bomi vs zangga	ko05208	NA	4/198	256/8136	0.875344	0.994839	0.994839
bomi vs zangga	ko04810	Regulation	5/198	317/8136	0.890574	0.994839	0.994839
bomi vs zangga	ko03018	RNA degra	1/198	91/8136	0.895089	0.994839	0.994839
bomi vs zangga	ko05230	Central ca	1/198	95/8136	0.905044	0.994839	0.994839
bomi vs zangga	ko04928	Parathyroi	2/198	161/8136	0.907235	0.994839	0.994839
bomi vs zangga	ko04927	Cortisol sy	1/198	96/8136	0.907382	0.994839	0.994839
bomi vs zangga	ko05031	Amphetam	1/198	97/8136	0.909663	0.994839	0.994839
bomi vs zangga	ko04512	ECM-recep	1/198	98/8136	0.911888	0.994839	0.994839
bomi vs zangga	ko04929	GnRH secr	1/198	98/8136	0.911888	0.994839	0.994839
bomi vs zangga	ko04360	Axon guid	4/198	281/8136	0.915847	0.994839	0.994839
bomi vs zangga	ko05214	Glioma	1/198	100/8136	0.916176	0.994839	0.994839
bomi vs zangga	ko05235	PD-L1 exp	1/198	101/8136	0.918241	0.994839	0.994839
bomi vs zangga	ko00564	Glyceroph	1/198	104/8136	0.924138	0.994839	0.994839
bomi vs zangga	ko04910	Insulin sign	2/198	173/8136	0.927207	0.994839	0.994839
bomi vs zangga	ko03010	Ribosome	1/198	107/8136	0.929612	0.994839	0.994839
bomi vs zangga	ko05418	Fluid shear	2/198	181/8136	0.938198	0.994839	0.994839
bomi vs zangga	ko05218	Melanoma	1/198	114/8136	0.940904	0.994839	0.994839
bomi vs zangga	ko04650	Natural kil	1/198	116/8136	0.943785	0.994839	0.994839
bomi vs zangga	ko04915	Estrogen s	2/198	186/8136	0.94425	0.994839	0.994839
bomi vs zangga	ko05215	Prostate ca	1/198	126/8136	0.956225	0.994839	0.994839
bomi vs zangga	ko04141	Protein pro	2/198	199/8136	0.957467	0.994839	0.994839
bomi vs zangga	ko00190	Oxidative p	1/198	131/8136	0.961376	0.994839	0.994839
bomi vs zangga	ko05206	MicroRNA	2/198	204/8136	0.961707	0.994839	0.994839
bomi vs zangga	ko04110	Cell cycle	1/198	135/8136	0.965058	0.994839	0.994839
bomi vs zangga	ko04510	Focal adhe	3/198	276/8136	0.967035	0.994839	0.994839
bomi vs zangga	ko04922	Glucagon	1/198	143/8136	0.971408	0.994839	0.994839

bomi vs zangga	ko05160	Hepatitis C	2/198	218/8136	0.971537	0.994839	0.994839
bomi vs zangga	ko04142	Lysosome	1/198	150/8136	0.976014	0.994839	0.994839
bomi vs zangga	ko04152	AMPK sign	1/198	152/8136	0.977189	0.994839	0.994839
bomi vs zangga	ko05162	Measles	1/198	152/8136	0.977189	0.994839	0.994839
bomi vs zangga	ko05165	Human pa	5/198	417/8136	0.977324	0.994839	0.994839
bomi vs zangga	ko04919	Thyroid ho	1/198	180/8136	0.988721	0.996814	0.996814
bomi vs zangga	ko04020	Calcium si	4/198	398/8136	0.989206	0.996814	0.996814
bomi vs zangga	ko04024	cAMP sign	3/198	351/8136	0.992516	0.996814	0.996814
bomi vs zangga	ko04151	PI3K-Akt s	4/198	437/8136	0.994881	0.996814	0.996814
bomi vs zangga	ko05203	Viral carcin	1/198	230/8136	0.996814	0.996814	0.996814
milin vs linzhi	ko00053	Ascorbate	7/244	31/8136	2.84E-05	0.007961	0.007602
milin vs linzhi	ko04514	Cell adhes	20/244	267/8136	0.000144	0.020132	0.019224
milin vs linzhi	ko00860	Porphyrin	7/244	46/8136	0.000394	0.03681	0.03515
milin vs linzhi	ko00040	Pentose ar	6/244	37/8136	0.000728	0.050992	0.048691
milin vs linzhi	ko00982	Drug meta	7/244	57/8136	0.001473	0.082472	0.078751
milin vs linzhi	ko00980	Metabolism	7/244	61/8136	0.002196	0.091894	0.087749
milin vs linzhi	ko05204	Chemical c	7/244	62/8136	0.002413	0.091894	0.087749
milin vs linzhi	ko04940	Type I diak	8/244	80/8136	0.002626	0.091894	0.087749
milin vs linzhi	ko05332	Graft-vers	7/244	67/8136	0.003761	0.101302	0.096732
milin vs linzhi	ko00531	Glycosami	4/244	22/8136	0.003771	0.101302	0.096732
milin vs linzhi	ko03050	Proteasom	6/244	51/8136	0.00398	0.101302	0.096732
milin vs linzhi	ko00120	Primary bi	4/244	23/8136	0.004459	0.101639	0.097053
milin vs linzhi	ko05416	Viral myoc	10/244	128/8136	0.005009	0.101639	0.097053
milin vs linzhi	ko00983	Drug meta	8/244	89/8136	0.005082	0.101639	0.097053
milin vs linzhi	ko04670	Leukocyte	12/244	180/8136	0.007827	0.142903	0.136456
milin vs linzhi	ko00140	Steroid ho	6/244	59/8136	0.008166	0.142903	0.136456
milin vs linzhi	ko05033	Nicotine a	7/244	79/8136	0.009266	0.152611	0.145726
milin vs linzhi	ko04530	Tight junct	15/244	259/8136	0.011061	0.172059	0.164297
milin vs linzhi	ko03460	Fanconi ar	5/244	46/8136	0.011718	0.172685	0.164894
milin vs linzhi	ko04612	Antigen pr	8/244	106/8136	0.014082	0.192144	0.183476
milin vs linzhi	ko05330	Allograft r	7/244	86/8136	0.014411	0.192144	0.183476
milin vs linzhi	ko05320	Autoimmu	7/244	88/8136	0.016195	0.206124	0.196825
milin vs linzhi	ko01240	NA	10/244	166/8136	0.027492	0.334685	0.319586
milin vs linzhi	ko03060	Protein ex	3/244	26/8136	0.041677	0.486235	0.4643
milin vs linzhi	ko05017	Spinocerel	10/244	181/8136	0.045532	0.499069	0.476555
milin vs linzhi	ko04976	Bile secret	8/244	133/8136	0.046342	0.499069	0.476555
milin vs linzhi	ko00330	Arginine a	5/244	67/8136	0.050003	0.518547	0.495154
milin vs linzhi	ko00601	Glycosphir	3/244	30/8136	0.059686	0.575586	0.549619
milin vs linzhi	ko05160	Hepatitis C	11/244	218/8136	0.063348	0.575586	0.549619
milin vs linzhi	ko05145	Toxoplasm	9/244	167/8136	0.063651	0.575586	0.549619
milin vs linzhi	ko00280	Valine, leu	4/244	51/8136	0.065893	0.575586	0.549619
milin vs linzhi	ko00830	Retinol me	5/244	73/8136	0.067506	0.575586	0.549619
milin vs linzhi	ko05169	Epstein-Ba	12/244	247/8136	0.067837	0.575586	0.549619
milin vs linzhi	ko00514	Other type	4/244	53/8136	0.073785	0.607642	0.58023
milin vs linzhi	ko04260	Cardiac m	8/244	149/8136	0.07901	0.62788	0.599554
milin vs linzhi	ko01250	NA	3/244	34/8136	0.080727	0.62788	0.599554
milin vs linzhi	ko00515	Mannose t	2/244	17/8136	0.090688	0.686289	0.655329
milin vs linzhi	ko00340	Histidine n	2/244	20/8136	0.119599	0.84562	0.807472
milin vs linzhi	ko05168	Herpes sin	11/244	246/8136	0.120827	0.84562	0.807472
milin vs linzhi	ko05207	NA	12/244	276/8136	0.12588	0.84562	0.807472
milin vs linzhi	ko00533	Glycosami	2/244	21/8136	0.129685	0.84562	0.807472
milin vs linzhi	ko05144	Malaria	3/244	42/8136	0.130655	0.84562	0.807472
milin vs linzhi	ko05020	Prion disea	14/244	335/8136	0.130983	0.84562	0.807472
milin vs linzhi	ko04720	Long-term	5/244	90/8136	0.132883	0.84562	0.807472
milin vs linzhi	ko04212	Longevity	5/244	93/8136	0.14663	0.912364	0.871204
milin vs linzhi	ko00604	Glycosphir	2/244	23/8136	0.150404	0.915503	0.874202
milin vs linzhi	ko05014	Amyotropl	16/244	406/8136	0.159768	0.93892	0.896562
milin vs linzhi	ko05133	Pertussis	5/244	96/8136	0.160958	0.93892	0.896562

milin vs linzhi	ko04723	Retrograde	9/244	206/8136	0.166006	0.948606	0.905812
milin vs linzhi	ko00520	Amino sug	3/244	48/8136	0.173564	0.959316	0.916038
milin vs linzhi	ko05410	Hypertrop	7/244	154/8136	0.179786	0.959316	0.916038
milin vs linzhi	ko00760	Nicotinate	3/244	49/8136	0.18107	0.959316	0.916038
milin vs linzhi	ko05140	Leishmania	6/244	127/8136	0.181585	0.959316	0.916038
milin vs linzhi	ko05134	Legionello	4/244	75/8136	0.187366	0.97153	0.927701
milin vs linzhi	ko00650	Butanoate	2/244	27/8136	0.193491	0.977103	0.933023
milin vs linzhi	ko05167	Kaposi sar	9/244	215/8136	0.196953	0.977103	0.933023
milin vs linzhi	ko05012	Parkinson	12/244	303/8136	0.19891	0.977103	0.933023
milin vs linzhi	ko05170	Human im	10/244	249/8136	0.214406	0.999229	0.954151
milin vs linzhi	ko04145	Phagosom	9/244	222/8136	0.222597	0.999229	0.954151
milin vs linzhi	ko04022	cGMP-PK(	10/244	252/8136	0.224916	0.999229	0.954151
milin vs linzhi	ko04918	Thyroid ho	4/244	82/8136	0.231338	0.999229	0.954151
milin vs linzhi	ko00220	Arginine b	2/244	31/8136	0.237901	0.999229	0.954151
milin vs linzhi	ko00410	beta-Alani	2/244	32/8136	0.249105	0.999229	0.954151
milin vs linzhi	ko05322	Systemic lu	7/244	174/8136	0.267186	0.999229	0.954151
milin vs linzhi	ko04750	Inflammat	6/244	146/8136	0.273846	0.999229	0.954151
milin vs linzhi	ko04261	Adrenergic	9/244	236/8136	0.277326	0.999229	0.954151
milin vs linzhi	ko04392	Hippo sign	2/244	35/8136	0.282769	0.999229	0.954151
milin vs linzhi	ko05321	Inflammat	4/244	90/8136	0.284262	0.999229	0.954151
milin vs linzhi	ko05414	Dilated car	7/244	179/8136	0.290715	0.999229	0.954151
milin vs linzhi	ko04640	Hematopo	5/244	120/8136	0.291517	0.999229	0.954151
milin vs linzhi	ko04142	Lysosome	6/244	150/8136	0.294625	0.999229	0.954151
milin vs linzhi	ko04919	Thyroid ho	7/244	180/8136	0.295479	0.999229	0.954151
milin vs linzhi	ko03008	Ribosome	3/244	64/8136	0.301034	0.999229	0.954151
milin vs linzhi	ko00430	Taurine an	1/244	12/8136	0.306247	0.999229	0.954151
milin vs linzhi	ko04020	Calcium si	14/244	398/8136	0.307041	0.999229	0.954151
milin vs linzhi	ko05010	Alzheimer	16/244	462/8136	0.311218	0.999229	0.954151
milin vs linzhi	ko04146	Peroxisom	4/244	96/8136	0.325055	0.999229	0.954151
milin vs linzhi	ko04120	Ubiquitin r	6/244	156/8136	0.326343	0.999229	0.954151
milin vs linzhi	ko05417	NA	9/244	248/8136	0.326995	0.999229	0.954151
milin vs linzhi	ko05340	Primary im	2/244	39/8136	0.327408	0.999229	0.954151
milin vs linzhi	ko05016	Huntingto	12/244	343/8136	0.333033	0.999229	0.954151
milin vs linzhi	ko04961	Endocrine	3/244	69/8136	0.342391	0.999229	0.954151
milin vs linzhi	ko04727	GABAergic	5/244	129/8136	0.344868	0.999229	0.954151
milin vs linzhi	ko00380	Tryptopha	2/244	41/8136	0.34947	0.999229	0.954151
milin vs linzhi	ko05030	Cocaine ad	3/244	70/8136	0.350655	0.999229	0.954151
milin vs linzhi	ko04726	Serotonerg	6/244	161/8136	0.353123	0.999229	0.954151
milin vs linzhi	ko04015	Rap1 signa	11/244	317/8136	0.353246	0.999229	0.954151
milin vs linzhi	ko05208	NA	9/244	256/8136	0.361025	0.999229	0.954151
milin vs linzhi	ko04742	Taste trans	3/244	72/8136	0.367146	0.999229	0.954151
milin vs linzhi	ko04371	Apelin sign	7/244	196/8136	0.373497	0.999229	0.954151
milin vs linzhi	ko04621	NOD-like	6/244	165/8136	0.374674	0.999229	0.954151
milin vs linzhi	ko00730	Thiamine r	1/244	16/8136	0.385927	0.999229	0.954151
milin vs linzhi	ko04068	FoxO signa	6/244	168/8136	0.390867	0.999229	0.954151
milin vs linzhi	ko04964	Proximal t	2/244	45/8136	0.3928	0.999229	0.954151
milin vs linzhi	ko04925	Aldosteron	5/244	137/8136	0.392819	0.999229	0.954151
milin vs linzhi	ko05323	Rheumato	5/244	138/8136	0.398808	0.999229	0.954151
milin vs linzhi	ko04971	Gastric aci	4/244	107/8136	0.400434	0.999229	0.954151
milin vs linzhi	ko00512	Mucin type	2/244	46/8136	0.403435	0.999229	0.954151
milin vs linzhi	ko04978	Mineral ab	3/244	77/8136	0.408005	0.999229	0.954151
milin vs linzhi	ko05034	Alcoholism	7/244	205/8136	0.418043	0.999229	0.954151
milin vs linzhi	ko05222	Small cell l	4/244	110/8136	0.420829	0.999229	0.954151
milin vs linzhi	ko00600	Sphingolip	3/244	79/8136	0.42414	0.999229	0.954151
milin vs linzhi	ko04137	Mitophagy	3/244	79/8136	0.42414	0.999229	0.954151
milin vs linzhi	ko00513	Various typ	2/244	48/8136	0.424434	0.999229	0.954151
milin vs linzhi	ko04973	Carbohydr	2/244	48/8136	0.424434	0.999229	0.954151
milin vs linzhi	ko04974	Protein dig	4/244	112/8136	0.434337	0.999229	0.954151

milin vs linzhi	ko04960	Aldosteror	2/244	49/8136	0.43479	0.999229	0.954151
milin vs linzhi	ko01524	Platinum c	3/244	81/8136	0.440123	0.999229	0.954151
milin vs linzhi	ko04064	NF-kappa	4/244	114/8136	0.447759	0.999229	0.954151
milin vs linzhi	ko04730	Long-term	4/244	114/8136	0.447759	0.999229	0.954151
milin vs linzhi	ko04970	Salivary se	4/244	114/8136	0.447759	0.999229	0.954151
milin vs linzhi	ko05202	Transcripti	8/244	247/8136	0.46356	0.999229	0.954151
milin vs linzhi	ko04713	Circadian e	6/244	182/8136	0.465946	0.999229	0.954151
milin vs linzhi	ko00563	Glycosylph	1/244	21/8136	0.472829	0.999229	0.954151
milin vs linzhi	ko02010	ABC transp	2/244	54/8136	0.484999	0.999229	0.954151
milin vs linzhi	ko04217	Necroptos	7/244	219/8136	0.486651	0.999229	0.954151
milin vs linzhi	ko04391	Hippo sigr	3/244	87/8136	0.486979	0.999229	0.954151
milin vs linzhi	ko05163	Human cy	9/244	292/8136	0.515325	0.999229	0.954151
milin vs linzhi	ko04213	Longevity	3/244	91/8136	0.517149	0.999229	0.954151
milin vs linzhi	ko04658	Th1 and Tl	4/244	125/8136	0.519506	0.999229	0.954151
milin vs linzhi	ko05231	Choline m	5/244	159/8136	0.521478	0.999229	0.954151
milin vs linzhi	ko00510	N-Glycan	2/244	58/8136	0.523128	0.999229	0.954151
milin vs linzhi	ko04728	Dopamine	6/244	193/8136	0.52324	0.999229	0.954151
milin vs linzhi	ko04672	Intestinal i	3/244	92/8136	0.524543	0.999229	0.954151
milin vs linzhi	ko04721	Synaptic v	4/244	126/8136	0.525819	0.999229	0.954151
milin vs linzhi	ko04740	Olfactory t	3/244	93/8136	0.531876	0.999229	0.954151
milin vs linzhi	ko05146	Amoebiasi	4/244	127/8136	0.532092	0.999229	0.954151
milin vs linzhi	ko00511	Other glyc	1/244	25/8136	0.53344	0.999229	0.954151
milin vs linzhi	ko05142	Chagas dis	4/244	128/8136	0.538324	0.999229	0.954151
milin vs linzhi	ko05203	Viral carcir	7/244	230/8136	0.538924	0.999229	0.954151
milin vs linzhi	ko05412	Arrhythmo	4/244	129/8136	0.544515	0.999229	0.954151
milin vs linzhi	ko05032	Morphine	4/244	130/8136	0.550663	0.999229	0.954151
milin vs linzhi	ko04141	Protein pro	6/244	199/8136	0.553464	0.999229	0.954151
milin vs linzhi	ko04927	Cortisol sy	3/244	96/8136	0.553487	0.999229	0.954151
milin vs linzhi	ko04668	TNF signal	4/244	131/8136	0.556768	0.999229	0.954151
milin vs linzhi	ko04622	RIG-I-like	2/244	62/8136	0.559324	0.999229	0.954151
milin vs linzhi	ko05310	Asthma	2/244	62/8136	0.559324	0.999229	0.954151
milin vs linzhi	ko05031	Amphetan	3/244	97/8136	0.560559	0.999229	0.954151
milin vs linzhi	ko00640	Propanoat	1/244	27/8136	0.56109	0.999229	0.954151
milin vs linzhi	ko04512	ECM-recep	3/244	98/8136	0.567563	0.999229	0.954151
milin vs linzhi	ko00630	Glyoxylate	1/244	28/8136	0.574297	0.999229	0.954151
milin vs linzhi	ko04930	Type II dia	2/244	64/8136	0.576677	0.999229	0.954151
milin vs linzhi	ko04110	Cell cycle	4/244	135/8136	0.580736	0.999229	0.954151
milin vs linzhi	ko04520	Adherens	4/244	135/8136	0.580736	0.999229	0.954151
milin vs linzhi	ko04014	Ras signali	9/244	309/8136	0.584538	0.999229	0.954151
milin vs linzhi	ko03013	RNA trans	3/244	101/8136	0.588162	0.999229	0.954151
milin vs linzhi	ko00480	Glutathion	2/244	66/8136	0.593528	0.999229	0.954151
milin vs linzhi	ko00500	Starch and	1/244	30/8136	0.599535	0.999229	0.954151
milin vs linzhi	ko05152	Tuberculos	7/244	245/8136	0.606474	0.999229	0.954151
milin vs linzhi	ko00564	Glyceroph	3/244	104/8136	0.608125	0.999229	0.954151
milin vs linzhi	ko04625	C-type lec	4/244	141/8136	0.615266	0.999229	0.954151
milin vs linzhi	ko04924	Renin secr	3/244	106/8136	0.621073	0.999229	0.954151
milin vs linzhi	ko05166	Human T-	8/244	284/8136	0.62308	0.999229	0.954151
milin vs linzhi	ko00030	Pentose pl	1/244	32/8136	0.623283	0.999229	0.954151
milin vs linzhi	ko04966	Collecting	1/244	32/8136	0.623283	0.999229	0.954151
milin vs linzhi	ko04611	Platelet ac	5/244	180/8136	0.632306	0.999229	0.954151
milin vs linzhi	ko04066	HIF-1 sign	4/244	145/8136	0.637286	0.999229	0.954151
milin vs linzhi	ko00310	Lysine deg	2/244	72/8136	0.64106	0.999229	0.954151
milin vs linzhi	ko00592	alpha-Linc	1/244	34/8136	0.645627	0.999229	0.954151
milin vs linzhi	ko04218	Cellular se	5/244	183/8136	0.646817	0.999229	0.954151
milin vs linzhi	ko05135	Yersinia in	6/244	219/8136	0.647212	0.999229	0.954151
milin vs linzhi	ko04070	Phosphatic	3/244	111/8136	0.652159	0.999229	0.954151
milin vs linzhi	ko04931	Insulin resi	4/244	149/8136	0.658479	0.999229	0.954151
milin vs linzhi	ko04144	Endocytos	9/244	329/8136	0.659722	0.999229	0.954151

milin vs linzhi	ko00010	Glycolysis	2/244	75/8136	0.663143	0.999229	0.954151
milin vs linzhi	ko04151	PI3K-Akt s	12/244	437/8136	0.666501	0.999229	0.954151
milin vs linzhi	ko00591	Linoleic ac	1/244	36/8136	0.666652	0.999229	0.954151
milin vs linzhi	ko04130	SNARE inte	1/244	36/8136	0.666652	0.999229	0.954151
milin vs linzhi	ko04136	Autophagy	1/244	36/8136	0.666652	0.999229	0.954151
milin vs linzhi	ko00240	Pyrimidine	2/244	76/8136	0.670258	0.999229	0.954151
milin vs linzhi	ko04724	Glutamate	5/244	189/8136	0.674749	0.999229	0.954151
milin vs linzhi	ko04914	Progestero	3/244	116/8136	0.681391	0.999229	0.954151
milin vs linzhi	ko04071	Sphingolip	5/244	193/8136	0.692544	0.999229	0.954151
milin vs linzhi	ko00970	Aminoacyl	1/244	39/8136	0.695882	0.999229	0.954151
milin vs linzhi	ko00590	Arachidon	2/244	80/8136	0.69751	0.999229	0.954151
milin vs linzhi	ko04664	Fc epsilon	3/244	119/8136	0.698039	0.999229	0.954151
milin vs linzhi	ko04926	Relaxin sig	4/244	157/8136	0.698322	0.999229	0.954151
milin vs linzhi	ko05165	Human pa	11/244	417/8136	0.713446	0.999229	0.954151
milin vs linzhi	ko00620	Pyruvate n	1/244	42/8136	0.722558	0.999229	0.954151
milin vs linzhi	ko03420	Nucleotide	1/244	42/8136	0.722558	0.999229	0.954151
milin vs linzhi	ko04913	Ovarian st	2/244	85/8136	0.728935	0.999229	0.954151
milin vs linzhi	ko00071	Fatty acid	1/244	43/8136	0.730922	0.999229	0.954151
milin vs linzhi	ko00534	Glycosami	1/244	43/8136	0.730922	0.999229	0.954151
milin vs linzhi	ko04024	cAMP sign	9/244	351/8136	0.732697	0.999229	0.954151
milin vs linzhi	ko00051	Fructose a	1/244	44/8136	0.739034	0.999229	0.954151
milin vs linzhi	ko00250	Alanine, as	1/244	44/8136	0.739034	0.999229	0.954151
milin vs linzhi	ko04215	Apoptosis	1/244	44/8136	0.739034	0.999229	0.954151
milin vs linzhi	ko04610	Compleme	2/244	87/8136	0.740711	0.999229	0.954151
milin vs linzhi	ko04210	Apoptosis	5/244	205/8136	0.741891	0.999229	0.954151
milin vs linzhi	ko01523	Antifolate	1/244	46/8136	0.754536	0.999229	0.954151
milin vs linzhi	ko04923	Regulation	2/244	90/8136	0.757558	0.999229	0.954151
milin vs linzhi	ko00190	Oxidative	3/244	131/8136	0.758067	0.999229	0.954151
milin vs linzhi	ko04380	Osteoclast	3/244	131/8136	0.758067	0.999229	0.954151
milin vs linzhi	ko03018	RNA degra	2/244	91/8136	0.76296	0.999229	0.954151
milin vs linzhi	ko05164	Influenza A	5/244	211/8136	0.764294	0.999229	0.954151
milin vs linzhi	ko04910	Insulin sigr	4/244	173/8136	0.76777	0.999229	0.954151
milin vs linzhi	ko04657	IL-17 signa	2/244	92/8136	0.768258	0.999229	0.954151
milin vs linzhi	ko04114	Oocyte me	3/244	134/8136	0.771482	0.999229	0.954151
milin vs linzhi	ko04917	Prolactin s	2/244	94/8136	0.778547	0.999229	0.954151
milin vs linzhi	ko04725	Cholinergi	4/244	178/8136	0.786737	0.999229	0.954151
milin vs linzhi	ko00565	Ether lipid	1/244	51/8136	0.789394	0.999229	0.954151
milin vs linzhi	ko04624	Toll and In	1/244	51/8136	0.789394	0.999229	0.954151
milin vs linzhi	ko05418	Fluid shear	4/244	181/8136	0.797515	0.999229	0.954151
milin vs linzhi	ko04972	Pancreatic	3/244	141/8136	0.800434	0.999229	0.954151
milin vs linzhi	ko04620	Toll-like re	2/244	100/8136	0.807055	0.999229	0.954151
milin vs linzhi	ko04922	Glucagon	3/244	143/8136	0.808122	0.999229	0.954151
milin vs linzhi	ko04920	Adipocyto	2/244	101/8136	0.811477	0.999229	0.954151
milin vs linzhi	ko05235	PD-L1 exp	2/244	101/8136	0.811477	0.999229	0.954151
milin vs linzhi	ko04630	JAK-STAT	3/244	144/8136	0.811873	0.999229	0.954151
milin vs linzhi	ko04540	Gap juncti	3/244	145/8136	0.815561	0.999229	0.954151
milin vs linzhi	ko04962	Vasopress	1/244	56/8136	0.81932	0.999229	0.954151
milin vs linzhi	ko05143	African try	1/244	56/8136	0.81932	0.999229	0.954151
milin vs linzhi	ko04013	MAPK sign	2/244	103/8136	0.820052	0.999229	0.954151
milin vs linzhi	ko04659	Th17 cell d	3/244	148/8136	0.826262	0.999229	0.954151
milin vs linzhi	ko04214	Apoptosis	1/244	59/8136	0.835201	0.999229	0.954151
milin vs linzhi	ko04662	B cell rece	2/244	107/8136	0.836163	0.999229	0.954151
milin vs linzhi	ko03040	Spliceosor	3/244	152/8136	0.839704	0.999229	0.954151
milin vs linzhi	ko05162	Measles	3/244	152/8136	0.839704	0.999229	0.954151
milin vs linzhi	ko04010	MAPK sign	10/244	431/8136	0.840386	0.999229	0.954151
milin vs linzhi	ko04666	Fc gamma	3/244	153/8136	0.842921	0.999229	0.954151
milin vs linzhi	ko00561	Glycerolipi	1/244	61/8136	0.845008	0.999229	0.954151
milin vs linzhi	ko05132	Salmonella	7/244	318/8136	0.847135	0.999229	0.954151

milin vs linzhi	ko04360	Axon guid	6/244	281/8136	0.853471	0.999229	0.954151
milin vs linzhi	ko00230	Purine me	3/244	158/8136	0.858186	0.999229	0.954151
milin vs linzhi	ko04613	NA	4/244	202/8136	0.861187	0.999229	0.954151
milin vs linzhi	ko04932	Non-alcoh	4/244	203/8136	0.863742	0.999229	0.954151
milin vs linzhi	ko04722	Neurotrop	3/244	160/8136	0.86392	0.999229	0.954151
milin vs linzhi	ko05100	Bacterial ir	2/244	115/8136	0.864541	0.999229	0.954151
milin vs linzhi	ko05206	MicroRNA	4/244	204/8136	0.866257	0.999229	0.954151
milin vs linzhi	ko04714	Thermoge	5/244	246/8136	0.866631	0.999229	0.954151
milin vs linzhi	ko04650	Natural kil	2/244	116/8136	0.867754	0.999229	0.954151
milin vs linzhi	ko04012	ErbB signa	2/244	117/8136	0.870897	0.999229	0.954151
milin vs linzhi	ko05150	Staphyloco	2/244	118/8136	0.873972	0.999229	0.954151
milin vs linzhi	ko05212	Pancreatic	2/244	118/8136	0.873972	0.999229	0.954151
milin vs linzhi	ko05205	Proteoglyc	6/244	291/8136	0.875119	0.999229	0.954151
milin vs linzhi	ko04330	Notch sigr	1/244	70/8136	0.882423	0.999229	0.954151
milin vs linzhi	ko01521	EGFR tyros	2/244	124/8136	0.89105	0.999229	0.954151
milin vs linzhi	ko04361	Axon rege	2/244	124/8136	0.89105	0.999229	0.954151
milin vs linzhi	ko05221	Acute mye	1/244	73/8136	0.892775	0.999229	0.954151
milin vs linzhi	ko04935	Growth ho	3/244	174/8136	0.898657	0.999229	0.954151
milin vs linzhi	ko01522	Endocrine	2/244	130/8136	0.905965	0.999229	0.954151
milin vs linzhi	ko04933	AGE-RAGE	2/244	130/8136	0.905965	0.999229	0.954151
milin vs linzhi	ko04912	GnRH sign	2/244	132/8136	0.910499	0.999229	0.954151
milin vs linzhi	ko05210	Colorectal	2/244	134/8136	0.914828	0.999229	0.954151
milin vs linzhi	ko04810	Regulation	6/244	317/8136	0.919246	0.999229	0.954151
milin vs linzhi	ko04911	Insulin sec	2/244	137/8136	0.920956	0.999229	0.954151
milin vs linzhi	ko04936	NA	2/244	138/8136	0.922905	0.999229	0.954151
milin vs linzhi	ko00562	Inositol ph	1/244	84/8136	0.923546	0.999229	0.954151
milin vs linzhi	ko04061	Viral prote	1/244	84/8136	0.923546	0.999229	0.954151
milin vs linzhi	ko04140	Autophagy	3/244	189/8136	0.92686	0.999229	0.954151
milin vs linzhi	ko03015	mRNA sur	1/244	86/8136	0.928109	0.999229	0.954151
milin vs linzhi	ko05171	NA	4/244	246/8136	0.941373	0.999229	0.954151
milin vs linzhi	ko04150	mTOR sign	3/244	202/8136	0.945293	0.999229	0.954151
milin vs linzhi	ko05230	Central ca	1/244	95/8136	0.945511	0.999229	0.954151
milin vs linzhi	ko04152	AMPK sign	2/244	152/8136	0.945856	0.999229	0.954151
milin vs linzhi	ko04929	GnRH secr	1/244	98/8136	0.950323	0.999229	0.954151
milin vs linzhi	ko04370	VEGF signa	1/244	99/8136	0.951831	0.999229	0.954151
milin vs linzhi	ko04310	Wnt signal	3/244	209/8136	0.953339	0.999229	0.954151
milin vs linzhi	ko04928	Parathyroi	2/244	161/8136	0.95701	0.999229	0.954151
milin vs linzhi	ko05225	Hepatocel	3/244	214/8136	0.958397	0.999229	0.954151
milin vs linzhi	ko05211	Renal cell	1/244	104/8136	0.958714	0.999229	0.954151
milin vs linzhi	ko04934	Cushing sy	3/244	215/8136	0.959345	0.999229	0.954151
milin vs linzhi	ko04350	TGF-beta	1/244	110/8136	0.965692	0.999229	0.954151
milin vs linzhi	ko04072	Phospholi	3/244	224/8136	0.967014	0.999229	0.954151
milin vs linzhi	ko04062	Chemokin	3/244	227/8136	0.969252	0.999229	0.954151
milin vs linzhi	ko05218	Melanoma	1/244	114/8136	0.969679	0.999229	0.954151
milin vs linzhi	ko05415	NA	3/244	230/8136	0.971348	0.999229	0.954151
milin vs linzhi	ko04060	Cytokine-c	3/244	232/8136	0.972669	0.999229	0.954151
milin vs linzhi	ko05161	Hepatitis B	2/244	182/8136	0.975138	0.999229	0.954151
milin vs linzhi	ko01200	Carbon me	1/244	121/8136	0.975576	0.999229	0.954151
milin vs linzhi	ko04211	Longevity	1/244	122/8136	0.97632	0.999229	0.954151
milin vs linzhi	ko04915	Estrogen s	2/244	186/8136	0.97763	0.999229	0.954151
milin vs linzhi	ko04660	T cell rece	1/244	129/8136	0.98093	0.999229	0.954151
milin vs linzhi	ko04270	Vascular sı	2/244	203/8136	0.985784	0.999229	0.954151
milin vs linzhi	ko05226	Gastric car	2/244	206/8136	0.986886	0.999229	0.954151
milin vs linzhi	ko05224	Breast can	2/244	209/8136	0.987905	0.999229	0.954151
milin vs linzhi	ko04510	Focal adhe	3/244	276/8136	0.990605	0.999229	0.954151
milin vs linzhi	ko04550	Signaling p	1/244	195/8136	0.997547	0.999229	0.954151
milin vs linzhi	ko04390	Hippo sign	1/244	196/8136	0.997623	0.999229	0.954151
milin vs linzhi	ko04921	Oxytocin s	1/244	232/8136	0.999229	0.999229	0.954151

milin vs zangga	ko00860	Porphyrin	6/219	46/8136	0.001353	0.241593	0.241593	
milin vs zangga	ko04061	Viral prote	8/219	84/8136	0.001823	0.241593	0.241593	
milin vs zangga	ko03060	Protein ex	4/219	26/8136	0.004794	0.423448	0.423448	
milin vs zangga	ko04623	Cytosolic D	5/219	45/8136	0.006868	0.445235	0.445235	
milin vs zangga	ko00053	Ascorbate	4/219	31/8136	0.009087	0.445235	0.445235	
milin vs zangga	ko04530	Tight junct	14/219	259/8136	0.010081	0.445235	0.445235	
milin vs zangga	ko04621	NOD-like	10/219	165/8136	0.013501	0.447378	0.447378	
milin vs zangga	ko00830	Retinol me	6/219	73/8136	0.013506	0.447378	0.447378	
milin vs zangga	ko00040	Pentose ar	4/219	37/8136	0.016832	0.495619	0.495619	
milin vs zangga	ko04062	Chemokin	12/219	227/8136	0.019477	0.516132	0.516132	
milin vs zangga	ko04060	Cytokine-c	12/219	232/8136	0.022665	0.546025	0.546025	
milin vs zangga	ko00512	Mucin type	4/219	46/8136	0.034529	0.762524	0.762524	
milin vs zangga	ko05133	Pertussis	6/219	96/8136	0.044477	0.906639	0.906639	
milin vs zangga	ko00982	Drug meta	4/219	57/8136	0.066742	0.996905	0.996905	
milin vs zangga	ko00140	Steroid ho	4/219	59/8136	0.073838	0.996905	0.996905	
milin vs zangga	ko00980	Metabolism	4/219	61/8136	0.081302	0.996905	0.996905	
milin vs zangga	ko05204	Chemical c	4/219	62/8136	0.085168	0.996905	0.996905	
milin vs zangga	ko04064	NF-kappa	6/219	114/8136	0.086761	0.996905	0.996905	
milin vs zangga	ko03420	Nucleotide	3/219	42/8136	0.102852	0.996905	0.996905	
milin vs zangga	ko05135	Yersinia in	9/219	219/8136	0.136323	0.996905	0.996905	
milin vs zangga	ko00513	Various ty	3/219	48/8136	0.138308	0.996905	0.996905	
milin vs zangga	ko05132	Salmonella	12/219	318/8136	0.149276	0.996905	0.996905	
milin vs zangga	ko04072	Phospholip	9/219	224/8136	0.150078	0.996905	0.996905	
milin vs zangga	ko03020	RNA polym	2/219	26/8136	0.154106	0.996905	0.996905	
milin vs zangga	ko04520	Adherens	6/219	135/8136	0.156314	0.996905	0.996905	
milin vs zangga	ko01240	NA	7/219	166/8136	0.160187	0.996905	0.996905	
milin vs zangga	ko05033	Nicotine a	4/219	79/8136	0.163237	0.996905	0.996905	
milin vs zangga	ko04936	NA	6/219	138/8136	0.167836	0.996905	0.996905	
milin vs zangga	ko00514	Other type	3/219	53/8136	0.170544	0.996905	0.996905	
milin vs zangga	ko04921	Oxytocin s	9/219	232/8136	0.173462	0.996905	0.996905	
milin vs zangga	ko04625	C-type lec	6/219	141/8136	0.179706	0.996905	0.996905	
milin vs zangga	ko04972	Pancreatic	6/219	141/8136	0.179706	0.996905	0.996905	
milin vs zangga	ko04914	Progestero	5/219	116/8136	0.202447	0.996905	0.996905	
milin vs zangga	ko04725	Cholinergi	7/219	178/8136	0.203724	0.996905	0.996905	
milin vs zangga	ko00510	N-Glycan	3/219	58/8136	0.204674	0.996905	0.996905	
milin vs zangga	ko05207	NA	10/219	276/8136	0.20995	0.996905	0.996905	
milin vs zangga	ko00983	Drug meta	4/219	89/8136	0.217884	0.996905	0.996905	
milin vs zangga	ko04622	RIG-I-like	3/219	62/8136	0.233007	0.996905	0.996905	
milin vs zangga	ko04212	Longevity	4/219	93/8136	0.241004	0.996905	0.996905	
milin vs zangga	ko03410	Base excisi	2/219	35/8136	0.242572	0.996905	0.996905	
milin vs zangga	ko04392	Hippo sign	2/219	35/8136	0.242572	0.996905	0.996905	
milin vs zangga	ko04930	Type II dia	3/219	64/8136	0.247432	0.996905	0.996905	
milin vs zangga	ko05231	Choline m	6/219	159/8136	0.257102	0.996905	0.996905	
milin vs zangga	ko03450	Non-hom	1/219	11/8136	0.259431	0.996905	0.996905	
milin vs zangga	ko05032	Morphine	5/219	130/8136	0.272286	0.996905	0.996905	
milin vs zangga	ko04620	Toll-like re	4/219	100/8136	0.282674	0.996905	0.996905	
milin vs zangga	ko04977	Vitamin di	2/219	39/8136	0.282796	0.996905	0.996905	
milin vs zangga	ko04976	Bile secret	5/219	133/8136	0.287912	0.996905	0.996905	
milin vs zangga	ko04920	Adipocyto	4/219	101/8136	0.288722	0.996905	0.996905	
milin vs zangga	ko04141	Protein pr	7/219	199/8136	0.288896	0.996905	0.996905	
milin vs zangga	ko03022	Basal trans	2/219	40/8136	0.29284	0.996905	0.996905	
milin vs zangga	ko04114	Oocyte me	5/219	134/8136	0.293159	0.996905	0.996905	
milin vs zangga	ko04068	FoxO signa	6/219	168/8136	0.298794	0.996905	0.996905	
milin vs zangga	ko00450	Selenocom	1/219	13/8136	0.298818	0.996905	0.996905	
milin vs zangga	ko04210	Apoptosis	7/219	205/8136	0.314703	0.996905	0.996905	
milin vs zangga	ko04723	Retrograde	7/219	206/8136	0.319048	0.996905	0.996905	
milin vs zangga	ko05171	NA	8/219	246/8136	0.343389	0.996905	0.996905	
milin vs zangga	ko04630	JAK-STAT	5/219	144/8136	0.346357	0.996905	0.996905	

milin vs zangga	ko05417	NA	8/219	248/8136	0.35156	0.996905	0.996905
milin vs zangga	ko01523	Antifolate	2/219	46/8136	0.352507	0.996905	0.996905
milin vs zangga	ko03460	Fanconi ar	2/219	46/8136	0.352507	0.996905	0.996905
milin vs zangga	ko00730	Thiamine r	1/219	16/8136	0.354022	0.996905	0.996905
milin vs zangga	ko04974	Protein dig	4/219	112/8136	0.356081	0.996905	0.996905
milin vs zangga	ko04750	Inflammato	5/219	146/8136	0.357099	0.996905	0.996905
milin vs zangga	ko05017	Spinocerel	6/219	181/8136	0.360963	0.996905	0.996905
milin vs zangga	ko04940	Type I diab	3/219	80/8136	0.365485	0.996905	0.996905
milin vs zangga	ko04713	Circadian e	6/219	182/8136	0.365797	0.996905	0.996905
milin vs zangga	ko04659	Th17 cell d	5/219	148/8136	0.367854	0.996905	0.996905
milin vs zangga	ko04022	cGMP-PKG	8/219	252/8136	0.367972	0.996905	0.996905
milin vs zangga	ko04730	Long-term	4/219	114/8136	0.368391	0.996905	0.996905
milin vs zangga	ko00061	Fatty acid	1/219	17/8136	0.371444	0.996905	0.996905
milin vs zangga	ko05160	Hepatitis C	7/219	218/8136	0.371841	0.996905	0.996905
milin vs zangga	ko04217	Necroptos	7/219	219/8136	0.376276	0.996905	0.996905
milin vs zangga	ko04142	Lysosome	5/219	150/8136	0.378614	0.996905	0.996905
milin vs zangga	ko00670	One carbo	1/219	18/8136	0.388399	0.996905	0.996905
milin vs zangga	ko03040	Spliceosor	5/219	152/8136	0.389371	0.996905	0.996905
milin vs zangga	ko04144	Endocytos	10/219	329/8136	0.39284	0.996905	0.996905
milin vs zangga	ko04666	Fc gamma	5/219	153/8136	0.394746	0.996905	0.996905
milin vs zangga	ko05410	Hypertrop	5/219	154/8136	0.400118	0.996905	0.996905
milin vs zangga	ko03050	Proteasom	2/219	51/8136	0.400884	0.996905	0.996905
milin vs zangga	ko04975	Fat digesti	2/219	51/8136	0.400884	0.996905	0.996905
milin vs zangga	ko04120	Ubiquitin r	5/219	156/8136	0.410846	0.996905	0.996905
milin vs zangga	ko04391	Hippo sigr	3/219	87/8136	0.416693	0.996905	0.996905
milin vs zangga	ko04610	Compleme	3/219	87/8136	0.416693	0.996905	0.996905
milin vs zangga	ko00563	Glycosylph	1/219	21/8136	0.436579	0.996905	0.996905
milin vs zangga	ko03430	Mismatch	1/219	21/8136	0.436579	0.996905	0.996905
milin vs zangga	ko04923	Regulation	3/219	90/8136	0.438253	0.996905	0.996905
milin vs zangga	ko05143	African try	2/219	56/8136	0.447511	0.996905	0.996905
milin vs zangga	ko01210	2-Oxocarb	1/219	22/8136	0.451784	0.996905	0.996905
milin vs zangga	ko04657	IL-17 signa	3/219	92/8136	0.452459	0.996905	0.996905
milin vs zangga	ko05142	Chagas dis	4/219	128/8136	0.453595	0.996905	0.996905
milin vs zangga	ko05412	Arrhythmo	4/219	129/8136	0.459562	0.996905	0.996905
milin vs zangga	ko00120	Primary bi	1/219	23/8136	0.46658	0.996905	0.996905
milin vs zangga	ko04380	Osteoclast	4/219	131/8136	0.47143	0.996905	0.996905
milin vs zangga	ko04668	TNF signal	4/219	131/8136	0.47143	0.996905	0.996905
milin vs zangga	ko04214	Apoptosis	2/219	59/8136	0.474501	0.996905	0.996905
milin vs zangga	ko04929	GnRH secr	3/219	98/8136	0.494144	0.996905	0.996905
milin vs zangga	ko04110	Cell cycle	4/219	135/8136	0.49487	0.996905	0.996905
milin vs zangga	ko00511	Other glyc	1/219	25/8136	0.494991	0.996905	0.996905
milin vs zangga	ko00020	Citrate cyc	1/219	26/8136	0.508627	0.996905	0.996905
milin vs zangga	ko05235	PD-L1 exp	3/219	101/8136	0.514395	0.996905	0.996905
milin vs zangga	ko03008	Ribosome	2/219	64/8136	0.517668	0.996905	0.996905
milin vs zangga	ko05418	Fluid shear	5/219	181/8136	0.540652	0.996905	0.996905
milin vs zangga	ko00330	Arginine a	2/219	67/8136	0.542419	0.996905	0.996905
milin vs zangga	ko05332	Graft-vers	2/219	67/8136	0.542419	0.996905	0.996905
milin vs zangga	ko04924	Renin secr	3/219	106/8136	0.547165	0.996905	0.996905
milin vs zangga	ko04145	Phagosom	6/219	222/8136	0.554702	0.996905	0.996905
milin vs zangga	ko04961	Endocrine	2/219	69/8136	0.558426	0.996905	0.996905
milin vs zangga	ko04350	TGF-beta s	3/219	110/8136	0.572437	0.996905	0.996905
milin vs zangga	ko04140	Autophagy	5/219	189/8136	0.579444	0.996905	0.996905
milin vs zangga	ko05016	Huntingto	9/219	343/8136	0.580124	0.996905	0.996905
milin vs zangga	ko04742	Taste trans	2/219	72/8136	0.581685	0.996905	0.996905
milin vs zangga	ko04514	Cell adhes	7/219	267/8136	0.582952	0.996905	0.996905
milin vs zangga	ko05010	Alzheimer	12/219	462/8136	0.593986	0.996905	0.996905
milin vs zangga	ko04970	Salivary se	3/219	114/8136	0.596823	0.996905	0.996905
milin vs zangga	ko04728	Dopamine	5/219	193/8136	0.598182	0.996905	0.996905

milin vs zangga	ko05100	Bacterial ir	3/219	115/8136	0.602777	0.996905	0.996905
milin vs zangga	ko00592	alpha-Linc	1/219	34/8136	0.605307	0.996905	0.996905
milin vs zangga	ko00062	Fatty acid	1/219	35/8136	0.615976	0.996905	0.996905
milin vs zangga	ko03030	DNA replic	1/219	35/8136	0.615976	0.996905	0.996905
milin vs zangga	ko03440	Homologo	1/219	35/8136	0.615976	0.996905	0.996905
milin vs zangga	ko04978	Mineral ab	2/219	77/8136	0.618429	0.996905	0.996905
milin vs zangga	ko05212	Pancreatic	3/219	118/8136	0.62029	0.996905	0.996905
milin vs zangga	ko04015	Rap1 signa	8/219	317/8136	0.625244	0.996905	0.996905
milin vs zangga	ko00591	Linoleic ac	1/219	36/8136	0.626357	0.996905	0.996905
milin vs zangga	ko04136	Autophagy	1/219	36/8136	0.626357	0.996905	0.996905
milin vs zangga	ko04137	Mitophagy	2/219	79/8136	0.63242	0.996905	0.996905
milin vs zangga	ko04726	Serotoner	4/219	161/8136	0.63447	0.996905	0.996905
milin vs zangga	ko04020	Calcium si	10/219	398/8136	0.635134	0.996905	0.996905
milin vs zangga	ko04270	Vascular s	5/219	203/8136	0.642923	0.996905	0.996905
milin vs zangga	ko04361	Axon rege	3/219	124/8136	0.653727	0.996905	0.996905
milin vs zangga	ko00970	Aminoacyl	1/219	39/8136	0.655855	0.996905	0.996905
milin vs zangga	ko04341	Hedgehog	1/219	39/8136	0.655855	0.996905	0.996905
milin vs zangga	ko05340	Primary im	1/219	39/8136	0.655855	0.996905	0.996905
milin vs zangga	ko05202	Transcripti	6/219	247/8136	0.658898	0.996905	0.996905
milin vs zangga	ko04658	Th1 and Tl	3/219	125/8136	0.659091	0.996905	0.996905
milin vs zangga	ko05145	Toxoplasm	4/219	167/8136	0.662948	0.996905	0.996905
milin vs zangga	ko05215	Prostate ca	3/219	126/8136	0.664396	0.996905	0.996905
milin vs zangga	ko04710	Circadian r	1/219	41/8136	0.674221	0.996905	0.996905
milin vs zangga	ko05164	Influenza A	5/219	211/8136	0.676418	0.996905	0.996905
milin vs zangga	ko05163	Human cy	7/219	292/8136	0.676713	0.996905	0.996905
milin vs zangga	ko03015	mRNA sur	2/219	86/8136	0.678246	0.996905	0.996905
milin vs zangga	ko05330	Allograft r	2/219	86/8136	0.678246	0.996905	0.996905
milin vs zangga	ko00260	Glycine, se	1/219	42/8136	0.683035	0.996905	0.996905
milin vs zangga	ko04910	Insulin sigr	4/219	173/8136	0.689893	0.996905	0.996905
milin vs zangga	ko05320	Autoimmu	2/219	88/8136	0.690458	0.996905	0.996905
milin vs zangga	ko05208	NA	6/219	256/8136	0.692504	0.996905	0.996905
milin vs zangga	ko04215	Apoptosis	1/219	44/8136	0.699956	0.996905	0.996905
milin vs zangga	ko04213	Longevity	2/219	91/8136	0.708061	0.996905	0.996905
milin vs zangga	ko05217	Basal cell c	2/219	92/8136	0.713741	0.996905	0.996905
milin vs zangga	ko05414	Dilated ca	4/219	179/8136	0.715289	0.996905	0.996905
milin vs zangga	ko04925	Aldosteror	3/219	137/8136	0.718813	0.996905	0.996905
milin vs zangga	ko04740	Olfactory t	2/219	93/8136	0.719328	0.996905	0.996905
milin vs zangga	ko04611	Platelet ac	4/219	180/8136	0.719371	0.996905	0.996905
milin vs zangga	ko04919	Thyroid ho	4/219	180/8136	0.719371	0.996905	0.996905
milin vs zangga	ko04917	Prolactin s	2/219	94/8136	0.724824	0.996905	0.996905
milin vs zangga	ko05161	Hepatitis B	4/219	182/8136	0.727406	0.996905	0.996905
milin vs zangga	ko05230	Central ca	2/219	95/8136	0.730229	0.996905	0.996905
milin vs zangga	ko04024	cAMP sign	8/219	351/8136	0.735086	0.996905	0.996905
milin vs zangga	ko04927	Cortisol sy	2/219	96/8136	0.735545	0.996905	0.996905
milin vs zangga	ko00760	Nicotinate	1/219	49/8136	0.738428	0.996905	0.996905
milin vs zangga	ko04915	Estrogen s	4/219	186/8136	0.742962	0.996905	0.996905
milin vs zangga	ko00280	Valine, leu	1/219	51/8136	0.752404	0.996905	0.996905
milin vs zangga	ko00565	Ether lipid	1/219	51/8136	0.752404	0.996905	0.996905
milin vs zangga	ko04624	Toll and In	1/219	51/8136	0.752404	0.996905	0.996905
milin vs zangga	ko04724	Glutamate	4/219	189/8136	0.754183	0.996905	0.996905
milin vs zangga	ko04810	Regulation	7/219	317/8136	0.756242	0.996905	0.996905
milin vs zangga	ko04261	Adrenergic	5/219	236/8136	0.767223	0.996905	0.996905
milin vs zangga	ko04260	Cardiac m	3/219	149/8136	0.770134	0.996905	0.996905
milin vs zangga	ko04931	Insulin resi	3/219	149/8136	0.770134	0.996905	0.996905
milin vs zangga	ko04013	MAPK sign	2/219	103/8136	0.770311	0.996905	0.996905
milin vs zangga	ko02010	ABC transp	1/219	54/8136	0.771986	0.996905	0.996905
milin vs zangga	ko00564	Glyceroph	2/219	104/8136	0.77494	0.996905	0.996905
milin vs zangga	ko04371	Apelin sigr	4/219	196/8136	0.778903	0.996905	0.996905

milin vs zangga	ko04390	Hippo sigr	4/219	196/8136	0.778903	0.996905	0.996905
milin vs zangga	ko05162	Measles	3/219	152/8136	0.781708	0.996905	0.996905
milin vs zangga	ko05166	Human T-	6/219	284/8136	0.782607	0.996905	0.996905
milin vs zangga	ko04962	Vasopressi	1/219	56/8136	0.784176	0.996905	0.996905
milin vs zangga	ko04662	B cell rece	2/219	107/8136	0.788344	0.996905	0.996905
milin vs zangga	ko05220	Chronic m	2/219	109/8136	0.796887	0.996905	0.996905
milin vs zangga	ko04150	mTOR sigr	4/219	202/8136	0.798502	0.996905	0.996905
milin vs zangga	ko04926	Relaxin sig	3/219	157/8136	0.799932	0.996905	0.996905
milin vs zangga	ko05169	Epstein-Ba	5/219	247/8136	0.800548	0.996905	0.996905
milin vs zangga	ko04932	Non-alcoh	4/219	203/8136	0.801629	0.996905	0.996905
milin vs zangga	ko05206	MicroRNA	4/219	204/8136	0.804717	0.996905	0.996905
milin vs zangga	ko05170	Human im	5/219	249/8136	0.806188	0.996905	0.996905
milin vs zangga	ko04979	Cholestero	1/219	60/8136	0.806645	0.996905	0.996905
milin vs zangga	ko04310	Wnt signal	4/219	209/8136	0.81958	0.996905	0.996905
milin vs zangga	ko04650	Natural kil	2/219	116/8136	0.824437	0.996905	0.996905
milin vs zangga	ko01212	Fatty acid	1/219	64/8136	0.826784	0.996905	0.996905
milin vs zangga	ko04010	MAPK sigr	9/219	431/8136	0.827963	0.996905	0.996905
milin vs zangga	ko05012	Parkinson	6/219	303/8136	0.831454	0.996905	0.996905
milin vs zangga	ko05150	Staphyloc	2/219	118/8136	0.831671	0.996905	0.996905
milin vs zangga	ko04664	Fc epsilon	2/219	119/8136	0.835187	0.996905	0.996905
milin vs zangga	ko05167	Kaposi sar	4/219	215/8136	0.83618	0.996905	0.996905
milin vs zangga	ko01200	Carbon me	2/219	121/8136	0.84202	0.996905	0.996905
milin vs zangga	ko04211	Longevity	2/219	122/8136	0.84534	0.996905	0.996905
milin vs zangga	ko04935	Growth ho	3/219	174/8136	0.852638	0.996905	0.996905
milin vs zangga	ko04330	Notch sigr	1/219	70/8136	0.853144	0.996905	0.996905
milin vs zangga	ko05030	Cocaine ad	1/219	70/8136	0.853144	0.996905	0.996905
milin vs zangga	ko05221	Acute mye	1/219	73/8136	0.864785	0.996905	0.996905
milin vs zangga	ko04660	T cell rece	2/219	129/8136	0.866865	0.996905	0.996905
milin vs zangga	ko04727	GABAergic	2/219	129/8136	0.866865	0.996905	0.996905
milin vs zangga	ko04670	Leukocyte	3/219	180/8136	0.868135	0.996905	0.996905
milin vs zangga	ko04933	AGE-RAGE	2/219	130/8136	0.869706	0.996905	0.996905
milin vs zangga	ko04115	p53 signal	1/219	75/8136	0.872031	0.996905	0.996905
milin vs zangga	ko05134	Legionello	1/219	75/8136	0.872031	0.996905	0.996905
milin vs zangga	ko05415	NA	4/219	230/8136	0.87219	0.996905	0.996905
milin vs zangga	ko00190	Oxidative	2/219	131/8136	0.872492	0.996905	0.996905
milin vs zangga	ko05210	Colorectal	2/219	134/8136	0.880525	0.996905	0.996905
milin vs zangga	ko05165	Human pa	8/219	417/8136	0.880602	0.996905	0.996905
milin vs zangga	ko00600	Sphingolip	1/219	79/8136	0.885383	0.996905	0.996905
milin vs zangga	ko00590	Arachidon	1/219	80/8136	0.888499	0.996905	0.996905
milin vs zangga	ko01230	Biosynthes	1/219	80/8136	0.888499	0.996905	0.996905
milin vs zangga	ko01524	Platinum d	1/219	81/8136	0.89153	0.996905	0.996905
milin vs zangga	ko04918	Thyroid ho	1/219	82/8136	0.894479	0.996905	0.996905
milin vs zangga	ko00562	Inositol ph	1/219	84/8136	0.90014	0.996905	0.996905
milin vs zangga	ko05152	Tuberculos	4/219	245/8136	0.901197	0.996905	0.996905
milin vs zangga	ko04714	Thermoge	4/219	246/8136	0.902908	0.996905	0.996905
milin vs zangga	ko05168	Herpes sin	4/219	246/8136	0.902908	0.996905	0.996905
milin vs zangga	ko04066	HIF-1 sign	2/219	145/8136	0.906153	0.996905	0.996905
milin vs zangga	ko04540	Gap juncti	2/219	145/8136	0.906153	0.996905	0.996905
milin vs zangga	ko03320	PPAR sign	1/219	87/8136	0.908069	0.996905	0.996905
milin vs zangga	ko04916	Melanoge	2/219	148/8136	0.912198	0.996905	0.996905
milin vs zangga	ko04720	Long-term	1/219	90/8136	0.915372	0.996905	0.996905
milin vs zangga	ko03018	RNA degra	1/219	91/8136	0.917675	0.996905	0.996905
milin vs zangga	ko04152	AMPK sigr	2/219	152/8136	0.919693	0.996905	0.996905
milin vs zangga	ko04672	Intestinal i	1/219	92/8136	0.919916	0.996905	0.996905
milin vs zangga	ko05014	Amyotrop	7/219	406/8136	0.926392	0.996905	0.996905
milin vs zangga	ko05031	Amphetan	1/219	97/8136	0.930243	0.996905	0.996905
milin vs zangga	ko04512	ECM-rece	1/219	98/8136	0.932143	0.996905	0.996905
milin vs zangga	ko04722	Neurotrop	2/219	160/8136	0.93292	0.996905	0.996905

milin vs zangga	ko04934	Cushing sy	3/219	215/8136	0.932984	0.996905	0.996905
milin vs zangga	ko04370	VEGF signa	1/219	99/8136	0.933992	0.996905	0.996905
milin vs zangga	ko04928	Parathyroi	2/219	161/8136	0.934421	0.996905	0.996905
milin vs zangga	ko03013	RNA trans	1/219	101/8136	0.93754	0.996905	0.996905
milin vs zangga	ko05211	Renal cell	1/219	104/8136	0.94251	0.996905	0.996905
milin vs zangga	ko04612	Antigen pr	1/219	106/8136	0.945603	0.996905	0.996905
milin vs zangga	ko03010	Ribosome	1/219	107/8136	0.947086	0.996905	0.996905
milin vs zangga	ko04360	Axon guid	4/219	281/8136	0.94847	0.996905	0.996905
milin vs zangga	ko05020	Prion disea	5/219	335/8136	0.951245	0.996905	0.996905
milin vs zangga	ko05222	Small cell	1/219	110/8136	0.951299	0.996905	0.996905
milin vs zangga	ko04070	Phosphatid	1/219	111/8136	0.952628	0.996905	0.996905
milin vs zangga	ko05205	Proteoglyc	4/219	291/8136	0.957314	0.996905	0.996905
milin vs zangga	ko04012	ErbB signa	1/219	117/8136	0.959877	0.996905	0.996905
milin vs zangga	ko04218	Cellular se	2/219	183/8136	0.960417	0.996905	0.996905
milin vs zangga	ko04640	Hematopo	1/219	120/8136	0.963075	0.996905	0.996905
milin vs zangga	ko04071	Sphingolip	2/219	193/8136	0.968661	0.996905	0.996905
milin vs zangga	ko04721	Synaptic v	1/219	126/8136	0.968731	0.996905	0.996905
milin vs zangga	ko04014	Ras signali	4/219	309/8136	0.969806	0.996905	0.996905
milin vs zangga	ko04550	Signaling	2/219	195/8136	0.970099	0.996905	0.996905
milin vs zangga	ko01522	Endocrine	1/219	130/8136	0.972014	0.996905	0.996905
milin vs zangga	ko04912	GnRH sign	1/219	132/8136	0.973524	0.996905	0.996905
milin vs zangga	ko05226	Gastric car	2/219	206/8136	0.976943	0.996905	0.996905
milin vs zangga	ko05323	Rheumato	1/219	138/8136	0.977585	0.996905	0.996905
milin vs zangga	ko04922	Glucagon	1/219	143/8136	0.980491	0.996905	0.996905
milin vs zangga	ko05225	Hepatocel	2/219	214/8136	0.980945	0.996905	0.996905
milin vs zangga	ko05203	Viral carcir	2/219	230/8136	0.987033	0.996905	0.996905
milin vs zangga	ko00230	Purine met	1/219	158/8136	0.987144	0.996905	0.996905
milin vs zangga	ko05322	Systemic lu	1/219	174/8136	0.991768	0.996905	0.996905
milin vs zangga	ko04151	PI3K-Akt s	5/219	437/8136	0.992864	0.996905	0.996905
milin vs zangga	ko04510	Focal adhe	2/219	276/8136	0.995814	0.996905	0.996905
milin vs zangga	ko04613	NA	1/219	202/8136	0.996235	0.996905	0.996905
milin vs zangga	ko05034	Alcoholism	1/219	205/8136	0.996538	0.996905	0.996905
milin vs zangga	ko05224	Breast can	1/219	209/8136	0.996905	0.996905	0.996905
linzhi vs zangga	ko00531	Glycosami	4/193	22/8136	0.001606	0.405997	0.405997
linzhi vs zangga	ko04613	NA	12/193	202/8136	0.003053	0.405997	0.405997
linzhi vs zangga	ko00520	Amino sug	5/193	48/8136	0.005341	0.420385	0.420385
linzhi vs zangga	ko01250	NA	4/193	34/8136	0.00816	0.420385	0.420385
linzhi vs zangga	ko05322	Systemic lu	10/193	174/8136	0.008336	0.420385	0.420385
linzhi vs zangga	ko05034	Alcoholism	11/193	205/8136	0.009482	0.420385	0.420385
linzhi vs zangga	ko04911	Insulin sec	8/193	137/8136	0.016069	0.565659	0.565659
linzhi vs zangga	ko05144	Malaria	4/193	42/8136	0.017012	0.565659	0.565659
linzhi vs zangga	ko04142	Lysosome	8/193	150/8136	0.026128	0.772222	0.772222
linzhi vs zangga	ko04217	Necroptos	10/193	219/8136	0.035445	0.942849	0.942849
linzhi vs zangga	ko04966	Collecting	3/193	32/8136	0.039378	0.952229	0.952229
linzhi vs zangga	ko04962	Vasopress	4/193	56/8136	0.043316	0.960181	0.960181
linzhi vs zangga	ko00510	N-Glycan	4/193	58/8136	0.048268	0.987641	0.987641
linzhi vs zangga	ko04979	Cholesterc	4/193	60/8136	0.05352	0.998524	0.998524
linzhi vs zangga	ko00970	Aminoacyl	3/193	39/8136	0.064412	0.998524	0.998524
linzhi vs zangga	ko03040	Spliceosor	7/193	152/8136	0.0695	0.998524	0.998524
linzhi vs zangga	ko00330	Arginine a	4/193	67/8136	0.074229	0.998524	0.998524
linzhi vs zangga	ko03460	Fanconi ar	3/193	46/8136	0.095087	0.998524	0.998524
linzhi vs zangga	ko04621	NOD-like	7/193	165/8136	0.09727	0.998524	0.998524
linzhi vs zangga	ko05014	Amyotrop	14/193	406/8136	0.101938	0.998524	0.998524
linzhi vs zangga	ko00500	Starch and	2/193	30/8136	0.158422	0.998524	0.998524
linzhi vs zangga	ko00140	Steroid ho	3/193	59/8136	0.164081	0.998524	0.998524
linzhi vs zangga	ko00220	Arginine b	2/193	31/8136	0.166844	0.998524	0.998524
linzhi vs zangga	ko04926	Relaxin sig	6/193	157/8136	0.169335	0.998524	0.998524
linzhi vs zangga	ko00030	Pentose pl	2/193	32/8136	0.175342	0.998524	0.998524

linzhi vs zangga	ko00410	beta-Alan	2/193	32/8136	0.175342	0.998524	0.998524
linzhi vs zangga	ko05204	Chemical c	3/193	62/8136	0.181712	0.998524	0.998524
linzhi vs zangga	ko01040	Biosynthes	2/193	34/8136	0.192532	0.998524	0.998524
linzhi vs zangga	ko05133	Pertussis	4/193	96/8136	0.193373	0.998524	0.998524
linzhi vs zangga	ko01212	Fatty acid	3/193	64/8136	0.193738	0.998524	0.998524
linzhi vs zangga	ko00190	Oxidative	5/193	131/8136	0.199931	0.998524	0.998524
linzhi vs zangga	ko00062	Fatty acid	2/193	35/8136	0.201208	0.998524	0.998524
linzhi vs zangga	ko04744	Phototrans	2/193	35/8136	0.201208	0.998524	0.998524
linzhi vs zangga	ko04976	Bile secret	5/193	133/8136	0.208335	0.998524	0.998524
linzhi vs zangga	ko05207	NA	9/193	276/8136	0.208845	0.998524	0.998524
linzhi vs zangga	ko00591	Linoleic ac	2/193	36/8136	0.209929	0.998524	0.998524
linzhi vs zangga	ko00040	Pentose ar	2/193	37/8136	0.218688	0.998524	0.998524
linzhi vs zangga	ko03450	Non-hom	1/193	11/8136	0.232217	0.998524	0.998524
linzhi vs zangga	ko00310	Lysine deg	3/193	72/8136	0.243556	0.998524	0.998524
linzhi vs zangga	ko00830	Retinol me	3/193	73/8136	0.249934	0.998524	0.998524
linzhi vs zangga	ko00430	Taurine an	1/193	12/8136	0.250455	0.998524	0.998524
linzhi vs zangga	ko00380	Tryptopha	2/193	41/8136	0.25397	0.998524	0.998524
linzhi vs zangga	ko04066	HIF-1 sign	5/193	145/8136	0.260934	0.998524	0.998524
linzhi vs zangga	ko05208	NA	8/193	256/8136	0.262367	0.998524	0.998524
linzhi vs zangga	ko00450	Selenocom	1/193	13/8136	0.268262	0.998524	0.998524
linzhi vs zangga	ko00071	Fatty acid	2/193	43/8136	0.271678	0.998524	0.998524
linzhi vs zangga	ko05020	Prion dise	10/193	335/8136	0.272361	0.998524	0.998524
linzhi vs zangga	ko04260	Cardiac m	5/193	149/8136	0.27914	0.998524	0.998524
linzhi vs zangga	ko00250	Alanine, as	2/193	44/8136	0.28053	0.998524	0.998524
linzhi vs zangga	ko04623	Cytosolic D	2/193	45/8136	0.289374	0.998524	0.998524
linzhi vs zangga	ko01523	Antifolate	2/193	46/8136	0.298205	0.998524	0.998524
linzhi vs zangga	ko05010	Alzheimer	13/193	462/8136	0.301994	0.998524	0.998524
linzhi vs zangga	ko05415	NA	7/193	230/8136	0.304722	0.998524	0.998524
linzhi vs zangga	ko00513	Various typ	2/193	48/8136	0.315812	0.998524	0.998524
linzhi vs zangga	ko00230	Purine me	5/193	158/8136	0.320933	0.998524	0.998524
linzhi vs zangga	ko04928	Parathyroi	5/193	161/8136	0.335044	0.998524	0.998524
linzhi vs zangga	ko00061	Fatty acid	1/193	17/8136	0.335378	0.998524	0.998524
linzhi vs zangga	ko04624	Toll and In	2/193	51/8136	0.342028	0.998524	0.998524
linzhi vs zangga	ko04020	Calcium si	11/193	398/8136	0.344724	0.998524	0.998524
linzhi vs zangga	ko00670	One carbo	1/193	18/8136	0.351177	0.998524	0.998524
linzhi vs zangga	ko00910	Nitrogen r	1/193	18/8136	0.351177	0.998524	0.998524
linzhi vs zangga	ko00983	Drug meta	3/193	89/8136	0.353881	0.998524	0.998524
linzhi vs zangga	ko05146	Amoebiasi	4/193	127/8136	0.355604	0.998524	0.998524
linzhi vs zangga	ko05145	Toxoplasm	5/193	167/8136	0.363412	0.998524	0.998524
linzhi vs zangga	ko05226	Gastric car	6/193	206/8136	0.363592	0.998524	0.998524
linzhi vs zangga	ko05412	Arrhythmo	4/193	129/8136	0.366445	0.998524	0.998524
linzhi vs zangga	ko04714	Thermoge	7/193	246/8136	0.366492	0.998524	0.998524
linzhi vs zangga	ko02010	ABC trans	2/193	54/8136	0.367923	0.998524	0.998524
linzhi vs zangga	ko04657	IL-17 sign	3/193	92/8136	0.373386	0.998524	0.998524
linzhi vs zangga	ko00340	Histidine r	1/193	20/8136	0.381662	0.998524	0.998524
linzhi vs zangga	ko04146	Peroxisom	3/193	96/8136	0.399212	0.998524	0.998524
linzhi vs zangga	ko05165	Human pa	11/193	417/8136	0.402805	0.998524	0.998524
linzhi vs zangga	ko00770	Pantothen	1/193	22/8136	0.410723	0.998524	0.998524
linzhi vs zangga	ko01210	2-Oxocarb	1/193	22/8136	0.410723	0.998524	0.998524
linzhi vs zangga	ko05016	Huntingto	9/193	343/8136	0.427056	0.998524	0.998524
linzhi vs zangga	ko05012	Parkinson	8/193	303/8136	0.430014	0.998524	0.998524
linzhi vs zangga	ko04622	RIG-I-like	2/193	62/8136	0.434829	0.998524	0.998524
linzhi vs zangga	ko04013	MAPK sign	3/193	103/8136	0.443651	0.998524	0.998524
linzhi vs zangga	ko04010	MAPK sign	11/193	431/8136	0.445971	0.998524	0.998524
linzhi vs zangga	ko05211	Renal cell	3/193	104/8136	0.4499	0.998524	0.998524
linzhi vs zangga	ko00511	Other glyc	1/193	25/8136	0.451785	0.998524	0.998524
linzhi vs zangga	ko00020	Citrate cyc	1/193	26/8136	0.46483	0.998524	0.998524
linzhi vs zangga	ko00532	Glycosami	1/193	26/8136	0.46483	0.998524	0.998524

linzhi vs zangga	ko03020	RNA polyr	1/193	26/8136	0.46483	0.998524	0.998524
linzhi vs zangga	ko04662	B cell rece	3/193	107/8136	0.468475	0.998524	0.998524
linzhi vs zangga	ko00650	Butanoate	1/193	27/8136	0.477566	0.998524	0.998524
linzhi vs zangga	ko05222	Small cell l	3/193	110/8136	0.486769	0.998524	0.998524
linzhi vs zangga	ko00630	Glyoxylate	1/193	28/8136	0.49	0.998524	0.998524
linzhi vs zangga	ko00900	Terpenoid	1/193	28/8136	0.49	0.998524	0.998524
linzhi vs zangga	ko04330	Notch sign	2/193	70/8136	0.497777	0.998524	0.998524
linzhi vs zangga	ko05030	Cocaine ac	2/193	70/8136	0.497777	0.998524	0.998524
linzhi vs zangga	ko05410	Hypertrop	4/193	154/8136	0.498958	0.998524	0.998524
linzhi vs zangga	ko04064	NF-kappa	3/193	114/8136	0.510682	0.998524	0.998524
linzhi vs zangga	ko05218	Melanoma	3/193	114/8136	0.510682	0.998524	0.998524
linzhi vs zangga	ko04141	Protein pro	5/193	199/8136	0.512406	0.998524	0.998524
linzhi vs zangga	ko00053	Ascorbate	1/193	31/8136	0.525564	0.998524	0.998524
linzhi vs zangga	ko00010	Glycolysis	2/193	75/8136	0.534798	0.998524	0.998524
linzhi vs zangga	ko05134	Legionello	2/193	75/8136	0.534798	0.998524	0.998524
linzhi vs zangga	ko00240	Pyrimidine	2/193	76/8136	0.541976	0.998524	0.998524
linzhi vs zangga	ko01200	Carbon me	3/193	121/8136	0.551081	0.998524	0.998524
linzhi vs zangga	ko04022	cGMP-PKG	6/193	252/8136	0.555109	0.998524	0.998524
linzhi vs zangga	ko05224	Breast can	5/193	209/8136	0.556295	0.998524	0.998524
linzhi vs zangga	ko05033	Nicotine a	2/193	79/8136	0.563049	0.998524	0.998524
linzhi vs zangga	ko03410	Base excisi	1/193	35/8136	0.569173	0.998524	0.998524
linzhi vs zangga	ko04392	Hippo sign	1/193	35/8136	0.569173	0.998524	0.998524
linzhi vs zangga	ko04721	Synaptic v	3/193	126/8136	0.578713	0.998524	0.998524
linzhi vs zangga	ko04130	SNARE inte	1/193	36/8136	0.579437	0.998524	0.998524
linzhi vs zangga	ko04136	Autophagy	1/193	36/8136	0.579437	0.998524	0.998524
linzhi vs zangga	ko05140	Leishmania	3/193	127/8136	0.584111	0.998524	0.998524
linzhi vs zangga	ko05142	Chagas dis	3/193	128/8136	0.589465	0.998524	0.998524
linzhi vs zangga	ko04660	T cell rece	3/193	129/8136	0.594775	0.998524	0.998524
linzhi vs zangga	ko04727	GABAergic	3/193	129/8136	0.594775	0.998524	0.998524
linzhi vs zangga	ko04668	TNF signal	3/193	131/8136	0.605262	0.998524	0.998524
linzhi vs zangga	ko04950	Maturity o	1/193	39/8136	0.608793	0.998524	0.998524
linzhi vs zangga	ko04391	Hippo sign	2/193	87/8136	0.615813	0.998524	0.998524
linzhi vs zangga	ko05414	Dilated ca	4/193	179/8136	0.618343	0.998524	0.998524
linzhi vs zangga	ko04611	Platelet ac	4/193	180/8136	0.622739	0.998524	0.998524
linzhi vs zangga	ko04710	Circadian r	1/193	41/8136	0.627221	0.998524	0.998524
linzhi vs zangga	ko04015	Rap1 signa	7/193	317/8136	0.631137	0.998524	0.998524
linzhi vs zangga	ko04720	Long-term	2/193	90/8136	0.634308	0.998524	0.998524
linzhi vs zangga	ko04923	Regulation	2/193	90/8136	0.634308	0.998524	0.998524
linzhi vs zangga	ko05132	Salmonella	7/193	318/8136	0.634445	0.998524	0.998524
linzhi vs zangga	ko04925	Aldosteron	3/193	137/8136	0.635638	0.998524	0.998524
linzhi vs zangga	ko04213	Longevity	2/193	91/8136	0.640317	0.998524	0.998524
linzhi vs zangga	ko05323	Rheumato	3/193	138/8136	0.64054	0.998524	0.998524
linzhi vs zangga	ko05203	Viral carcir	5/193	230/8136	0.641621	0.998524	0.998524
linzhi vs zangga	ko00051	Fructose a	1/193	44/8136	0.653257	0.998524	0.998524
linzhi vs zangga	ko04625	C-type lec	3/193	141/8136	0.654971	0.998524	0.998524
linzhi vs zangga	ko04964	Proximal t	1/193	45/8136	0.661527	0.998524	0.998524
linzhi vs zangga	ko04261	Adrenergic	5/193	236/8136	0.66406	0.998524	0.998524
linzhi vs zangga	ko00512	Mucin type	1/193	46/8136	0.669601	0.998524	0.998524
linzhi vs zangga	ko00860	Porphyrin	1/193	46/8136	0.669601	0.998524	0.998524
linzhi vs zangga	ko05166	Human T-	6/193	284/8136	0.671533	0.998524	0.998524
linzhi vs zangga	ko05031	Amphetam	2/193	97/8136	0.674756	0.998524	0.998524
linzhi vs zangga	ko05219	Bladder ca	1/193	47/8136	0.677483	0.998524	0.998524
linzhi vs zangga	ko04370	VEGF signa	2/193	99/8136	0.685629	0.998524	0.998524
linzhi vs zangga	ko04390	Hippo sign	4/193	196/8136	0.68867	0.998524	0.998524
linzhi vs zangga	ko04620	Toll-like re	2/193	100/8136	0.690953	0.998524	0.998524
linzhi vs zangga	ko04931	Insulin resi	3/193	149/8136	0.691416	0.998524	0.998524
linzhi vs zangga	ko05152	Tuberculos	5/193	245/8136	0.695987	0.998524	0.998524
linzhi vs zangga	ko03013	RNA trans	2/193	101/8136	0.696203	0.998524	0.998524

linzhi vs zangga	ko04920	Adipocyto	2/193	101/8136	0.696203	0.998524	0.998524
linzhi vs zangga	ko05202	Transcripti	5/193	247/8136	0.702794	0.998524	0.998524
linzhi vs zangga	ko05417	NA	5/193	248/8136	0.706158	0.998524	0.998524
linzhi vs zangga	ko00280	Valine, leu	1/193	51/8136	0.707184	0.998524	0.998524
linzhi vs zangga	ko03050	Proteasom	1/193	51/8136	0.707184	0.998524	0.998524
linzhi vs zangga	ko04975	Fat digesti	1/193	51/8136	0.707184	0.998524	0.998524
linzhi vs zangga	ko05216	Thyroid ca	1/193	51/8136	0.707184	0.998524	0.998524
linzhi vs zangga	ko04150	mTOR sigr	4/193	202/8136	0.711205	0.998524	0.998524
linzhi vs zangga	ko04932	Non-alcoh	4/193	203/8136	0.714843	0.998524	0.998524
linzhi vs zangga	ko04120	Ubiquitin r	3/193	156/8136	0.720884	0.998524	0.998524
linzhi vs zangga	ko00514	Other type	1/193	53/8136	0.720998	0.998524	0.998524
linzhi vs zangga	ko04612	Antigen pr	2/193	106/8136	0.721361	0.998524	0.998524
linzhi vs zangga	ko05231	Choline m	3/193	159/8136	0.73283	0.998524	0.998524
linzhi vs zangga	ko04530	Tight junct	5/193	259/8136	0.741425	0.998524	0.998524
linzhi vs zangga	ko05164	Influenza A	4/193	211/8136	0.742754	0.998524	0.998524
linzhi vs zangga	ko00982	Drug meta	1/193	57/8136	0.746711	0.998524	0.998524
linzhi vs zangga	ko04730	Long-term	2/193	114/8136	0.757964	0.998524	0.998524
linzhi vs zangga	ko04810	Regulation	6/193	317/8136	0.769418	0.998524	0.998524
linzhi vs zangga	ko00561	Glycerolipi	1/193	61/8136	0.770065	0.998524	0.998524
linzhi vs zangga	ko00980	Metabolisr	1/193	61/8136	0.770065	0.998524	0.998524
linzhi vs zangga	ko04664	Fc epsilon	2/193	119/8136	0.778681	0.998524	0.998524
linzhi vs zangga	ko03008	Ribosome	1/193	64/8136	0.786163	0.998524	0.998524
linzhi vs zangga	ko01521	EGFR tyros	2/193	124/8136	0.797838	0.998524	0.998524
linzhi vs zangga	ko04725	Cholinergi	3/193	178/8136	0.799376	0.998524	0.998524
linzhi vs zangga	ko04658	Th1 and Th	2/193	125/8136	0.801489	0.998524	0.998524
linzhi vs zangga	ko04919	Thyroid ho	3/193	180/8136	0.805508	0.998524	0.998524
linzhi vs zangga	ko05017	Spinocerel	3/193	181/8136	0.808515	0.998524	0.998524
linzhi vs zangga	ko05418	Fluid shea	3/193	181/8136	0.808515	0.998524	0.998524
linzhi vs zangga	ko04218	Cellular se	3/193	183/8136	0.814412	0.998524	0.998524
linzhi vs zangga	ko04933	AGE-RAGE	2/193	130/8136	0.818886	0.998524	0.998524
linzhi vs zangga	ko04151	PI3K-Akt s	8/193	437/8136	0.821624	0.998524	0.998524
linzhi vs zangga	ko04380	Osteoclast	2/193	131/8136	0.822198	0.998524	0.998524
linzhi vs zangga	ko04915	Estrogen s	3/193	186/8136	0.82297	0.998524	0.998524
linzhi vs zangga	ko04742	Taste trans	1/193	72/8136	0.823816	0.998524	0.998524
linzhi vs zangga	ko05163	Human cy	5/193	292/8136	0.828496	0.998524	0.998524
linzhi vs zangga	ko04115	p53 signal	1/193	75/8136	0.836167	0.998524	0.998524
linzhi vs zangga	ko04728	Dopamine	3/193	193/8136	0.841646	0.998524	0.998524
linzhi vs zangga	ko05169	Epstein-Ba	4/193	247/8136	0.843222	0.998524	0.998524
linzhi vs zangga	ko04936	NA	2/193	138/8136	0.843904	0.998524	0.998524
linzhi vs zangga	ko04550	Signaling p	3/193	195/8136	0.846661	0.998524	0.998524
linzhi vs zangga	ko00600	Sphingolip	1/193	79/8136	0.851306	0.998524	0.998524
linzhi vs zangga	ko04137	Mitophagy	1/193	79/8136	0.851306	0.998524	0.998524
linzhi vs zangga	ko04972	Pancreatic	2/193	141/8136	0.852452	0.998524	0.998524
linzhi vs zangga	ko00590	Arachidon	1/193	80/8136	0.854868	0.998524	0.998524
linzhi vs zangga	ko01230	Biosynthes	1/193	80/8136	0.854868	0.998524	0.998524
linzhi vs zangga	ko04940	Type I diak	1/193	80/8136	0.854868	0.998524	0.998524
linzhi vs zangga	ko04922	Glucagon	2/193	143/8136	0.857913	0.998524	0.998524
linzhi vs zangga	ko04918	Thyroid ho	1/193	82/8136	0.861739	0.998524	0.998524
linzhi vs zangga	ko04014	Ras signali	5/193	309/8136	0.863185	0.998524	0.998524
linzhi vs zangga	ko04540	Gap juncti	2/193	145/8136	0.863189	0.998524	0.998524
linzhi vs zangga	ko04270	Vascular s	3/193	203/8136	0.865373	0.998524	0.998524
linzhi vs zangga	ko04061	Viral prote	1/193	84/8136	0.868286	0.998524	0.998524
linzhi vs zangga	ko04210	Apoptosis	3/193	205/8136	0.869727	0.998524	0.998524
linzhi vs zangga	ko04659	Th17 cell c	2/193	148/8136	0.87077	0.998524	0.998524
linzhi vs zangga	ko04913	Ovarian st	1/193	85/8136	0.871443	0.998524	0.998524
linzhi vs zangga	ko04723	Retrograde	3/193	206/8136	0.871858	0.998524	0.998524
linzhi vs zangga	ko03320	PPAR sign	1/193	87/8136	0.877533	0.998524	0.998524
linzhi vs zangga	ko04610	Compleme	1/193	87/8136	0.877533	0.998524	0.998524

linzhi vs zangga	ko04310	Wnt signal	3/193	209/8136	0.878067	0.998524	0.998524
linzhi vs zangga	ko05162	Measles	2/193	152/8136	0.880279	0.998524	0.998524
linzhi vs zangga	ko04934	Cushing sy	3/193	215/8136	0.889694	0.998524	0.998524
linzhi vs zangga	ko05167	Kaposi sar	3/193	215/8136	0.889694	0.998524	0.998524
linzhi vs zangga	ko04672	Intestinal i	1/193	92/8136	0.891532	0.998524	0.998524
linzhi vs zangga	ko05217	Basal cell c	1/193	92/8136	0.891532	0.998524	0.998524
linzhi vs zangga	ko04212	Longevity	1/193	93/8136	0.894134	0.998524	0.998524
linzhi vs zangga	ko04740	Olfactory t	1/193	93/8136	0.894134	0.998524	0.998524
linzhi vs zangga	ko05160	Hepatitis C	3/193	218/8136	0.895129	0.998524	0.998524
linzhi vs zangga	ko04917	Prolactin s	1/193	94/8136	0.896675	0.998524	0.998524
linzhi vs zangga	ko04722	Neurotrop	2/193	160/8136	0.897396	0.998524	0.998524
linzhi vs zangga	ko05230	Central car	1/193	95/8136	0.899154	0.998524	0.998524
linzhi vs zangga	ko04927	Cortisol sy	1/193	96/8136	0.901575	0.998524	0.998524
linzhi vs zangga	ko04145	Phagosom	3/193	222/8136	0.902004	0.998524	0.998524
linzhi vs zangga	ko04512	ECM-recep	1/193	98/8136	0.906244	0.998524	0.998524
linzhi vs zangga	ko04929	GnRH secr	1/193	98/8136	0.906244	0.998524	0.998524
linzhi vs zangga	ko01240	NA	2/193	166/8136	0.908715	0.998524	0.998524
linzhi vs zangga	ko05235	PD-L1 exp	1/193	101/8136	0.912837	0.998524	0.998524
linzhi vs zangga	ko04060	Cytokine-c	3/193	232/8136	0.917454	0.998524	0.998524
linzhi vs zangga	ko05205	Proteoglyc	4/193	291/8136	0.919403	0.998524	0.998524
linzhi vs zangga	ko04935	Growth ho	2/193	174/8136	0.922003	0.998524	0.998524
linzhi vs zangga	ko04924	Renin secr	1/193	106/8136	0.922817	0.998524	0.998524
linzhi vs zangga	ko03010	Ribosome	1/193	107/8136	0.924672	0.998524	0.998524
linzhi vs zangga	ko04971	Gastric aci	1/193	107/8136	0.924672	0.998524	0.998524
linzhi vs zangga	ko04350	TGF-beta s	1/193	110/8136	0.929975	0.998524	0.998524
linzhi vs zangga	ko04670	Leukocyte	2/193	180/8136	0.930756	0.998524	0.998524
linzhi vs zangga	ko04974	Protein dig	1/193	112/8136	0.933303	0.998524	0.998524
linzhi vs zangga	ko04713	Circadian e	2/193	182/8136	0.933462	0.998524	0.998524
linzhi vs zangga	ko05161	Hepatitis B	2/193	182/8136	0.933462	0.998524	0.998524
linzhi vs zangga	ko05168	Herpes sim	3/193	246/8136	0.935384	0.998524	0.998524
linzhi vs zangga	ko05171	NA	3/193	246/8136	0.935384	0.998524	0.998524
linzhi vs zangga	ko04970	Salivary se	1/193	114/8136	0.936473	0.998524	0.998524
linzhi vs zangga	ko05100	Bacterial in	1/193	115/8136	0.938001	0.998524	0.998524
linzhi vs zangga	ko04914	Progestero	1/193	116/8136	0.939493	0.998524	0.998524
linzhi vs zangga	ko04140	Autophagy	2/193	189/8136	0.942169	0.998524	0.998524
linzhi vs zangga	ko05212	Pancreatic	1/193	118/8136	0.94237	0.998524	0.998524
linzhi vs zangga	ko04640	Hematopo	1/193	120/8136	0.945112	0.998524	0.998524
linzhi vs zangga	ko04211	Longevity	1/193	122/8136	0.947723	0.998524	0.998524
linzhi vs zangga	ko04371	Apelin sign	2/193	196/8136	0.949791	0.998524	0.998524
linzhi vs zangga	ko04361	Axon rege	1/193	124/8136	0.950211	0.998524	0.998524
linzhi vs zangga	ko05416	Viral myoc	1/193	128/8136	0.954838	0.998524	0.998524
linzhi vs zangga	ko04144	Endocytos	4/193	329/8136	0.956571	0.998524	0.998524
linzhi vs zangga	ko01522	Endocrine	1/193	130/8136	0.956989	0.998524	0.998524
linzhi vs zangga	ko05032	Morphine	1/193	130/8136	0.956989	0.998524	0.998524
linzhi vs zangga	ko04912	GnRH sign	1/193	132/8136	0.959038	0.998524	0.998524
linzhi vs zangga	ko04114	Oocyte me	1/193	134/8136	0.96099	0.998524	0.998524
linzhi vs zangga	ko04110	Cell cycle	1/193	135/8136	0.961931	0.998524	0.998524
linzhi vs zangga	ko04520	Adherens	1/193	135/8136	0.961931	0.998524	0.998524
linzhi vs zangga	ko04510	Focal adhe	3/193	276/8136	0.962399	0.998524	0.998524
linzhi vs zangga	ko05135	Yersinia in	2/193	219/8136	0.968661	0.998524	0.998524
linzhi vs zangga	ko04024	cAMP sign	4/193	351/8136	0.970115	0.998524	0.998524
linzhi vs zangga	ko04750	Inflammat	1/193	146/8136	0.970903	0.998524	0.998524
linzhi vs zangga	ko04072	Phospholip	2/193	224/8136	0.971749	0.998524	0.998524
linzhi vs zangga	ko04916	Melanoge	1/193	148/8136	0.972292	0.998524	0.998524
linzhi vs zangga	ko04152	AMPK sign	1/193	152/8136	0.974874	0.998524	0.998524
linzhi vs zangga	ko04921	Oxytocin s	2/193	232/8136	0.976092	0.998524	0.998524
linzhi vs zangga	ko04726	Serotoner	1/193	161/8136	0.979843	0.998524	0.998524
linzhi vs zangga	ko04068	FoxO signa	1/193	168/8136	0.983021	0.998524	0.998524

linzhi vs zangga	ko05170	Human im	2/193	249/8136	0.983289	0.998524	0.998524
linzhi vs zangga	ko04910	Insulin sigr	1/193	173/8136	0.98498	0.998524	0.998524
linzhi vs zangga	ko04724	Glutamate	1/193	189/8136	0.989861	0.998524	0.998524
linzhi vs zangga	ko04071	Sphingolip	1/193	193/8136	0.990811	0.998524	0.998524
linzhi vs zangga	ko04360	Axon guid	2/193	281/8136	0.991582	0.998524	0.998524
linzhi vs zangga	ko05206	MicroRNA	1/193	204/8136	0.99299	0.998524	0.998524
linzhi vs zangga	ko05225	Hepatocel	1/193	214/8136	0.994522	0.998524	0.998524
linzhi vs zangga	ko04062	Chemokin	1/193	227/8136	0.996025	0.998524	0.998524
linzhi vs zangga	ko04514	Cell adhes	1/193	267/8136	0.998524	0.998524	0.998524

geneID	Count
Soc_11G0(	14
Soc_11G0(	7
Soc_13G0(	9
Soc_10G0(	16
Soc_11G0(	10
Soc_12G0(	5
Soc_11G0(	11
Soc_15G0(	10
Soc_11G0(	10
Soc_20G0(	7
Soc_15G0(	9
Soc_11G0(	5
Soc_11G0(	9
Soc_13G0(	4
Soc_13G0(	4
Soc_11G0(	12
Soc_11G0(	12
Soc_12G0(	3
Soc_1G001	9
Soc_11G0(	4
Soc_24G0(	2
Soc_15G0(	4
Soc_12G0(	3
Soc_12G0(	3
Soc_11G0(	9
Soc_12G0(	12
Soc_15G0(	2
Soc_13G0(	4
Soc_11G0(	11
Soc_11G0(	6
Soc_15G0(	4
Soc_15G0(	6
Soc_15G0(	9
Soc_15G0(	9
Soc_13G0(	6
Soc_13G0(	5
Soc_15G0(	5
Soc_11G0(	5
Soc_13G0(	7
Soc_11G0(	4
Soc_13G0(	10
Soc_11G0(	5
Soc_15G0(	8
Soc_19G0(	7
Soc_11G0(	11
Soc_11G0(	6
Soc_18G0(	5
Soc_13G0(	9
Soc_13G0(	12
Soc_10G0(	2
Soc_15G0(	10
Soc_20G0(	6
Soc_11G0(	6
Soc_6G000	2
Soc_12G0(	3
Soc_14G0(	5

Soc_13G0(	4
Soc_15G0(	6
Soc_13G0(	6
Soc_13G0(	8
Soc_11G0(	7
Soc_11G0(	16
Soc_11G0(	8
Soc_11G0(	5
Soc_20G0(	2
Soc_11G0(	6
Soc_12G0(	12
Soc_13G0(	9
Soc_11G0(	6
Soc_11G0(	9
Soc_13G0(	4
Soc_11G0(	11
Soc_14G0(	4
Soc_11G0(	8
Soc_13G0(	3
Soc_20G0(	6
Soc_10G0(	4
Soc_11G0(	8
Soc_1G000	1
Soc_5G000	4
Soc_15G0(	5
Soc_10G0(	14
Soc_15G0(	2
Soc_13G0(	5
Soc_11G0(	5
Soc_13G0(	13
Soc_12G0(	2
Soc_1G001	5
Soc_13G0(	8
Soc_15G0(	1
Soc_15G0(	2
Soc_22G0(	2
Soc_13G0(	5
Soc_10G0(	10
Soc_12G0(	3
Soc_13G0(	9
Soc_15G0(	7
Soc_18G0(	6
Soc_5G000	4
Soc_13G0(	4
Soc_22G0(	1
Soc_13G0(	2
Soc_18G0(	4
Soc_15G0(	6
Soc_13G0(	3
Soc_11G0(	3
Soc_18G0(	3
Soc_22G0(	1
Soc_13G0(	3
Soc_13G0(	2
Soc_18G0(	5
Soc_1G001	1
Soc_18G0(	5
Soc_13G0(	6

Soc_11G00	3
Soc_15G00	7
Soc_15G00	2
Soc_11G00	2
Soc_15G00	1
Soc_11G00	3
Soc_16G00	3
Soc_11G00	9
Soc_18G00	3
Soc_12G00	3
Soc_23G00	1
Soc_15G00	1
Soc_1G002	1
Soc_11G00	7
Soc_12G00	2
Soc_11G00	1
Soc_11G00	7
Soc_12G00	3
Soc_11G00	5
Soc_12G00	1
Soc_11G00	5
Soc_19G00	5
Soc_22G00	2
Soc_15G00	6
Soc_18G00	1
Soc_13G00	9
Soc_5G000	3
Soc_22G00	1
Soc_12G00	2
Soc_15G00	2
Soc_18G00	3
Soc_22G00	2
Soc_15G00	4
Soc_5G000	4
Soc_13G00	4
Soc_12G00	5
Soc_7G000	3
Soc_6G000	2
Soc_15G00	3
Soc_15G00	4
Soc_18G00	5
Soc_20G00	1
Soc_15G00	4
Soc_18G00	10
Soc_11G00	5
Soc_15G00	2
Soc_9G000	1
Soc_13G00	1
Soc_11G00	3
Soc_15G00	5
Soc_13G00	3
Soc_11G00	6
Soc_13G00	4
Soc_11G00	3
Soc_13G00	1
Soc_11G00	6
Soc_13G00	5
Soc_13G00	2

Soc_23G0(	1
Soc_12G0(	1
Soc_7G000	1
Soc_11G0(	1
Soc_11G0(	3
Soc_11G0(	1
Soc_20G0(	2
Soc_3G000	1
Soc_18G0(	1
Soc_15G0(	3
Soc_11G0(	10
Soc_22G0(	1
Soc_15G0(	4
Soc_14G0(	3
Soc_11G0(	4
Soc_6G000	1
Soc_6G000	1
Soc_11G0(	1
Soc_8G000	1
Soc_11G0(	10
Soc_15G0(	2
Soc_11G0(	5
Soc_11G0(	4
Soc_11G0(	3
Soc_15G0(	2
Soc_6G000	1
Soc_15G0(	2
Soc_13G0(	5
Soc_18G0(	1
Soc_6G000	2
Soc_15G0(	2
Soc_13G0(	1
Soc_15G0(	2
Soc_13G0(	2
Soc_1G002	1
Soc_1G000	1
Soc_14G0(	4
Soc_12G0(	2
Soc_15G0(	7
Soc_15G0(	1
Soc_5G000	1
Soc_20G0(	1
Soc_13G0(	3
Soc_13G0(	4
Soc_15G0(	1
Soc_15G0(	2
Soc_13G0(	2
Soc_14G0(	6
Soc_15G0(	2
Soc_19G0(	2
Soc_15G0(	2
Soc_15G0(	2
Soc_3G000	1
Soc_3G000	1
Soc_18G0(	5
Soc_15G0(	5
Soc_18G0(	2
Soc_10G0(	5

Soc_18G00	1
Soc_15G00	1
Soc_15G00	2
Soc_7G000	1
Soc_18G00	3
Soc_21G00	3
Soc_11G00	5
Soc_3G001	3
Soc_9G000	1
Soc_13G00	1
Soc_6G000	1
Soc_13G00	1
Soc_9G000	1
Soc_3G000	1
Soc_15G00	2
Soc_20G00	1
Soc_9G000	1
Soc_13G00	1
Soc_18G00	1
Soc_15G00	1
Soc_11G00	5
Soc_15G00	3
Soc_7G000	1
Soc_18G00	1
Soc_3G000	2
Soc_13G00	1
Soc_13G00	1
Soc_15G00	2
Soc_18G00	2
Soc_2G000	1
Soc_13G00	1
Soc_15G00	1
Soc_11G00	2
Soc_20G00	2
Soc_6G000	2
Soc_13G00	1
Soc_11G00	1
Soc_15G00	1
Soc_23G00	1
Soc_14G00	3
Soc_22G00	1
Soc_15G00	1
Soc_7G000	1
Soc_6G000	1
Soc_11G00	9
Soc_13G00	5
Soc_15G00	11
Soc_13G00	10
Soc_15G00	4
Soc_21G00	10
Soc_13G00	7
Soc_6G001	4
Soc_11G00	5
Soc_11G00	9
Soc_15G00	5
Soc_20G00	6
Soc_11G00	7
Soc_15G00	3

Soc_12G0(	2
Soc_14G0(	3
Soc_14G0(	3
Soc_14G0(	13
Soc_14G0(	6
Soc_13G0(	9
Soc_18G0(	6
Soc_13G0(	5
Soc_14G0(	14
Soc_12G0(	3
Soc_11G0(	6
Soc_5G000	2
Soc_11G0(	3
Soc_13G0(	6
Soc_20G0(	2
Soc_14G0(	8
Soc_20G0(	2
Soc_9G000	2
Soc_22G0(	3
Soc_12G0(	8
Soc_21G0(	1
Soc_15G0(	3
Soc_15G0(	2
Soc_14G0(	4
Soc_14G0(	3
Soc_11G0(	5
Soc_22G0(	1
Soc_11G0(	2
Soc_20G0(	3
Soc_11G0(	9
Soc_20G0(	3
Soc_6G001	2
Soc_13G0(	2
Soc_11G0(	4
Soc_11G0(	6
Soc_11G0(	7
Soc_14G0(	4
Soc_20G0(	1
Soc_11G0(	8
Soc_13G0(	4
Soc_13G0(	3
Soc_15G0(	2
Soc_4G001	1
Soc_6G000	1
Soc_11G0(	3
Soc_15G0(	2
Soc_22G0(	1
Soc_4G000	5
Soc_1G001	3
Soc_14G0(	5
Soc_17G0(	1
Soc_14G0(	4
Soc_11G0(	3
Soc_9G000	1
Soc_20G0(	1
Soc_14G0(	3
Soc_13G0(	2
Soc_20G0(	2

Soc_5G000	2
Soc_18G00	3
Soc_14G00	3
Soc_14G00	6
Soc_11G00	4
Soc_20G00	1
Soc_1G001	4
Soc_22G00	1
Soc_13G00	6
Soc_17G00	1
Soc_13G00	6
Soc_22G00	5
Soc_12G00	2
Soc_13G00	3
Soc_8G000	1
Soc_13G00	5
Soc_22G00	1
Soc_6G001	1
Soc_13G00	2
Soc_18G00	2
Soc_13G00	2
Soc_12G00	1
Soc_14G00	5
Soc_17G00	2
Soc_7G000	1
Soc_10G00	1
Soc_1G001	3
Soc_11G00	3
Soc_11G00	3
Soc_1G001	4
Soc_13G00	4
Soc_13G00	4
Soc_15G00	3
Soc_12G00	2
Soc_14G00	5
Soc_12G00	1
Soc_12G00	1
Soc_11G00	3
Soc_11G00	7
Soc_22G00	1
Soc_11G00	1
Soc_13G00	2
Soc_5G000	2
Soc_18G00	1
Soc_15G00	2
Soc_14G00	2
Soc_20G00	1
Soc_17G00	1
Soc_14G00	7
Soc_14G00	4
Soc_12G00	1
Soc_22G00	1
Soc_5G000	3
Soc_5G000	2
Soc_11G00	4
Soc_15G00	3
Soc_3G000	4
Soc_3G000	2

Soc_5G000	3
Soc_15G00	1
Soc_16G00	5
Soc_22G00	3
Soc_14G00	3
Soc_15G00	2
Soc_11G00	5
Soc_12G00	1
Soc_14G00	2
Soc_14G00	3
Soc_8G000	1
Soc_12G00	2
Soc_5G000	1
Soc_12G00	1
Soc_13G00	2
Soc_17G00	3
Soc_11G00	4
Soc_13G00	3
Soc_4G000	2
Soc_14G00	8
Soc_1G001	1
Soc_8G000	1
Soc_13G00	3
Soc_11G00	4
Soc_11G00	2
Soc_15G00	2
Soc_13G00	5
Soc_13G00	2
Soc_15G00	2
Soc_13G00	4
Soc_11G00	8
Soc_18G00	2
Soc_15G00	4
Soc_20G00	4
Soc_1G001	3
Soc_1G001	2
Soc_14G00	3
Soc_15G00	3
Soc_13G00	1
Soc_13G00	2
Soc_14G00	2
Soc_10G00	7
Soc_8G000	1
Soc_15G00	2
Soc_13G00	2
Soc_6G001	2
Soc_1G001	1
Soc_15G00	2
Soc_11G00	6
Soc_15G00	2
Soc_3G000	1
Soc_14G00	3
Soc_14G00	5
Soc_11G00	2
Soc_18G00	1
Soc_7G000	1
Soc_13G00	2
Soc_17G00	1

Soc_21G00	3
Soc_13G00	2
Soc_15G00	3
Soc_11G00	3
Soc_17G00	1
Soc_11G00	5
Soc_11G00	5
Soc_13G00	3
Soc_5G000	3
Soc_4G000	1
Soc_20G00	1
Soc_13G00	1
Soc_5G000	3
Soc_10G00	1
Soc_20G00	1
Soc_11G00	7
Soc_13G00	3
Soc_11G00	3
Soc_13G00	1
Soc_11G00	4
Soc_13G00	1
Soc_21G00	1
Soc_7G000	2
Soc_16G00	1
Soc_15G00	1
Soc_13G00	4
Soc_18G00	1
Soc_8G000	1
Soc_11G00	2
Soc_3G000	3
Soc_11G00	4
Soc_8G000	1
Soc_24G00	2
Soc_15G00	2
Soc_13G00	1
Soc_11G00	1
Soc_8G000	1
Soc_15G00	2
Soc_14G00	1
Soc_11G00	2
Soc_8G000	1
Soc_11G00	4
Soc_21G00	2
Soc_8G000	1
Soc_8G000	1
Soc_11G00	1
Soc_9G000	1
Soc_11G00	2
Soc_8G000	1
Soc_14G00	3
Soc_8G000	1
Soc_5G000	2
Soc_15G00	1
Soc_14G00	1
Soc_3G000	2
Soc_15G00	1
Soc_14G00	4
Soc_17G00	1

Soc_20G00	3
Soc_3G000	1
Soc_8G000	1
Soc_13G00	2
Soc_18G00	1
Soc_8G000	1
Soc_20G00	2
Soc_8G000	1
Soc_23G00	1
Soc_14G00	1
Soc_8G000	1
Soc_6G001	1
Soc_15G00	2
Soc_22G00	2
Soc_8G000	1
Soc_13G00	1
Soc_12G00	4
Soc_13G00	10
Soc_11G00	8
Soc_13G00	4
Soc_18G00	3
Soc_11G00	8
Soc_21G00	3
Soc_11G00	13
Soc_13G00	8
Soc_14G00	5
Soc_15G00	2
Soc_15G00	7
Soc_11G00	4
Soc_13G00	4
Soc_16G00	6
Soc_18G00	5
Soc_17G00	6
Soc_13G00	4
Soc_18G00	3
Soc_13G00	6
Soc_11G00	9
Soc_15G00	4
Soc_13G00	2
Soc_12G00	9
Soc_16G00	6
Soc_15G00	9
Soc_15G00	16
Soc_15G00	4
Soc_13G00	4
Soc_15G00	6
Soc_10G00	2
Soc_13G00	7
Soc_20G00	8
Soc_13G00	3
Soc_11G00	8
Soc_13G00	4
Soc_11G00	7
Soc_20G00	2
Soc_15G00	5
Soc_13G00	4
Soc_13G00	5
Soc_13G00	4

Soc_11G0(	6
Soc_23G0(	2
Soc_13G0(	4
Soc_15G0(	9
Soc_8G000	2
Soc_13G0(	4
Soc_15G0(	11
Soc_13G0(	5
Soc_18G0(	6
Soc_13G0(	2
Soc_13G0(	5
Soc_15G0(	10
Soc_18G0(	4
Soc_13G0(	2
Soc_13G0(	2
Soc_13G0(	2
Soc_18G0(	5
Soc_11G0(	6
Soc_15G0(	11
Soc_11G0(	5
Soc_11G0(	8
Soc_19G0(	1
Soc_23G0(	4
Soc_11G0(	7
Soc_15G0(	6
Soc_11G0(	4
Soc_13G0(	2
Soc_11G0(	5
Soc_13G0(	5
Soc_13G0(	8
Soc_11G0(	8
Soc_13G0(	7
Soc_1G000	1
Soc_13G0(	4
Soc_18G0(	3
Soc_10G0(	3
Soc_18G0(	6
Soc_1G001	3
Soc_15G0(	5
Soc_15G0(	12
Soc_16G0(	3
Soc_10G0(	4
Soc_15G0(	5
Soc_11G0(	5
Soc_13G0(	3
Soc_1G000	3
Soc_10G0(	2
Soc_15G0(	3
Soc_12G0(	6
Soc_13G0(	4
Soc_13G0(	2
Soc_15G0(	3
Soc_18G0(	5
Soc_8G000	2
Soc_18G0(	2
Soc_17G0(	1
Soc_1G001	3
Soc_10G0(	2

Soc_18G0(	4
Soc_20G0(	6
Soc_13G0(	6
Soc_11G0(	6
Soc_13G0(	7
Soc_22G0(	2
Soc_19G0(	3
Soc_23G0(	3
Soc_18G0(	6
Soc_22G0(	1
Soc_13G0(	9
Soc_15G0(	4
Soc_23G0(	3
Soc_11G0(	8
Soc_15G0(	4
Soc_15G0(	5
Soc_22G0(	1
Soc_18G0(	2
Soc_11G0(	6
Soc_18G0(	6
Soc_18G0(	4
Soc_15G0(	4
Soc_18G0(	5
Soc_12G0(	3
Soc_20G0(	1
Soc_13G0(	2
Soc_18G0(	2
Soc_14G0(	6
Soc_11G0(	11
Soc_20G0(	1
Soc_15G0(	5
Soc_15G0(	6
Soc_1G000	3
Soc_16G0(	4
Soc_11G0(	5
Soc_19G0(	5
Soc_11G0(	3
Soc_15G0(	3
Soc_18G0(	2
Soc_15G0(	4
Soc_23G0(	1
Soc_17G0(	1
Soc_15G0(	4
Soc_13G0(	7
Soc_22G0(	1
Soc_23G0(	1
Soc_18G0(	4
Soc_18G0(	5
Soc_13G0(	6
Soc_15G0(	6
Soc_5G000	1
Soc_12G0(	2
Soc_13G0(	1
Soc_3G000	1
Soc_11G0(	5
Soc_18G0(	3
Soc_5G000	1
Soc_12G0(	1

Soc_6G000	1
Soc_19G00	2
Soc_23G00	1
Soc_19G00	5
Soc_4G000	3
Soc_1G000	2
Soc_13G00	3
Soc_15G00	3
Soc_13G00	3
Soc_16G00	3
Soc_10G00	2
Soc_18G00	1
Soc_10G00	2
Soc_1G000	2
Soc_15G00	3
Soc_20G00	1
Soc_13G00	6
Soc_18G00	3
Soc_23G00	4
Soc_20G00	1
Soc_4G000	1
Soc_22G00	2
Soc_15G00	2
Soc_15G00	3
Soc_15G00	2
Soc_23G00	4
Soc_17G00	1
Soc_18G00	1
Soc_18G00	5
Soc_18G00	2
Soc_11G00	6
Soc_11G00	1
Soc_17G00	1
Soc_14G00	5
Soc_18G00	2
Soc_23G00	1
Soc_8G000	1
Soc_18G00	4
Soc_4G000	1
Soc_6G001	1
Soc_16G00	1
Soc_18G00	4
Soc_8G000	1
Soc_11G00	4
Soc_18G00	3
Soc_18G00	2
Soc_5G000	1
Soc_19G00	1
Soc_10G00	6
Soc_18G00	5
Soc_13G00	3
Soc_18G00	5
Soc_22G00	4
Soc_15G00	2
Soc_13G00	3
Soc_15G00	2
Soc_11G00	5
Soc_16G00	2

Soc_15G00	1
Soc_8G000	1
Soc_16G00	2
Soc_23G00	1
Soc_6G001	1
Soc_23G00	1
Soc_7G000	1
Soc_11G00	6
Soc_5G000	2
Soc_7G000	1
Soc_15G00	4
Soc_4G000	2
Soc_23G00	1
Soc_18G00	2
Soc_23G00	1
Soc_18G00	2
Soc_11G00	6
Soc_5G000	1
Soc_15G00	2
Soc_1G000	1
Soc_11G00	2
Soc_15G00	3
Soc_21G00	2
Soc_15G00	3
Soc_12G00	1
Soc_15G00	3
Soc_18G00	2
Soc_20G00	1
Soc_18G00	1
Soc_11G00	4
Soc_9G000	1
Soc_22G00	2
Soc_15G00	4
Soc_11G00	5
Soc_20G00	1
Soc_22G00	1
Soc_15G00	2
Soc_15G00	1
Soc_8G000	1
Soc_7G000	1
Soc_8G000	1
Soc_20G00	4
Soc_8G000	1
Soc_24G00	1
Soc_5G000	1
Soc_18G00	2
Soc_24G00	1
Soc_18G00	2
Soc_11G00	1
Soc_8G000	1
Soc_15G00	2
Soc_19G00	1
Soc_18G00	2
Soc_8G000	1
Soc_3G000	2
Soc_21G00	1
Soc_18G00	3
Soc_22G00	1

Soc_11G00	2
Soc_18G00	1
Soc_3G000	1
Soc_18G00	1
Soc_12G00	5
Soc_8G000	1
Soc_11G00	4
Soc_15G00	3
Soc_10G00	4
Soc_23G00	1
Soc_11G00	7
Soc_11G00	20
Soc_11G00	7
Soc_16G00	6
Soc_21G00	7
Soc_21G00	7
Soc_21G00	7
Soc_13G00	8
Soc_13G00	7
Soc_8G000	4
Soc_2G001	6
Soc_12G00	4
Soc_13G00	10
Soc_21G00	8
Soc_11G00	12
Soc_1G000	6
Soc_10G00	7
Soc_10G00	15
Soc_13G00	5
Soc_13G00	8
Soc_13G00	7
Soc_13G00	7
Soc_11G00	10
Soc_5G000	3
Soc_10G00	10
Soc_19G00	8
Soc_11G00	5
Soc_18G00	3
Soc_13G00	11
Soc_13G00	9
Soc_11G00	4
Soc_21G00	5
Soc_11G00	12
Soc_15G00	4
Soc_16G00	8
Soc_16G00	3
Soc_6G000	2
Soc_11G00	2
Soc_13G00	11
Soc_10G00	12
Soc_6G000	2
Soc_11G00	3
Soc_10G00	14
Soc_10G00	5
Soc_18G00	5
Soc_10G00	2
Soc_10G00	16
Soc_18G00	5

Soc_10G00	9
Soc_16G00	3
Soc_16G00	7
Soc_13G00	3
Soc_13G00	6
Soc_10G00	4
Soc_22G00	2
Soc_11G00	9
Soc_10G00	12
Soc_11G00	10
Soc_13G00	9
Soc_16G00	10
Soc_4G001	4
Soc_5G000	2
Soc_11G00	2
Soc_13G00	7
Soc_14G00	6
Soc_16G00	9
Soc_10G00	2
Soc_13G00	4
Soc_16G00	7
Soc_13G00	5
Soc_2G001	6
Soc_22G00	7
Soc_10G00	3
Soc_22G00	1
Soc_10G00	14
Soc_10G00	16
Soc_11G00	4
Soc_11G00	6
Soc_11G00	9
Soc_18G00	2
Soc_10G00	12
Soc_16G00	3
Soc_10G00	5
Soc_11G00	2
Soc_10G00	3
Soc_10G00	6
Soc_10G00	11
Soc_10G00	9
Soc_10G00	3
Soc_11G00	7
Soc_18G00	6
Soc_15G00	1
Soc_11G00	6
Soc_4G001	2
Soc_14G00	5
Soc_13G00	5
Soc_14G00	4
Soc_15G00	2
Soc_16G00	3
Soc_11G00	7
Soc_18G00	4
Soc_12G00	3
Soc_18G00	3
Soc_6G000	2
Soc_4G001	2
Soc_16G00	4

Soc_4G001	2
Soc_2G001	3
Soc_11G00	4
Soc_10G00	4
Soc_17G00	4
Soc_11G00	8
Soc_10G00	6
Soc_1G001	1
Soc_19G00	2
Soc_18G00	7
Soc_10G00	3
Soc_11G00	9
Soc_21G00	3
Soc_13G00	4
Soc_18G00	5
Soc_6G000	2
Soc_10G00	6
Soc_13G00	3
Soc_10G00	4
Soc_16G00	3
Soc_11G00	4
Soc_2G001	1
Soc_18G00	4
Soc_11G00	7
Soc_16G00	4
Soc_10G00	4
Soc_18G00	6
Soc_14G00	3
Soc_11G00	4
Soc_18G00	2
Soc_13G00	2
Soc_10G00	3
Soc_11G00	1
Soc_13G00	3
Soc_2G000	1
Soc_18G00	2
Soc_11G00	4
Soc_15G00	4
Soc_11G00	9
Soc_10G00	3
Soc_2G001	2
Soc_1G000	1
Soc_13G00	7
Soc_2G001	3
Soc_18G00	4
Soc_14G00	3
Soc_10G00	8
Soc_1G000	1
Soc_2G000	1
Soc_11G00	5
Soc_21G00	4
Soc_11G00	2
Soc_2G001	1
Soc_10G00	5
Soc_10G00	6
Soc_5G000	3
Soc_10G00	4
Soc_13G00	9

Soc_11G00	2
Soc_10G00	12
Soc_2G001	1
Soc_10G00	1
Soc_8G000	1
Soc_6G000	2
Soc_10G00	5
Soc_10G00	3
Soc_12G00	5
Soc_7G000	1
Soc_24G00	2
Soc_11G00	3
Soc_11G00	4
Soc_13G00	11
Soc_11G00	1
Soc_7G000	1
Soc_1G000	2
Soc_11G00	1
Soc_11G00	1
Soc_10G00	9
Soc_7G000	1
Soc_22G00	1
Soc_18G00	1
Soc_16G00	2
Soc_18G00	5
Soc_19G00	1
Soc_17G00	2
Soc_10G00	3
Soc_18G00	3
Soc_1G000	2
Soc_13G00	5
Soc_18G00	4
Soc_18G00	2
Soc_10G00	3
Soc_18G00	2
Soc_11G00	4
Soc_2G001	1
Soc_18G00	1
Soc_11G00	4
Soc_4G001	3
Soc_18G00	2
Soc_7G000	3
Soc_18G00	2
Soc_21G00	2
Soc_18G00	3
Soc_17G00	3
Soc_2G000	1
Soc_11G00	1
Soc_12G00	2
Soc_13G00	3
Soc_18G00	1
Soc_11G00	2
Soc_11G00	3
Soc_18G00	3
Soc_10G00	10
Soc_11G00	3
Soc_11G00	1
Soc_18G00	7

Soc_11G0(	6
Soc_1G002	3
Soc_21G0(	4
Soc_10G0(	4
Soc_10G0(	3
Soc_13G0(	2
Soc_14G0(	4
Soc_10G0(	5
Soc_7G000	2
Soc_18G0(	2
Soc_13G0(	2
Soc_18G0(	2
Soc_11G0(	6
Soc_2G000	1
Soc_11G0(	2
Soc_18G0(	2
Soc_21G0(	1
Soc_18G0(	3
Soc_18G0(	2
Soc_11G0(	2
Soc_18G0(	2
Soc_18G0(	2
Soc_11G0(	6
Soc_4G001	2
Soc_11G0(	2
Soc_9G000	1
Soc_11G0(	1
Soc_18G0(	3
Soc_10G0(	1
Soc_18G0(	4
Soc_10G0(	3
Soc_3G000	1
Soc_21G0(	2
Soc_9G000	1
Soc_6G000	1
Soc_13G0(	3
Soc_1G000	2
Soc_21G0(	3
Soc_23G0(	1
Soc_14G0(	3
Soc_21G0(	1
Soc_1G001	3
Soc_11G0(	3
Soc_11G0(	1
Soc_10G0(	3
Soc_11G0(	3
Soc_18G0(	2
Soc_1G000	1
Soc_21G0(	1
Soc_21G0(	2
Soc_18G0(	1
Soc_5G000	2
Soc_11G0(	2
Soc_11G0(	2
Soc_18G0(	3
Soc_11G0(	1
Soc_18G0(	1
Soc_9G000	1

Soc_11G0(	6
Soc_11G0(	8
Soc_6G000	4
Soc_11G0(	5
Soc_5G000	4
Soc_10G0(	14
Soc_11G0(	10
Soc_4G000	6
Soc_5G000	4
Soc_11G0(	12
Soc_11G0(	12
Soc_15G0(	4
Soc_18G0(	6
Soc_5G000	4
Soc_5G000	4
Soc_5G000	4
Soc_5G000	4
Soc_11G0(	6
Soc_5G000	3
Soc_15G0(	9
Soc_6G000	3
Soc_11G0(	12
Soc_15G0(	9
Soc_1G002	2
Soc_15G0(	6
Soc_11G0(	7
Soc_10G0(	4
Soc_11G0(	6
Soc_15G0(	3
Soc_11G0(	9
Soc_15G0(	6
Soc_13G0(	6
Soc_13G0(	5
Soc_1G002	7
Soc_6G000	3
Soc_4G000	10
Soc_5G000	4
Soc_11G0(	3
Soc_17G0(	4
Soc_2G000	2
Soc_4G000	2
Soc_18G0(	3
Soc_17G0(	6
Soc_4G000	1
Soc_11G0(	5
Soc_11G0(	4
Soc_4G000	2
Soc_19G0(	5
Soc_13G0(	4
Soc_18G0(	7
Soc_4G000	2
Soc_13G0(	5
Soc_13G0(	6
Soc_15G0(	1
Soc_11G0(	7
Soc_10G0(	7
Soc_15G0(	8
Soc_1G001	5

Soc_11G0(	8
Soc_15G0(	2
Soc_6G000	2
Soc_15G0(	1
Soc_10G0(	4
Soc_13G0(	5
Soc_10G0(	6
Soc_6G000	3
Soc_10G0(	6
Soc_11G0(	5
Soc_13G0(	8
Soc_10G0(	4
Soc_6G000	1
Soc_11G0(	7
Soc_11G0(	7
Soc_11G0(	5
Soc_13G0(	1
Soc_19G0(	5
Soc_13G0(	10
Soc_11G0(	5
Soc_13G0(	5
Soc_17G0(	2
Soc_13G0(	2
Soc_11G0(	5
Soc_18G0(	3
Soc_18G0(	3
Soc_1G001	1
Soc_6G000	1
Soc_13G0(	3
Soc_13G0(	2
Soc_15G0(	1
Soc_15G0(	3
Soc_15G0(	4
Soc_19G0(	4
Soc_18G0(	1
Soc_15G0(	4
Soc_11G0(	4
Soc_12G0(	2
Soc_11G0(	3
Soc_13G0(	4
Soc_23G0(	1
Soc_15G0(	1
Soc_15G0(	3
Soc_1G002	2
Soc_13G0(	5
Soc_17G0(	2
Soc_7G000	2
Soc_13G0(	3
Soc_11G0(	6
Soc_23G0(	2
Soc_19G0(	3
Soc_17G0(	5
Soc_10G0(	9
Soc_20G0(	2
Soc_14G0(	7
Soc_15G0(	12
Soc_17G0(	3
Soc_10G0(	5

Soc_13G00	3
Soc_13G00	1
Soc_6G000	1
Soc_6G000	1
Soc_6G000	1
Soc_23G00	2
Soc_15G00	3
Soc_10G00	8
Soc_13G00	1
Soc_8G000	1
Soc_17G00	2
Soc_11G00	4
Soc_18G00	10
Soc_13G00	5
Soc_17G00	3
Soc_6G000	1
Soc_9G000	1
Soc_15G00	1
Soc_11G00	6
Soc_15G00	3
Soc_15G00	4
Soc_11G00	3
Soc_13G00	1
Soc_15G00	5
Soc_11G00	7
Soc_13G00	2
Soc_7G000	2
Soc_23G00	1
Soc_13G00	4
Soc_7G000	2
Soc_15G00	6
Soc_18G00	1
Soc_13G00	2
Soc_5G000	2
Soc_19G00	4
Soc_13G00	3
Soc_16G00	2
Soc_11G00	4
Soc_17G00	4
Soc_18G00	2
Soc_15G00	4
Soc_17G00	2
Soc_10G00	8
Soc_18G00	2
Soc_13G00	1
Soc_11G00	4
Soc_15G00	1
Soc_13G00	1
Soc_18G00	1
Soc_10G00	4
Soc_18G00	7
Soc_19G00	5
Soc_19G00	3
Soc_13G00	3
Soc_18G00	2
Soc_19G00	1
Soc_13G00	2
Soc_13G00	4

Soc_22G00	4
Soc_15G00	3
Soc_15G00	6
Soc_15G00	1
Soc_11G00	2
Soc_15G00	2
Soc_15G00	4
Soc_18G00	3
Soc_11G00	5
Soc_13G00	4
Soc_11G00	4
Soc_11G00	5
Soc_14G00	1
Soc_13G00	4
Soc_7G000	2
Soc_6G000	1
Soc_10G00	9
Soc_17G00	6
Soc_4G000	2
Soc_11G00	2
Soc_11G00	4
Soc_13G00	2
Soc_13G00	2
Soc_18G00	3
Soc_4G000	1
Soc_10G00	1
Soc_15G00	1
Soc_15G00	2
Soc_13G00	2
Soc_14G00	3
Soc_18G00	2
Soc_17G00	1
Soc_3G000	1
Soc_18G00	4
Soc_11G00	2
Soc_18G00	2
Soc_11G00	8
Soc_6G000	1
Soc_13G00	1
Soc_15G00	1
Soc_7G000	1
Soc_7G000	1
Soc_9G000	1
Soc_11G00	4
Soc_13G00	4
Soc_15G00	4
Soc_13G00	2
Soc_17G00	2
Soc_13G00	1
Soc_5G000	2
Soc_10G00	1
Soc_6G000	1
Soc_13G00	2
Soc_22G00	1
Soc_10G00	7
Soc_10G00	1
Soc_6G000	1
Soc_18G00	2

Soc_18G00	3
Soc_6G000	1
Soc_5G000	2
Soc_5G000	1
Soc_17G00	1
Soc_1G000	1
Soc_18G00	1
Soc_10G00	4
Soc_17G00	5
Soc_15G00	1
Soc_9G000	1
Soc_17G00	4
Soc_18G00	1
Soc_22G00	2
Soc_8G000	1
Soc_18G00	2
Soc_11G00	1
Soc_13G00	4
Soc_5G000	2
Soc_18G00	1
Soc_18G00	1
Soc_22G00	2
Soc_11G00	1
Soc_13G00	1
Soc_22G00	2
Soc_11G00	2
Soc_18G00	1
Soc_8G000	1
Soc_13G00	5
Soc_18G00	2
Soc_4G000	1
Soc_9G000	1
Soc_5G000	1
Soc_8G000	4
Soc_15G00	12
Soc_10G00	5
Soc_12G00	4
Soc_20G00	10
Soc_20G00	11
Soc_11G00	8
Soc_15G00	4
Soc_10G00	8
Soc_10G00	10
Soc_10G00	3
Soc_17G00	4
Soc_1G001	4
Soc_15G00	4
Soc_10G00	3
Soc_11G00	7
Soc_12G00	4
Soc_13G00	3
Soc_11G00	7
Soc_10G00	14
Soc_9G000	2
Soc_21G00	3
Soc_5G000	2
Soc_10G00	6
Soc_9G000	2

Soc_11G00	2
Soc_21G00	3
Soc_15G00	2
Soc_15G00	4
Soc_15G00	3
Soc_10G00	5
Soc_15G00	2
Soc_8G000	2
Soc_11G00	5
Soc_10G00	9
Soc_4G000	2
Soc_21G00	2
Soc_3G000	1
Soc_1G001	3
Soc_21G00	3
Soc_11G00	1
Soc_1G001	2
Soc_10G00	5
Soc_10G00	8
Soc_10G00	1
Soc_1G001	2
Soc_15G00	10
Soc_22G00	5
Soc_11G00	2
Soc_11G00	2
Soc_15G00	2
Soc_15G00	13
Soc_13G00	7
Soc_1G001	2
Soc_10G00	5
Soc_19G00	5
Soc_20G00	1
Soc_15G00	2
Soc_10G00	11
Soc_4G001	1
Soc_11G00	1
Soc_10G00	3
Soc_20G00	4
Soc_15G00	5
Soc_10G00	6
Soc_1G000	4
Soc_15G00	7
Soc_15G00	2
Soc_15G00	3
Soc_22G00	1
Soc_21G00	3
Soc_10G00	11
Soc_22G00	1
Soc_20G00	1
Soc_10G00	9
Soc_18G00	8
Soc_11G00	2
Soc_12G00	3
Soc_10G00	11
Soc_10G00	3
Soc_6G000	1
Soc_20G00	1
Soc_3G000	1

Soc_6G000	1
Soc_3G000	3
Soc_11G00	1
Soc_1G001	3
Soc_8G000	1
Soc_6G000	1
Soc_10G00	2
Soc_1G001	2
Soc_1G001	4
Soc_11G00	3
Soc_10G00	3
Soc_13G00	5
Soc_21G00	1
Soc_9G000	2
Soc_18G00	2
Soc_10G00	2
Soc_20G00	3
Soc_11G00	6
Soc_10G00	5
Soc_10G00	2
Soc_13G00	1
Soc_10G00	1
Soc_10G00	3
Soc_8G000	1
Soc_8G000	1
Soc_15G00	3
Soc_15G00	3
Soc_15G00	3
Soc_11G00	3
Soc_11G00	3
Soc_15G00	1
Soc_10G00	2
Soc_1G001	4
Soc_13G00	4
Soc_11G00	1
Soc_10G00	7
Soc_10G00	2
Soc_5G000	2
Soc_11G00	7
Soc_8G000	3
Soc_21G00	2
Soc_10G00	3
Soc_20G00	5
Soc_12G00	1
Soc_15G00	3
Soc_11G00	1
Soc_15G00	5
Soc_15G00	1
Soc_21G00	1
Soc_18G00	6
Soc_10G00	2
Soc_10G00	1
Soc_10G00	2
Soc_10G00	4
Soc_11G00	2
Soc_13G00	3
Soc_10G00	5
Soc_11G00	2

Soc_18G00	2
Soc_20G00	5
Soc_10G00	5
Soc_5G000	1
Soc_1G001	1
Soc_17G00	1
Soc_7G000	1
Soc_11G00	4
Soc_15G00	4
Soc_5G000	3
Soc_15G00	1
Soc_10G00	2
Soc_18G00	3
Soc_10G00	5
Soc_18G00	4
Soc_21G00	1
Soc_10G00	2
Soc_10G00	6
Soc_8G000	1
Soc_21G00	1
Soc_15G00	2
Soc_5G000	1
Soc_10G00	2
Soc_4G000	3
Soc_10G00	2
Soc_18G00	3
Soc_10G00	3
Soc_10G00	3
Soc_10G00	3
Soc_10G00	2
Soc_10G00	8
Soc_10G00	2
Soc_21G00	3
Soc_6G000	1
Soc_10G00	5
Soc_7G000	1
Soc_10G00	3
Soc_11G00	4
Soc_11G00	2
Soc_11G00	3
Soc_6G000	1
Soc_10G00	1
Soc_11G00	2
Soc_1G000	1
Soc_20G00	1
Soc_11G00	1
Soc_18G00	2
Soc_8G000	1
Soc_10G00	5
Soc_1G001	2
Soc_11G00	3
Soc_6G000	1
Soc_11G00	3
Soc_11G00	2
Soc_1G000	1
Soc_10G00	3
Soc_20G00	1
Soc_20G00	1

Soc_12G00	3
Soc_10G00	2
Soc_13G00	3
Soc_10G00	3
Soc_6G000	1
Soc_5G000	1
Soc_15G00	1
Soc_5G000	1
Soc_11G00	3
Soc_15G00	1
Soc_13G00	2
Soc_18G00	1
Soc_8G000	1
Soc_10G00	3
Soc_1G001	1
Soc_6G000	1
Soc_10G00	2
Soc_15G00	1
Soc_5G000	3
Soc_10G00	4
Soc_15G00	2
Soc_5G000	1
Soc_8G000	1
Soc_11G00	1
Soc_5G000	1
Soc_13G00	2
Soc_4G000	1
Soc_10G00	2
Soc_15G00	2
Soc_1G002	3
Soc_15G00	3
Soc_5G000	1
Soc_9G000	1
Soc_15G00	1
Soc_10G00	2
Soc_10G00	1
Soc_1G001	1
Soc_8G000	1
Soc_5G000	2
Soc_15G00	1
Soc_22G00	1
Soc_10G00	4
Soc_15G00	1
Soc_6G000	1
Soc_15G00	1
Soc_15G00	1
Soc_3G000	1
Soc_3G000	1
Soc_10G00	3
Soc_15G00	2
Soc_10G00	4
Soc_15G00	1
Soc_20G00	2
Soc_5G000	1
Soc_2G000	1
Soc_8G000	2
Soc_1G000	1
Soc_15G00	1

Soc_11G0(	2
Soc_24G0(	1
Soc_10G0(	1
Soc_15G0(	1
Soc_8G000	2
Soc_10G0(	1
Soc_5G000	1
Soc_13G0(	1
Soc_1G000	1
